# Efficient Synthesis of Boron-Containing α-Acyloxyamide Analogs via Microwave Irradiation

**DOI:** 10.3390/molecules18089488

**Published:** 2013-08-08

**Authors:** Chih-Cheng Chai, Pin-Yi Liu, Chia-Hung Lin, Hsien-Chi Chen, Yang-Chang Wu, Fang-Rong Chang, Po-Shen Pan

**Affiliations:** 1 Department of Chemistry, Tamkang University, New Taipei 25137, Taiwan; 2 School of Pharmacy, College of Pharmacy, China Medical University, Taichung 404, Taiwan; 3 Chinese Medicine Research and Development Center, China Medical University, Taichung 404, Taiwan; 4 Center for Molecular Medicine, China Medical University Hospital, Taichung 404, Taiwan; 5 Graduate Institute of Natural Products, Kaohsiung Medical University, Kaohsiung 807, Taiwan; 6 Cancer Center, Kaohsiung Medical University Hospital, Kaohsiung 807, Taiwan

**Keywords:** boron, multicomponent reaction, Passerini reaction, HepG2

## Abstract

In this report, a Passerini three-component reaction utilizing boron-containing carboxylic acids or aldehydes is discussed. The reaction was carried out in water and facilitated by the use of microwave irradiation. This methodology allowed for the efficient formation of a broad range of boron-containing α-acyloxyamides under mild conditions within a short time. Two series of boron-containing α-acyloxyamides were synthesized and subsequently screened for cytotoxicity using the MTT cell viability assay. Two potential lead compounds were found to have potent activity against the HepG2 cancer cell line, demonstrating the potential of this methodology for use in the development of novel pharmaceuticals.

## 1. Introduction

**Figure 1:**
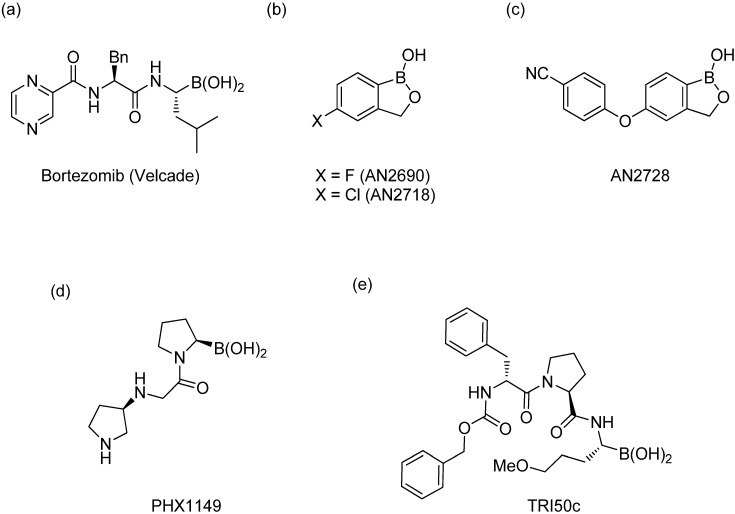
Boron-containing analogs as pharmaceutical agents.

Boron-based compounds possess a unique and potentially valuable feature, whereby the empty *p*-orbital on the boron atom is able to interact with a nucleophile from a biological target [1,2,3,4,5,6,7], forming a stable tetrahedral complex. This distinctive property provides great promise in the field of pharmaceuticals, as it allows boron-based compounds to react with a target of interest from a different perspective to their conventional carbon-based analogs. The FDA approval of bortezomib [Velcade^TM^, Figure 1a] in 2003 for the treatment of multiple myeloma and mantle cell lymphoma [8,9,10] represents one of the greatest examples of successful utilization of organoboron entities for treating human diseases. Encouraged by the success of bortezomib, several boron-containing molecules have since been developed to treat a wide range of diseases, a number of which are currently undergoing clinical trials (Figure 1b–e) [11,12,13,14,15,16,17].

Multicomponent reactions (MCRs) are convergent synthetic strategies in which three or more reagents are combined in one pot to produce the desired products [18,19]. It is an invaluable platform in the development of pharmaceutical agents as, owing to their versatility, MCRs are perfectly suited for producing boron-containing compounds for potential biological applications.

One of the most widely utilized MCRs is the isocyanide-based Passerini reaction, where an isocyanide, an aldehyde, and a carboxylic acid are condensed, generating α-acyloxyamides [20,21]. Although widely used by the organic and medicinal chemistry communities, the Passerini reaction has several shortcomings. One such drawback is that poor yields are often observed when a weakly acidic carboxylic acid [22] or no Lewis acid promoters [23] are involved in the reaction. In the last decades, tremendous progress has been made in the optimization of the Passerini reaction [24,25,26,27,28,29,30]. However, these studies mainly focused on improving the conditions for constructing molecules containing the elements carbon, hydrogen, oxygen, nitrogen, halogens, and sulfur. Reports on utilizing an MCR as a platform to generate boron-containing analogs are very limited, and those that have been published often demonstrate the requirement for a prolonged reaction time or excessive purification procedures [31]. Herein, we report the use of the Passerini reaction for the efficient synthesis of boron-containing α-acyloxyamides. This eco-friendly procedure could be carried out on a broad range of substrates at a moderate temperature under microwave irradiation conditions, with a reduced reaction time possible.

## 2. Results and Discussion

### 2.1. Chemistry

Initial experiments were carried out in order to determine the optimal conditions for the synthesis of boron-containing α-acyloxyamide analogs (Table 1). First, 1.0 equivalent of 4-carboxyphenylboronic acid pinacol ester (**1a**), 1.0 equivalent of benzaldehyde (**2**), and 1.0 equivalent of cyclohexyl isocyanide (**3**) were dissolved in the selected solvent and allowed to react at 45 °C for 90 min under microwave irradiation (150 W). It was found that while dichloromethane (entry 1) and methanol (entry 2) did not give desired product **A1**, using THF gave a moderate yield of 39% (entry 3). The yield was greatly improved to 77% when water was used as the solvent (entry 4). Increasing the reactant concentrations from 0.25 M to 1.0 M (entry 5) resulted in an increased isolated yield (84%), and extension of the reaction time from 90 min to 120 min (entry 6) gave an even higher yield (88%). In an attempt to improve the yield further, the temperature was increased to 55 °C for 120 min; however, rather than giving an increased amount of the desired product, a lower isolated yield was obtained (60%), suggesting product degradation due to overheating (entry 7). Thus, this series of optimization experiments highlighted a range of temperatures, reagent concentrations, and solvents that were effective for producing desired product **A1** in good isolated yields. The desired product was purified using a simple precipitation procedure with an appropriate solvent system, and no additional column chromatography or reverse-phase high performance liquid chromatography was required for affording satisfactory purity. This finding could be of particular importance for the construction of boron-containing libraries, as one of the main challenges in boron chemistry is the purification of the final product, with excessive purification protocols often required for achieving satisfactory purity.

**Table 1:** Optimization of Passerini reaction using boron-containing acid building block. 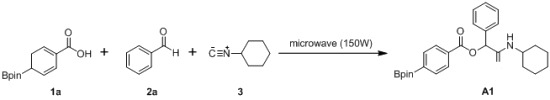

Entry	Temp. (°C)	Solvent	Conc. (M)	Time (min)	Yields (%)
1	45 °C	DCM	0.25	90	N.R.
2	45 °C	MeOH	0.25	90	N.R.
3	45 °C	THF	0.25	90	39
4	45 °C	H_2_O	0.25	90	77
5	45 °C	H_2_O	1	90	84
6	45 °C	H_2_O	1	120	88
7	55 °C	H_2_O	1	120	N.D. ^a^
8	55 °C	H_2_O	1	1day	69 ^b^

^a^ The decomposition of the product was observed; ^b^ Reaction performed without microwave irradiation.

Three carboxylphenylboronate esters **1a–c**, seven aldehydes **2a–g**, and cyclohexyl isocyanide (**3**) were then used to evaluate the scope of the reaction under the optimized conditions that were deduced from the results shown in Table 1. As demonstrated in Table 2, in all cases, the desired products **A1–20** were isolated in moderate to good yields, ranging from 56%–88%. It was found that the position of the boronate ester did not influence the reaction as 4- and 3-carboxyphenylboronic acid pinacol esters **1a** and **1b** (entries 1 and 2) gave the desired products **A1** and **A2** in good yields, respectively. Apart from the result shown in entry 3, attachment of an electron-withdrawing group to the acid building block appeared to impede the reaction, as analogs **A6**, **A9**, **A12**, **A15**, and **A18** were obtained in lower isolated yields. However, this trend was not observed when an electron-withdrawing group was attached to the aldehyde building block, as demonstrated by the good yields of analogs** A4–9** obtained. Further, the attachment of an electron-donating group to the aldehyde building block was also tolerated, with analogs **A10–15** achieved in good yields. Finally, as heteroaryl moieties are frequently found in many pharmaceutical agents [32,33,34,35], 3-pyridinecarboxaldehyde (**2f**), and furfural (**2g**) were used to construct the corresponding Passerini boronate esters. Five Passerini boronate esters containing heteroaryl motifs **A16–20** were successfully synthesized in moderate to good yields using the optimized microwave-assisted conditions.

**Table 2:** Synthesis of Passerini products with boron containing acid building blocks. 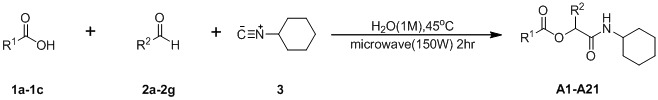

Entry	R^1^	R^2^	Product	Yields (%)
1	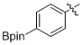 (1a)	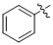 (2a)	**A1**	88
2	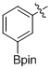 (1b)	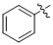	**A2**	83
3	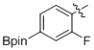 (1c)	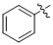	**A3**	80
4	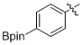	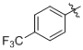 (2b)	**A4**	76
5	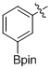	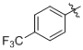	**A5**	79
6	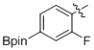	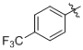	**A6**	51
7	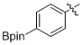	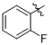 (2c)	**A7**	87
8	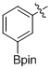	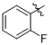	**A8**	86
9	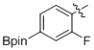	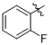	**A9**	68
10	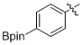	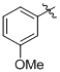 (2d)	**A10**	70
11	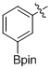	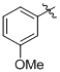	**A11**	77
12	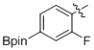	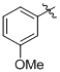	**A12**	63
13	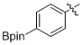	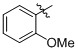 (e)	**A13**	87
14	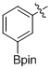	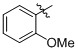	**A14**	79
15	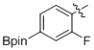	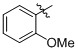	**A15**	63
16	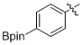	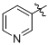 (f)	**A16**	56
17	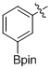	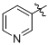	**A17**	52
18	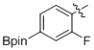	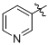	**A18**	56
19	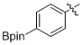	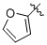 (g)	**A19**	57
20	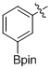	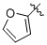	**A20**	63

Encouraged by the results presented in Table 2, experiments were initiated in order to explore the possibility of using boron-containing aldehyde building blocks to synthesize Passerini analogs (Table 3). Although they are structurally similar, the optimal conditions for preparing **A1** did not appear to be adequate for producing **B1**. For instance, the conditions of entries 4–6 in Table 1 were able to generate **A1** in 77%, 84%, and 88% yields, while only affording **B1** in 0%, 58%, and 65% (Table 3, entries 1–3). It was found that prolonged microwave irradiation was required to promote the reaction, with an improved yield of 75% achieved after 150 min of reaction time (entry 4). However, after an additional 60 min irradiation, the yield declined to 60% (entry 5), suggesting degradation of the product might have occurred under such extensive microwave treatment.

**Table 3:** Optimisation of Passerini reaction using boron-containing aldehyde building block. 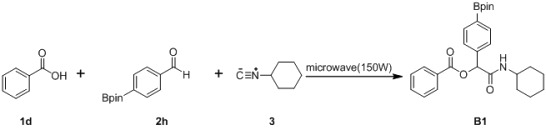

Entry	Temp. (°C)	Solvent	Conc. (M)	Time (min)	Yields (%)
1	45 °C	H_2_O	0.25	90	N.R.
2	45 °C	H_2_O	1	90	58
3	45 °C	H_2_O	1	120	65
4	45 °C	H_2_O	1	150	75
5	45 °C	H_2_O	1	210	60

After elucidating the optimal reaction conditions (Table 3, entry 4), six acid building blocks, two boron-containing aldehydes, and cyclohexyl isocyanide were used to synthesize **B1–12**. In all cases, the desired products were isolated in good yields, ranging from 50%–89% (Table 4). It was found that the reactions were greatly influenced by the nature of the acid building blocks. For instance, reactions with benzoic acid (**1d**) gave higher yields (entries 1 and 2, 69%–75%) than for those achieved for the compound with an electron-withdrawing group (entries 3 and 4, 57%–59%). Furthermore, acids with an electron-donating group generally gave even higher yields than those without (entries 5–10, 72%–89%). Incorporation of a heteroaryl motif to the Passerini product was successfully accomplished in moderate yields of 50%–55% by using pyrazinecarboxylic acid (**1i**) (entries 11 and 12).

**Table 4:** Synthesis of Passerini products with boron containing aldehyde building blocks. 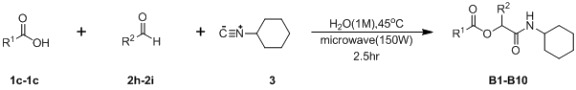

Entry	R^1^	R^2^	Product	Yields (%)
1	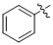 (1d)	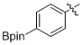 (2h)	**B1**	75
2	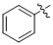	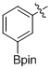 (2i)	**B2**	69
3	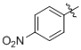 (1e)	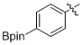	**B3**	57
4	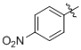	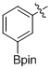	**B4**	59
5	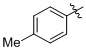 (1f)	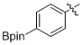	**B5**	72
6	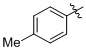	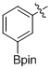	**B6**	63
7	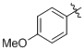 (1g)	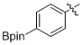	**B7**	80
8	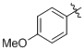	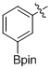	**B8**	82
9	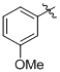 (1h)	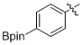	**B9**	85
10	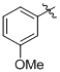	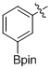	**B10**	89
11	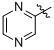 (1i)	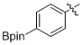	**B11**	51
12	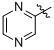	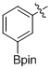	**B12**	42

### 2.2. *In Vitro* Biological Evaluation

The thirty three boron-containing α-acyloxyamides mentioned above were subsequently screened for anti-proliferative activity against the HepG2 (human hepatocellular carcinoma) cancer cell line using the MTT cell viability assay. It was found that **A4** and **A5** gave IC_50_ values of 33.6 μM and 27.5 μM, respectively, where non-boron analog **A21** was observed to be inactive. Further boron-containing α-acyloxyamides are currently being synthesized using the method developed in the present study, and their structure–activity relationships will be reported in due course.

## 3. Experimental

### 3.1. General

All starting materials were obtained from commercial suppliers and used without further purification unless otherwise noted. Reactions were performed on a CEM Co., Discover microwave reactor using sealed vessels. ^1^H-, ^13^C-, ^11^B-NMR spectra were recorded on a Bruker Avance 600 FT-NMR spectrometer at 600.13, 150.90, and 192.54 MHz, respectively. All ^11^B chemical shifts were referenced to external BF_3_·OEt_2_ (0.0 ppm). Data are represented as follows: chemical shifts (ppm), multiplicity (s = singlet, d = doublet, t = triplet, m = multiplet, br = broad), coupling constant J (Hz). Melting points were determined by using a Fargo MP-2D melting point apparatus and were uncorrected. High resolution ESI mass spectra were obtained by Finnigan MAT 95S. 

### 3.2. General Procedure A for the Synthesis of Boron-Containing α-Acyloxyl Amides **A1–21**


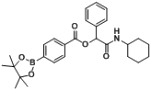

*2-(Cyclohexylamino)-2-oxo-1-phenylethyl4-(4,4,5,5-tetramethyl-1,3,2-dioxaborolan-2-yl)benzoate* (**A1**). A 10 mL glass tube containing the 4-carboxyphenylboronic acid ester (248 mg, 1.00 mmol), benzaldehyde (0.10 mL, 1.00 mmol), and D.I. H_2_O (1 mL) was first microwave irradiated for 6 min (45 °C, 150 W) under medium speed magnetic stirring. Cyclohexyl isocyanide (**3**, 0.124 mL, 1.00 mmol) was then added to the reaction mixture. The additional microwave irradiation was applied for 120 min (45 °C, 150 W) under medium speed magnetic stirring. After being diluted in dichloromethane, the resulted reaction mixture was washed twice with a saturated aqueous solution of NaHCO_3_ and with brine. The resulted organic layer was collected and dried over MgSO_4_ and concentrated *in vacuo*. The crude product was then dissolved in ethyl acetate (3 mL) prior the slow addition of *n*-hexane. The resulting precipitate was formed and collected by filtration affording the desired product in 88% yield. mp = 198 °C. ^1^H-NMR (CDCl_3_) δ: 8.06 (d, *J* = 7.8 Hz, 2H), 7.90 (d, *J* = 8.3 Hz, 2H), 7.53 (d, *J* = 7.2 Hz, 2H), 7.42–7.33 (m, 3H), 6.31 (s, 1H), 6.03 (br, 1H), 3.87–3.79 (m, 1H), 1.94 (d, *J* = 8.8 Hz, 1H), 1.91–1.85 (m, 1H), 1.72–1.61 (m, 2H), 1.61–1.56 (m, 3H), 1.41–1.31 (m, 14H), 1.23–1.08 (m, 3H). ^13^C-NMR (CDCl_3_) δ: 167.3, 164.9, 135.7, 134.9, 131.4, 128.9, 128.8, 128.7, 127.4, 84.3, 76.0, 48.1, 32.8, 25.4, 24.9, 24.6. ^11^B-NMR (CDCl_3_) δ: 31.0. HRMS (ESI, positive ion): *m/z* [M+H]^+^, found 464.2607. C_27_H_34_BNO_5_ requires 464.2606.


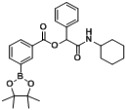

*2-(Cyclohexylamino)-2-oxo-1-phenylethyl3-(4,4,5,5-tetramethyl-1,3,2-dioxaborolan-2-yl)benzoate* (**A2**). The desired compound (384 mg, 83% yield) was prepared by General Procedure A using 3-carboxyphenyl boronic acid ester (248 mg, 1.00 mmol), benzaldehyde (0.102 mL, 1.00 mmol), and cyclohexyl isocyanide (0.124 mL, 1.00 mmol); mp = 152 °C. ^1^H-NMR (CDCl_3_) δ: 8.50 (s, 1H), 8.16 (d, *J* = 7.8 Hz, 1H), 8.03 (d, *J* = 7.4 Hz, 1H), 7.54 (d, *J* = 7.2 Hz, 2H), 7.48 (t, *J* = 7.5 Hz, 1H), 7.32–7.41 (m, 3H), 6.31 (s, 1H), 6.15 (br, 1H), 3.80–3.89 (m, 1H), 1.87–1.98 (m, 2H), 1.64–1.74 (m, 3H), 1.56–1.64 (m, 2H), 1.32–1.43 (m, 12H), 1.13–1.27 (m, 4H). ^13^C-NMR (CDCl_3_) δ: 167.4, 164.9, 139.8, 136.0, 132.4, 128.9, 128.7, 128.1, 127.4, 84.2, 76.0, 48.1, 32.8, 25.5, 24.8, 24.6. ^11^B-NMR (CDCl_3_) δ: 31.0. HRMS (ESI, positive ion): *m/z* [M+H]^+^, found 464.2583. C_27_H_34_BNO_5_ requires 464.2606.


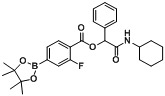

*2-(Cyclohexylamino)-2-oxo-1-phenylethyl2-fluoro-4-(4,4,5,5-tetramethyl-1,3,2-dioxaborolan-2-yl)benzoate* (**A3**). The desired compound (311 mg, 80% yield) was prepared by General Procedure A using 4-carboxy-3-fluorophenylboronic acid ester (266 mg, 1.00 mmol), benzaldehyde (0.102 mL, 1.00 mmol) and cyclohexyl isocyanide (0.124 mL, 1.00 mmol); mp = 181 °C. ^1^H-NMR (CDCl_3_) δ: 7.93 (t, *J* = 7.3 Hz, 1H), 7.67–7.59 (m, 2H), 7.51 (d, *J* = 6.9 Hz, 2H), 7.38–7.28 (m, 3H), 6.82 (br, 1H), 6.31 (s, 1H), 3.87–3.79 (m, 1H), 1.96–1.88 (m, 2H), 1.70 (td, *J* = 8.9, 4.3 Hz, 2H), 1.63–1.55 (m, 1H), 1.41–1.30 (m, 12H), 1.29–1.17 (m, 4H). ^13^C-NMR (CDCl_3_) δ: 167.1, 162.5, 161.2(d), 135.7, 131.8, 130.3, 130.3, 128.8, 128.7, 128.6, 127.3, 122.7, 122.6, 119.7, 119.6, 84.5, 76.0, 47.9, 32.6, 25.3, 24.7, 24.4. ^11^B-NMR (CDCl_3_) δ: 30.44. HRMS (ESI, positive ion): *m/z* [M+H]^+^, found 482.2515. C_27_H_33_BFNO_5_ requires 482.2520.


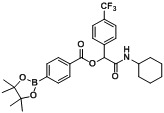

*2-(Cyclohexylamino)-2-oxo-1-(4-(trifluoromethyl)phenyl)ethyl4-(4,4,5,5-tetramethyl-1,3,2-dioxaborolan-2-yl)benzoate* (**A4**). The desired compound (405 mg, 76% yield) was prepared by General Procedure A using 4-carboxyphenylboronic acid ester (248 mg, 1.00 mmol), 4-(trifluoromethyl)benzaldehyde (0.13 mL, 1.00 mmol), and cyclohexyl isocyanide (0.124 mL, 1.00 mmol). mp = 249 °C. ^1^H-NMR (CDCl_3_) δ: 8.06 (d, *J* = 8.3 Hz, 2H), 7.93 (d, *J* = 8.3 Hz, 2H), 7.69–7.62 (m, 4H), 6.35 (s, 1H), 6.17 (br, 1H), 3.86–3.78 (m, 1H), 1.95–1.86 (m, 2H), 1.72–1.63 (m, 2H), 1.60 (td, *J* = 12.9, 3.8 Hz, 1H), 1.41–1.31 (m, 14H), 1.23–1.11 (m, 3H). ^13^C-NMR (CDCl_3_) δ: 166.6, 164.7, 139.5, 135.0, 130.9, 128.7, 127.6, 125.7, 125.7, 123.9(d), 84.3, 75.1, 48.3, 32.8, 25.4, 24.8, 24.6. ^11^B-NMR (CDCl_3_) δ: 31.2. HRMS (ESI, positive ion): *m/z* [M+H]^+^, found 532.2483. C_28_H_33_BF_3_NO_5_ requires 532.2488.


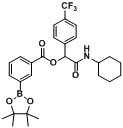

*2-(Cyclohexylamino)-2-oxo-1-(4-(trifluoromethyl)phenyl)ethyl3-(4,4,5,5-tetramethyl-1,3,2-dioxaborolan-2-yl)benzoate* (A5). The desired compound (416 mg, 79% yield) was prepared by General Procedure A using 3-carboxyphenylboronic acid ester (248 mg, 1.00 mmol), 4-(trifluoromethyl)benzaldehyde (0.136 mL, 1.00 mmol), and cyclohexyl isocyanide (0.124 mL, 1.00 mmol); mp = 150 °C. ^1^H-NMR (CDCl_3_) δ: 8.51 (s, 1H), 8.16 (td, *J* = 7.8, 1.5 Hz, 1H), 8.08–8.03 (m, 1H), 7.68 (d, *J* = 8.1 Hz, 2H), 7.63 (d, *J* = 8.3 Hz, 2H), 7.50 (t, *J* = 7.5 Hz, 1H), 6.38 (br, 1H), 6.33 (s, 1H), 3.87–3.78 (m, 1H), 1.92 (dt, *J* = 12.4, 3.1 Hz, 2H), 1.73–1.65 (m, 2H), 1.63–1.56 (m, 1H), 1.41–1.32 (m, 13H), 1.26–1.14 (m, 3H). ^13^C-NMR (CDCl_3_) δ: 166.7, 164.7, 140.0, 139.7, 135.9, 132.3, 130.8, 128.3, 128.1, 127.6, 125.6, 125.5, 123.9(d), 84.2, 75.2, 48.2, 32.7, 25.4, 24.8, 24.5. ^11^B-NMR (CDCl_3_) δ: 30.6. HRMS (ESI, positive ion): *m/z* [M+H]^+^, found 532.2487. C_28_H_33_BF_3_NO_5_ requires 532.2488.


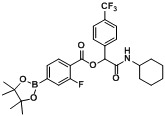

*2-(Cyclohexylamino)-2-oxo-1-(4-(trifluoromethyl)phenyl)ethyl2-fluoro-4-(4,4,5,5-tetramethyl-1,3,2-dioxaborolan-2-yl)benzoate* (**A6**). The desired compound (279 mg, 51% yield) was prepared by General Procedure A using 4-carboxy-3-fluorophenyl boronic acid ester (266 mg, 1.00 mmol), 4-(trifluoromethyl)benzaldehyde (0.136 mL, 1.00 mmol), and cyclohexyl isocyanide (0.124 mL, 1.00 mmol); mp = 220 °C. ^1^H-NMR (CDCl_3_) δ: 7.93 (t, *J* = 7.3 Hz, 1H), 7.68–7.60 (m, 6H), 6.83 (br, 1H), 6.34 (s, 1H), 3.85–3.78 (m, 1H), 1.96–1.88 (m, 2H), 1.71 (td, *J* = 13.4, 4.0 Hz, 2H), 1.60 (td, *J* = 12.6, 3.7 Hz, 1H), 1.43–1.31 (m, 14H), 1.28–1.20 (m, 3H). ^13^C-NMR (CDCl_3_) δ: 166.4, 162.5, 161.3, 139.7, 132.0, 130.9, 130.5, 127.6, 125.6, 125.6, 124.8, 122.8, 119.3, 119.2, 84.7, 75.6, 48.1, 32.7, 32.6, 25.4, 24.8, 24.4. ^11^B-NMR (CDCl_3_) δ: 30.6. HRMS (ESI, positive ion): *m/z* [M+H]^+^, found 550.2376. C_28_H_32_BF_4_NO_5_ requires 550.2394.


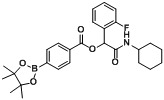

*2-(Cyclohexylamino)-1-(2-fluorophenyl)-2-oxoethyl4-(4,4,5,5-tetramethyl-1,3,2-dioxaborolan-2-yl)benzoate* (**A7**). The desired compound (418 mg, 87% yield) was prepared by General Procedure A using 4-carboxyphenylboronic acid ester (248 mg, 1.00 mmol), 2-fluorobenzaldehyde (0.105 mL, 1.00 mmol), and cyclohexyl isocyanide (0.124 mL, 1.00 mmol); mp = 167 °C. ^1^H-NMR (CDCl_3_) δ: 8.05 (d, *J* = 8.30 Hz, 2H), 7.89 (d, *J* = 8.1 Hz, 2H), 7.58 (dt, *J* = 7.4, 1.5 Hz, 1H), 7.33 (ddt, *J* = 7.7, 5.5, 1.7 Hz, 1H), 7.19–7.14 (m, 1H), 7.08 (t, *J* = 9.1 Hz, 1H), 6.48 (s, 1H), 6.17 (br, 1H), 3.87–3.79 (m, 1H), 2.00–1.93 (m, 1H), 1.88–1.81 (m, 1H), 1.73–1.61 (m, 2H), 1.61–1.55 (m, 1H), 1.40–1.29 (m, 13H), 1.27–1.10 (m, 3H) ^13^C-NMR (CDCl_3_) δ: 166.5, 164.9, 160.7(d), 134.8, 131.2, 130.7, 130.0, 128.7, 124.4, 124.4, 123.2, 115.7, 84.2, 70.8, 48.2, 32.6, 25.4, 24.8, 24.5. ^11^B-NMR (CDCl_3_) δ: 31.1. HRMS (ESI, positive ion): *m/z* [M+H]^+^, found 482.2512. C_27_H_33_BFNO_5_ requires 482.2520.


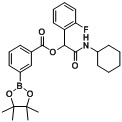

*2-(Cyclohexylamino)-1-(2-fluorophenyl)-2-oxoethyl3-(4,4,5,5-tetramethyl-1,3,2-dioxaborolan-2-yl)benzoate* (**A8**). The desired compound (415 mg, 86% yield) was prepared by General Procedure A using 3-carboxyphenylboronic acid ester (248 mg, 1.00 mmol), 2-fluorobenzaldehyde (0.105 mL, 1.00 mmol), and cyclohexyl isocyanide (0.124 mL, 1.00 mmol); mp = 173 °C. ^1^H-NMR (CDCl_3_) δ: 8.50 (s, 1H), 8.13 (d, *J* = 7.7 Hz, 1H), 8.01 (d, *J* = 7.4 Hz, 1H), 7.60–7.55 (m, 1H), 7.45 (t, *J* = 7.5 Hz, 1H), 7.34–7.28 (m, 1H), 7.15 (t, *J* = 7.6 Hz, 1H), 7.06 (t, *J* = 9.2 Hz, 1H), 6.32 (br, 1H), 3.87–3.80 (m, 1H), 1.96 (d, *J* = 11.6 Hz, 1H), 1.85 (d, *J* = 12.0 Hz, 1H), 1.72–1.61 (m, 2H), 1.59–1.53 (m, 1H), 1.40–1.29 (m, 14H), 1.29–1.12 (m, 3H). ^13^C-NMR (CDCl_3_) δ: 166.5, 164.8, 160.6, 139.7, 135.9, 132.2, 130.6, 129.9, 128.5, 127.9, 124.3, 123.2, 115.5, 84.0, 70.7, 48.1, 32.5, 25.3, 24.7, 24.4. ^11^B-NMR (CDCl_3_) δ: 30.6. HRMS (ESI, positive ion): *m/z* [M+H]^+^, found 482.2514. C_27_H_33_BFNO_5_ requires 482.2520.


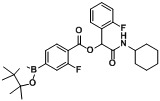

*2-(Cyclohexylamino)-1-(2-fluorophenyl)-2-oxoethyl2-fluoro-4-(4,4,5,5-tetramethyl-1,3,2-dioxaborolan-2-yl)benzoate* (**A9**). The desired compound (340 mg, 68% yield) was prepared by General Procedure A using 4-carboxy-3-fluorophenylboronic acid ester (266 mg, 1.00 mmol), 2-fluorobenzaldehyde (0.10 mL, 1.00 mmol), and cyclohexyl isocyanide (0.124 mL, 1.00 mmol); mp = 69 °C. ^1^H-NMR (CDCl_3_) δ: 7.89 (t, *J* = 7.3 Hz, 1H), 7.63–7.55 (m, 2H), 7.50 (dt, *J* = 7.4, 1.6 Hz, 1H), 7.31–7.26 (m, 1H), 7.11 (dt, *J* = 7.5, 0.8 Hz, 1H), 7.06–7.01 (m, 1H), 6.78 (br, 1H), 6.45 (s, 1H), 3.87–3.79 (m, 1H), 1.94 (dd, *J* = 12.0, 3.0 Hz, 1H), 1.91–1.85 (m, 1H), 1.68 (tdd, *J* = 17.1, 13.1, 4.0 Hz, 3H), 1.60–1.52 (m, 1H), 1.38–1.19 (m, 18H). ^13^C-NMR (CDCl_3_) δ: 166.3, 162.5, 161.8, 160.1, 131.7, 130.7, 130.0, 124.2, 123.2, 122.5, 119.4, 115.6, 84.4, 71.2, 47.9, 32.4, 25.3, 24.7, 24.3. ^11^B-NMR (CDCl_3_) δ: 30.8. HRMS (ESI, positive ion): *m/z* [M+H]^+^, found 500.2425. C_27_H_32_BF_2_NO_5_ requires 500.2425.


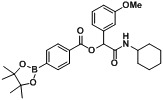

*2-(Cyclohexylamino)-1-(3-methoxyphenyl)-2-oxoethyl4-(4,4,5,5-tetramethyl-1,3,2-dioxaborolan-2-yl)benzoate* (**A10**). The desired compound (347 mg, 70% yield) was prepared by General Procedure A using 4-carboxyphenylboronic acid ester (248 mg, 1.00 mmol), 3-methoxybenzaldehyde (0.12 mL, 1.00 mmol), and cyclohexyl isocyanide (0.124 mL, 1.00 mmol); mp = 176 °C. ^1^H-NMR (CDCl_3_) δ: 8.06 (d, *J* = 8.1 Hz, 2H), 7.90 (d, *J* = 8.1 Hz, 2H), 7.29 (t, *J* = 8.0 Hz, 1H), 7.11 (d, *J* = 7.7 Hz, 1H), 7.08 (s, 1H), 6.89 (dd, *J* = 8.2, 2.3Hz, 1H), 6.27 (s, 1H), 6.08 (br, 1H), 3.84–3.77 (m, 4H), 1.92 (d, *J* = 9.5 Hz, 1H), 1.89–1.84 (m, 1H), 1.66 (dt, *J* = 14.4, 4.0 Hz, 2H), 1.61–1.55 (m, 1H), 1.40–1.30 (m, 14H), 1.21–1.08 (m, 3H). ^13^C-NMR (CDCl_3_) δ: 167.1, 164.9, 159.7, 137.1, 134.8, 131.4, 129.7, 128.7, 119.5, 114.4, 113.0, 84.2, 75.8, 55.2, 48.1, 32.7, 25.4, 24.8, 24.6. ^11^B-NMR (CDCl_3_) δ: 30.8. HRMS (ESI, positive ion): *m/z* [M+H]^+^, found 494.2717. C_28_H_36_BNO_6_ requires 494.2720.


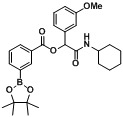

*2-(Cyclohexylamino)-1-(3-methoxyphenyl)-2-oxoethyl3-(4,4,5,5-tetramethyl-1,3,2-dioxaborolan-2-yl)benzoate* (**A11**). The desired compound (377 mg, 77% yield) was prepared by General Procedure A using 3-carboxyphenylboronic acid ester (248 mg, 1.00 mmol), 3-methoxybenzaldehyde (0.121 mL, 1.00 mmol), and cyclohexyl isocyanide (0.124 mL, 1.00 mmol); mp = 167 °C. ^1^H-NMR (CDCl_3_) δ: 8.51 (s, 1H), 8.16 (td, *J* = 7.8, 1.5 Hz, 1H), 8.03 (d, *J* = 7.2 Hz, 1H), 7.48 (t, *J* = 7.5 Hz, 1H), 7.29 (t, *J* = 8.0 Hz, 1H), 7.13–7.08 (m, 2H), 6.89 (dd, *J* = 8.2, 2.5 Hz, 1H), 6.27 (s, 1H), 6.15 (br, 1H), 3.87–3.80 (m, 4H), 1.97–1.87 (m, 2H), 1.74–1.64 (m, 3H), 1.59 (td, *J* = 12.8, 3.6 Hz, 1H), 1.41–1.33 (m, 14H), 1.27–1.14 (m, 3H). ^13^C-NMR (CDCl_3_) δ: 167.3, 164.8, 159.7, 139.8, 137.2, 136.0, 132.4, 129.7, 128.7, 128.0, 119.6, 114.5, 112.9, 84.2, 75.8, 55.3, 48.1, 32.7, 25.5, 24.8, 24.6. ^11^B-NMR (CDCl_3_) δ: 30.5. HRMS (ESI, positive ion): *m/z* [M+H]^+^, found 494.2704. C_28_H_36_BNO_6_ requires 494.2720.


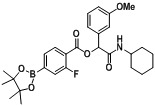

*2-(Cyclohexylamino)-1-(3-methoxyphenyl)-2-oxoethyl2-fluoro-4-(4,4,5,5-tetramethyl-1,3,2-dioxaborolan-2-yl)benzoate* (**A12**). The desired compound (320 mg, 63% yield) was prepared by General Procedure A using 4-carboxy-3-fluorophenylboronic acid ester (266 mg, 1.00 mmol), 3-methoxybenzaldehyde (0.12 mL, 1.00 mmol), and cyclohexyl isocyanide (0.124 mL, 1.00 mmol); mp = 163 °C. ^1^H-NMR (CDCl_3_) δ: 7.95 (t, *J* = 7.2 Hz, 1H), 7.68–7.59 (m, 2H), 7.30–7.26 (m, 1H), 7.13–7.06 (m, 2H), 6.89–6.85 (m, 1H), 6.75 (br, 1H), 6.29 (s, 1H), 3.86–3.81 (m, 1H), 3.80 (s, 3H), 1.93 (br, s, 2H), 1.71 (td, *J* = 8.6, 4.2 Hz, 2H), 1.63–1.57 (m, 1H), 1.43–1.38 (m, 2H), 1.36 (s, 13H), 1.28–1.19 (m, 3H). ^13^C-NMR (CDCl_3_) δ: 166.9, 162.5, 161.2, 159.6, 137.1, 131.9, 130.3, 129.6, 122.6, 119.6, 119.4, 114.4, 112.9, 84.5, 76.2, 55.1, 47.9, 32.6, 25.4, 24.7, 24.4. ^11^B-NMR (CDCl_3_) δ: 30.2. HRMS (ESI, positive ion): *m/z* [M+H]^+^, found 512.2615. C_28_H_35_BFNO_6_ requires 512.2625.


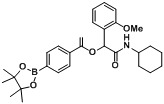

*2-(Cyclohexylamino)-1-(2-methoxyphenyl)-2-oxoethyl4-(4,4,5,5-tetramethyl-1,3,2-dioxaborolan-2-yl)benzoate* (**A13**). The desired compound (428 mg, 87% yield) was prepared by General Procedure A using 4-carboxyphenylboronic acid ester (248 mg, 1.00 mmol), 2-methoxybenzaldehyde (0.121 mL, 1.00 mmol), and cyclohexyl isocyanide (0.124 mL, 1.00 mmol); mp = 182 °C. ^1^H-NMR (CDCl_3_) δ: 8.08 (d, *J* = 8.1 Hz, 2H), 7.88 (d, *J* = 8.1 Hz, 2H), 7.58 (dd, *J* = 7.5, 1.4 Hz, 1H), 7.34–7.29 (m, 1H), 7.00 (t, *J* = 7.5 Hz, 1H), 6.92 (d, *J* = 8.3 Hz, 1H), 6.57 (s, 1H), 6.17 (br, 1H), 3.89–3.84 (m, 3H), 3.82–3.74 (m, 1H), 1.98–1.92 (m, 1H), 1.79–1.72 (m, 1H), 1.69–1.63 (m, 1H), 1.60–1.51 (m, 2H), 1.38–1.26 (m, 14H), 1.25–1.12 (m, 2H), 1.10–1.03 (m, 1H). ^13^C-NMR (CDCl_3_) δ: 167.3, 165.3, 156.5, 134.6, 131.8, 129.9, 128.7, 128.5, 124.2, 121.0, 110.9, 84.1, 77.2, 76.8, 70.8, 55.5, 47.8, 32.5, 25.4, 24.7, 24.3. ^11^B-NMR (CDCl_3_) δ: 31.0. HRMS (ESI, positive ion): *m/z* [M+H]^+^, found 494.2713. C_28_H_36_BNO_6_ requires 494.2720.


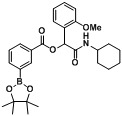

*2-(Cyclohexylamino)-1-(2-methoxyphenyl)-2-oxoethyl3-(4,4,5,5-tetramethyl-1,3,2-dioxaborolan-2-yl)benzoate* (**A14**). The desired compound (392 mg, 79% yield) was prepared by General Procedure A using 3-carboxyphenylboronic acid ester (248 mg, 1.00 mmol), 2-methoxybenzaldehyde (0.121 mL, 1.00 mmol), and cyclohexyl isocyanide (0.124 mL, 1.00 mmol); mp = 164 °C. ^1^H-NMR (CDCl_3_) δ: 8.54 (s, 1H), 8.18 (d, *J* = 7.8 Hz, 1H), 7.99 (d, *J* = 7.2 Hz, 1H), 7.58 (d, *J* = 7.4 Hz, 1H), 7.44 (t, *J* = 7.5 Hz, 1H), 7.30 (t, *J* = 7.7 Hz, 1H), 6.99 (t, *J* = 7.5 Hz, 1H), 6.91 (d, *J* = 8.1 Hz, 1H), 6.57 (s, 1H), 6.24 (br, 1H), 3.87 (s, 3H), 3.82–3.76 (m, 1H), 1.95 (d, *J* = 9.4 Hz, 1H), 1.77 (d, *J* = 11.6 Hz, 1H), 1.70–1.63 (m, 1H), 1.61–1.51 (m, 2H), 1.38–1.27 (m, 13H), 1.26–1.06 (m, 4H). ^13^C-NMR (CDCl_3_) δ: 167.4, 165.3, 156.6, 139.3, 136.0, 132.3, 129.9, 129.0, 128.6, 127.7, 124.2, 120.9, 110.9, 83.9, 70.9, 55.5, 47.7, 32.5, 25.3, 24.7, 24.3. ^11^B-NMR (CDCl_3_) δ: 30.6. HRMS (ESI, positive ion): *m/z* [M+H]^+^, found 494.2715. C_28_H_36_BNO_6_ requires 494.2720.


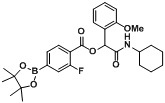

*2-(Cyclohexylamino)-1-(2-methoxyphenyl)-2-oxoethyl2-fluoro-4-(4,4,5,5-tetramethyl-1,3,2-dioxaborolan-2-yl)benzoate* (**A15**). The desired compound (324 mg, 63% yield) was prepared by General Procedure A using 4-carboxy-3-fluorophenylboronic acid ester (266 mg, 1.00 mmol), 2-methoxybenzaldehyde (0.121 mL, 1.00 mmol), and cyclohexyl isocyanide (0.124 mL, 1.00 mmol); mp = 75 °C. ^1^H-NMR (CDCl_3_) δ: 7.93 (t, *J* = 7.2 Hz, 1H), 7.60 (d, *J* = 7.6 Hz, 1H), 7.56 (d, *J* = 11.1 Hz, 1H), 7.51 (dd, *J* = 7.6, 1.5 Hz, 1H), 7.30–7.26 (m, 2H), 6.95 (t, *J* = 7.4 Hz, 1H), 6.88 (d, *J* = 8.3 Hz, 1H), 6.60 (br, 1H), 6.56 (s, 1H), 3.86–3.76 (m, 4H), 1.95 (dd, *J* = 12.0, 3.0 Hz, 1H), 1.84–1.78 (m, 1H), 1.72–1.65 (m, 1H), 1.65–1.58 (m, 1H), 1.58–1.52 (m, 1H), 1.39–1.28 (m, 14H), 1.27–1.11 (m, 3H). ^13^C-NMR (CDCl_3_) δ: 167.2, 162.8, 162.0, 160.3, 156.8, 131.6, 130.0, 129.1, 124.1, 122.5, 122.4, 120.8, 120.1, 111.0, 84.3, 71.7, 55.5, 47.6, 32.4, 25.3, 24.6, 24.3. ^11^B-NMR (CDCl_3_) δ: 30.1. HRMS (ESI, positive ion): *m/z* [M+H]^+^, found 512.2608. C_28_H_35_BFNO_6_ requires 512.2625.


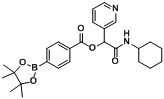

*2-(Cyclohexylamino)-2-oxo-1-(pyridin-3-yl)ethyl4-(4,4,5,5-tetramethyl-1,3,2-dioxaborolan-2-yl)benzoate* (**A16**). The desired compound (260 mg, 56% yield) was prepared by General Procedure A using 4-carboxyphenylboronic acid ester (248 mg, 1.00 mmol), pyridine-3-aldehyde (0.09 mL, 1.00 mmol), and cyclohexyl isocyanide (0.124 mL, 1.00 mmol); mp = 230 °C. ^1^H-NMR (CDCl_3_) δ: 8.77 (s, 1H), 8.60 (d, *J* = 4.5 Hz, 1H), 8.04 (d, *J* = 8.3 Hz, 2H), 7.93-7.86 (m, 3H), 7.32 (dd, *J* = 7.9, 4.8 Hz, 1H), 6.32 (s, 1H), 6.25 (br, 1H), 3.84–3.78 (m, 1H), 1.95–1.85 (m, 2H), 1.71–1.62 (m, 2H), 1.61–1.56 (m, 1H), 1.39–1.30 (m, 13H), 1.25–1.10 (m, 4H). ^13^C-NMR (CDCl_3_) δ: 166.5, 164.8, 150.1, 148.5, 135.3, 135.0, 131.7, 130.9, 128.7, 123.5, 84.3, 73.8, 48.3, 32.7, 25.3, 24.8, 24.6. ^11^B-NMR (CDCl_3_) δ: 30.6. HRMS (ESI, positive ion): *m/z* [M+H]^+^, found 465.2553. C_26_H_33_BN_2_O_5_ requires 465.2566.


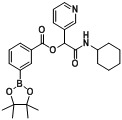

*2-(Cyclohexylamino)-2-oxo-1-(pyridin-3-yl)ethyl3-(4,4,5,5-tetramethyl-1,3,2-dioxaborolan-2-yl)benzoate* (**A17**). The desired compound (240 mg, 52% yield) was prepared by General Procedure A using 3-carboxyphenylboronic acid ester (248 mg, 1.00 mmol), pyridine-3-aldehyde (0.094 mL, 1.00 mmol), and cyclohexyl isocyanide (0.124 mL, 1.00 mmol); mp = 127 °C. ^1^H-NMR (CDCl_3_) δ: 8.76 (s, 1H), 8.56 (s, 1H), 8.46 (s, 1H), 8.10 (d, *J* = 7.8 Hz, 1H), 8.00 (d, *J* = 7.4 Hz, 1H), 7.88 (d, *J* = 8.1 Hz, 1H), 7.43 (t, *J* = 7.5 Hz, 1H), 7.32–7.27 (m, 1H), 6.60 (br, 1H), 6.28 (s, 1H), 3.82–3.74 (m, 1H), 1.93–1.81 (m, 2H), 1.67–1.59 (m, 2H), 1.57–1.50 (m, 1H), 1.35–1.27 (m, 13H), 1.22–1.09 (m, 4H). ^13^C-NMR (CDCl_3_) δ: 166.5, 164.7, 149.8, 148.5, 140.0, 135.9, 135.3, 132.3, 128.1, 123.5, 84.1, 73.7, 48.2, 32.6, 25.3, 24.7, 24.5. ^11^B-NMR (CDCl_3_) δ: 30.9. HRMS (ESI, positive ion): *m/z* [M+H]^+^, found 465.2548. C_26_H_33_BN_2_O_5_ requires 465.2566.


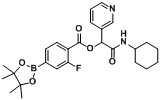

*2-(Cyclohexylamino)-2-oxo-1-(pyridin-3-yl)ethyl2-fluoro-4-(4,4,5,5-tetramethyl-1,3,2-dioxaborolan-2-yl)benzoate* (**A18**). The desired compound (270 mg, 52% yield) was prepared by General Procedure A using 4-carboxy-3-fluorophenylboronic acid ester (266 mg, 1.00 mmol), pyridine-3-aldehyde (0.094 mL, 1.00 mmol), and cyclohexyl isocyanide (0.124 mL, 1.00 mmol); mp = 203 °C. ^1^H-NMR (CDCl_3_) δ: 8.75 (s, 1H), 8.57 (dd, *J* = 4.7, 1.1 Hz, 1H), 7.91 (t, *J* = 7.2 Hz, 1H), 7.83 (td, *J* = 7.9, 1.7 Hz, 1H), 7.66–7.58 (m, 2H), 6.83 (br, 1H), 6.32 (s, 1H), 3.86–3.78 (m, 1H), 1.95–1.89 (m, 2H), 1.70 (td, *J* = 8.9, 4.1 Hz, 2H), 1.62–1.55 (m, 1H), 1.41–1.31 (m, 14H), 1.26–1.19 (m, 3H). ^13^C-NMR (CDCl_3_) δ: 166.2, 162.6, 161.3, 150.0, 148.6, 135.2, 131.9, 131.8, 130.5, 123.4, 122.7, 119.2, 84.6, 74.1, 48.1, 32.6, 25.4, 24.8, 24.4. ^11^B-NMR (CDCl_3_) δ: 30.0. HRMS (ESI, positive ion): *m/z* [M+H]^+^, found 483.2455. C_26_H_32_BFN_2_O_5_ requires 483.3012.


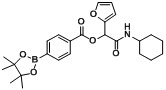

*2-(Cyclohexylamino)-1-(furan-2-yl)-2-oxoethyl4-(4,4,5,5-tetramethyl-1,3,2-dioxaborolan-2-yl)benzoate* (**A19**). The desired compound (260 mg, 57% yield) was prepared by General Procedure A using 4-carboxyphenyl boronic acid ester (248 mg, 1.00 mmol), furan-2-carbaldehyde (0.08 mL, 1.00 mmol), and cyclohexyl isocyanide (0.124 mL, 1.00 mmol); mp = 182.5 °C. ^1^H-NMR (CDCl_3_) δ: 8.02 (d, *J* = 8.3 Hz, 2H), 7.87 (d, *J* = 8.3 Hz, 2H), 7.41 (s, 1H), 6.55 (d, *J* = 3.2 Hz, 1H), 6.39 (s, 1H), 6.36 (dd, *J* = 3.1, 1.8 Hz, 1H), 6.20 (br, 1H), 3.87–3.80 (m, 1H), 1.99–1.93 (m, 1H), 1.90–1.83 (m, 1H), 1.71–1.61 (m, 2H), 1.57 (td, *J* = 12.9, 3.7 Hz, 1H), 1.38–1.29 (m, 14H), 1.25–1.09 (m, 3H). ^13^C-NMR (CDCl_3_) δ: 164.9, 164.8, 148.1, 143.5, 134.7, 131.1, 128.7, 111.2, 110.6, 84.2, 69.2, 48.2, 32.6, 25.3, 24.7, 24.5. ^11^B-NMR (CDCl_3_) δ: 30.6. HRMS (ESI, positive ion): *m/z* [M+H]^+^, found 454.2399. C_25_H_32_BNO_6_ requires 454.2406.


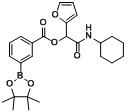

*2-(Cyclohexylamino)-1-(furan-2-yl)-2-oxoethyl3-(4,4,5,5-tetramethyl-1,3,2-dioxaborolan-2-yl)benzoate* (**A20**). The desired compound (280 mg, 63% yield) was prepared by General Procedure A using 3-carboxyphenylboronic acid ester (248 mg, 1.00 mmol), furan-2-carbaldehyde (0.082 mL, 1.00 mmol), and cyclohexyl isocyanide (0.124 mL, 1.00 mmol); mp = 161.5 °C. ^1^H-NMR (CDCl_3_) δ: 8.46 (s, 1H), 8.12 (d, *J* = 7.8 Hz, 1H), 8.00 (d, *J* = 7.3 Hz, 1H), 7.44 (t, *J* = 7.4 Hz, 1H), 7.40 (s, 1H), 6.55 (d, *J* = 3.3 Hz, 1H), 6.38 (s, 1H), 6.35 (dd, *J* = 3.0, 1.9 Hz, 1H), 6.30 (br, 1H), 3.89–3.81 (m, 1H), 1.97 (d, *J* = 9.2 Hz, 1H), 1.91–1.85 (m, 1H), 1.73–1.62 (m, 2H), 1.54–1.60 (m, 1H), 1.29–1.40 (m, 14H), 1.12–1.28 (m, 3H). ^13^C-NMR (CDCl_3_) δ: 165.0, 164.7, 148.2, 143.4, 139.7, 135.9, 132.4, 128.4, 127.9, 111.2, 110.5, 84.0, 69.2, 48.2, 32.5, 25.3, 24.7, 24.4. ^11^B-NMR (CDCl_3_) δ: 30.7. HRMS (ESI, positive ion): *m/z* [M+H]^+^, found 454.2394. C_25_H_32_BNO_6_ requires 454.2406.


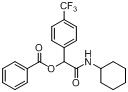

*2-(Cyclohexylamino)-2-oxo-1-(4-(trifluoromethyl)phenyl)ethyl benzoate* (**A21**). The desired compound (360 mg, 89% yield) was prepared by General Procedure A using benzoic acid (122.12 mg, 1.00 mmol), 4-(trifluoromethyl)benzaldehyde (0.13 mL, 1.00 mmol), and cyclohexyl isocyanide (0.124 mL, 1.00 mmol); mp = 201 °C. ^1^H-NMR (CDCl_3_) δ: 8.10 (d, *J* = 8.4 Hz, 2H), 7.67–7.63 (m, 5H), 7.52 (t, *J* = 7.5 Hz, 2H), 6.34 (s, 1H), 6.14 (br, 1H), 3.84–3.79 (m, 1H), 1.94–1.90 (m, 2H), 1.72–1.63 (m, 2H), 1.90–1.60 (m, 4H), 1.39–1.33 (m, 2H), 1.23–1.16 (m, 3H). ^13^C-NMR (CDCl_3_) δ: 166.6, 164.6, 139.6, 133.9, 131.1, 130.9, 129.7, 128.9, 128.7, 127.58, 125.7, 124.7, 122.9, 75.1, 48.3, 32.9, 32.8, 25.3, 24.6. 

### 3.3. General Procedure B for the Synthesis of Boron-Containing α-Acyloxyl Amide **B1–10**


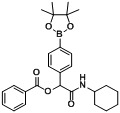

*2-(Cyclohexylamino)-2-oxo-1-(4-(4,4,5,5-tetramethyl-1,3,2-dioxaborolan-2-yl)phenyl)ethyl benzoate* (**B1**). A 10 mL glass tube containing the benzoic acid (122 mg, 1.00 mmol), *p*-formylphenylboronic acid ester (232 mg, 1.00 mmol), and D.I. H_2_O (1 mL) was first microwave irradiated for 6 min (45 °C, 150 W) under medium speed magnetic stirring. The cyclohexyl isocyanide (**3**, 0.124 mL, 1.00 mmol) was then added to the reaction mixture. The additional microwave irradiation was applied for 150 min (45 °C, 150 W) under medium speed magnetic stirring. After being diluted in dichloromethane, the resulted reaction mixture was washed twice with a saturated aqueous solution of NaHCO_3_ and with brine. The resulted organic layer was collected and dried over MgSO_4_ and concentrated *in vacuo*. The crude product was then dissolved in ethyl acetate (3.0 mL) prior the slow addition of *n*-hexane. The resulting precipitate was formed and collected by filtration affording the desired product in 75% yield, mp = 166 °C. ^1^H-NMR (CDCl_3_) δ: 8.08 (d, *J* = 7.7 Hz, 2H), 7.83 (d, *J* = 7.9 Hz, 2H), 7.61–7.56 (m, 1H), 7.54 (d, *J* = 7.9 Hz, 2H), 7.45 (t, *J* = 7.7 Hz, 2H), 6.29 (s, 1H), 6.11 (br, 1H), 3.83–3.75 (m, 1H), 1.93–1.82 (m, 2H), 1.69–1.60 (m, 2H), 1.59–1.53 (m, 1H), 1.36–1.27 (m, 15H), 1.19–1.04 (m, 3H). ^13^C-NMR (CDCl_3_) δ: 167.0, 164.9, 138.5, 135.1, 133.5, 129.7, 129.2, 128.5, 126.5, 83.8, 75.9, 48.2, 32.7, 25.3, 24.7, 24.6. ^11^B-NMR (CDCl_3_) δ: 31.5. HRMS (ESI, positive ion): *m/z* [M+H]^+^, found 464.2616. C_27_H_34_BNO_5_ requires 464.2614.


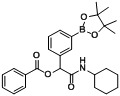

*2-(Cyclohexylamino)-2-oxo-1-(3-(4,4,5,5-tetramethyl-1,3,2-dioxaborolan-2-yl)phenyl)ethyl benzoate* (**B2**). The desired compound (320 mg, 69% yield) was prepared by General Procedure B using benzoic acid (122.12 mg, 1.00 mmol), *m*-formylphenylboronic acid ester (232 mg, 1.00 mmol), and cyclohexyl isocyanide (0.124 mL, 1.00 mmol); mp = 165 °C. ^1^H-NMR (CDCl_3_) δ: 8.08 (d, *J* = 7.4 Hz, 2H), 7.96 (s, 1H), 7.80 (d, *J* = 7.0 Hz, 1H), 7.65 (d, *J* = 7.8 Hz, 1H), 7.57 (t, *J* = 7.4 Hz, 1H), 7.45 (t, *J* = 7.6 Hz, 2H), 7.39 (t, *J* = 7.6 Hz, 1H), 6.29 (s, 1H), 6.11 (br, 1H), 3.85–3.78 (m, 1H), 1.93 (d, *J* = 9.6 Hz, 1H), 1.86 (d, *J* = 9.6 Hz, 1H), 1.70–1.60 (m, 2H), 1.60–1.54 (m, 1H), 1.40–1.25 (m, 14H), 1.22–1.06 (m, 3H). ^13^C-NMR (CDCl_3_) δ: 167.2, 164.9, 135.3, 135.0, 134.0, 133.4, 130.2, 129.7, 129.3, 128.4, 128.0, 83.8, 76.0, 48.1, 32.7, 25.3, 24.7, 24.6. ^11^B-NMR (CDCl_3_) δ: 30.6. HRMS (ESI, positive ion): *m/z* [M+H]^+^, found 464.2607. C_27_H_34_BNO_5_ requires 464.2614.


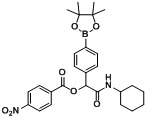

*2-(Cyclohexylamino)-2-oxo-1-(4-(4,4,5,5-tetramethyl-1,3,2-dioxaborolan-2-yl)phenyl)ethyl-4-nitro-benzoate* (**B3**). The desired compound (290 mg, 57% yield) was prepared by General Procedure B using 4-nitrobenzoic acid (167.19 mg, 1.00 mmol), *p*-formylphenylboronic acid ester (232 mg, 1.00 mmol), and cyclohexyl isocyanide (0.124 mL, 1.00 mmol); mp = 108 °C. ^1^H-NMR (CDCl_3_) δ: 8.30–8.20 (m, 4H), 7.84 (d, *J* = 8.1 Hz, 2H), 7.53 (d, *J* = 7.6 Hz, 2H), 6.23 (s, 1H), 5.93 (br, 1H), 3.80–3.73 (m, 1H), 1.92–1.86 (m, 1H), 1.80 (d, *J* = 11.4 Hz, 1H), 1.69–1.53 (m, 3H), 1.37–1.26 (m, 14H), 1.17–1.00 (m, 3H). ^13^C-NMR (CDCl_3_) δ: 166.4, 163.5, 150.7, 137.6, 135.3, 134.7, 130.9, 126.8, 123.6, 83.9, 76.7, 48.5, 32.6, 25.3, 24.7, 24.6. ^11^B-NMR (CDCl_3_) δ: 31.0. HRMS (ESI, positive ion): *m/z* [M+H]^+^, found 509.2448. C_27_H_33_BN_2_O_7_ requires 509.2465.


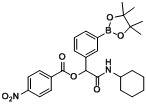

*2-(Cyclohexylamino)-2-oxo-1-(3-(4,4,5,5-tetramethyl-1,3,2-dioxaborolan-2-yl)phenyl)ethyl-4-nitro-benzoate* (**B4**). The desired compound (299 mg, 59% yield) was prepared by General Procedure B using 4-nitrobenzoic acid (167 mg, 1.00 mmol), *m*-formylphenylboronic acid ester (232 mg, 1.00 mmol), and cyclohexyl isocyanide (0.124 mL, 1.00 mmol); mp = 94 °C. ^1^H-NMR (CDCl_3_) δ: 8.21 (s, 4H), 7.94 (s, 1H), 7.79 (d, *J* = 7.1 Hz, 1H), 7.63 (d, *J* = 8.0 Hz, 1H), 7.38 (t, *J* = 7.6 Hz, 1H), 6.22 (s, 1H), 6.16 (br, 1H), 3.78–3.70 (m, 1H), 1.87 (d, *J* = 10.4 Hz, 1H), 1.80–1.75 (m, 1H), 1.66–1.55 (m, 2H), 1.55–1.50 (m, 1H), 1.34–1.23 (m, 14H), 1.17–0.99 (m, 3H). ^13^C-NMR (CDCl_3_) δ: 166.6, 163.4, 150.5, 135.6, 134.7, 134.1, 134.0, 130.8, 130.3, 128.2, 123.4, 83.8, 76.7, 48.4, 32.5, 25.2, 24.6, 24.5. ^11^B-NMR (CDCl_3_) δ: 30.8. HRMS (ESI, positive ion): *m/z* [M+H]^+^, found 509.2442. C_27_H_33_BN_2_O_7_ requires 509.2465.


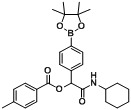

*2-(Cyclohexylamino)-2-oxo-1-(4-(4,4,5,5-tetramethyl-1,3,2-dioxaborolan-2-yl)phenyl)ethyl-4-methyl-benzoate* (**B5**). The desired compound (344 mg, 72% yield) was prepared *by General Procedure B* using 4-methylbenzoic acid (163.38 mg, 1.00 mmol), *p*-formylphenylboronic acid ester (232 mg, 1.00 mmol), and cyclohexyl isocyanide (0.124 mL, 1.00 mmol); mp = 90 °C. ^1^H-NMR (CDCl_3_) δ: 7.97 (d, J = 7.8 Hz, 2H), 7.83 (d, J = 7.9 Hz, 2H), 7.53 (d, J = 7.9 Hz, 2H), 7.26 (d, J = 7.8 Hz, 2H), 6.28 (s, 1H), 6.07 (br, 1H), 3.83–3.75 (m, 1H), 2.41 (s, 3H), 1.92–1.84 (m, 3H), 1.69–1.61 (m, 2H), 1.60–1.55 (m, 1H), 1.37–1.30 (m, 14H), 1.18–1.07 (m, 3H). ^13^C-NMR (CDCl_3_) δ: 167.2, 164.9, 144.4, 138.6, 135.1, 129.7, 129.3, 126.5, 83.8, 75.7, 48.1, 32.7, 25.3, 24.8, 24.6, 21.6. ^11^B-NMR (CDCl_3_) δ: 30.9. HRMS (ESI, positive ion): m/z [M+H]^+^, found 478.2752. C_28_H_36_BNO_5_ requires 478.2771.


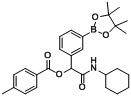

*2-(Cyclohexylamino)-2-oxo-1-(3-(4,4,5,5-tetramethyl-1,3,2-dioxaborolan-2-yl)phenyl)ethyl-4-methyl-benzoate* (**B6**). The desired compound (302 mg, 63% yield) was prepared by General Procedure B using 4-methylbenzoic acid (163.38 mg, 1.00 mmol), *m*-formylphenylboronic acid ester (232 mg, 1.00 mmol), and cyclohexyl isocyanide (0.124 mL, 1.00 mmol); mp = 81 °C. ^1^H-NMR (CDCl_3_) δ: 8.00–7.93 (m, 3H), 7.79 (d, *J* = 7.4 Hz, 1H), 7.64 (d, *J* = 7.8 Hz, 1H), 7.38 (t, *J* = 7.6 Hz, 1H), 7.24 (d, *J* = 7.8 Hz, 2H), 6.28 (s, 1H), 6.14 (br, 1H), 3.85–3.77 (m, 1H), 2.39 (s, 3H), 1.92 (d, *J* = 9.6 Hz, 1H), 1.86 (d, *J* = 11.4 Hz, 1H), 1.70–1.61 (m, 2H), 1.59–1.53 (m, 1H), 1.37–1.28 (m, 14H), 1.23–1.07 (m, 3H). ^13^C-NMR (CDCl_3_) δ: 167.4, 164.9, 144.2, 135.2, 135.1, 133.9, 130.2, 129.7, 129.1, 128.0, 126.5, 83.8, 75.8, 48.1, 32.6, 25.3, 24.7, 24.5, 21.6. ^11^B-NMR (CDCl_3_) δ: 30.7. HRMS (ESI, positive ion): *m/z* [M+H]^+^, found 478.2752. C_28_H_36_BNO_5_ requires 478.2771.


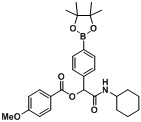

*2-(Cyclohexylamino)-2-oxo-1-(4-(4,4,5,5-tetramethyl-1,3,2-dioxaborolan-2-yl)phenyl)ethyl-4-methoxybenzoate* (**B7**). The desired compound (393 mg, 80% yield) was prepared by General Procedure B using 4-methoxybenzoic acid (182.58 mg, 1.00 mmol), *p*-formylphenylboronic acid ester (232 mg, 1.00 mmol), and cyclohexyl isocyanide (0.124 mL, 1.00 mmol); mp = 94 °C. ^1^H-NMR (CDCl_3_) δ: 8.02 (d, *J* = 8.7 Hz, 2H), 7.81 (d, *J* = 8.1 Hz, 2H), 7.53 (d, *J* = 8.1 Hz, 2H), 6.90 (d, *J* = 8.7 Hz, 2H), 6.28–6.23 (m, 2H), 3.82–3.72 (m, 4H), 1.89–1.78 (m, 2H), 1.66–1.57 (m, 2H), 1.56–1.50 (m, 1H), 1.33–1.24 (m, 14H), 1.14–1.02 (m, 3H). ^13^C-NMR (CDCl_3_) δ: 167.2, 164.5, 163.7, 138.7, 134.9, 131.7, 126.3, 121.4, 113.7, 83.6, 75.5, 55.2, 48.0, 32.5, 25.2, 24.6, 24.5. ^11^B-NMR (CDCl_3_) δ: 30.8. HRMS (ESI, positive ion): *m/z* [M+H]^+^, found 494.2717. C_28_H_36_BNO_6_ requires 494.2720.


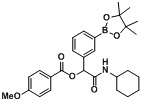
*2-(Cyclohexylamino)-2-oxo-1-(3-(4,4,5,5-tetramethyl-1,3,2-dioxaborolan-2-yl)phenyl)ethyl-4-methoxybenzoate* (**B8**). The desired compound (404 mg, 82% yield) was prepared by General Procedure B using 4-methoxybenzoic acid (182.58 mg, 1.00 mmol), *m*-formylphenylboronic acid ester (232 mg, 1.00 mmol), and cyclohexyl isocyanide (0.124 mL, 1.00 mmol); mp = 80 °C. ^1^H-NMR (CDCl_3_) δ: 8.01 (d, *J* = 8.56 Hz, 2H), 7.95 (s, 1H), 7.77 (d, *J* = 7.6 Hz, 1H), 7.62 (d, *J* = 7.6 Hz, 1H), 7.35 (t, *J* = 7.6 Hz, 1H), 6.87 (d, *J* = 9.0 Hz, 2H), 6.29 (br, 1H), 6.25 (s, 1H), 3.81–3.72 (m, 4H), 1.87 (d, *J* = 10.4 Hz, 1H), 1.80 (d, *J* = 10.4 Hz, 1H), 1.66–1.56 (m, 2H), 1.55–1.48 (m, 1H), 1.34–1.22 (m, 14H), 1.18–1.02 (m, 3H). ^13^C-NMR (CDCl_3_) δ: 167.4, 164.5, 163.5, 135.1, 135.0, 133.8, 131.6, 130.0, 127.8, 121.4, 113.6, 83.6, 75.5, 55.2, 48.0, 32.4, 25.1, 24.6, 24.4. ^11^B-NMR (CDCl_3_) δ: 31.6. HRMS (ESI, positive ion): *m/z* [M+H]^+^, found 494.2707. C_28_H_36_BNO_6_ requires 494.2720.


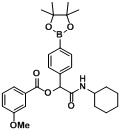
*2-(Cyclohexylamino)-2-oxo-1-(4-(4,4,5,5-tetramethyl-1,3,2-dioxaborolan-2-yl)phenyl)ethyl-3-methoxybenzoate* (**B9**). The desired compound (419 mg, 85% yield) was prepared by General Procedure B using 3-methoxybenzoic acid (182.58 mg, 1.00 mmol), *p*-formylphenylboronic acid ester (232 mg, 1.00 mmol), and cyclohexyl isocyanide (0.124 mL, 1.00 mmol); mp = 74 °C. ^1^H-NMR (CDCl_3_) δ: 7.81 (d, *J* = 7.4 Hz, 2H), 7.65 (d, *J* = 7.8 Hz, 1H), 7.58–7.50 (m, 3H), 7.32 (dt, *J* = 7.8, 3.5 Hz, 1H), 7.10–7.05 (m, 1H), 6.33 (br, 1H), 6.24 (s, 1H), 3.803.71 (m, 4H), 1.86 (d, *J* = 11.8 Hz, 1H), 1.83–1.77 (m, 1H), 1.61 (t, *J* = 13.8 Hz, 2H), 1.52 (d, *J* = 9.6 Hz, 1H), 1.33–1.23 (m, 14H), 1.16–1.01 (m, 3H). ^13^C-NMR (CDCl_3_) δ: 167.0, 164.7, 159.4, 138.4, 134.9, 130.4, 129.4, 126.3, 121.8, 119.5, 114.3, 83.6, 75.9, 55.2, 48.1, 32.5, 25.2, 24.6, 24.5. ^11^B-NMR (CDCl_3_) δ: 31.5. HRMS (ESI, positive ion): *m/z* [M+H]^+^, found 494.2702. C_28_H_36_BNO_6_ requires 494.2720.


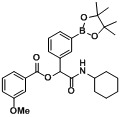
*2-(Cyclohexylamino)-2-oxo-1-(3-(4,4,5,5-tetramethyl-1,3,2-dioxaborolan-2-yl)phenyl)ethyl-3-methoxybenzoate* (**B10**). The desired compound (437 mg, 89% yield) was prepared by General Procedure B using 3-methoxybenzoic acid (182.58 mg, 1.00 mmol), *m*-formylphenylboronic acid ester (232 mg, 1.00 mmol), and cyclohexyl isocyanide (0.124 mL, 1.00 mmol); mp = 67 °C. ^1^H-NMR (CDCl_3_) δ: 7.96 (s, 1H), 7.78 (d, *J* = 7.4 Hz, 1H), 7.63 (d, *J* = 7.4 Hz, 1H), 7.66 (d, *J* = 7.8 Hz, 1H), 7.57 (s, 1H), 7.36 (t, *J* = 7.4 Hz, 1H), 7.32 (t, *J* = 7.8 Hz, 1H), 7.08 (dd, *J* = 8.3, 2.6 Hz, 1H), 6.29–6.22 (m, 2H), 3.82–3.76 (m, 4H), 1.89 (d, *J* = 9.6 Hz, 1H), 1.82 (d, *J* = 11.8 Hz, 1H), 1.67–1.58 (m, 2H), 1.56–1.51 (m, 1H), 1.34–1.26 (m, 14H), 1.19–1.13 (m, 1H), 1.13–1.05 (m, 2H). ^13^C-NMR (CDCl_3_) δ: 167.2, 164.8, 159.4, 135.2, 134.9, 133.8, 130.4, 130.0, 129.4, 127.9, 121.9, 119.6, 114.3, 83.7, 75.9, 55.2, 48.1, 32.5, 25.2, 24.6, 24.5. ^11^B-NMR (CDCl_3_) δ: 31.5. HRMS (ESI, positive ion): *m/z* [M+H]^+^, found 494.2713. C_28_H_36_BNO_6_ requires 494.2720.


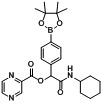
*2-(Cyclohexylamino)-2-oxo-1-(4-(4,4,5,5-tetramethyl-1,3,2-dioxaborolan-2-yl)phenyl)ethyl pyrazine-2-carboxylate* (**B11**). The desired compound (237 mg, 51% yield) was prepared by General Procedure B using pyrazine-2-carboxylic acid (124 mg, 1.00 mmol), *p*-formylphenylboronic acid ester (232 mg, 1.00 mmol), and cyclohexyl isocyanide (0.124 mL, 1.00 mmol); mp = 184 °C. ^1^H-NMR (CDCl_3_) δ 9.30 (s, 1H), 8.78 (s, 1H), 8.72 (s, 1H), 7.82(d, *J*= 7.8 Hz, 2H), 7.53 (d, *J* = 7.8 Hz, 2H), 6.46 (br.), 6.32 (s, 1H), 3.80–3.79 (m, 1H), 1.91–1.84 (m, 2H), 1.69–1.64 (m, 2H), 1.58–1.56 (m, 1H), 1.34–1.32 (m, 14H), 1.25–1.20 (m, 3H). ^13^C-NMR (CDCl_3_) δ 166.5, 162.4, 148.0, 146.4, 144.4, 142.8, 137.8, 135.2, 126.7, 83.87, 83.8, 48.2, 32.7, 32.6, 25.3, 24.7, 24.5. ^11^B-NMR (CDCl_3_) δ 30.9. HRMS (ESI, positive ion): *m/z* [M+H]^+^, found 466.2503. C_2__5_H_3__2_BN_3_O_5_ requires 466.2519.


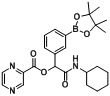
*2-(Cyclohexylamino)-2-oxo-1-(3-(4,4,5,5-tetramethyl-1,3,2-dioxaborolan-2-yl)phenyl)ethyl pyrazine-2-carboxylate* (**B12**). The desired compound (195 mg, 42% yield) was prepared by General Procedure B using pyrazine-2-carboxylic acid (124 mg, 1.00 mmol), *m*-formylphenylboronic acid ester (232 mg, 1.00 mmol), and cyclohexyl isocyanide (0.124 mL, 1.00 mmol); mp = 140 °C. ^1^H-NMR (CDCl_3_) δ 9.32 (s, 1H), 8.78 (s, 1H), 8.73 (s, 1H), 7.95 (s, 1H), 7.80 (d, *J* = 7.2 Hz, 1H), 7.65 (d, *J* = 7.8 Hz, 1H), 7.40 (t, *J* = 7.2, 15 Hz, 1H), 6.42 (br.), 6.34 (s, 1H), 3.84–3.77 (m, 1H), 1.88–1.87 (m, 2H), 1.73–1.66 (m, 4H), 1.61–1.58 (m, 1H), 1.38–1.33 (m, 14H), 1.26–1.16 (m, 3H). ^13^C-NMR (CDCl_3_) δ 166.7, 162.5, 148.0, 146.5, 144.4, 143.0, 135.7, 134.4, 134.2, 130.5, 128.3, 83.9, 48.3, 32.8, 32.7, 25.4, 24.9, 24.8, 24.61. ^11^B-NMR (CDCl_3_) δ 30.4. HRMS (ESI, positive ion): *m/z* [M+H]^+^, found 466.2512. C_25_H_32_BN_3_O_5_ requires 466.2519.

### 3.4. *In Vitro* Biological Evaluation

#### *In Vitro* Cytotoxicity Assay

The anti-tumor activities of boron-containing analogs against lung (A549), breast (MDA-MB-231), and liver (HepG2) cancer cell lines were evaluated using a microculture tetrazolium test (MTT, Sigma-Aldrich, Saint Louis, MO, USA). Briefly, tumor cells or fibroblasts (5000 cells in 100 μL complete medium per well) were seeded into a 96-well plate (Nunc, Roskilde, Denmark). After incubation at 37 °C for 24 h, 100 μL of culture medium with or without boron-containing analogs was added to each well in triplicate for 48 h of consecutive incubation at 37 °C. In the treatment of each cell line, cells were incubated for another 24, 48 and 72 h. MTT solution 50 μL (Sigma) was added to each well. Following incubation at 37 °C for an additional 4 h, supernatants were removed and 100 μL DMSO was added to dissolve the MTT-formazan product. The plate was read using a microplate reader (Labsystems, Helsinki, Finland) at 550 nm. The cell inhibitions at 20, 10, 5, 2.5 μg/mL of boron-containing analogs were estimated and the IC50 for boron-containing analogs calculated for the control group was set to 100% [36].

## 4. Conclusions

In conclusion, a convenient and efficient microwave-assisted Passerini MCR under aqueous conditions was developed. Broad ranges of boron-containing α-acyloxyamides were synthesized in moderate to good yields using this method. In addition, a simple acid/base extraction protocol was developed, which enabled simple and effective purification of these boron-containing compounds. This is a major achievement that renders this synthetic strategy suitable for use in the library synthesis of boron molecules. All of the synthesized analogs were screened using the MTT assay, with two compounds found to be active against the HepG2 cell line. Further structure–activity relationship evaluations based on these two analogs is currently ongoing, and the results will be reported in due course.

## Supplementary Materials

Supplementary materials can be accessed at: http://www.mdpi.com/1420-3049/18/8/9488/s1.
