# Supplementary material for: Efficient Synthesis of Boron-Containing α-Acyloxyamide Analogs via Microwave Irradiation
Source: Molecules. 2013 Aug 8;18(8):9488–511. doi: 10.3390/molecules18089488 (PMC6270651; doi:10.3390/molecules18089488)

Supplementary Materials

Figure S1. HRMS (ESI, positive ion) $[M+H]^+$ of Compound (**A1**).

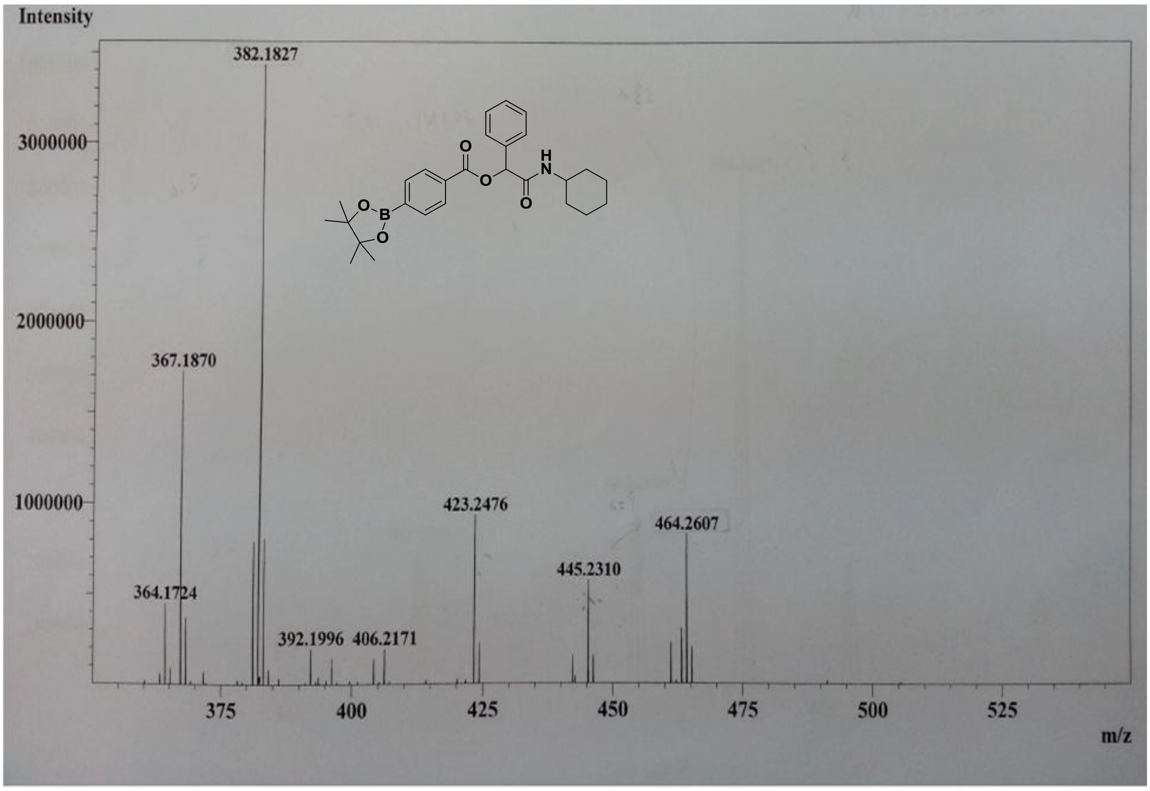

A

Figure S2. 600 MHz ^{1}H-NMR of Compound (**A1**) in Chloroform-*d*.

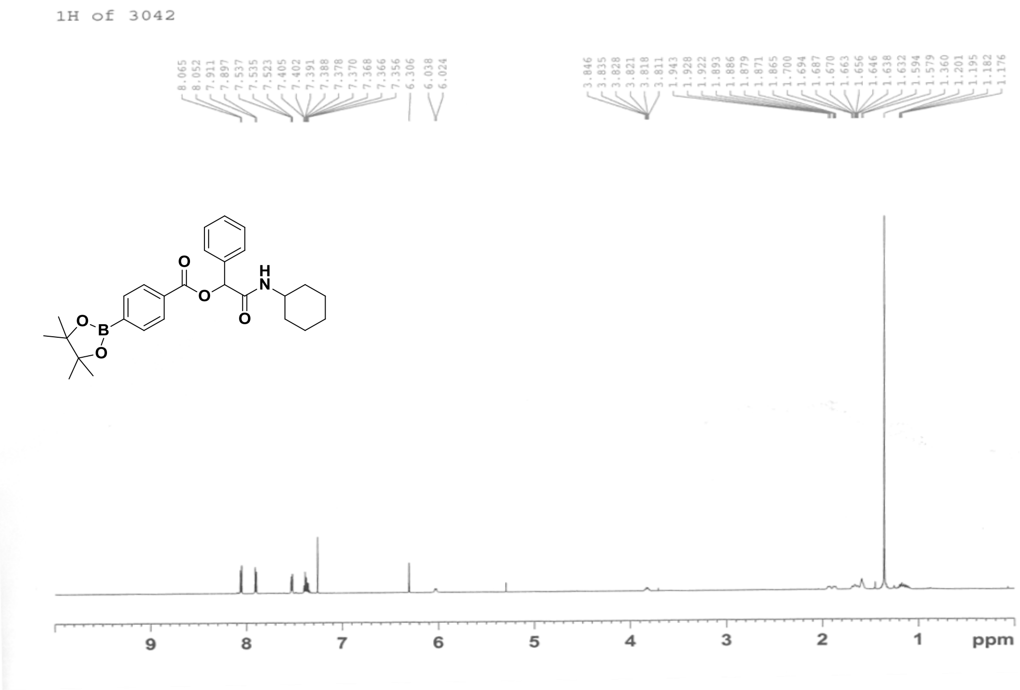

Figure S3. 600 MHz ^{13}C-NMR of Compound (**A1**) in Chloroform-*d*.

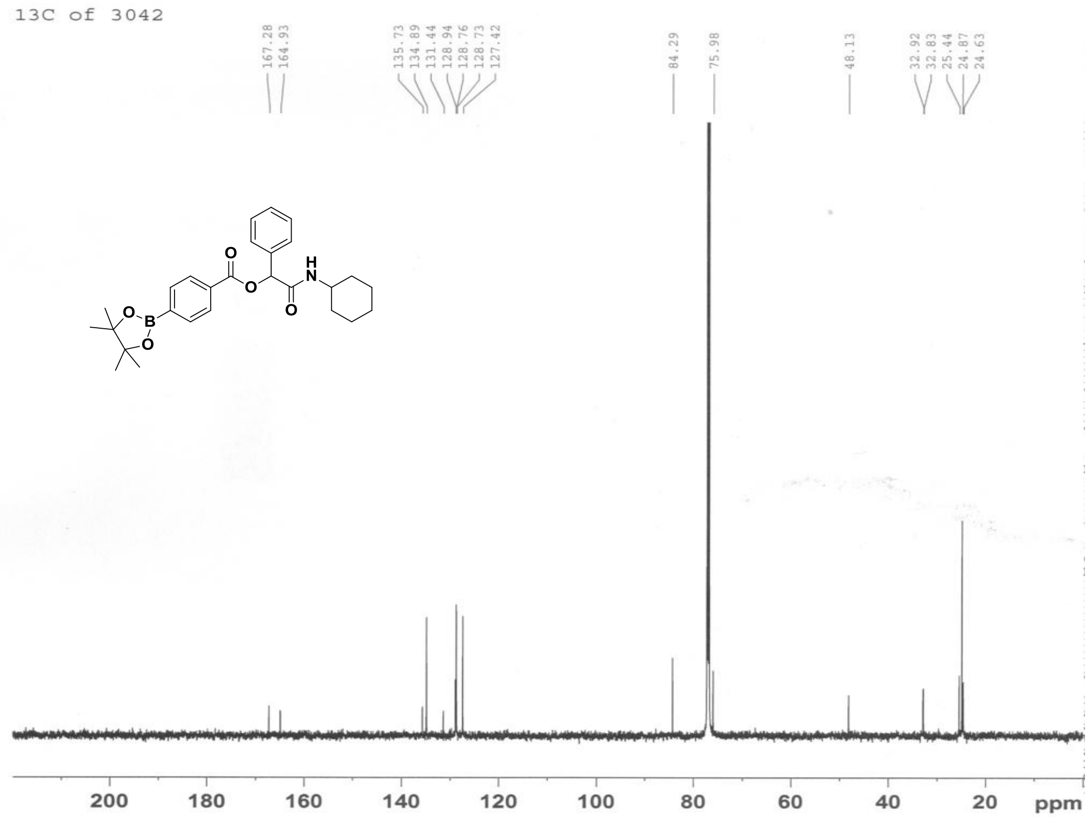

Figure S4. 600 MHz ^{11}B-NMR of Compound (**A1**) in Chloroform-*d*.

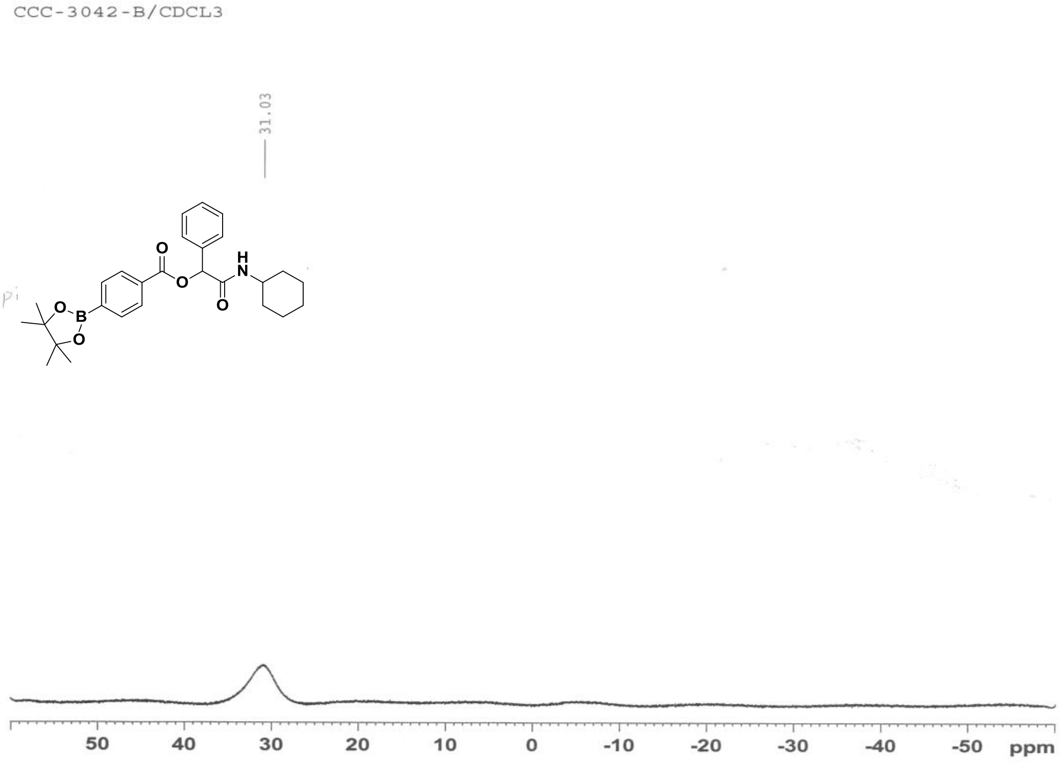

Figure S5. HRMS (ESI, positive ion) $[M+H]^+$ of Compound (**A2**).

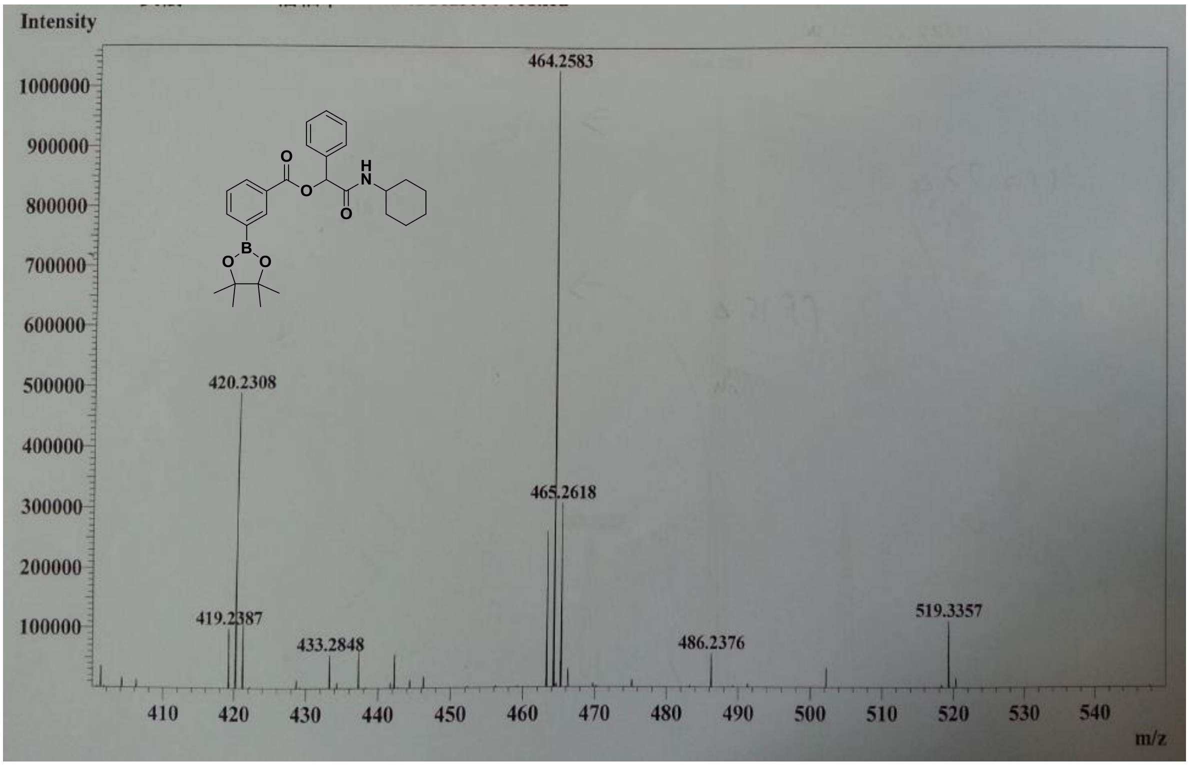

Figure S6. 600 MHz ^{13}C-NMR of Compound (**A2**) in Chloroform-*d*.

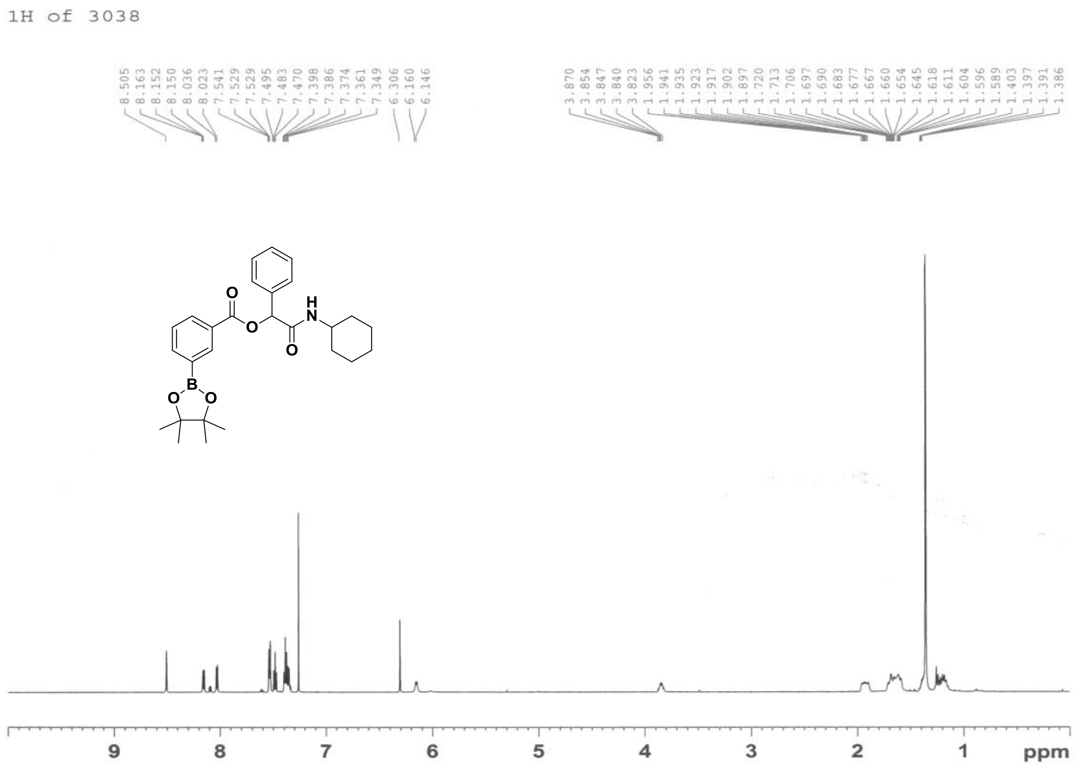

Figure S7. 600 MHz ^{13}C-NMR of Compound (**A2**) in Chloroform-*d*.

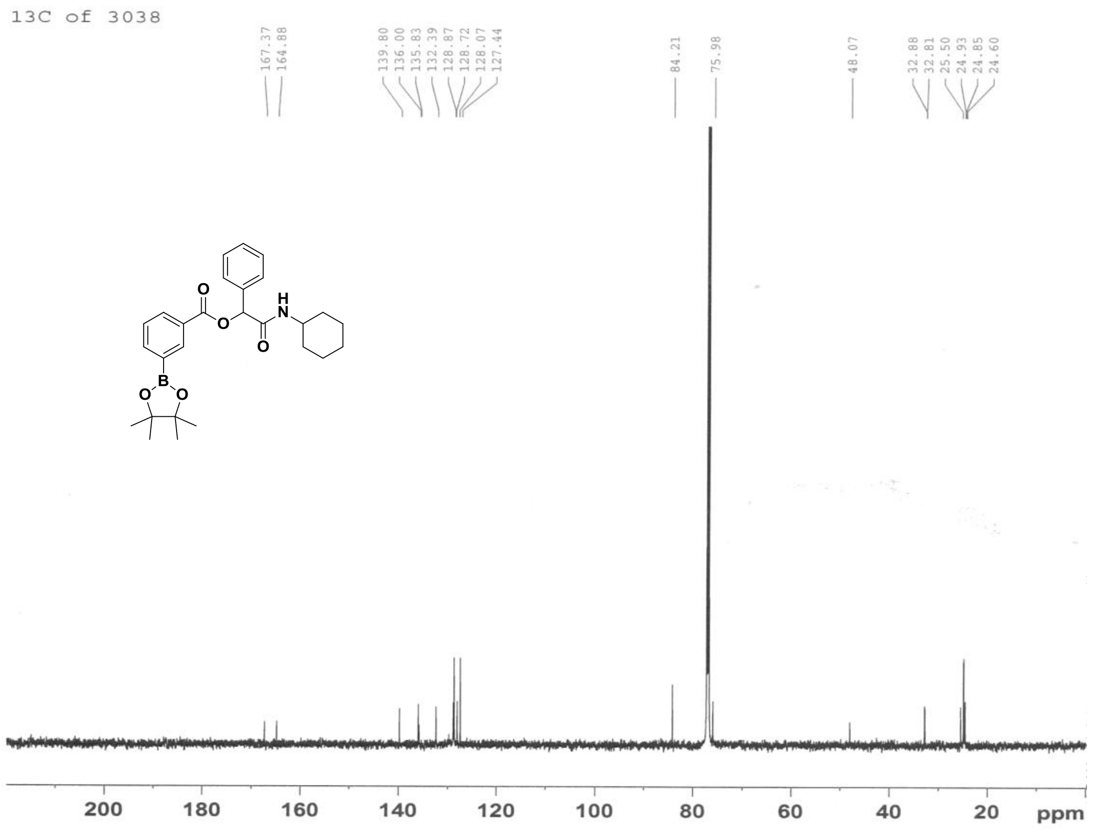

Figure S8. 600 MHz ^{11}B-NMR of Compound (**A2**) in Chloroform-*d*.

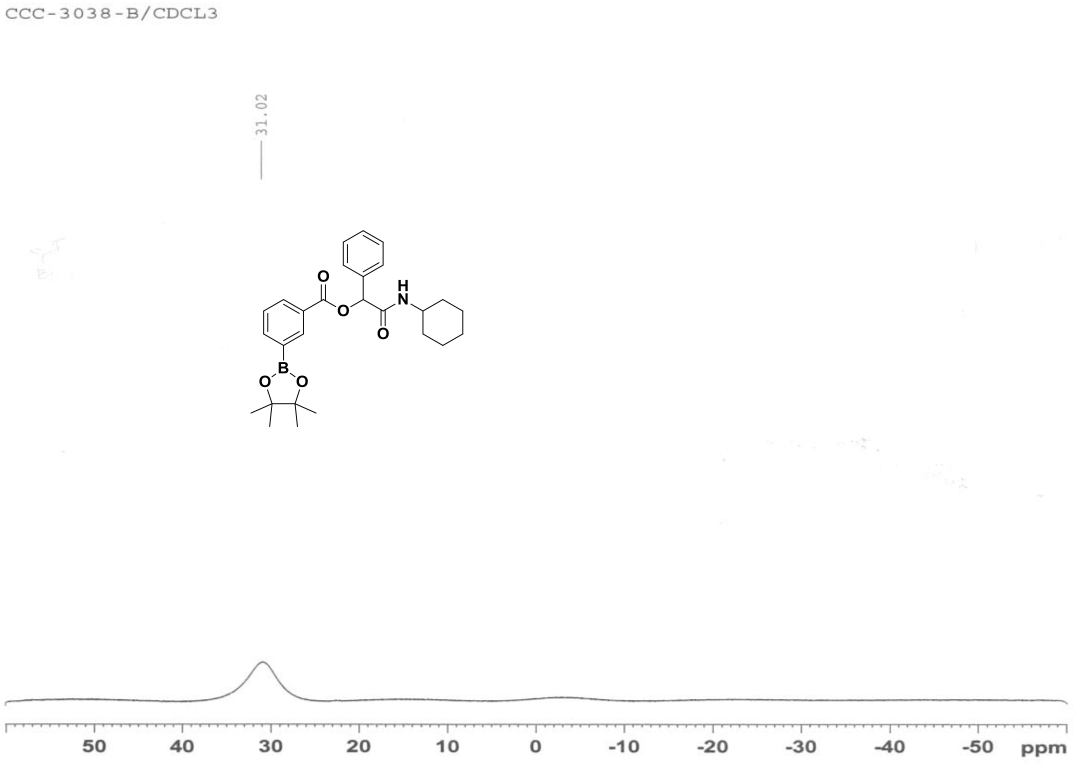

Figure S9. HRMS (ESI, positive ion) $[M+H]^+$ of Compound (**A3**).

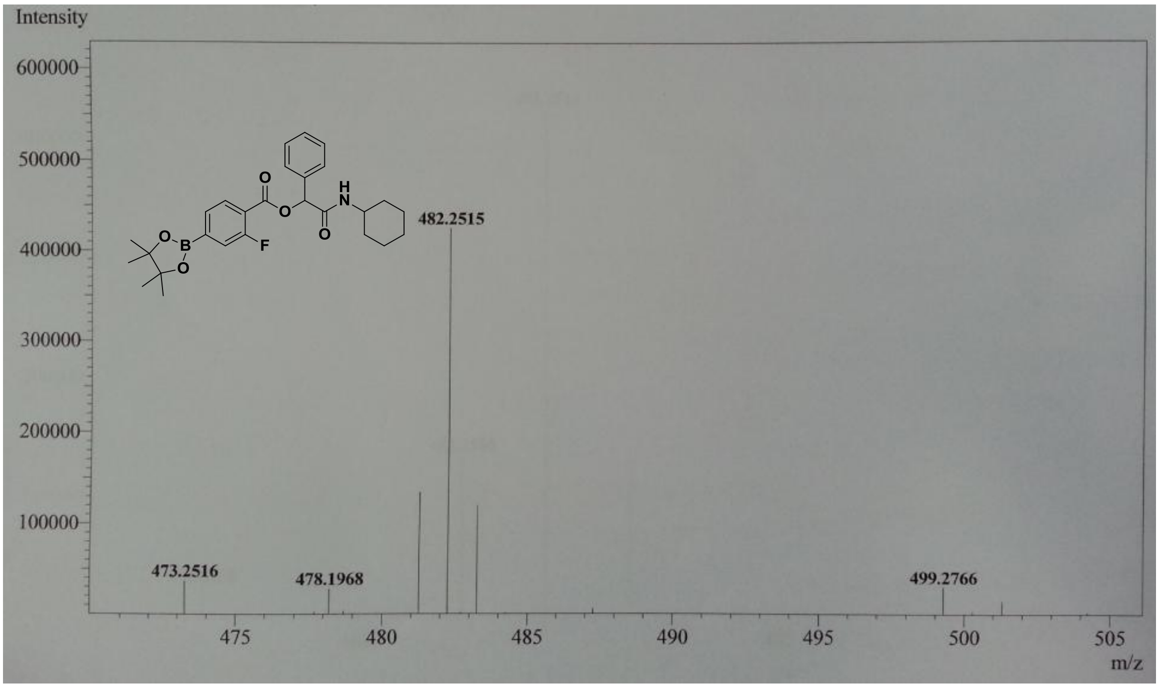

Figure S10. 600 MHz ^{1}H-NMR of Compound (**A3**) in Chloroform-*d*.

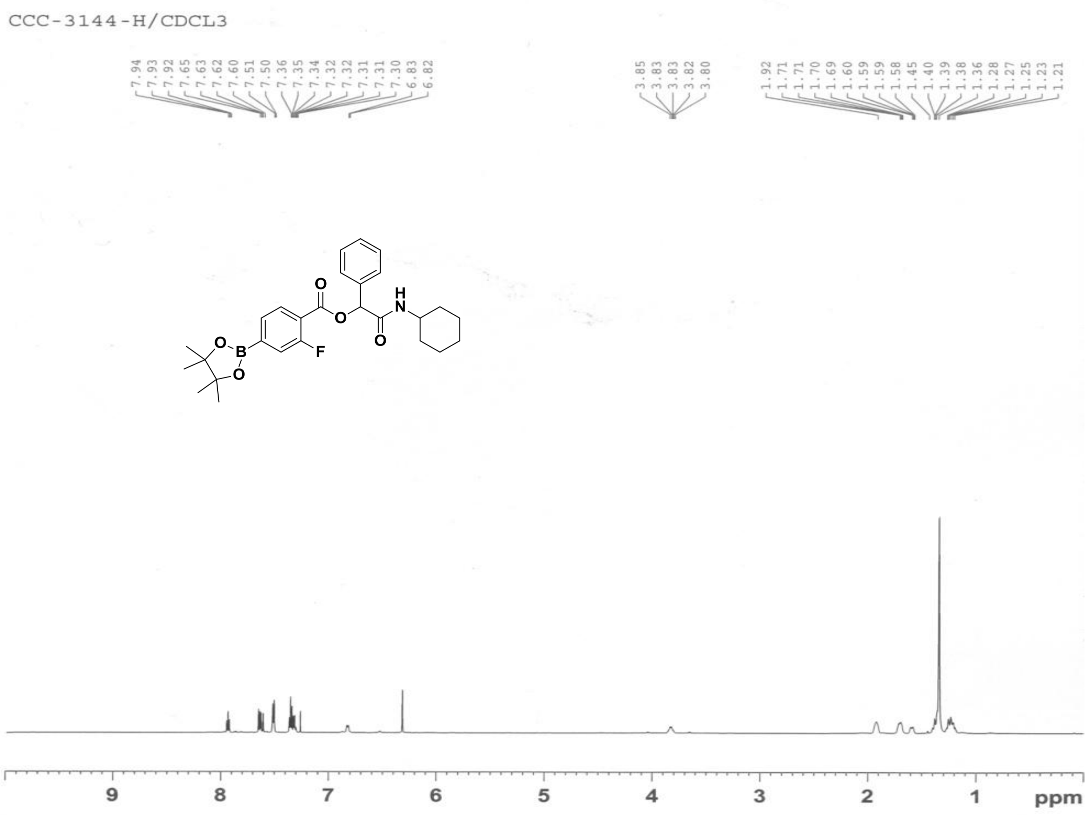

Figure S11. 600 MHz ^{13}C-NMR of Compound (**A3**) in Chloroform-*d*.

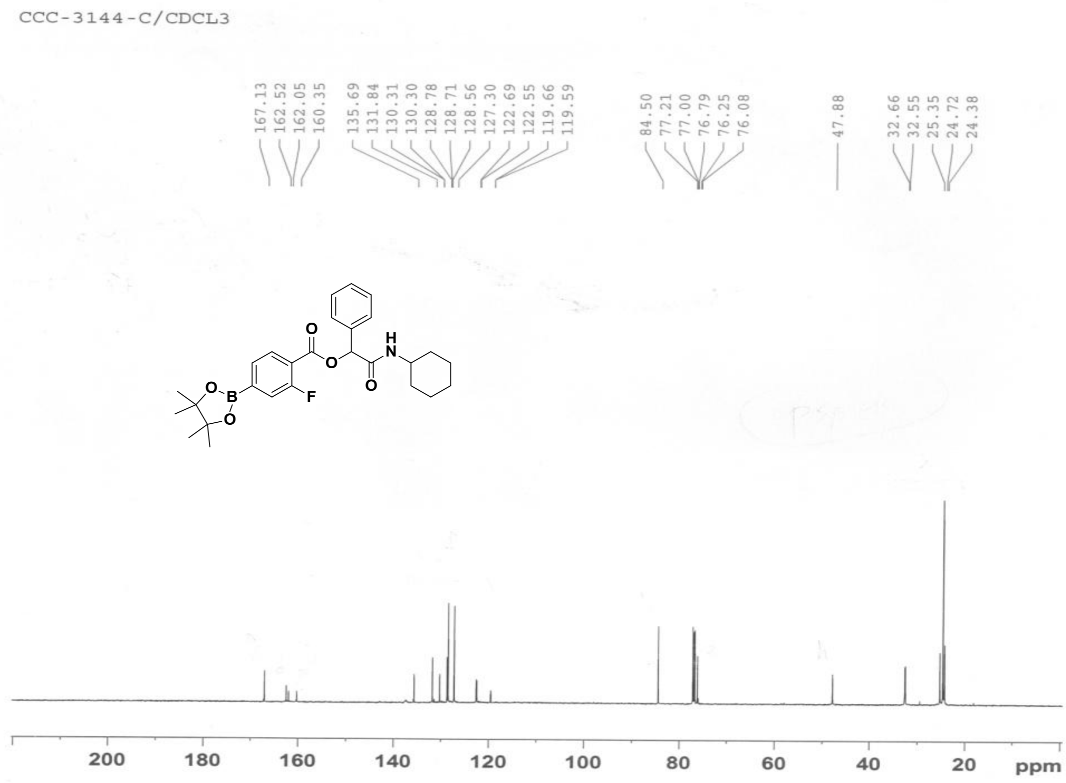

Figure S12. 600 MHz ^{11}B-NMR of Compound (**A3**) in Chloroform-*d*.

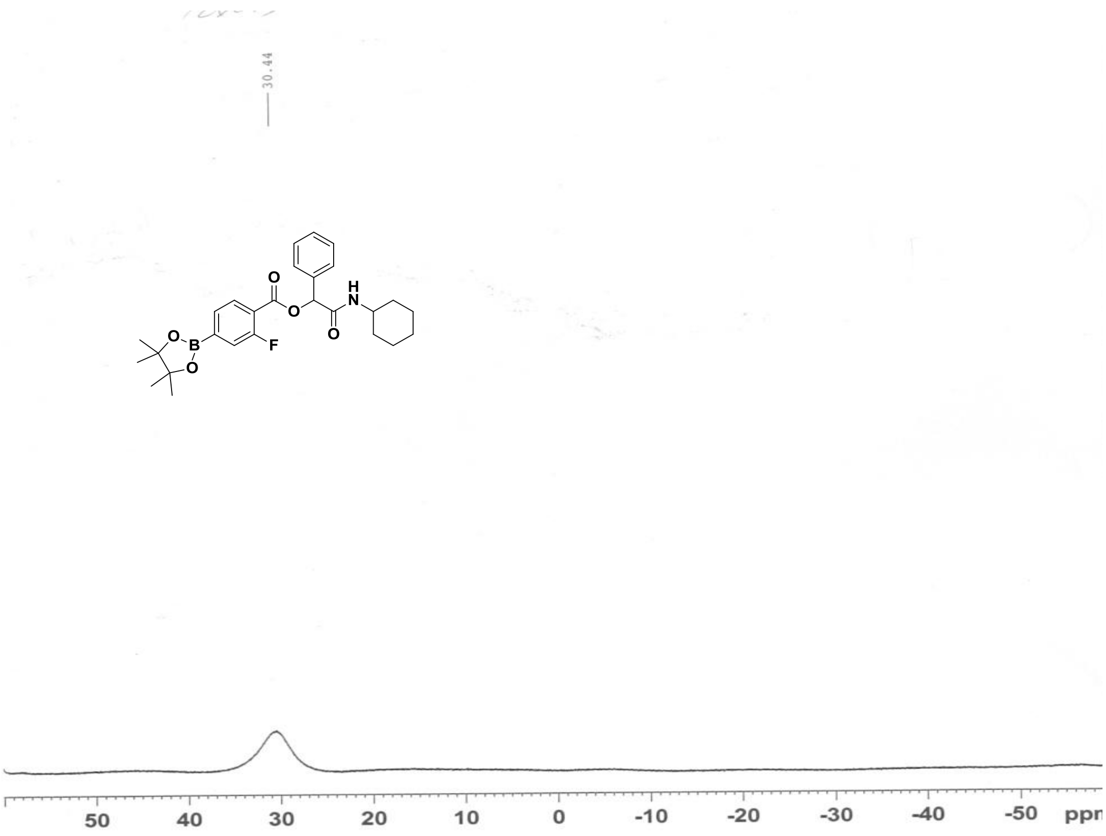

Figure S13. HRMS (ESI, positive ion) $[M+H]^+$ of Compound (**A4**).

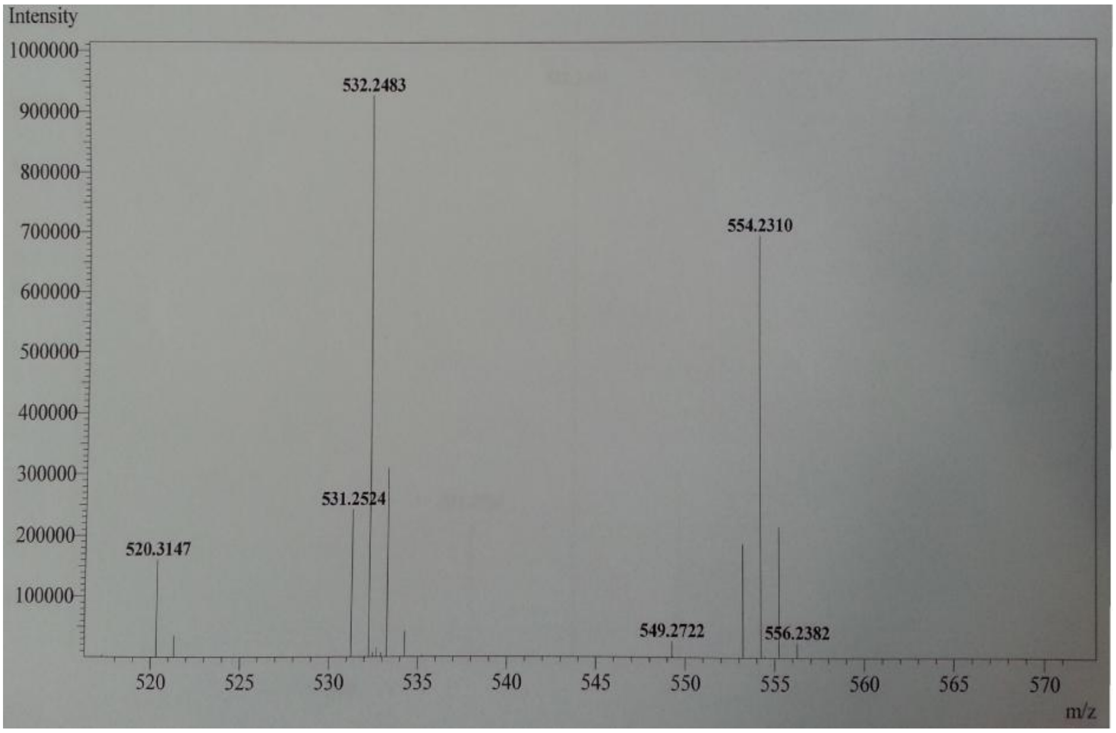

Figure S14. 600 MHz ^{1}H-NMR of Compound (**A4**) in Chloroform-*d*.

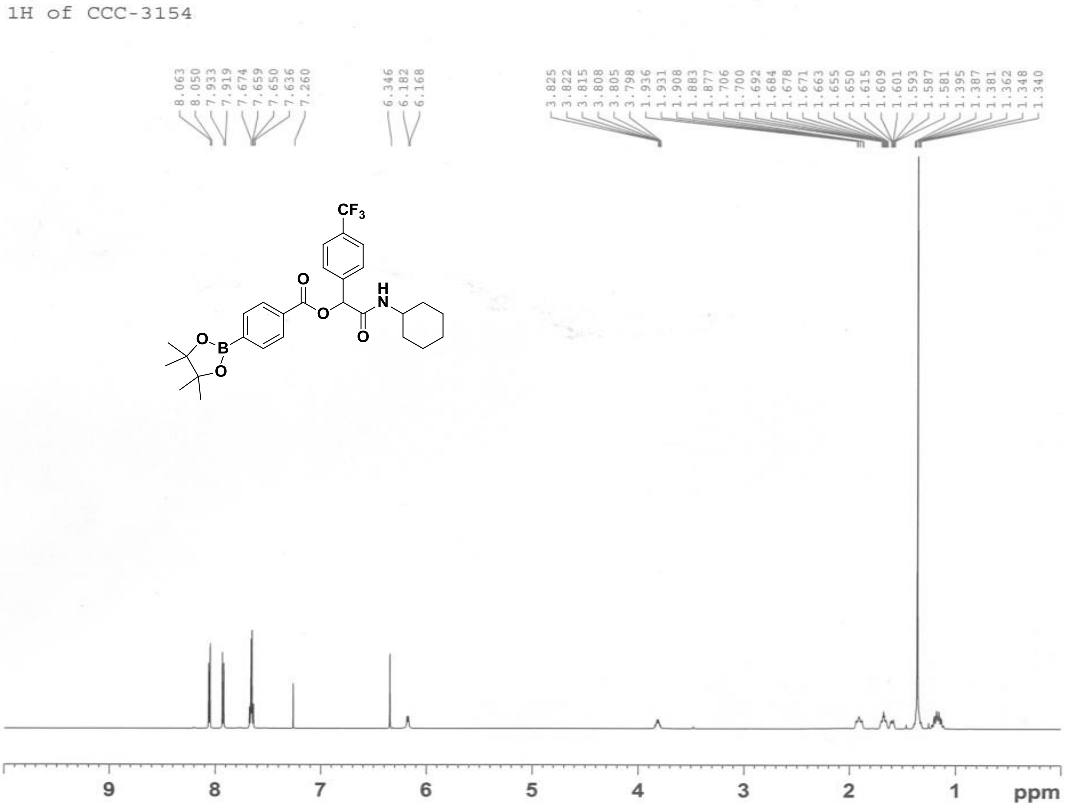

Figure S15. 600 MHz ^{13}C-NMR of Compound (**A4**) in Chloroform-*d*.

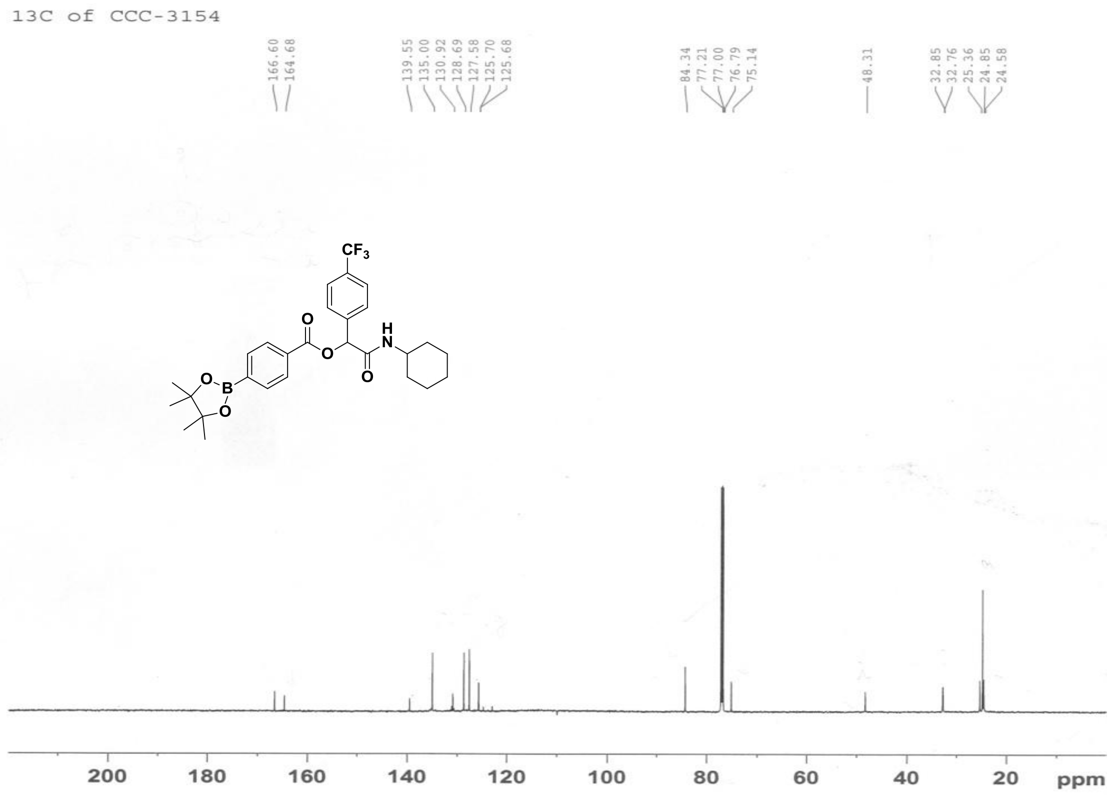

Figure S16. 600 MHz ^{11}B-NMR of Compound (**A4**) in Chloroform-*d*.

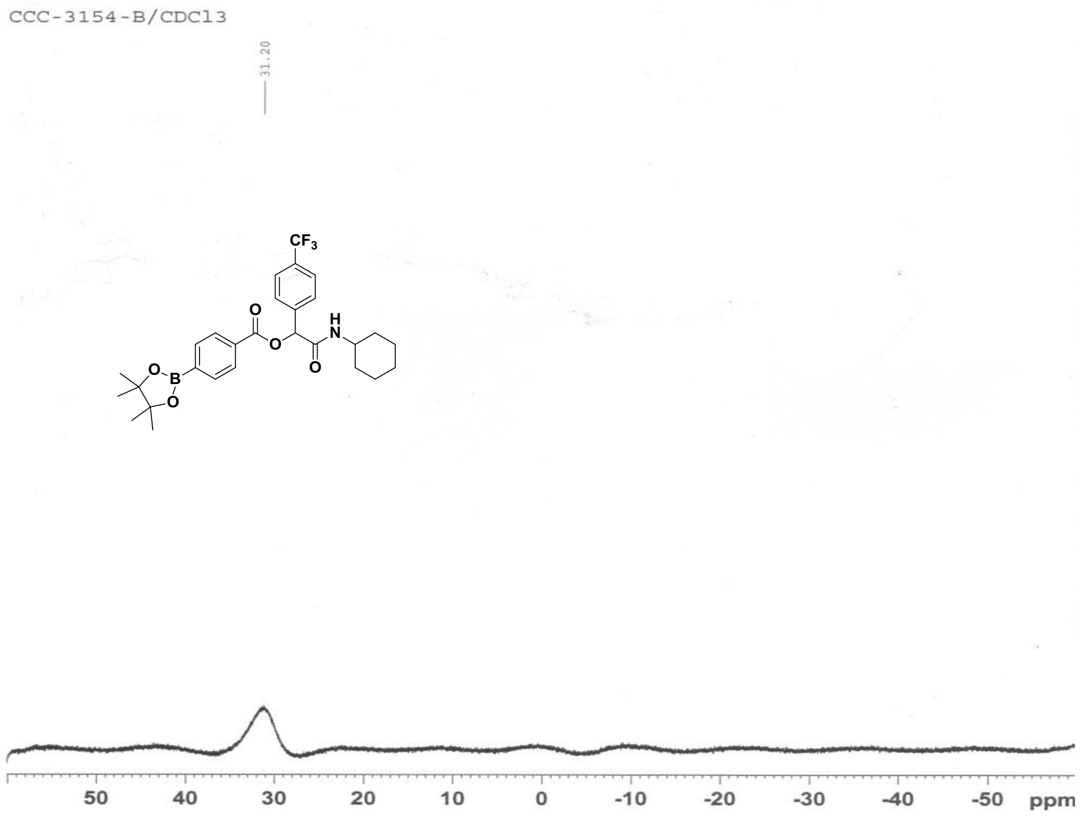

Figure S17. HRMS (ESI, positive ion) $[M+H]^+$ of Compound (**A5**).

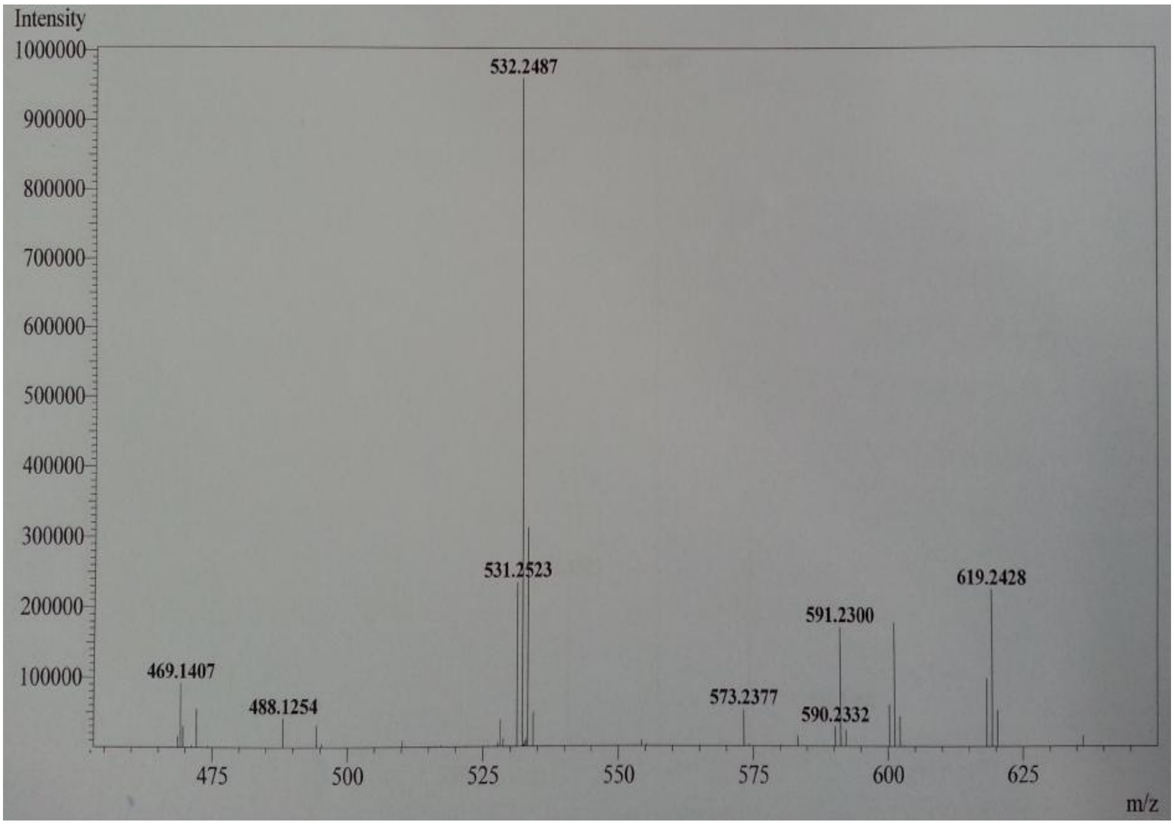

Figure S18. 600 MHz ^{1}H-NMR of Compound (**A5**) in Chloroform-*d*.

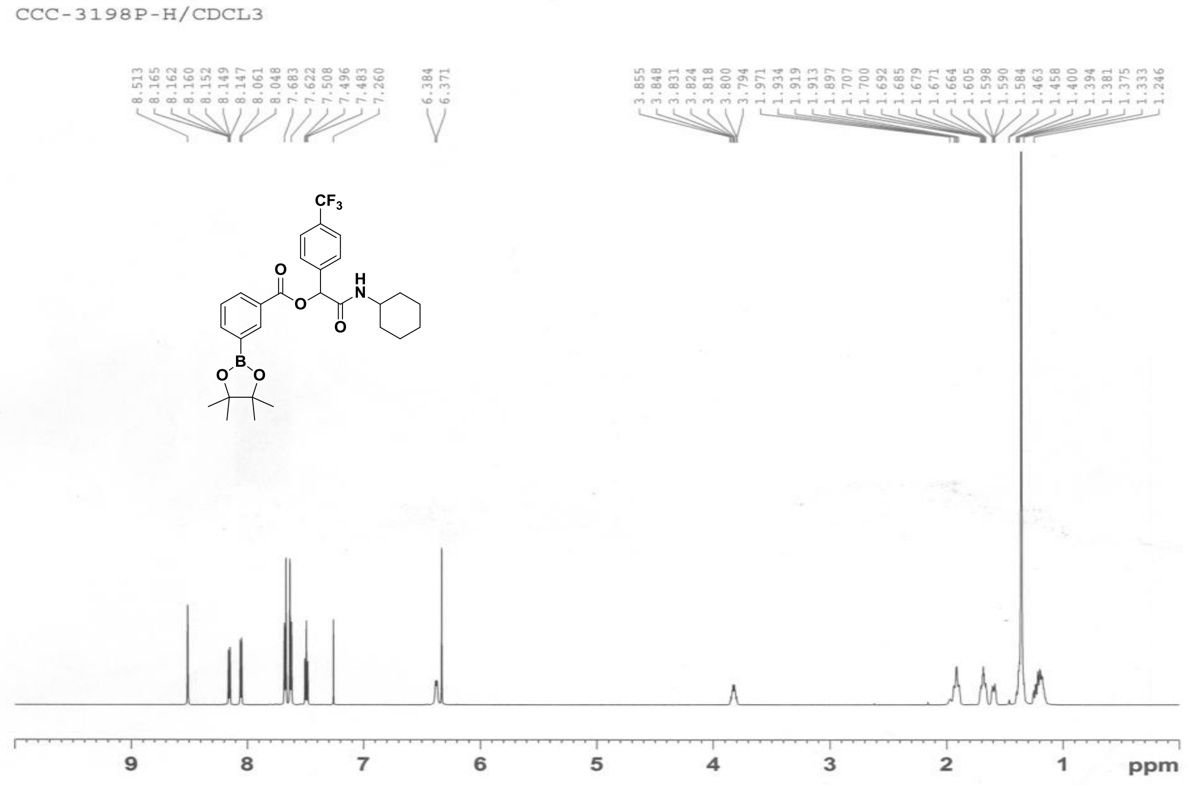

Figure S19. 600 MHz ^{13}C-NMR of Compound (**A5**) in Chloroform-*d*.

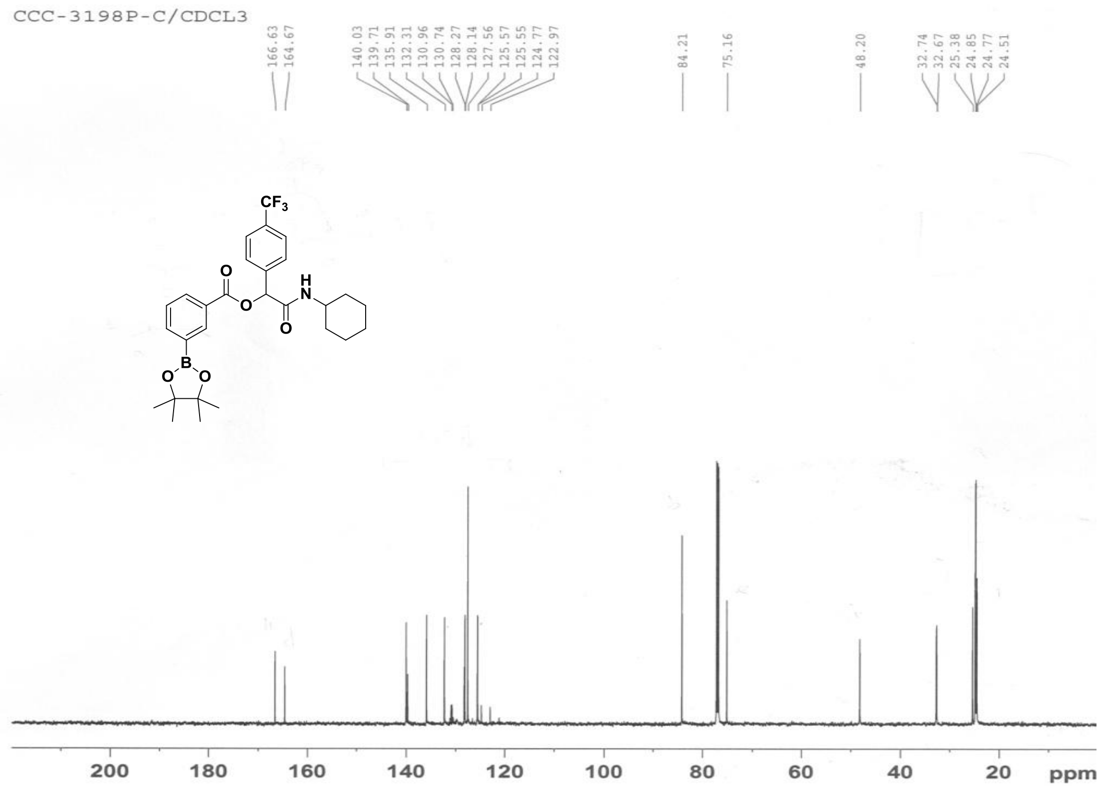

Figure S20. 600 MHz ^{11}B-NMR of Compound (**A5**) in Chloroform-*d*.

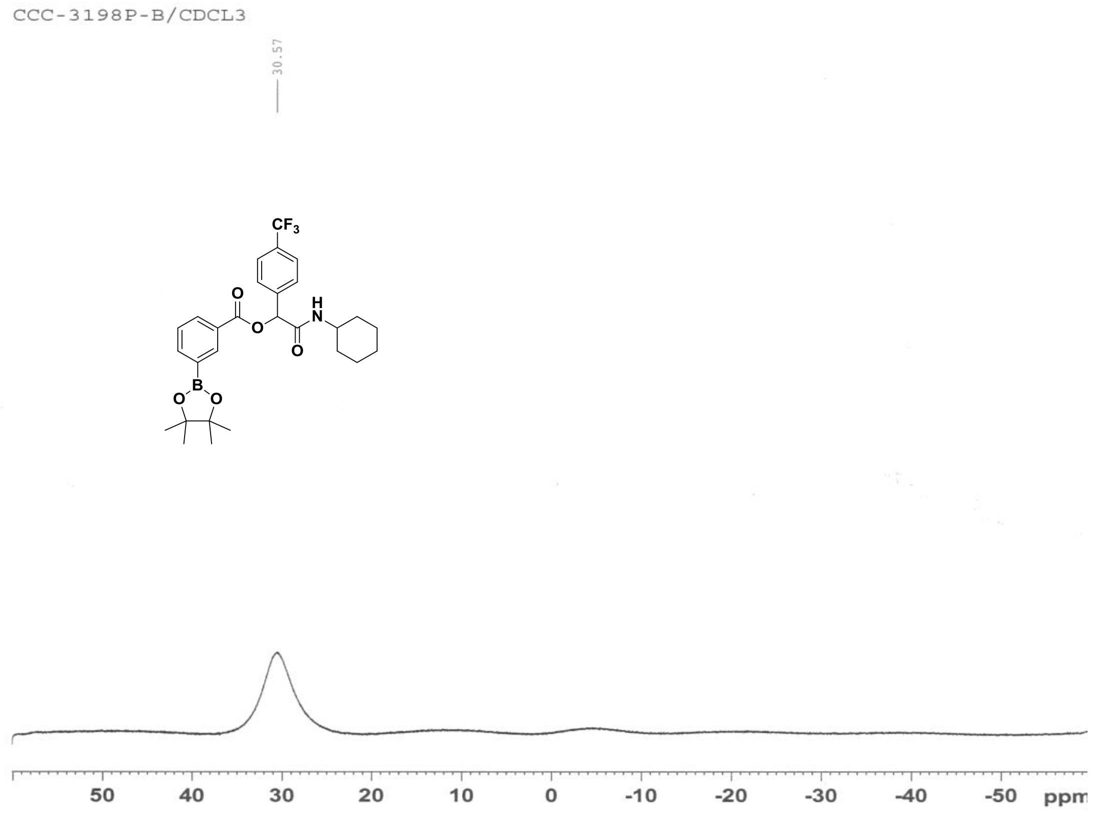

Figure S21. HRMS (ESI, positive ion) $[M+H]^+$ of Compound (**A6**).

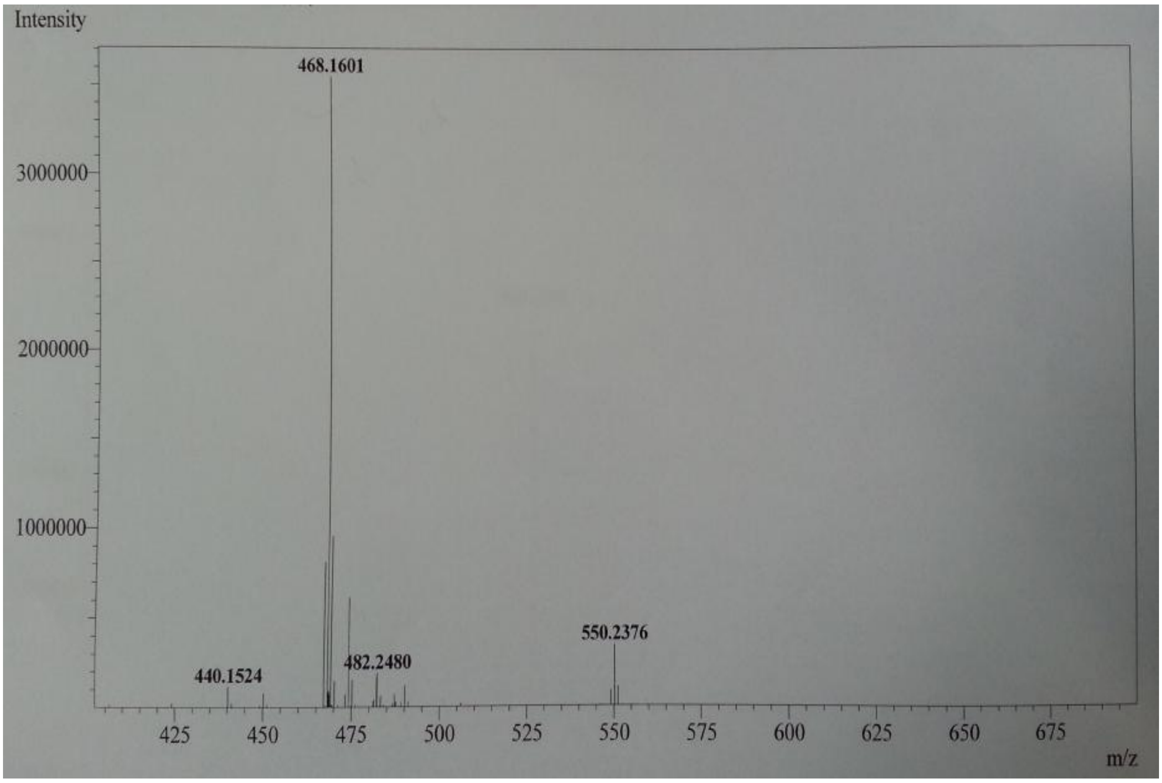

Figure S22. 600 MHz ^{1}H-NMR of Compound (**A6**) in Chloroform-*d*.

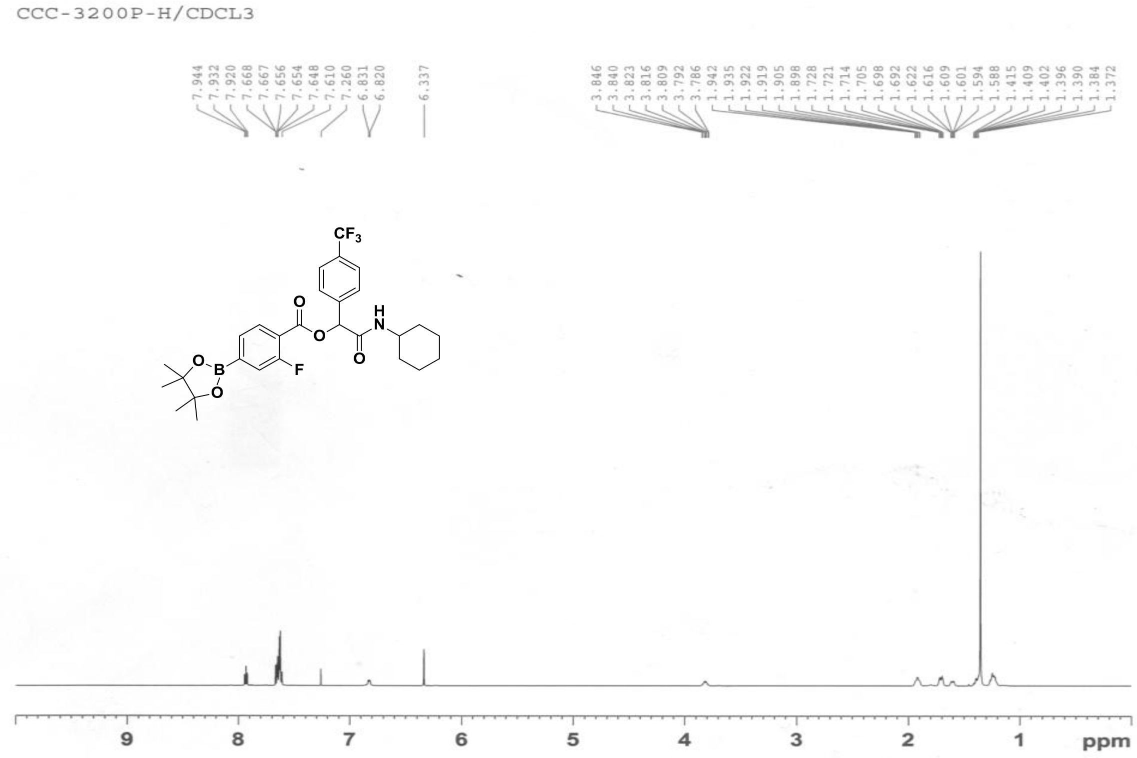

Figure S23. 600 MHz ^{13}C-NMR of Compound (**A6**) in Chloroform-*d*.

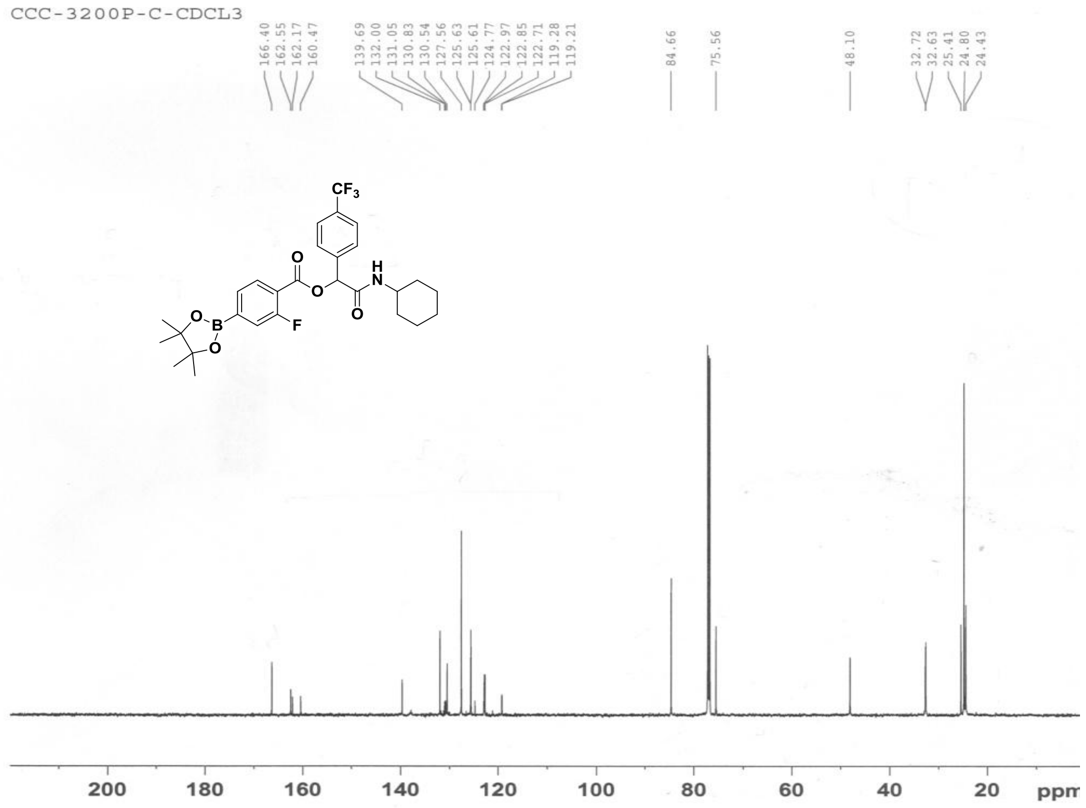

Figure S24. 600 MHz ^{11}B-NMR of Compound (**A6**) in Chloroform-*d*.

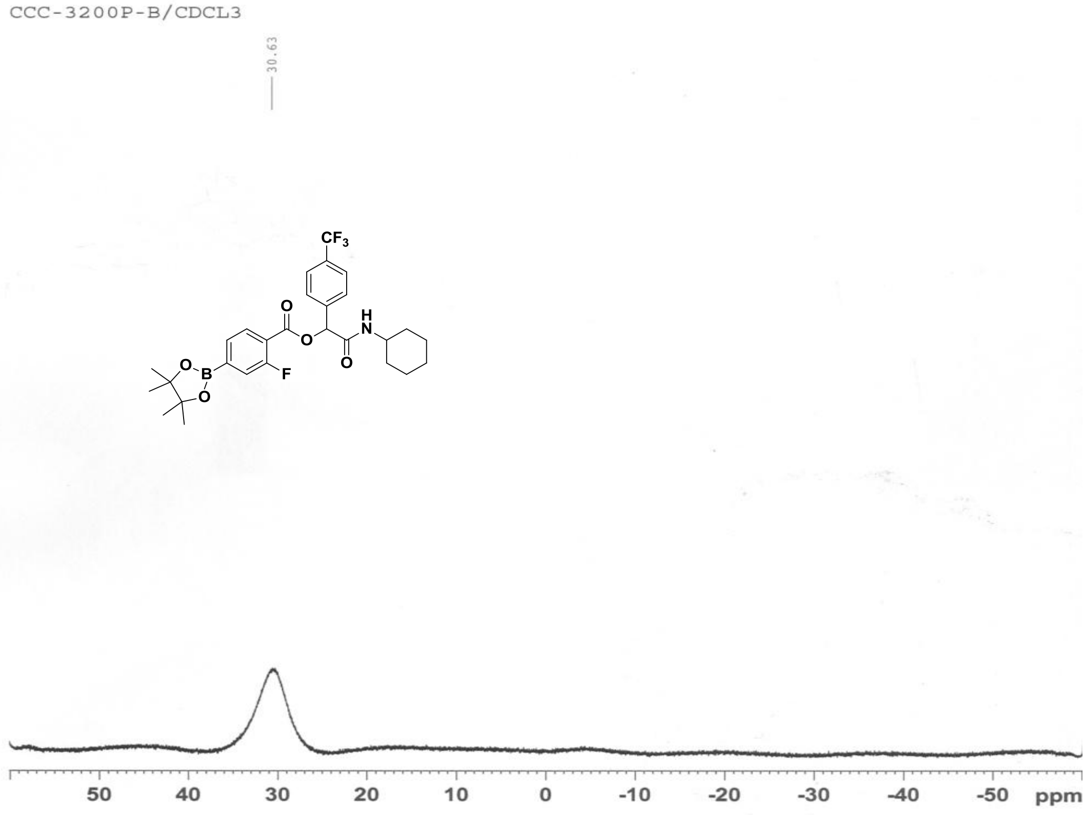

Figure S25. HRMS (ESI, positive ion) [M+H]$^+$ of Compound (**A7**).

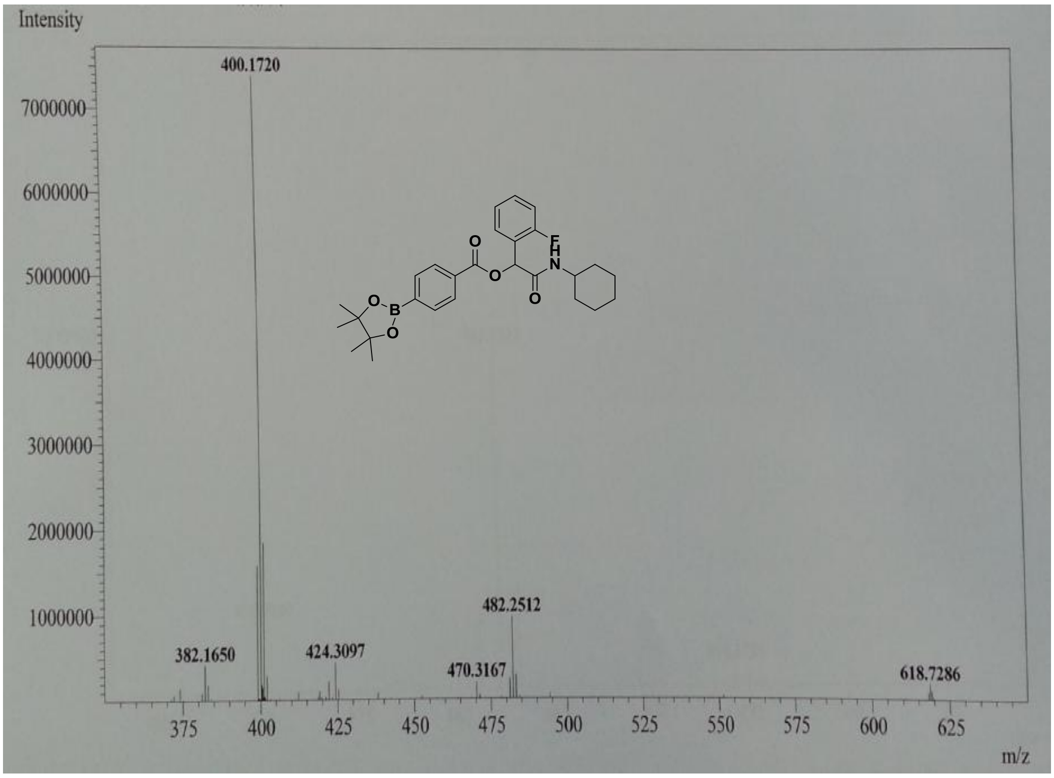

Figure S26. 600 MHz ^{1}H-NMR of Compound (**A7**) in Chloroform-*d*.

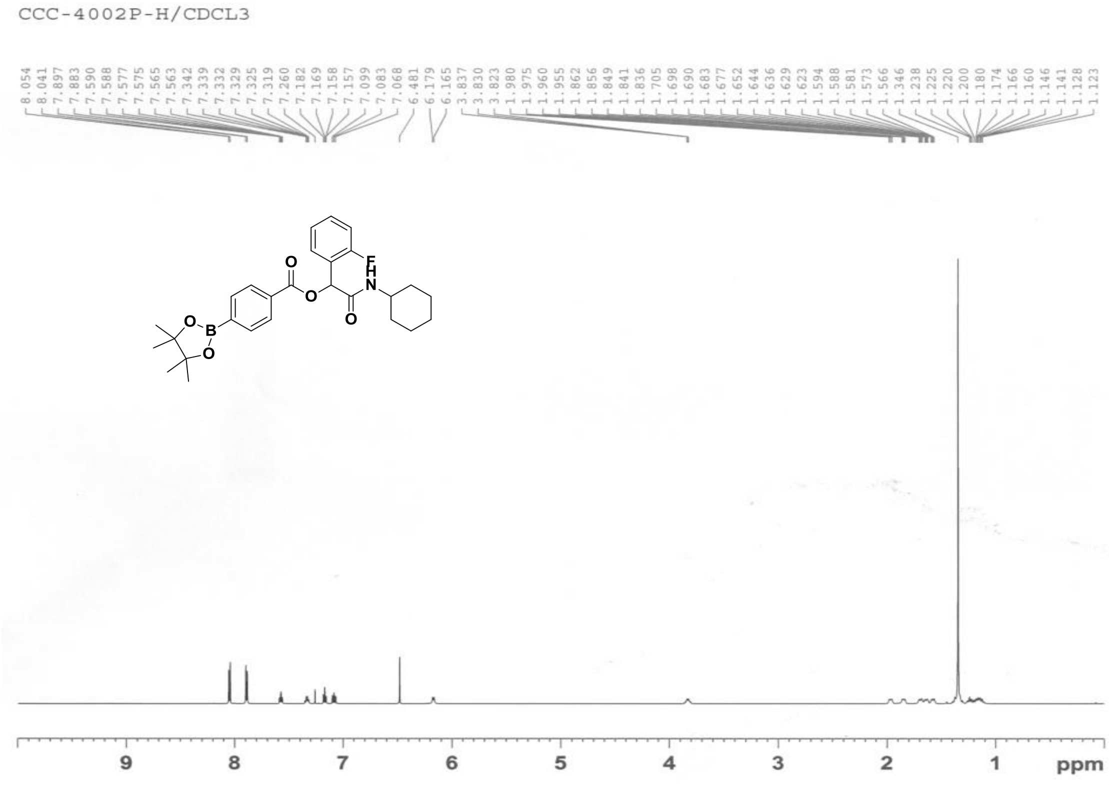

Figure S27. 600 MHz ^{13}C-NMR of Compound (**A7**) in Chloroform-*d*.

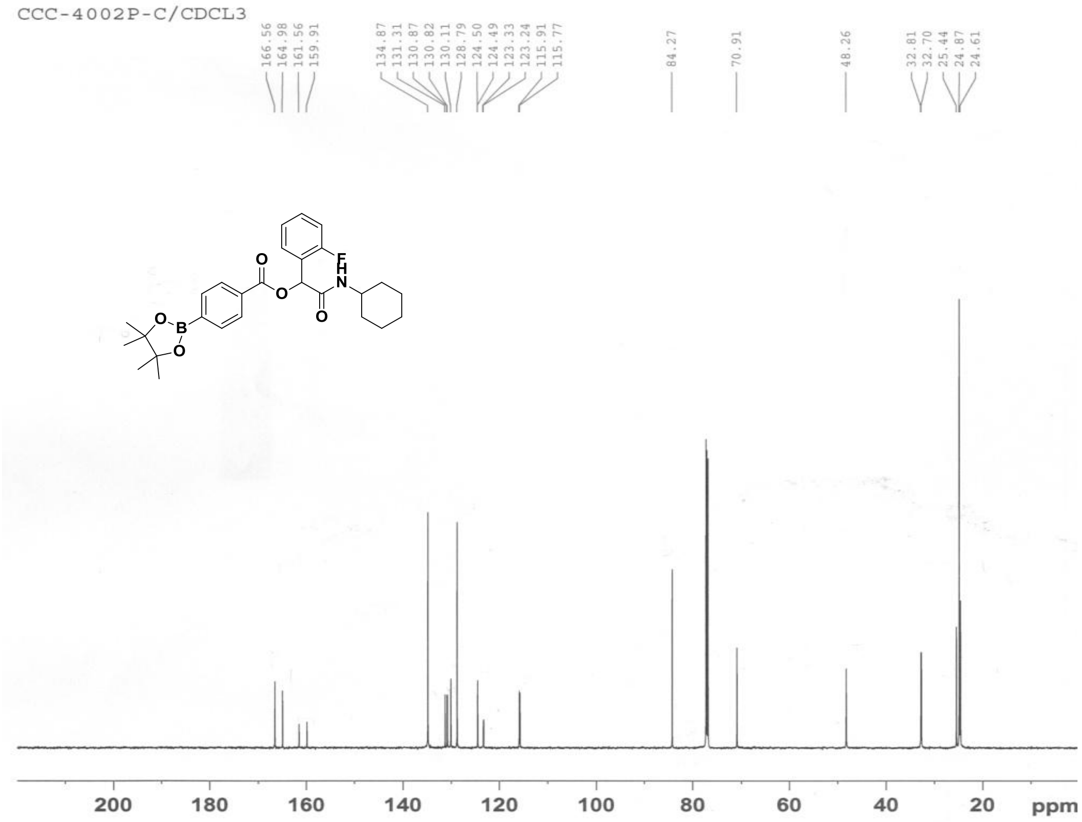

Figure S28. 600 MHz ^{11}B-NMR of Compound (**A7**) in Chloroform-*d*.

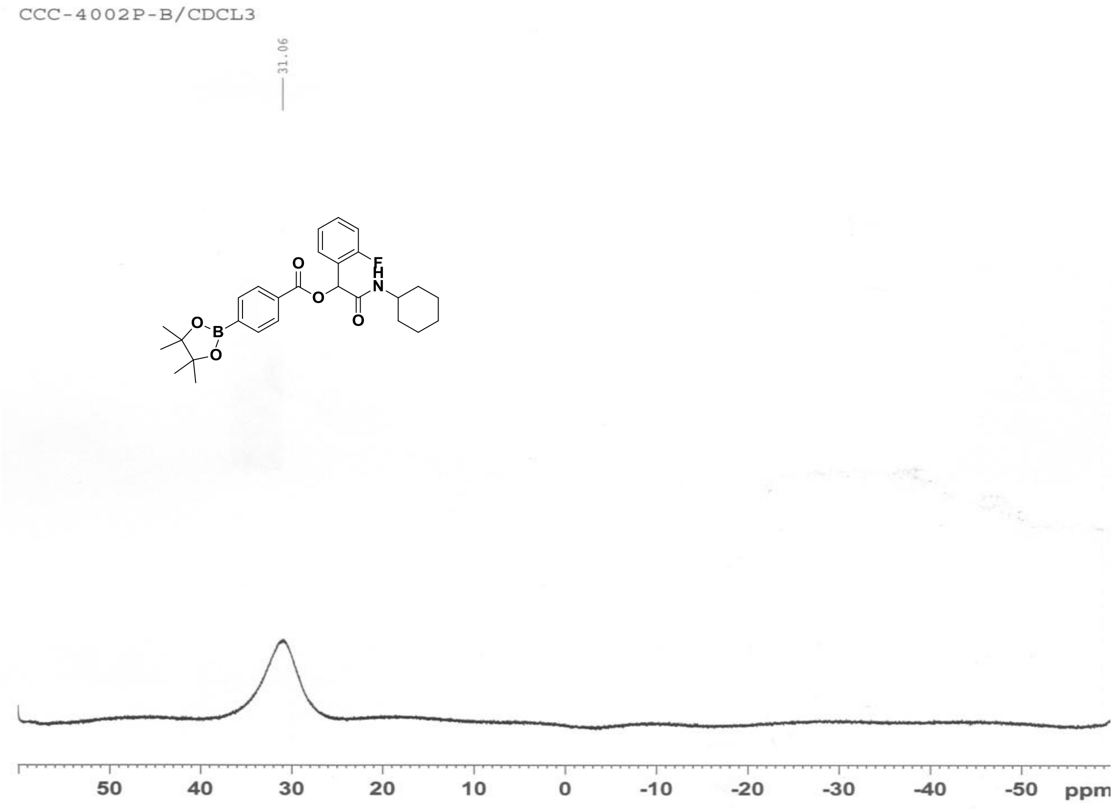

Figure S29. HRMS (ESI, positive ion) $[M+H]^+$ of Compound (**A8**).

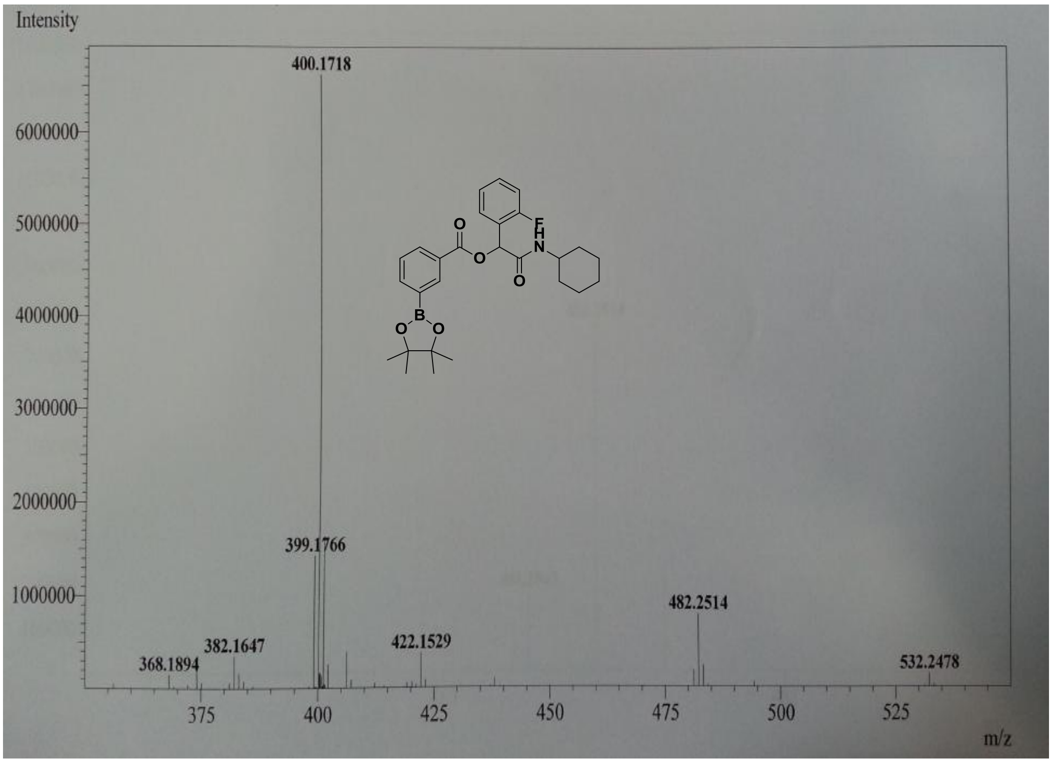

Figure S30. 600 MHz ^{1}H-NMR of Compound (**A8**) in Chloroform-*d*.

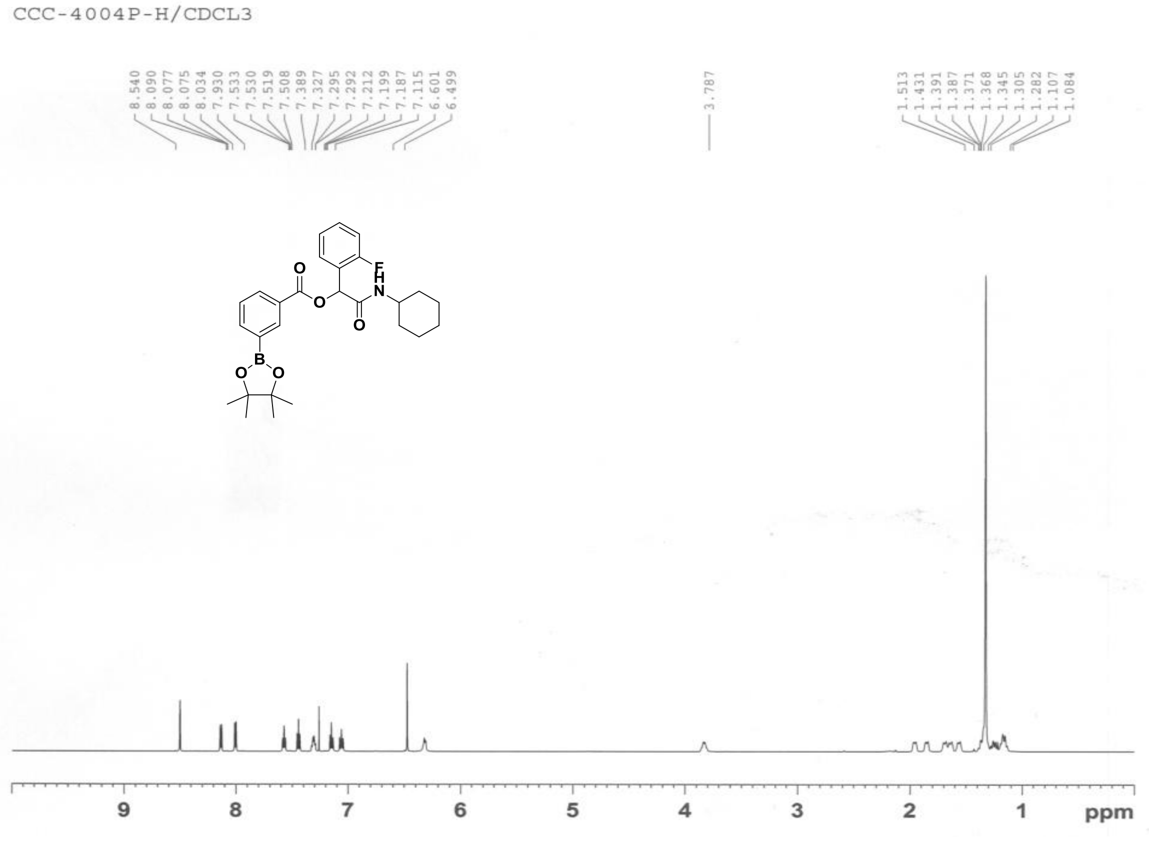

Figure S31. 600 MHz ^{13}C-NMR of Compound (**A8**) in Chloroform-*d*.

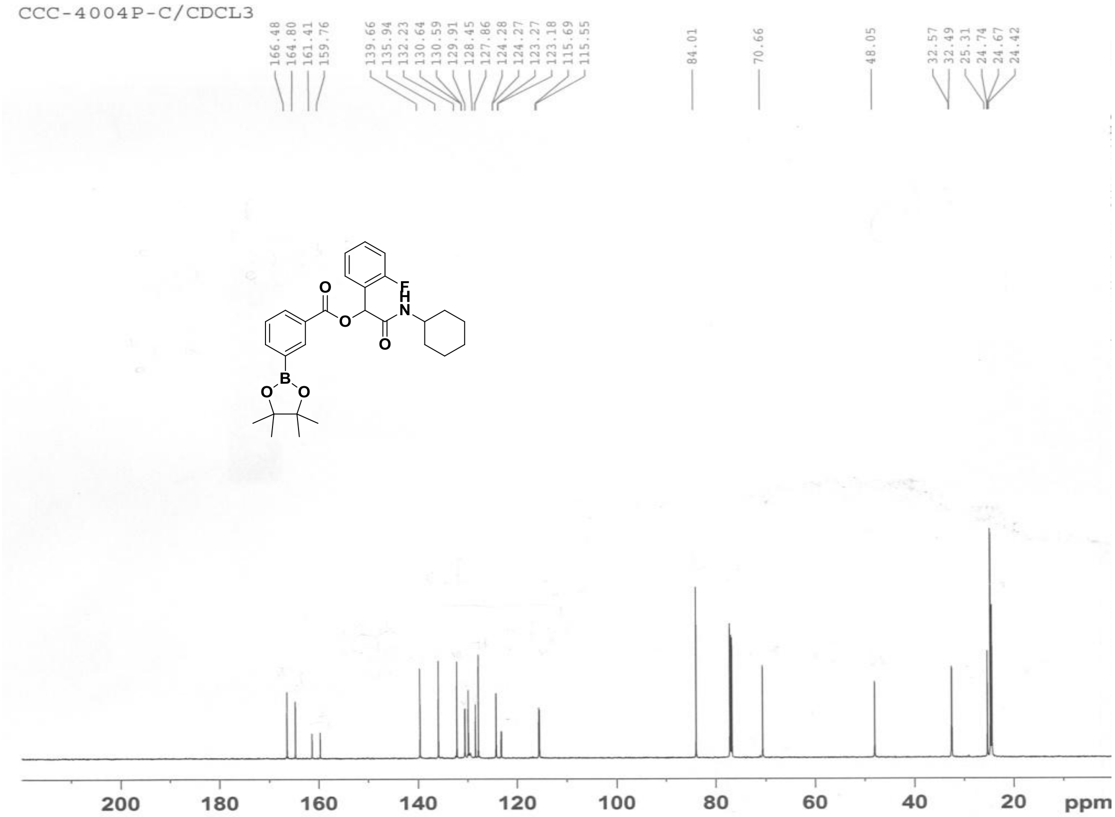

Figure S32. 600 MHz ^{11}B-NMR of Compound (**A8**) in Chloroform-*d*.

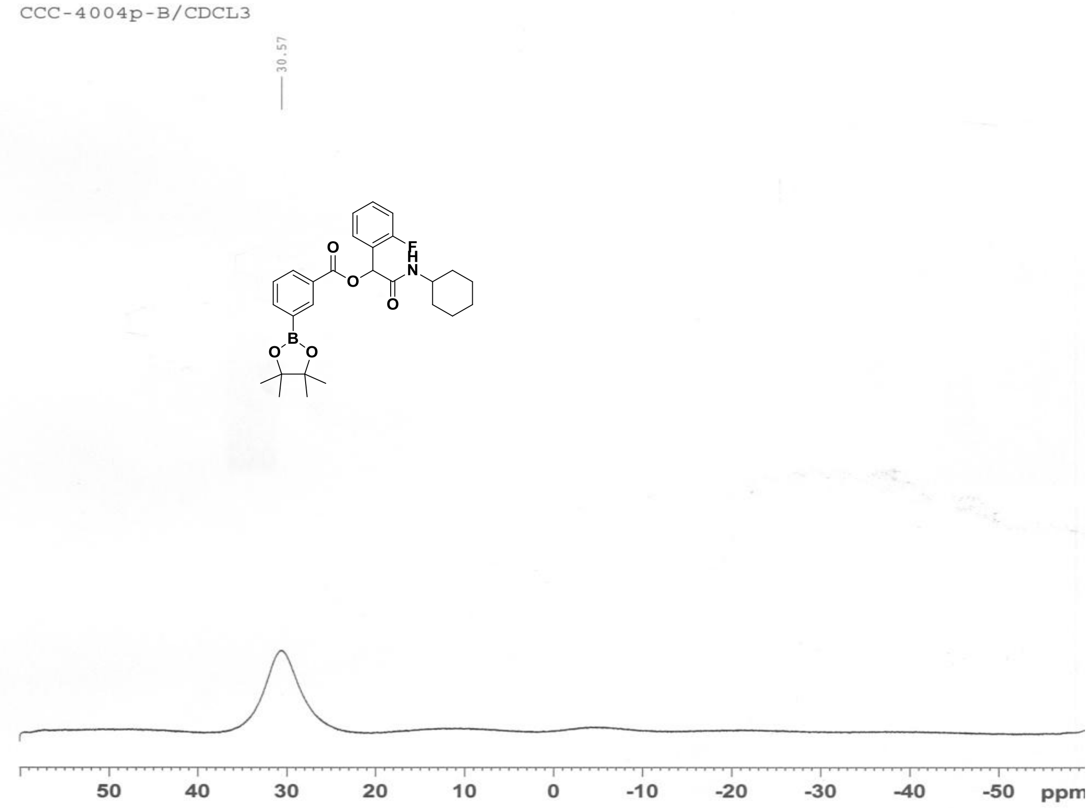

Figure S33. HRMS (ESI, positive ion) [M+H]$^+$ of Compound (**A9**).

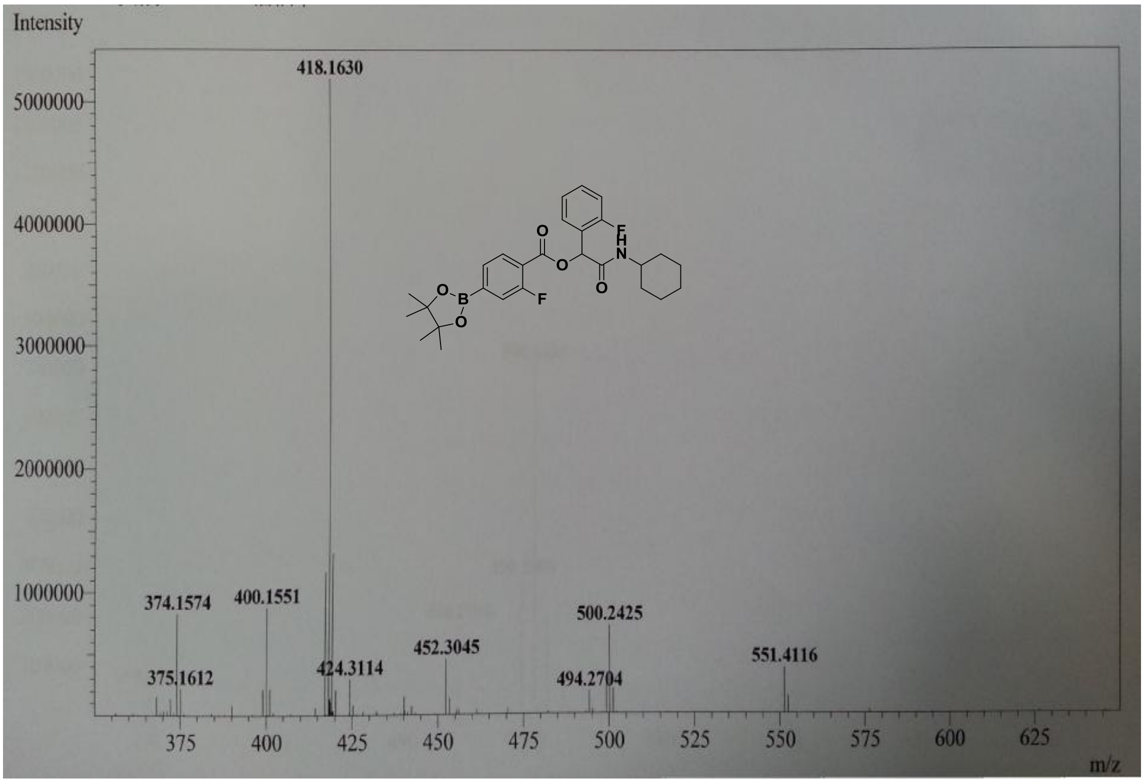

Figure S34. 600 MHz ^{1}H-NMR of Compound (**A9**) in Chloroform-*d*.

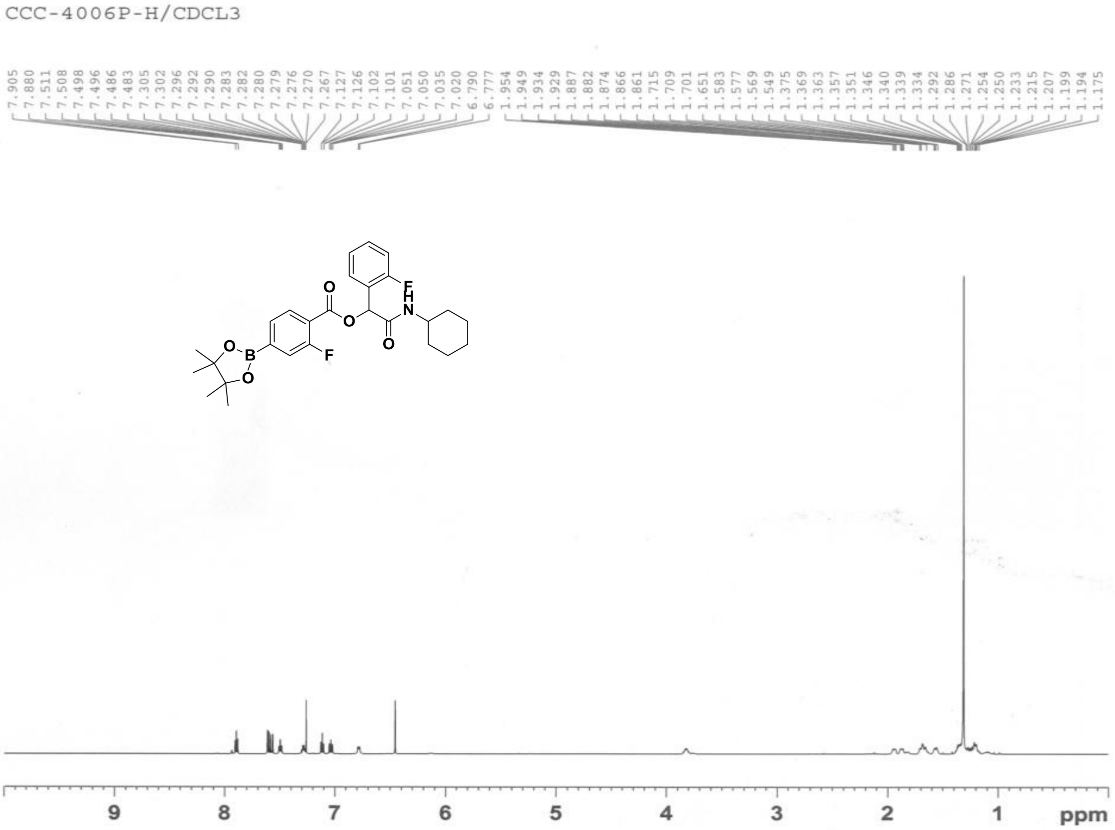

Figure S35. 600 MHz ^{13}C-NMR of Compound (**A9**) in Chloroform-*d*.

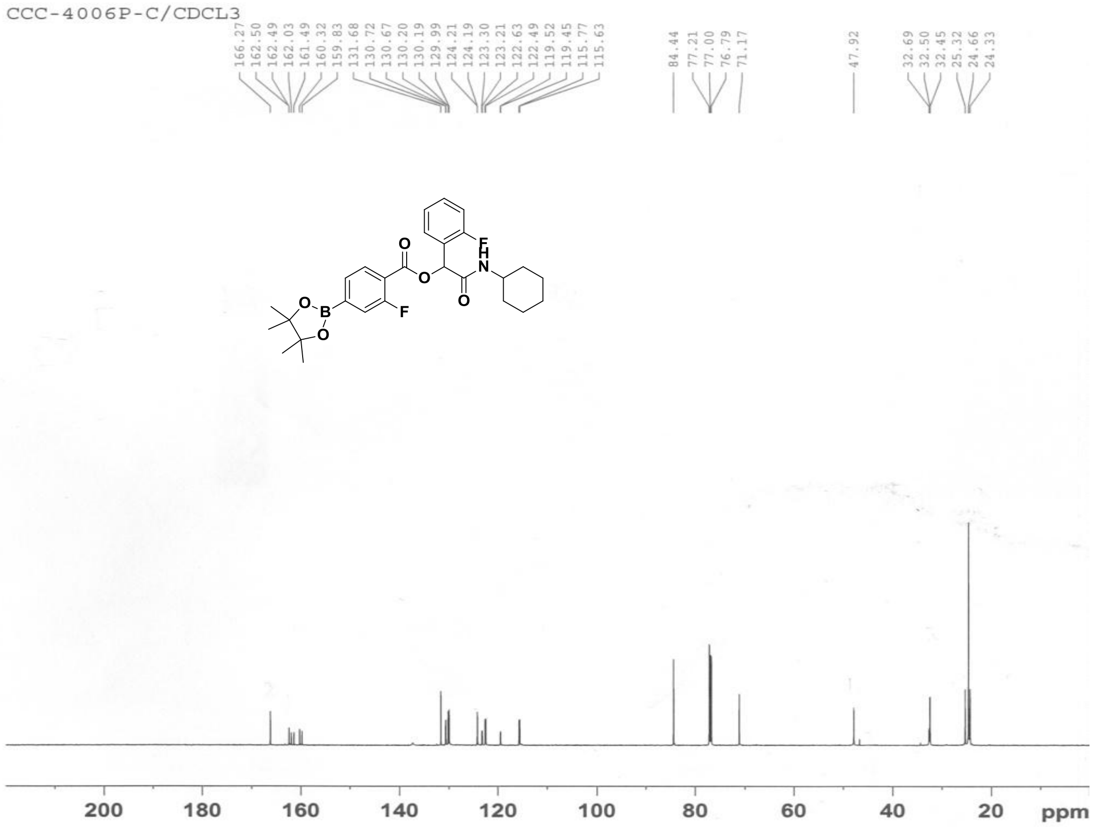

Figure S36. 600 MHz ^{11}B-NMR of Compound (**A9**) in Chloroform-*d*.

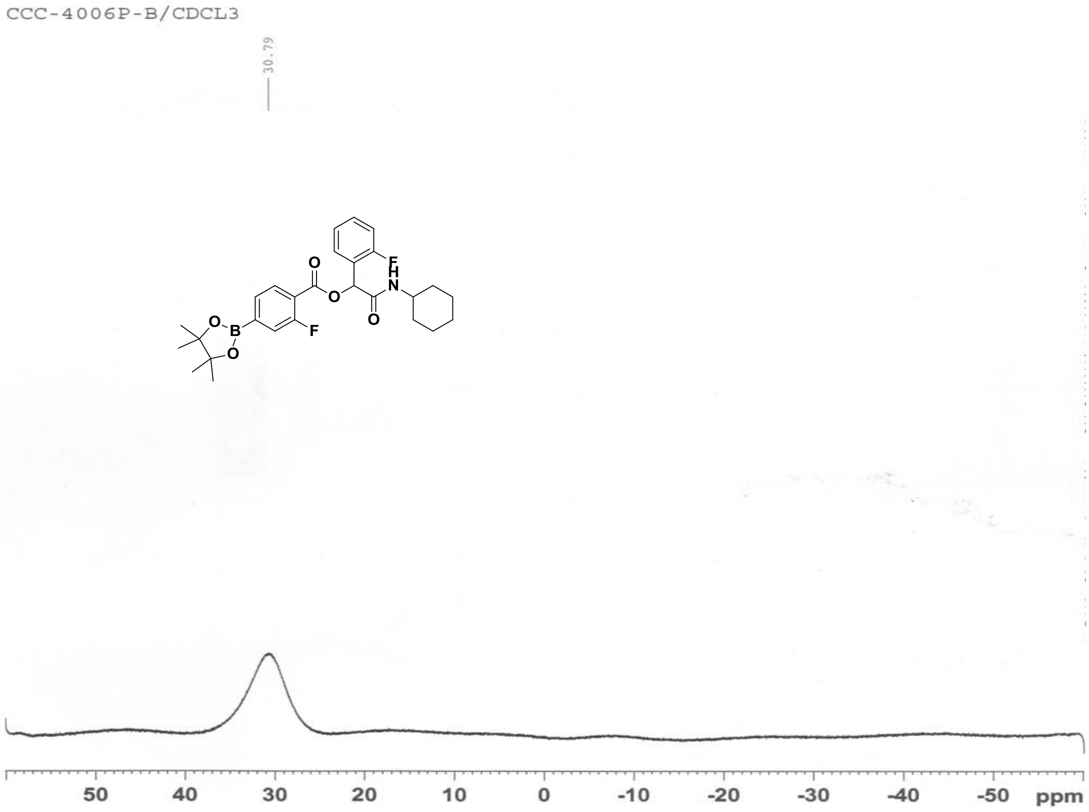

Figure S37. HRMS (ESI, positive ion) $[M+H]^+$ of Compound (**A10**).

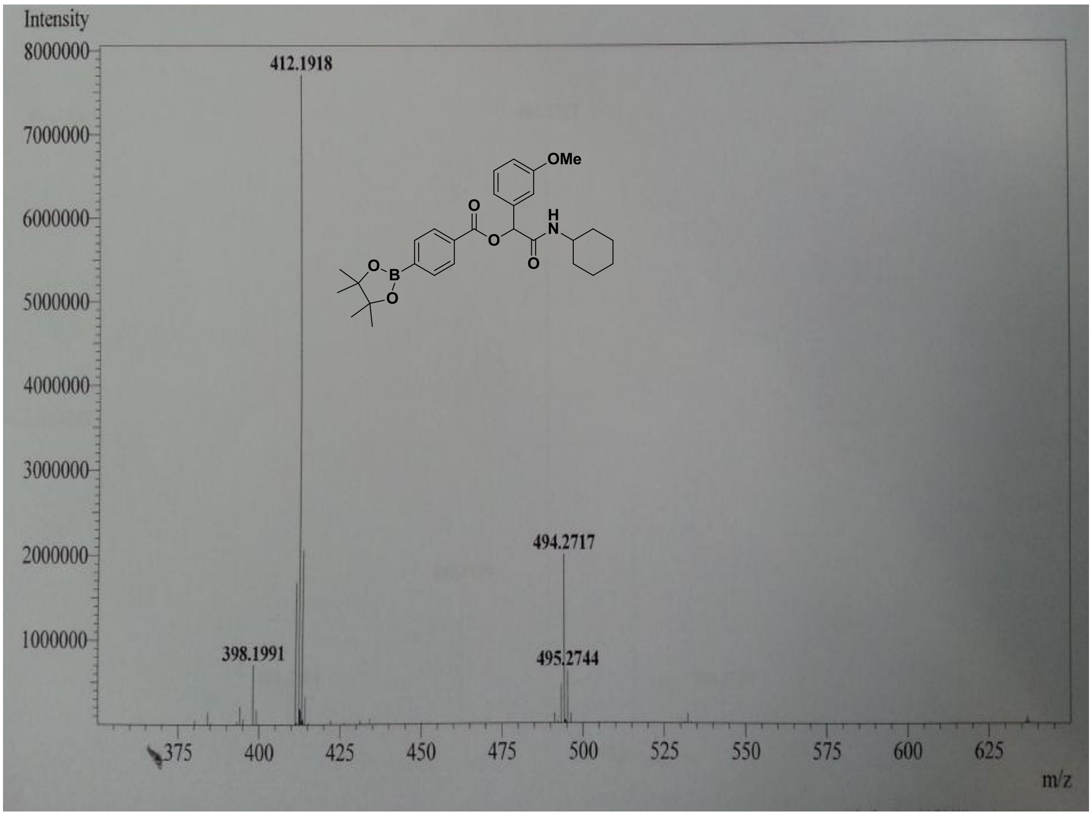

Figure S38. 600 MHz ^{1}H-NMR of Compound (**A10**) in Chloroform-*d*.

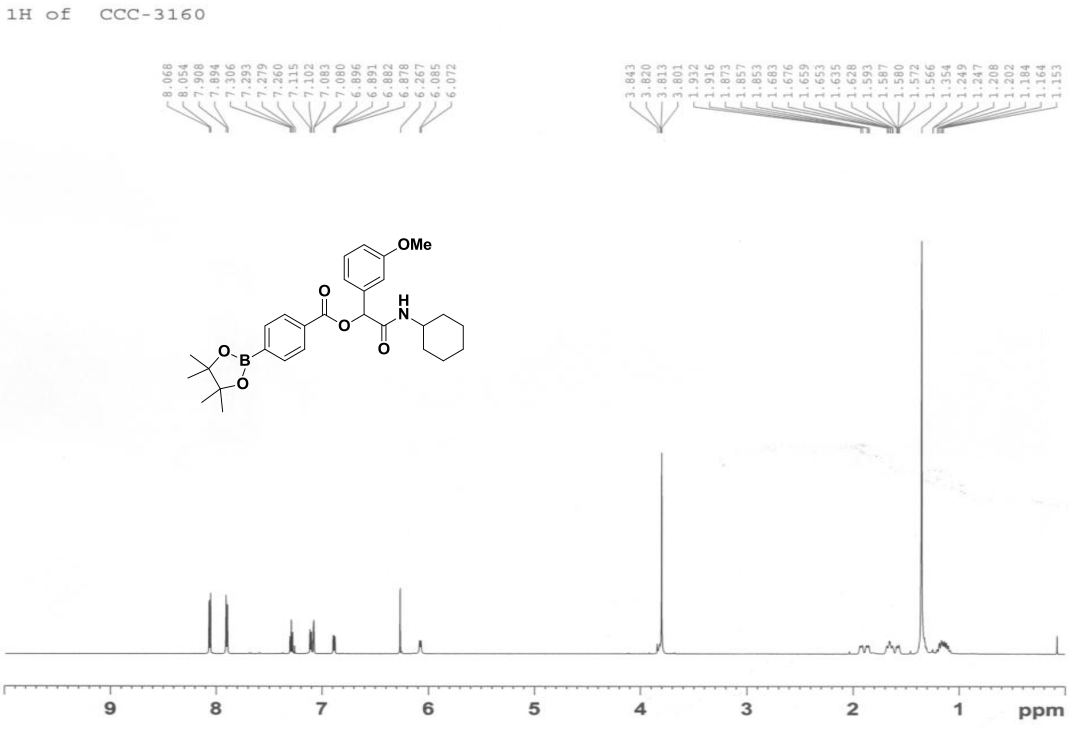

Figure S39. 600 MHz ^{13}C-NMR of Compound (**A10**) in Chloroform-*d*.

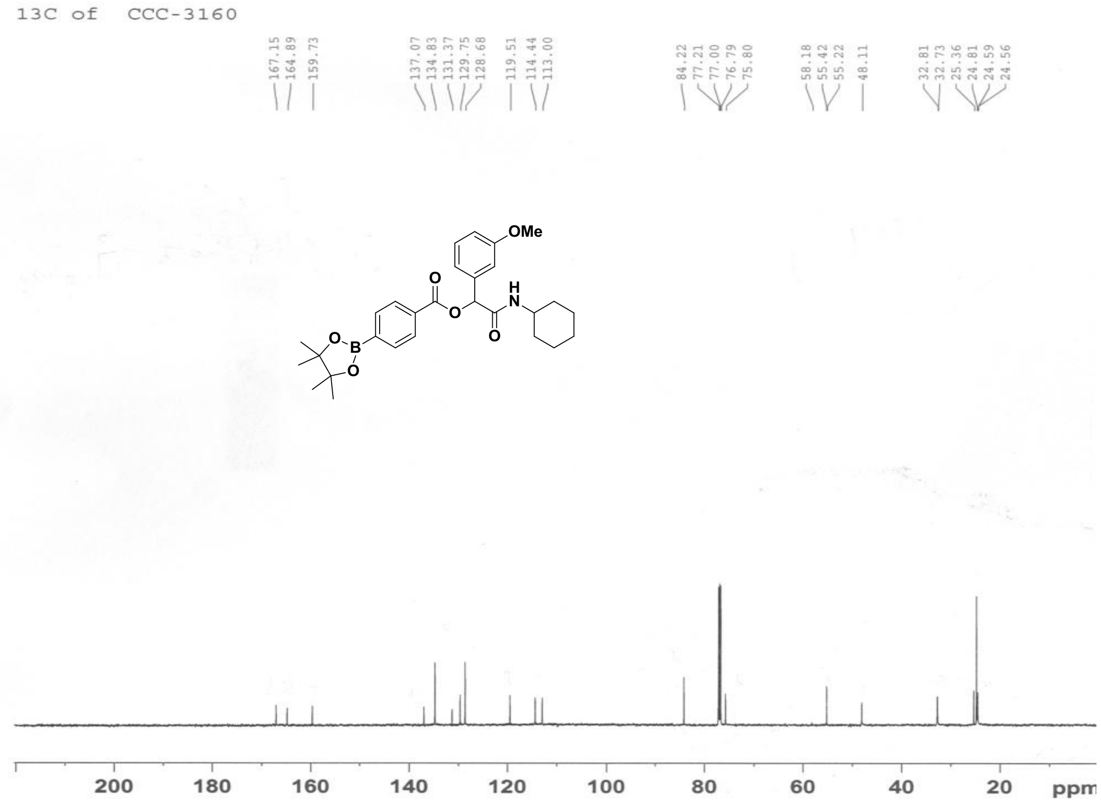

Figure S40. 600 MHz ^{11}B-NMR of Compound (**A10**) in Chloroform-*d*.

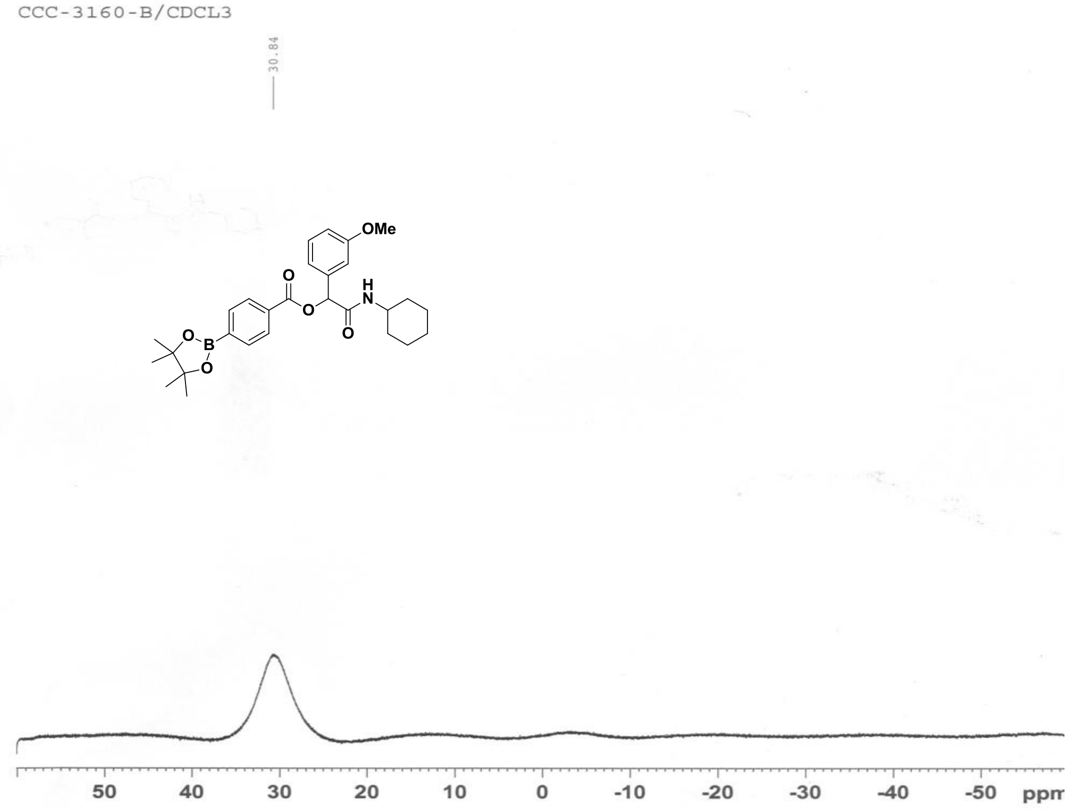

Figure S41. HRMS (ESI, positive ion) [M+H]+ of Compound (**A11**).

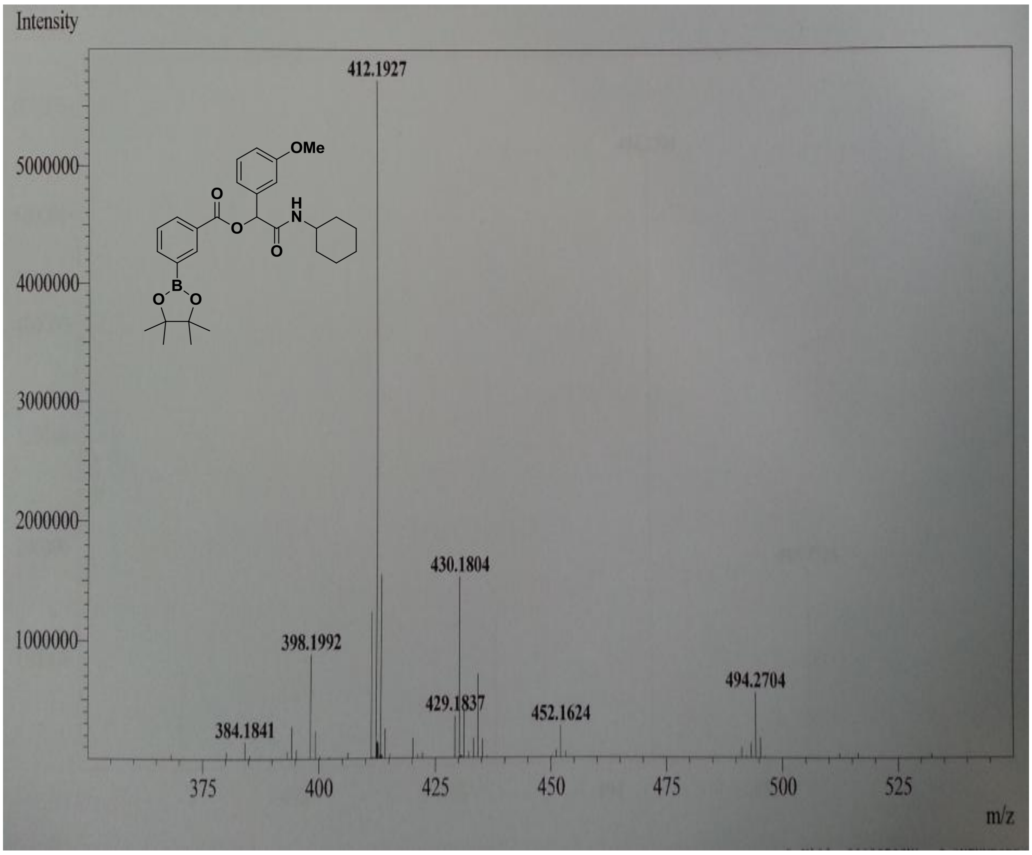

Figure S42. 600 MHz ^{1}H-NMR of Compound (**A11**) in Chloroform-*d*.

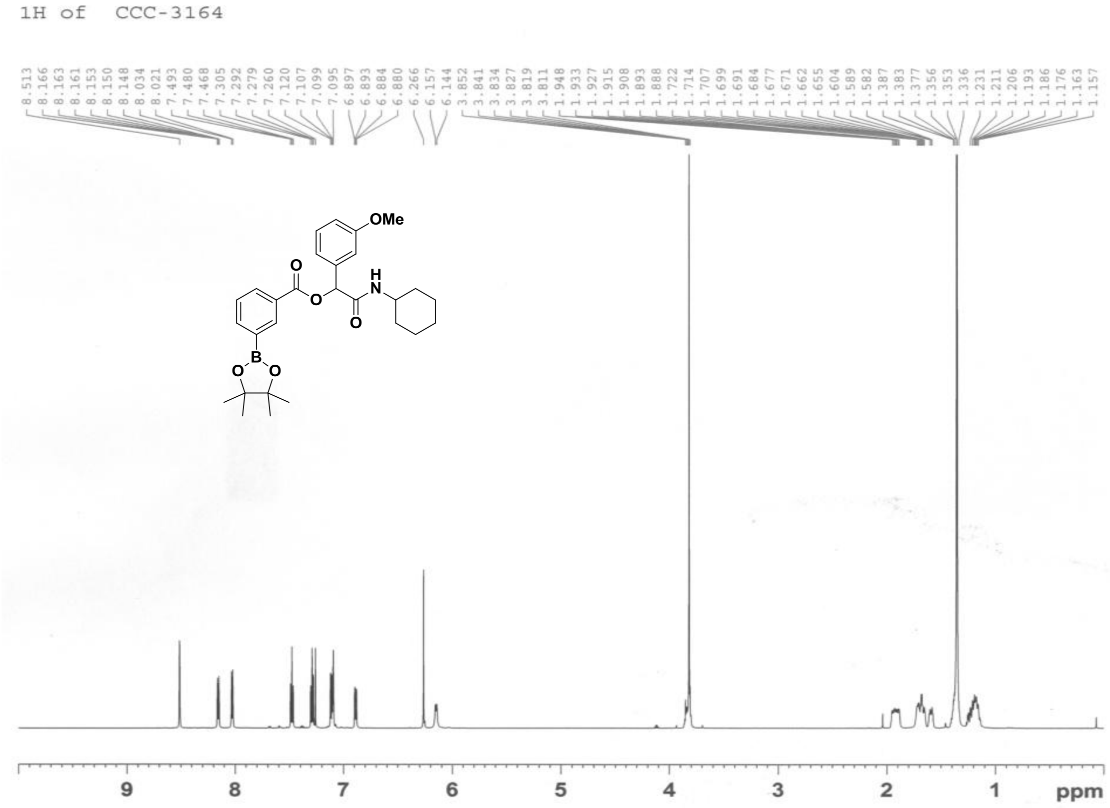

Figure S43. 600 MHz ^{13}C-NMR of Compound (**A11**) in Chloroform-*d*.

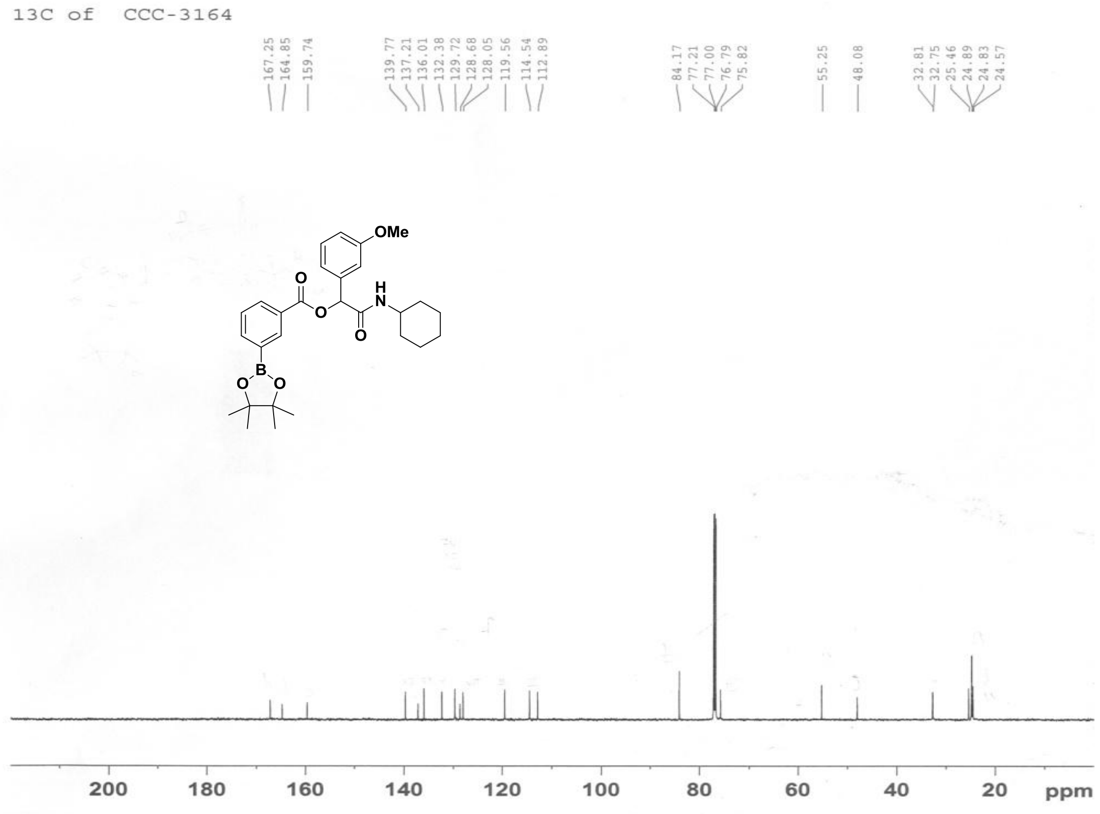

Figure S44. 600 MHz ^{11}B-NMR of Compound (**A11**) in Chloroform-*d*.

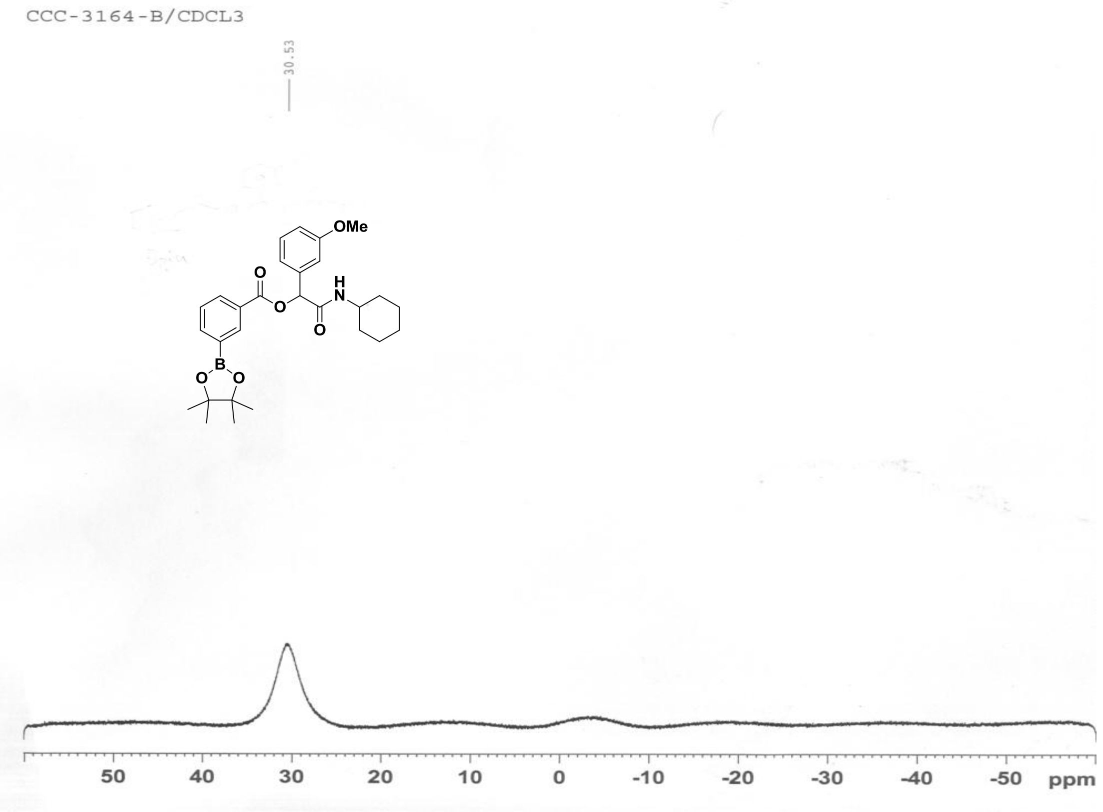

Figure S45. HRMS (ESI, positive ion) $[M+H]^+$ of Compound (**A12**).

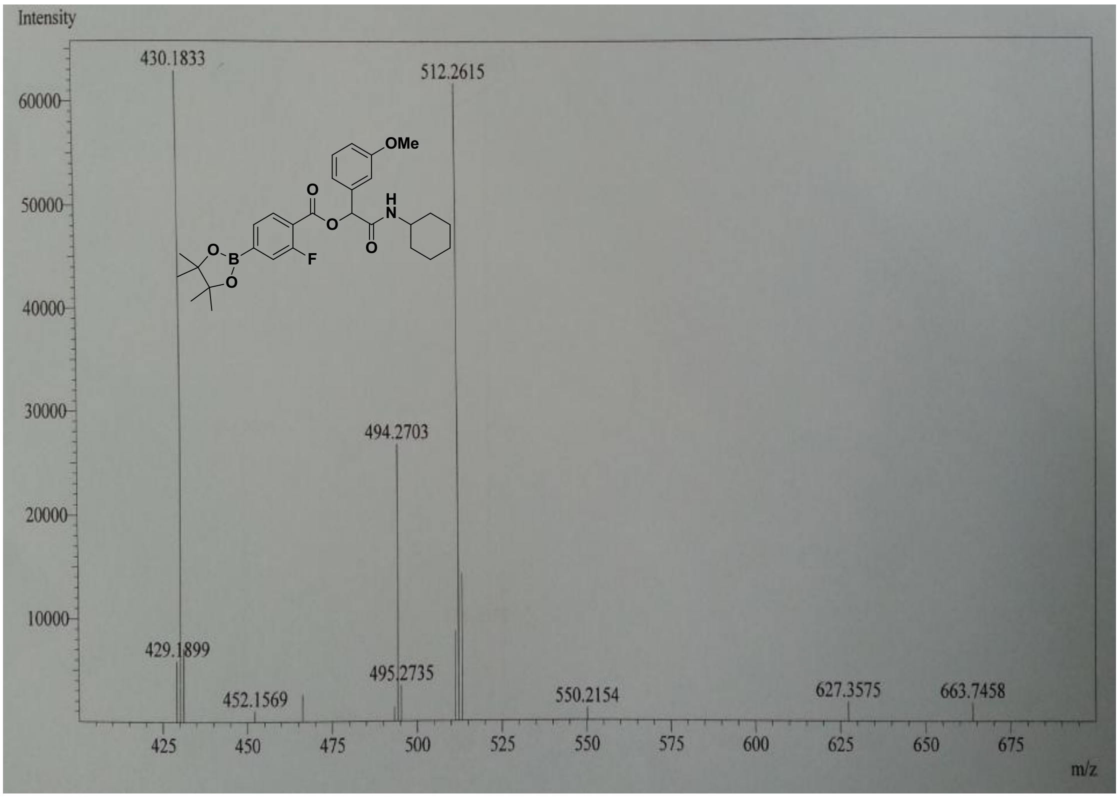

Figure S46. 600 MHz ^{1}H-NMR of Compound (**A12**) in Chloroform-*d*.

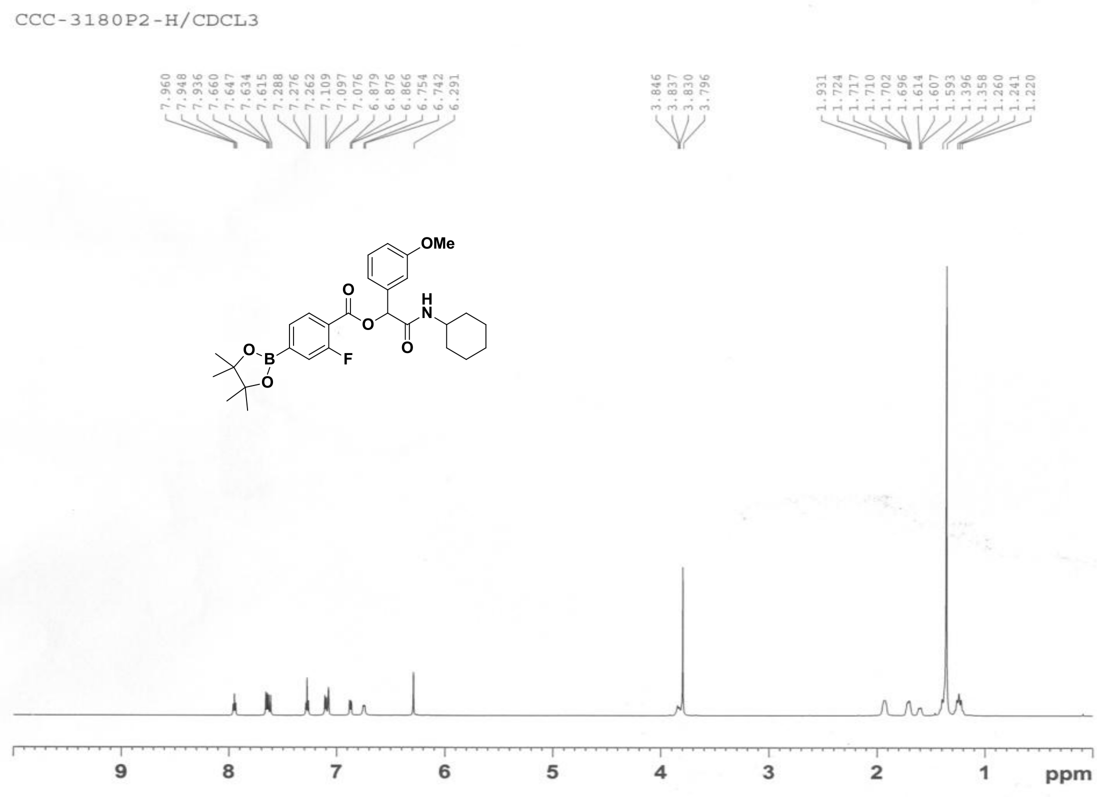

Figure S47. 600 MHz ^{13}C-NMR of Compound (**A12**) in Chloroform-*d*.

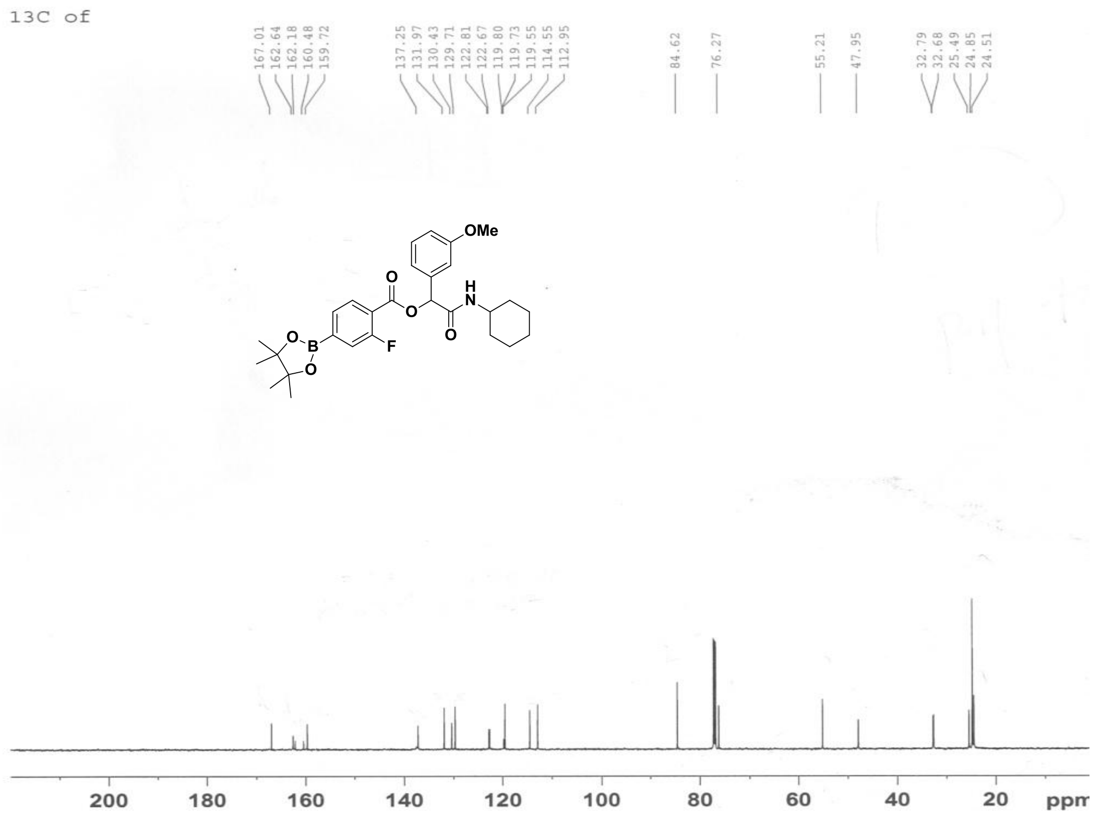

Figure S48. 600 MHz ^{11}B-NMR of Compound (**A12**) in Chloroform-*d*.

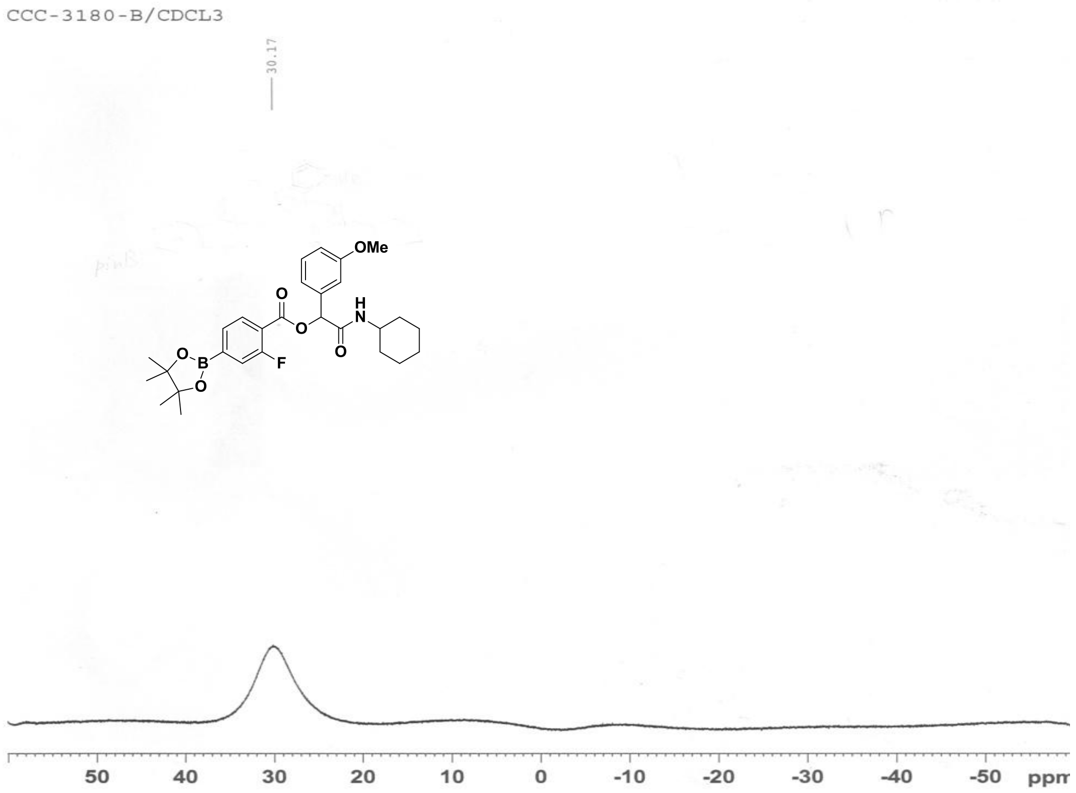

Figure S49. HRMS (ESI, positive ion) $[M+H]^+$ of Compound (**A13**).

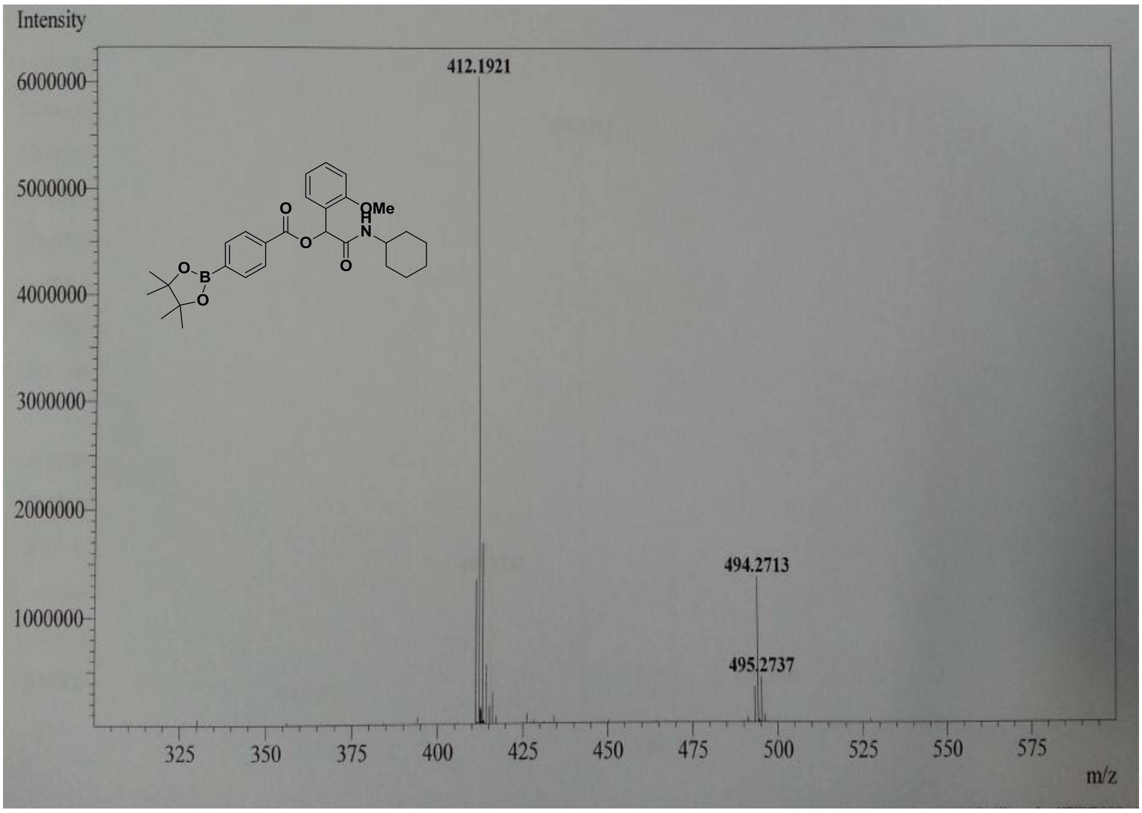

Figure S50. 600 MHz ^{1}H-NMR of Compound (**A13**) in Chloroform-*d*.

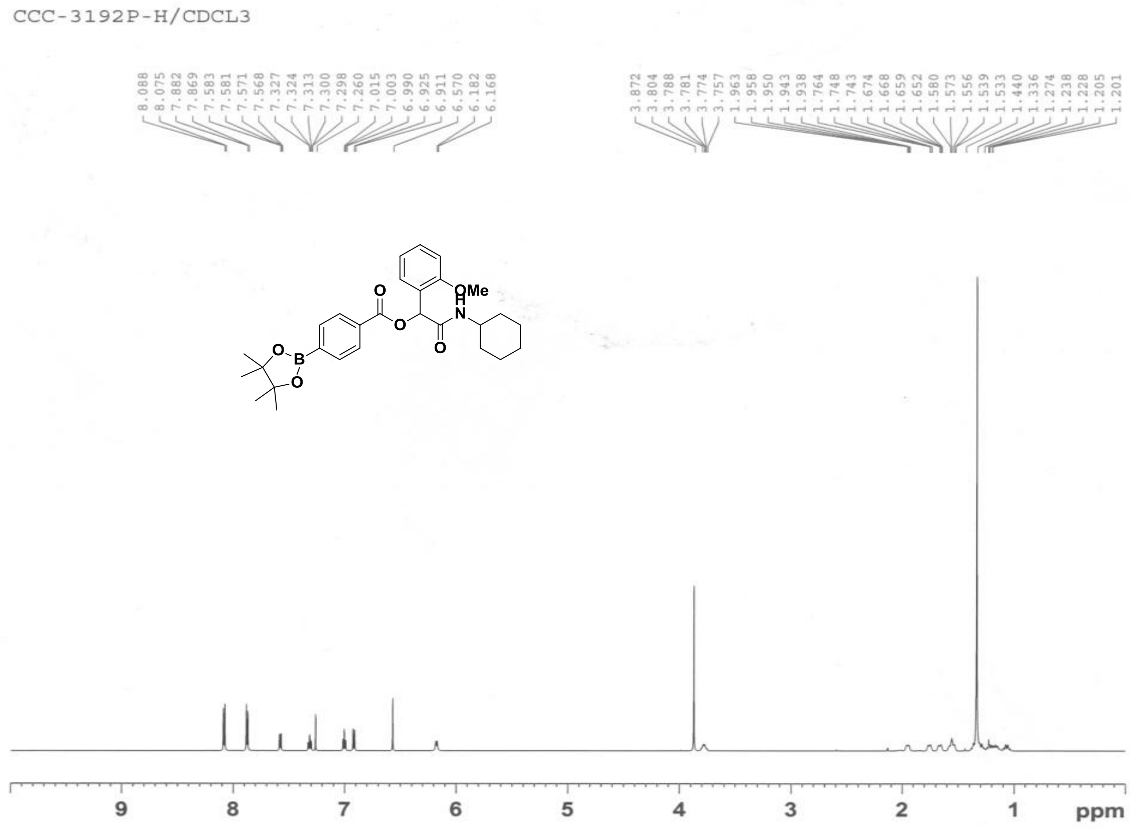

Figure S51. 600 MHz ^{13}C-NMR of Compound (**A13**) in Chloroform-*d*.

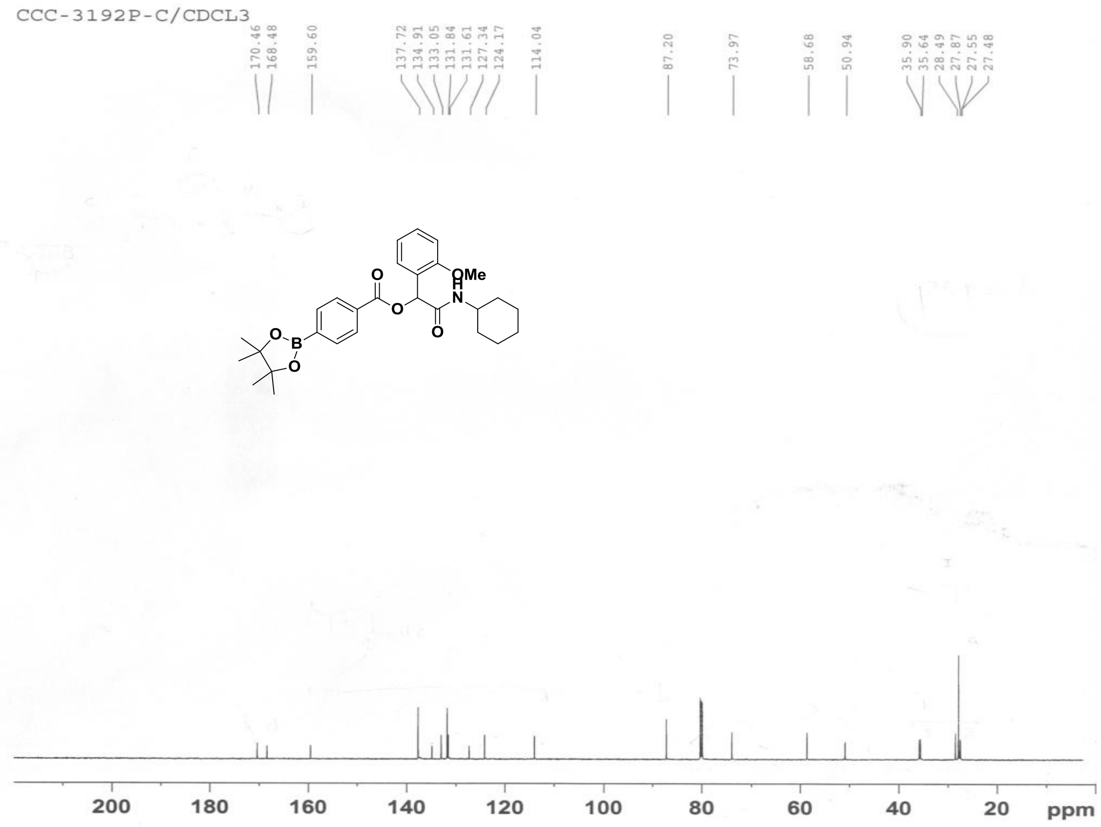

Figure S52. 600 MHz ^{11}B-NMR of Compound (**A13**) in Chloroform-*d*.

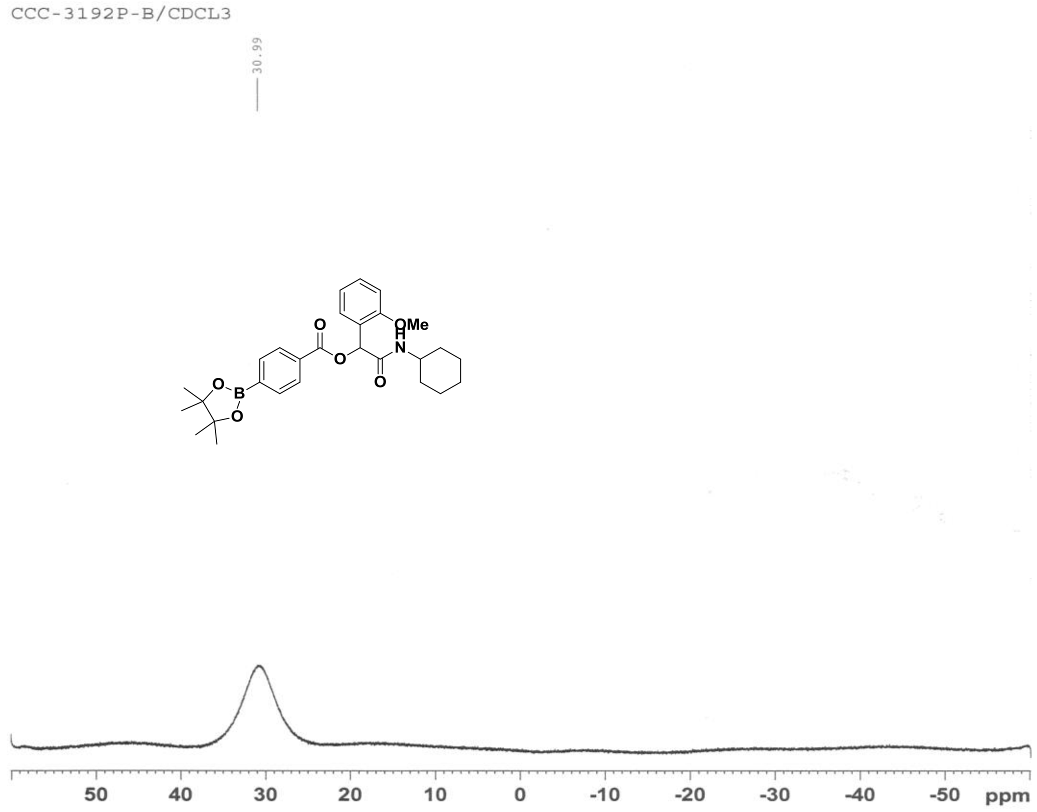

Figure S53. HRMS (ESI, positive ion) $[M+H]^+$ of Compound (**A14**).

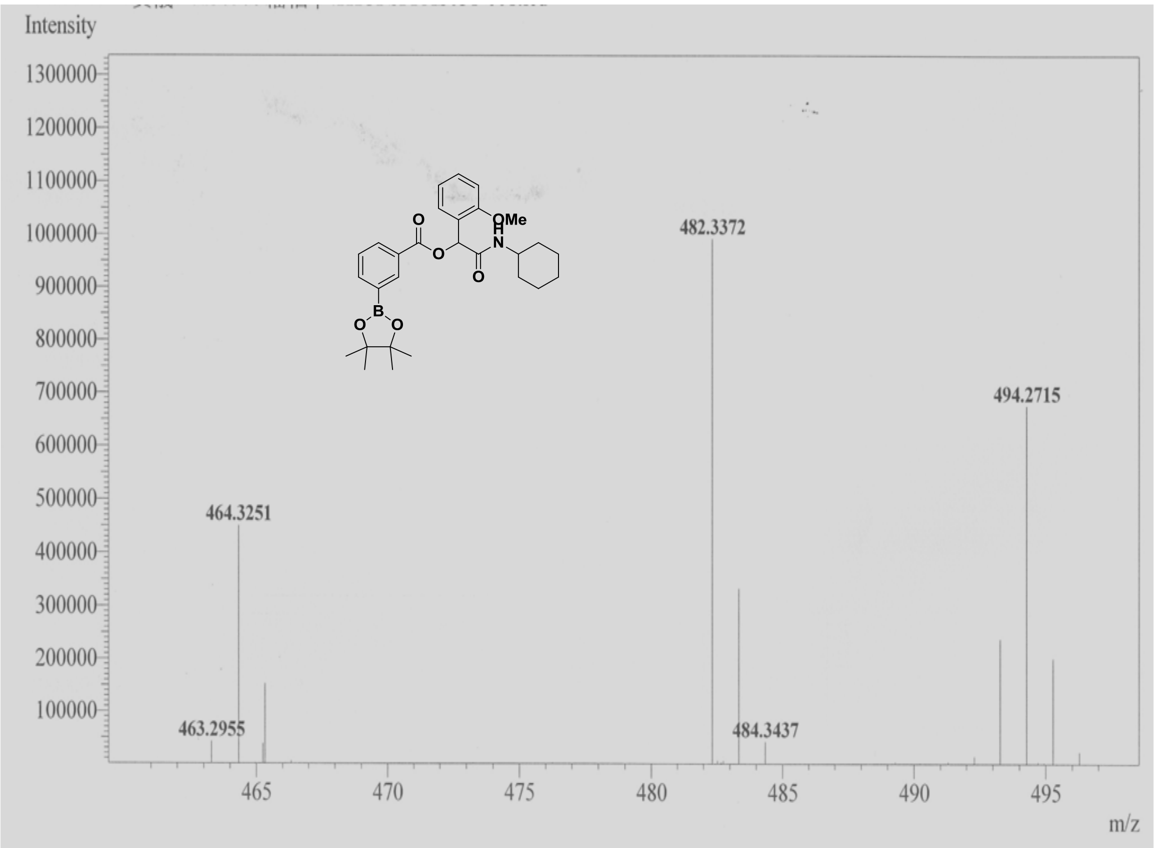

Figure S54. 600 MHz ^{1}H-NMR of Compound (**A14**) in Chloroform-*d*.

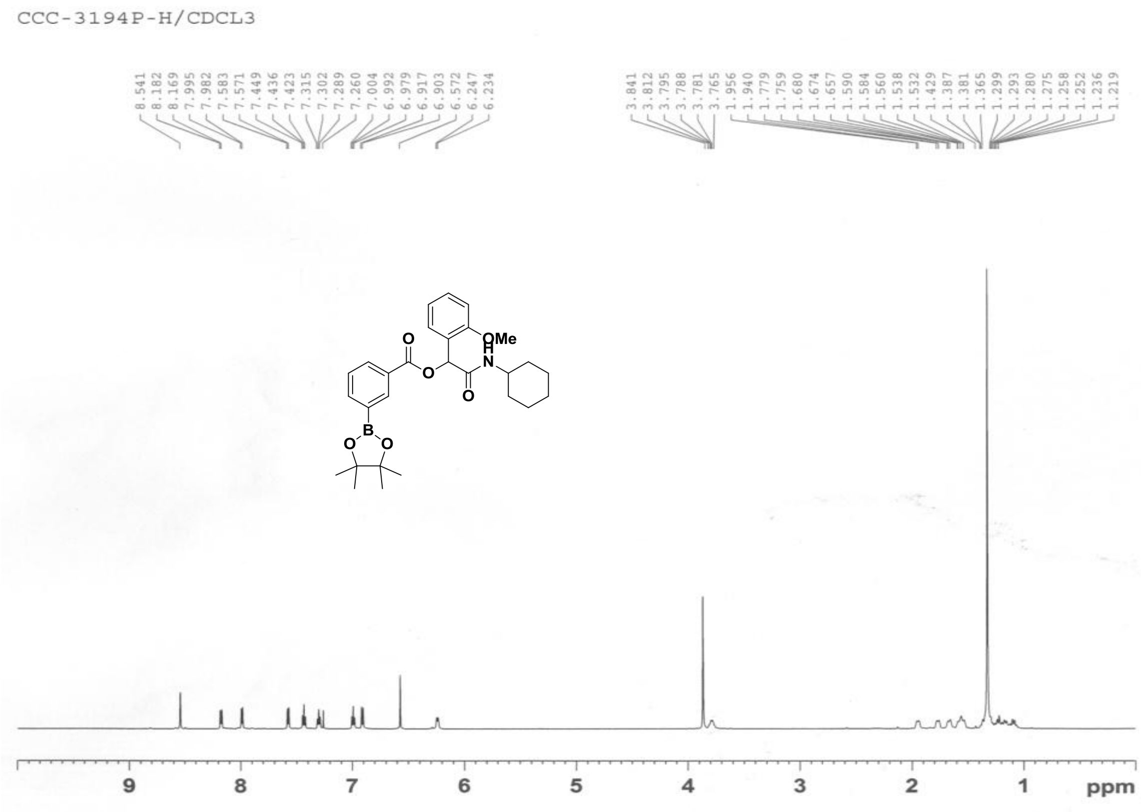

Figure S55. 600 MHz ^{13}C-NMR of Compound (**A14**) in Chloroform-*d*.

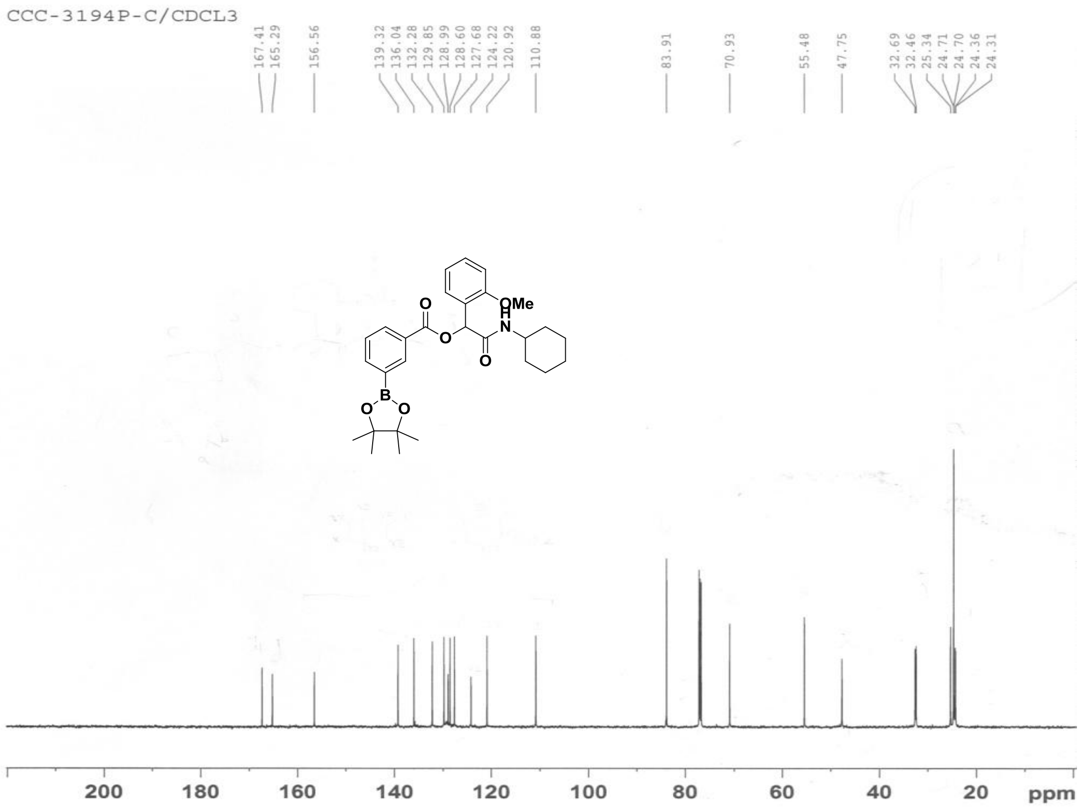

Figure S56. 600 MHz ^{11}B-NMR of Compound (**A14**) in Chloroform-*d*.

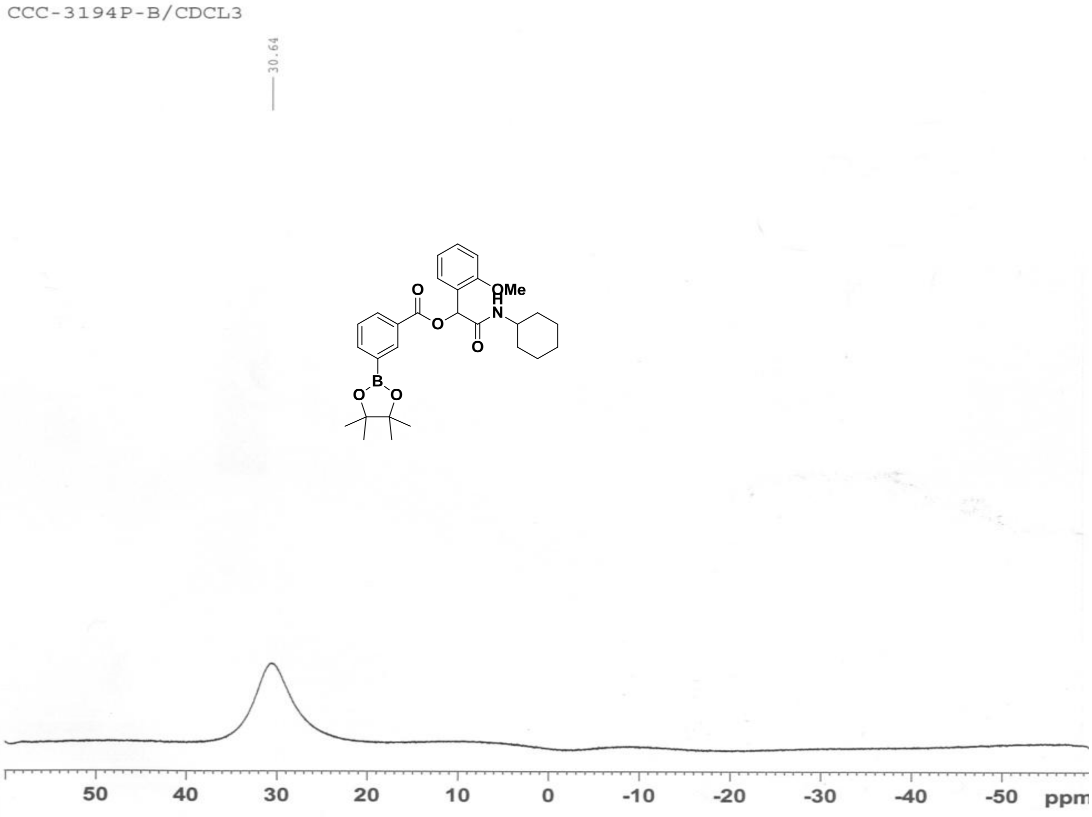

Figure S57. HRMS (ESI, positive ion) $[M+H]^+$ of Compound (**A15**).

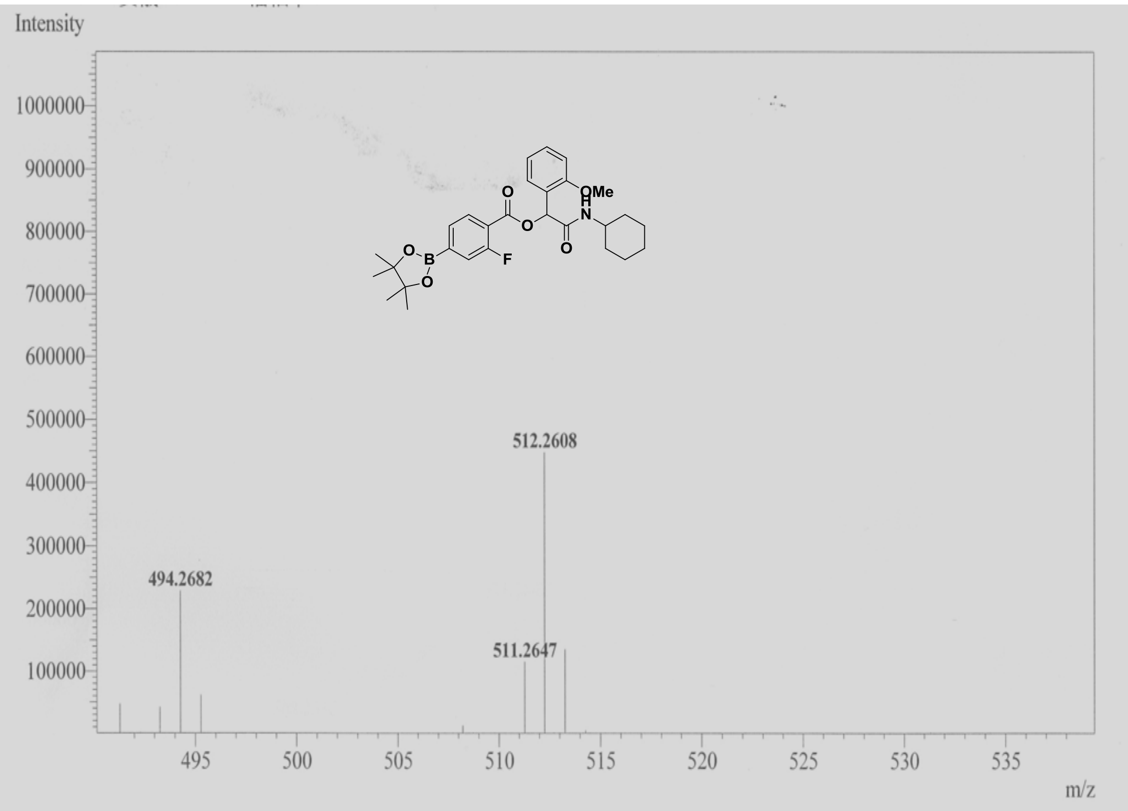

Figure S58. 600 MHz ^{1}H-NMR of Compound (**A15**) in Chloroform-*d*.

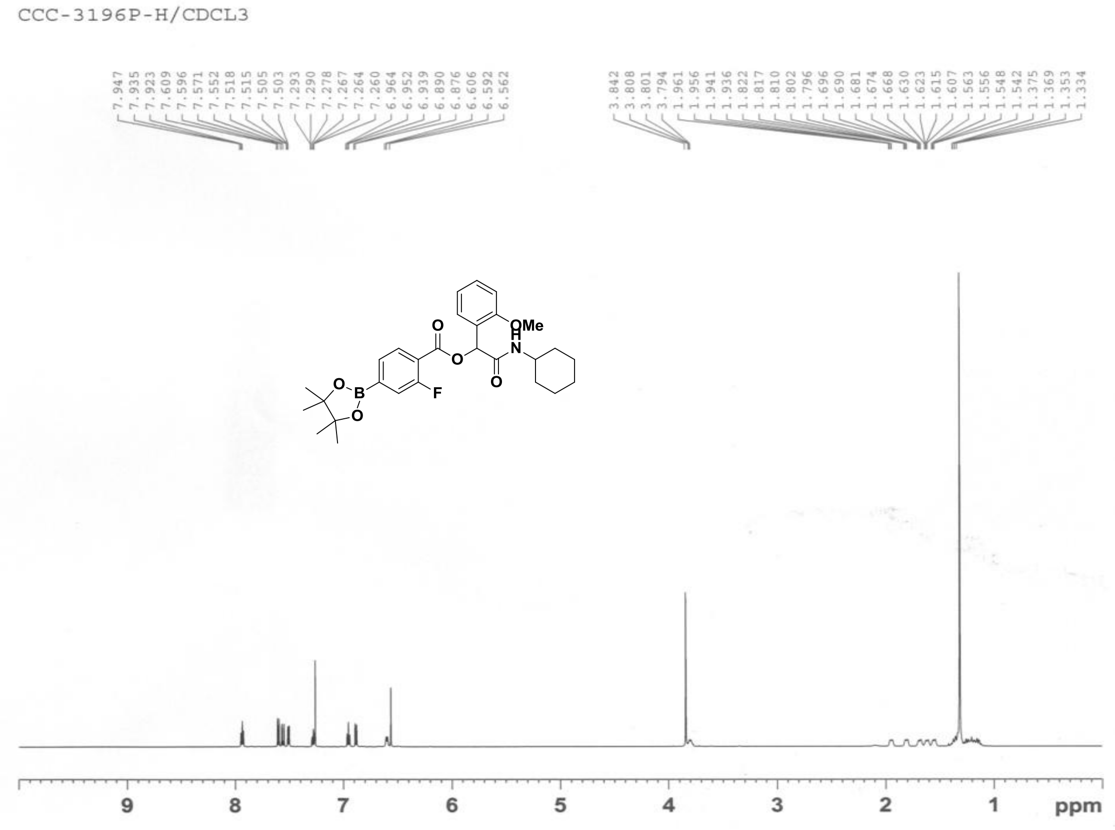

Figure S59. 600 MHz ^{13}C-NMR of Compound (**A15**) in Chloroform-*d*.

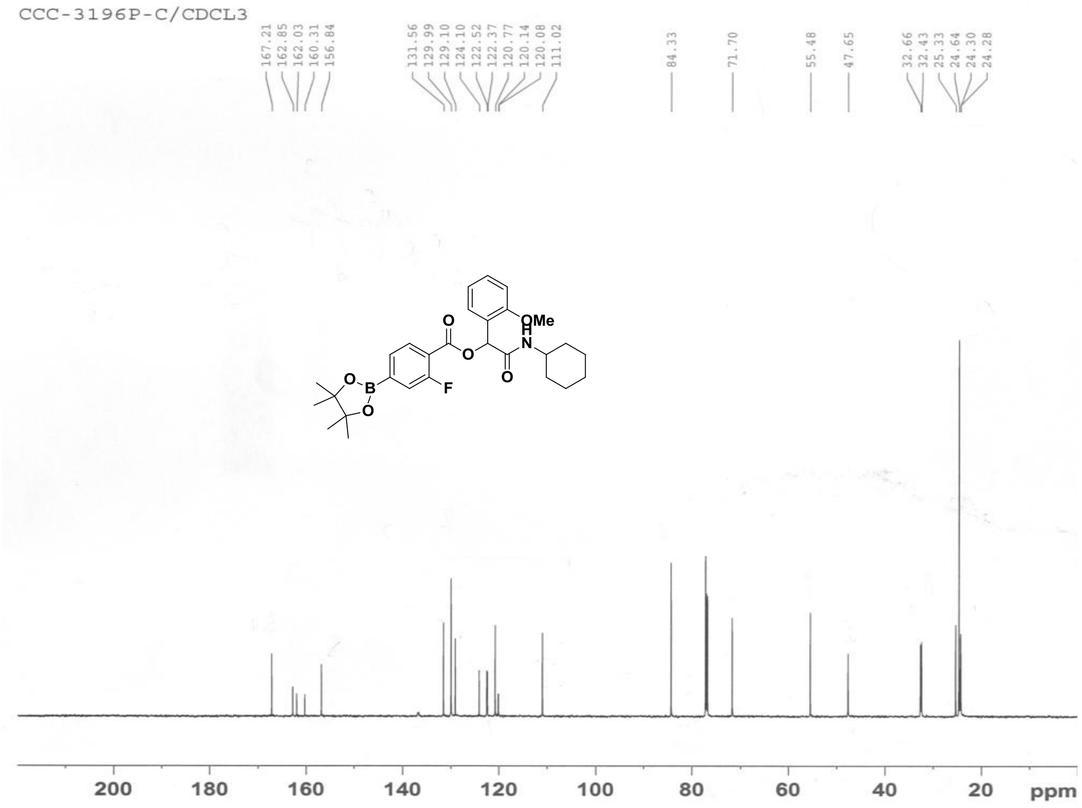

Figure S60. 600 MHz ^{11}B-NMR of Compound (**A15**) in Chloroform-*d*.

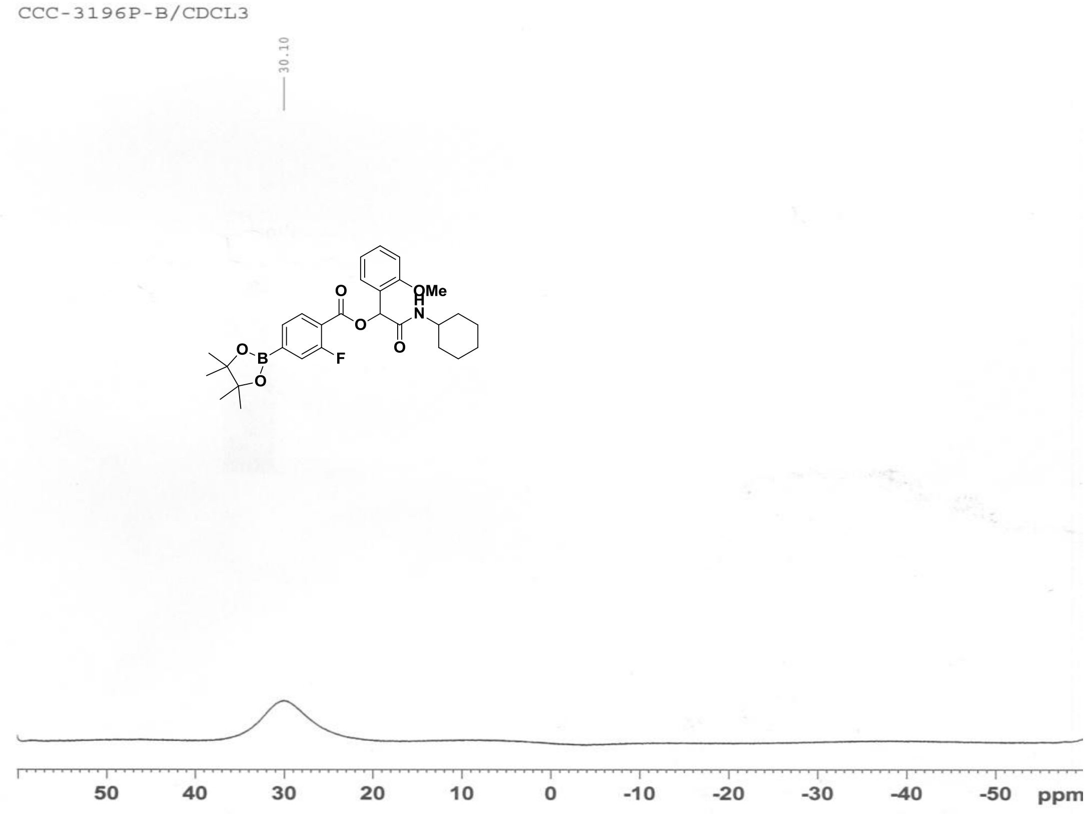

Figure S61. HRMS (ESI, positive ion) $[M+H]^+$ of Compound (**A16**).

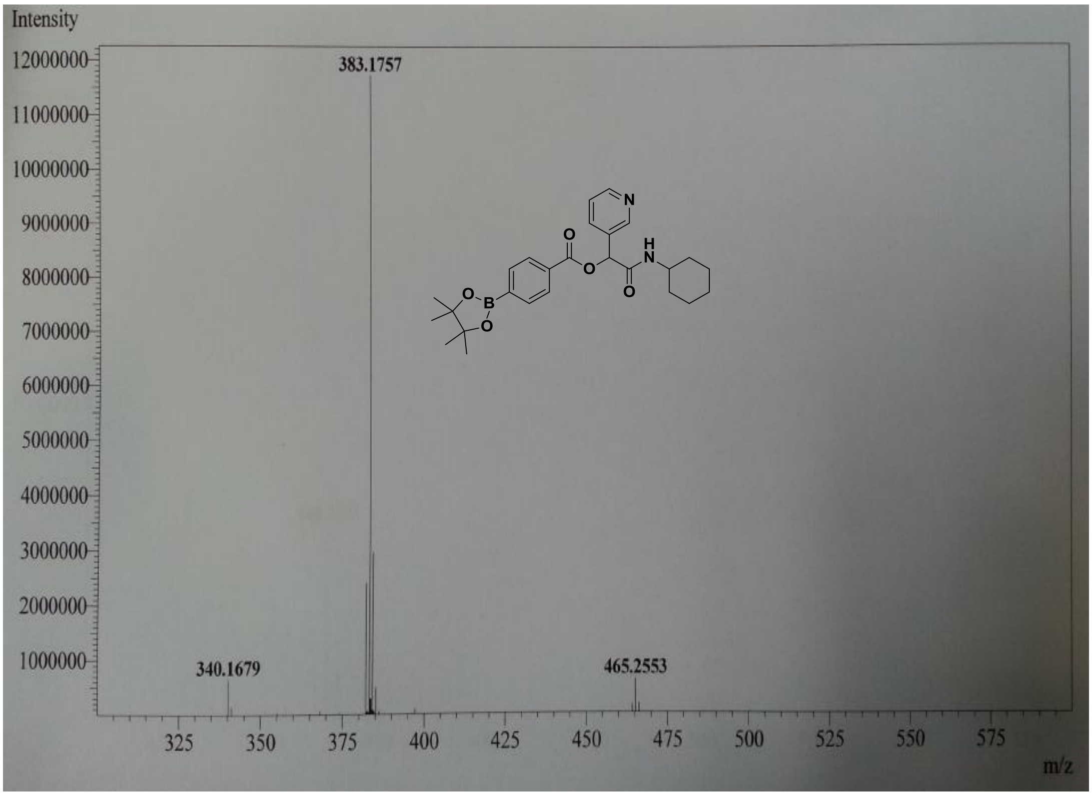

Figure S62. 600 MHz ^{1}H-NMR of Compound (**A16**) in Chloroform-*d*.

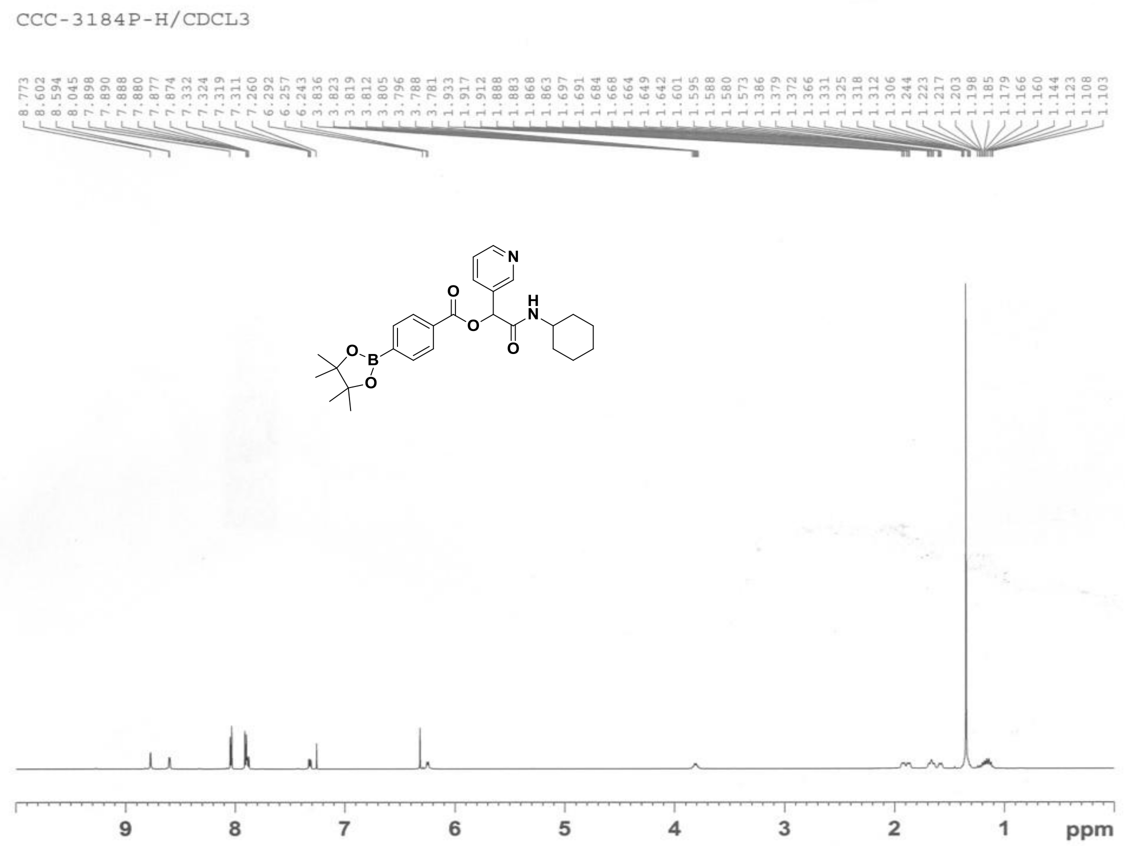

Figure S63. 600 MHz ^{13}C-NMR of Compound (**A16**) in Chloroform-*d*.

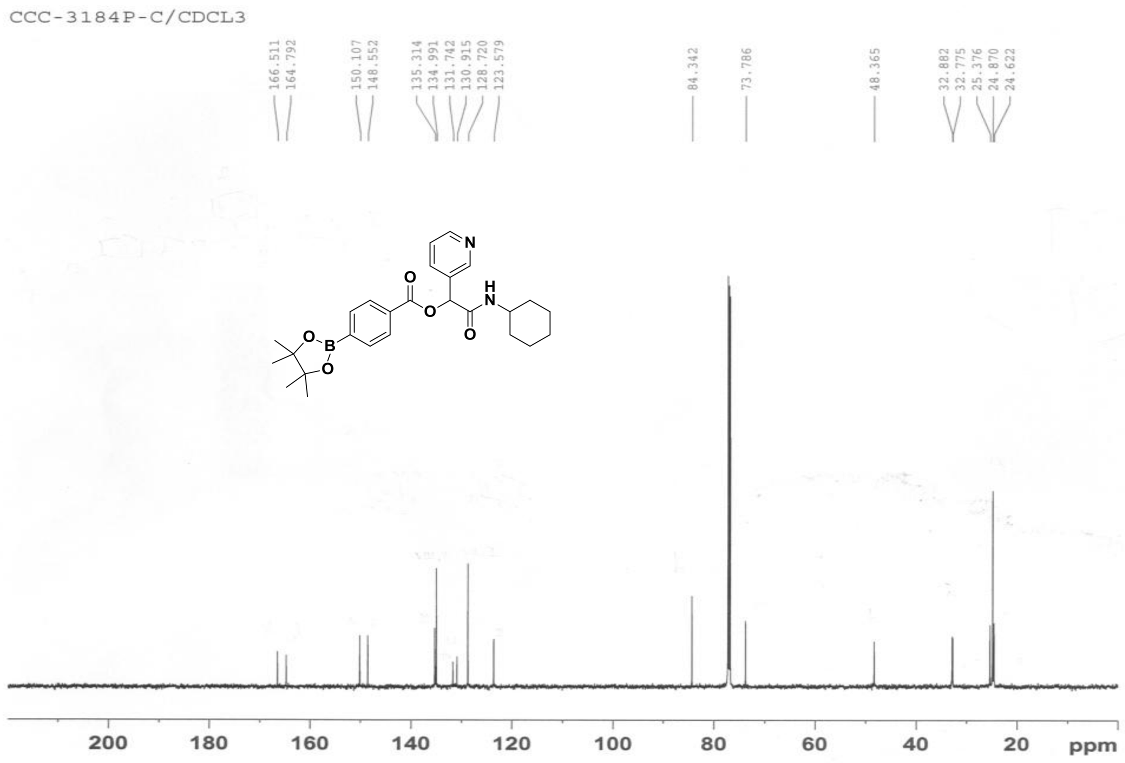

Figure S64. 600 MHz ^{11}B-NMR of Compound (**A16**) in Chloroform-*d*.

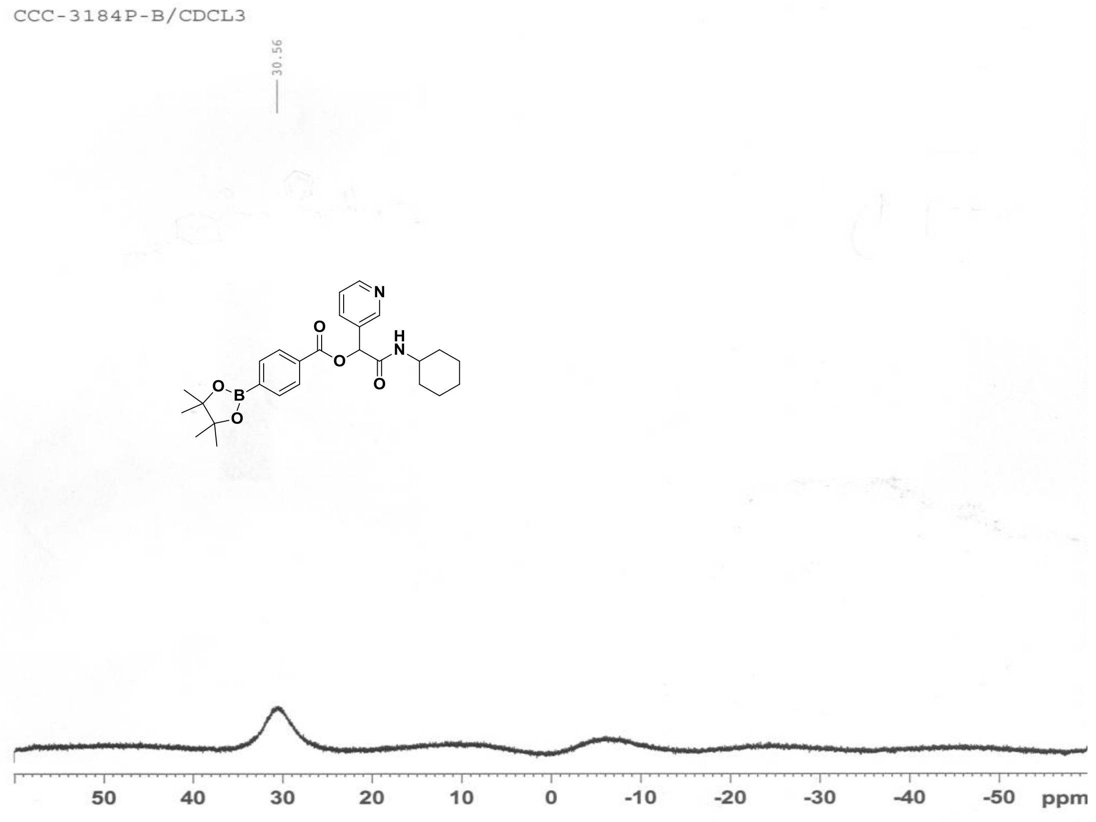

Figure S65. HRMS (ESI, positive ion) $[M+H]^+$ of Compound (**A17**).

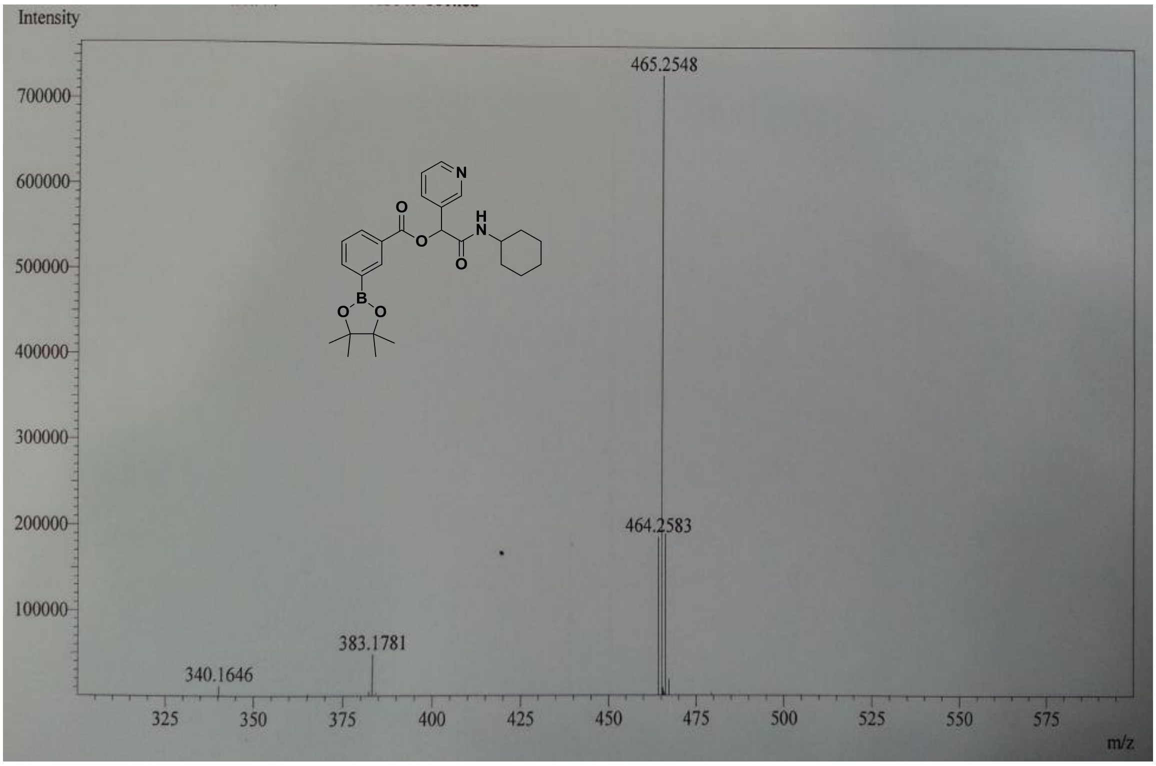

Figure S66. 600 MHz ^{1}H-NMR of Compound (**A17**) in Chloroform-*d*.

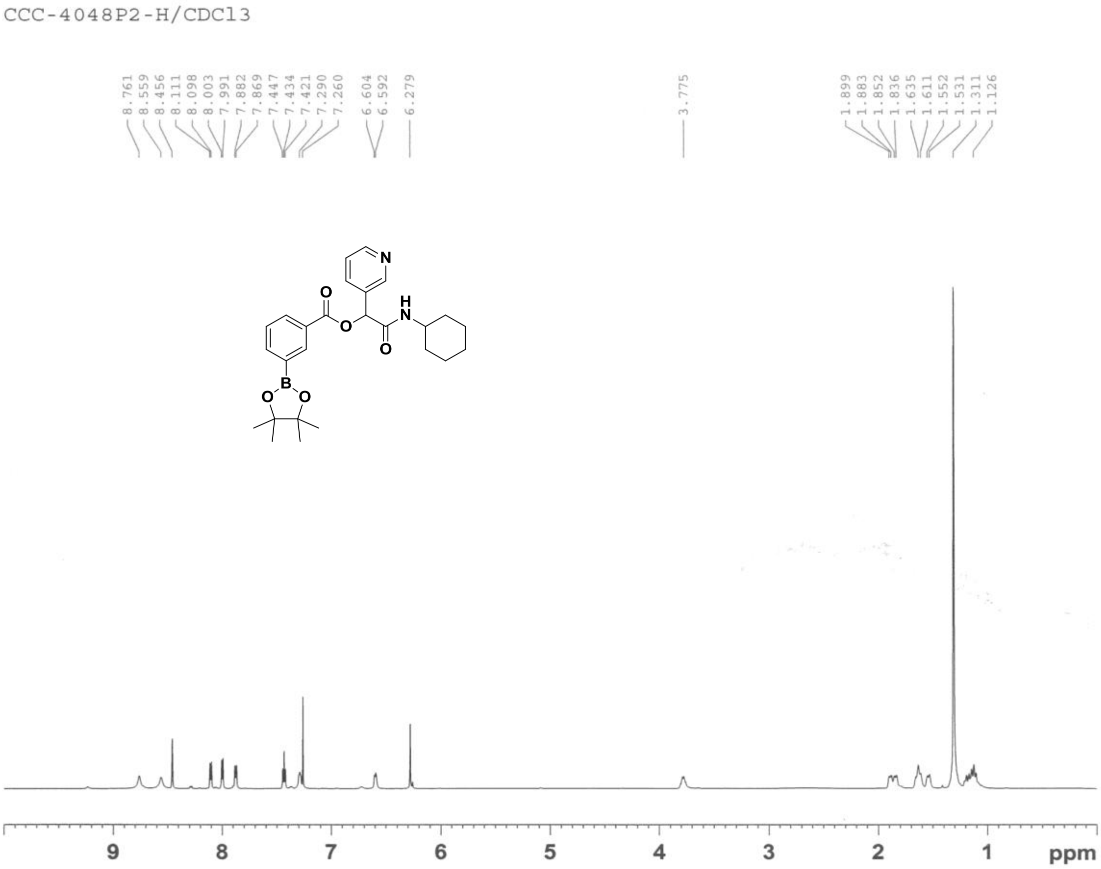

Figure S67. 600 MHz ^{13}C-NMR of Compound (**A17**) in Chloroform-*d*.

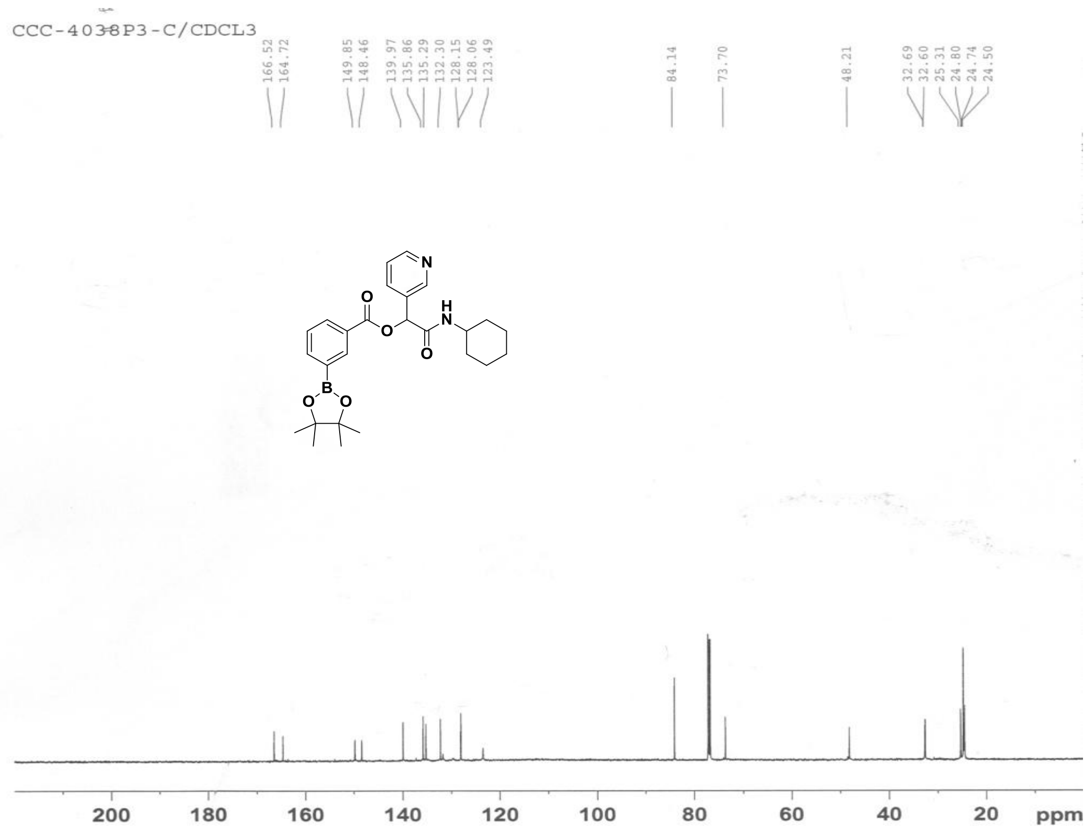

Figure S68. 600 MHz ^{11}B-NMR of Compound (**A17**) in Chloroform-*d*.

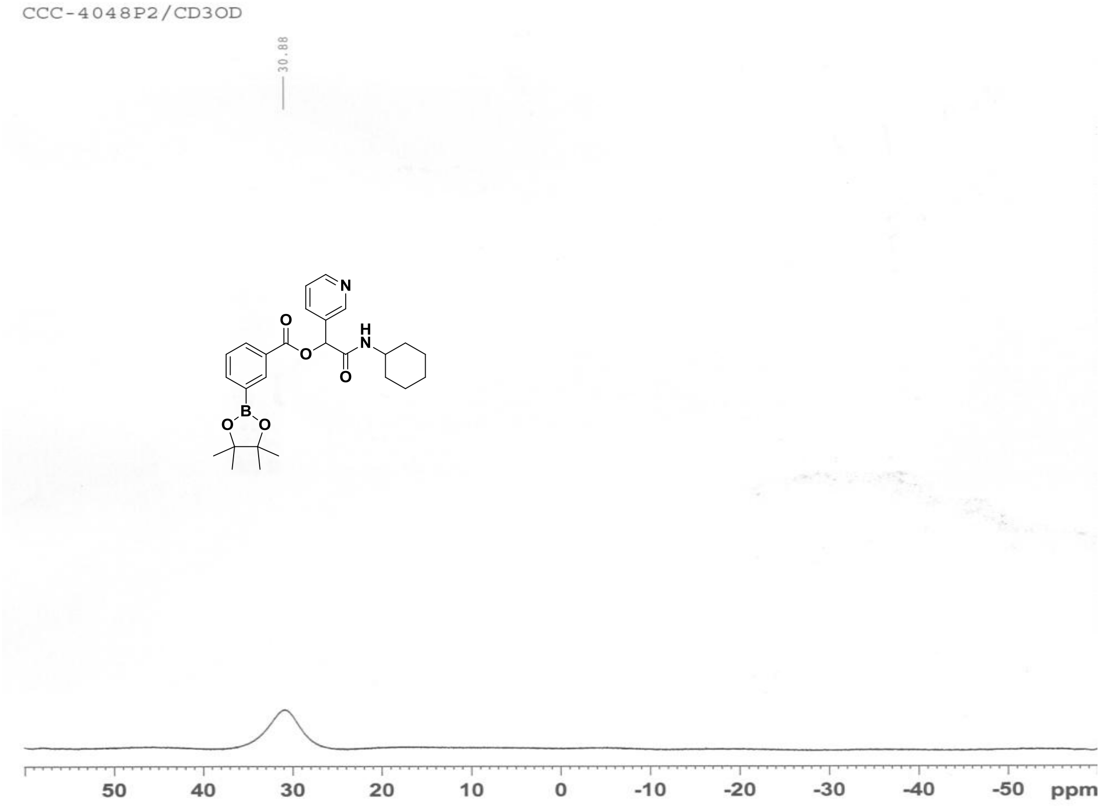

Figure S69. HRMS (ESI, positive ion) [M+H]$^+$ of Compound (**A18**).

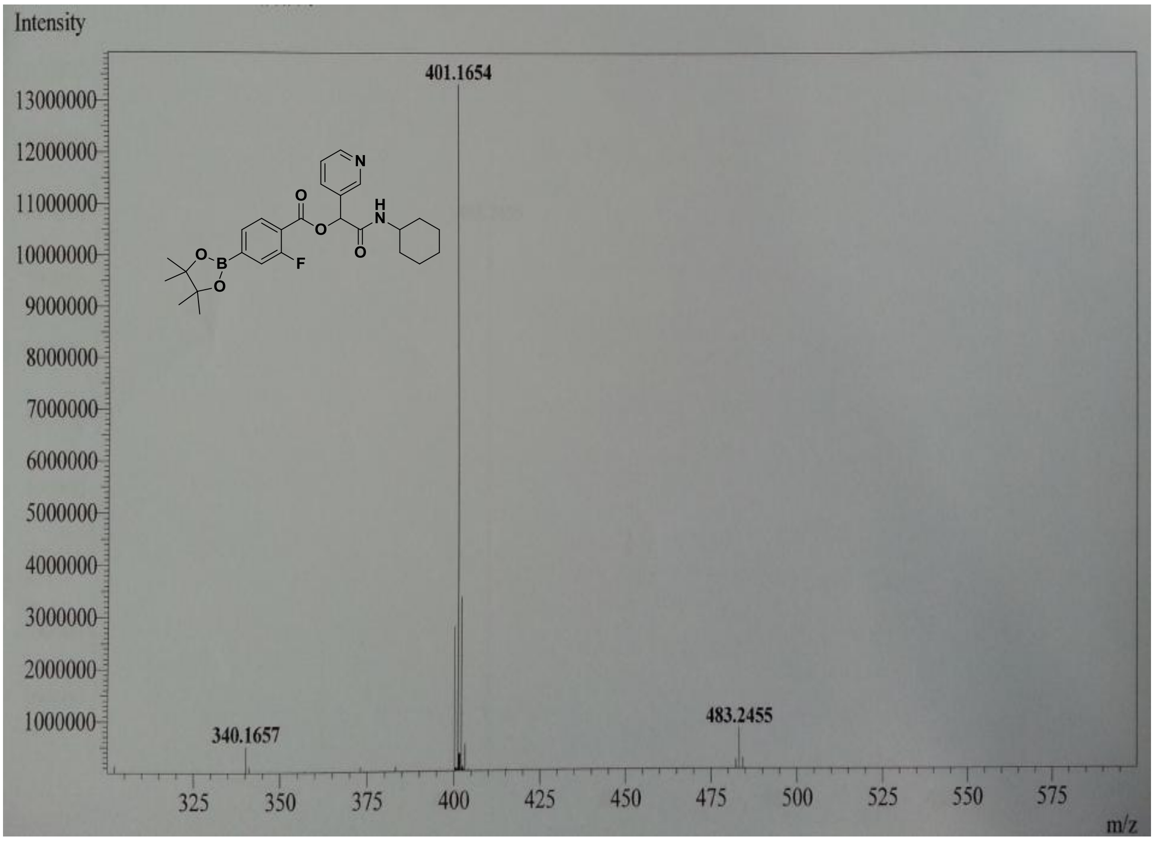

Figure S70. 600 MHz ^{1}H-NMR of Compound (**A18**) in Chloroform-*d*.

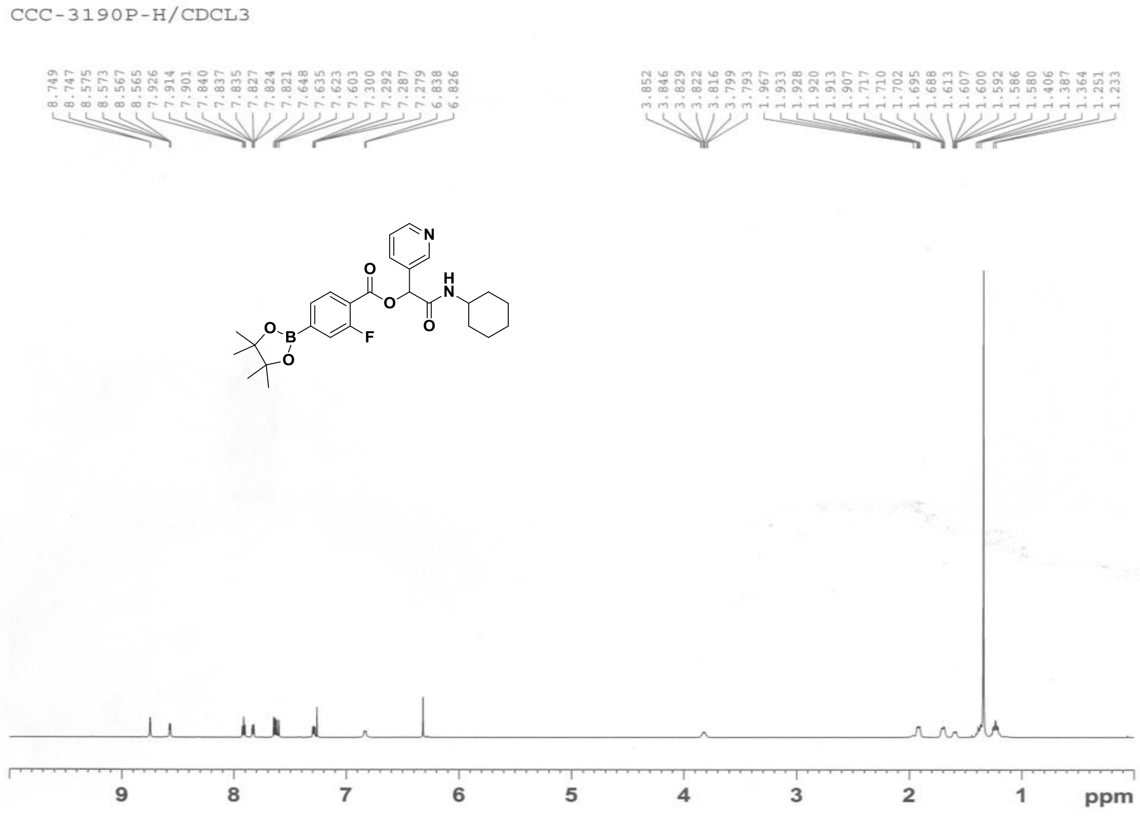

Figure S71. 600 MHz ^{13}C-NMR of Compound (**A18**) in Chloroform-*d*.

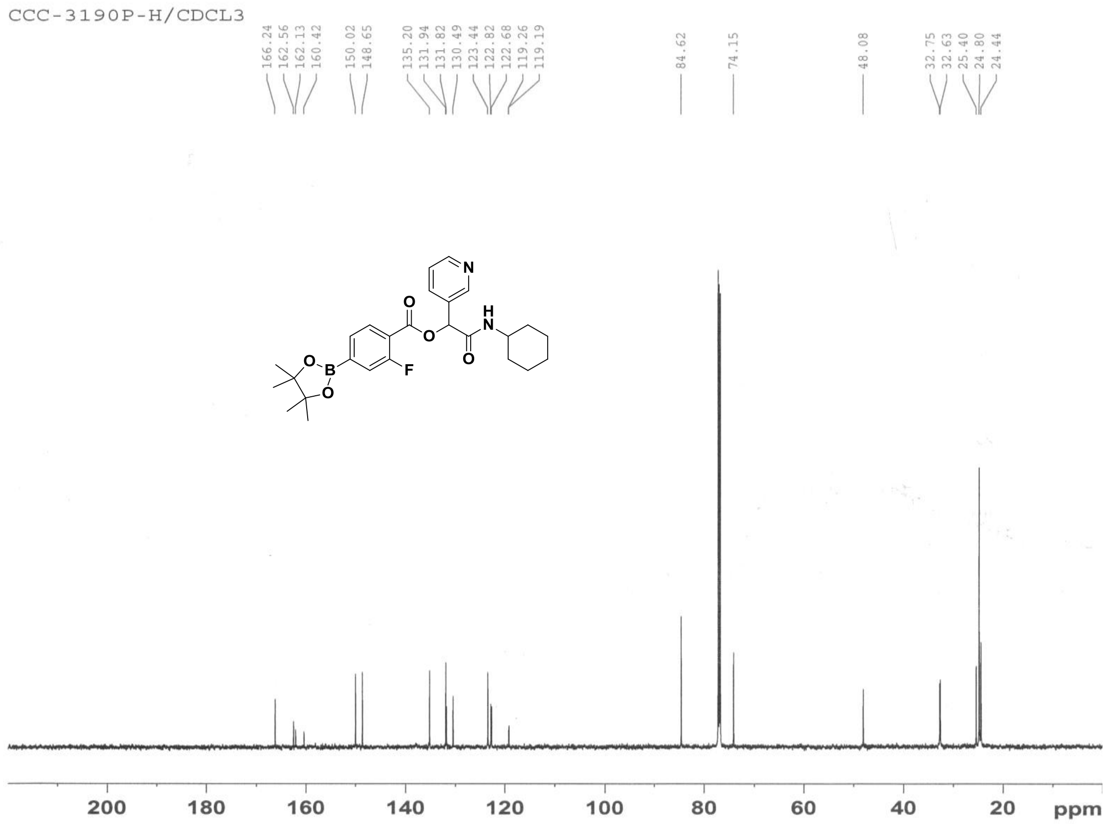

Figure S72. 600 MHz ^{11}B-NMR of Compound (**A18**) in Chloroform-*d*.

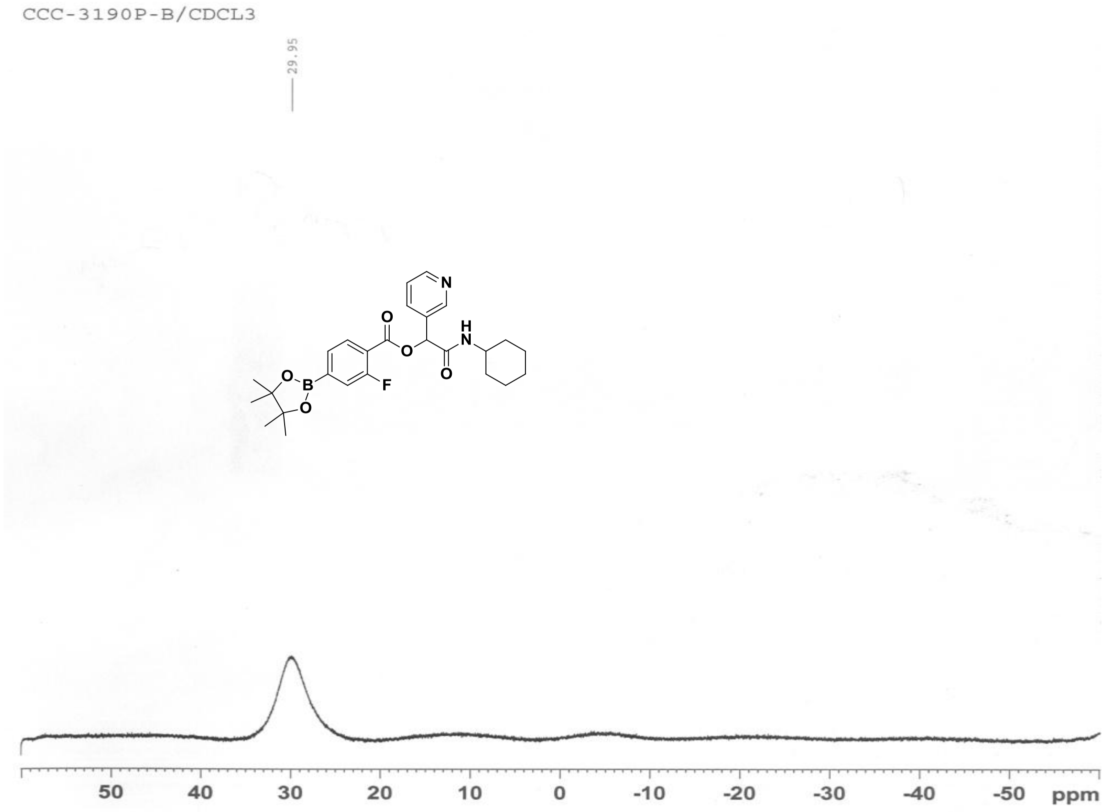

Figure S73. HRMS (ESI, positive ion) [M+H]+ of Compound (**A19**).

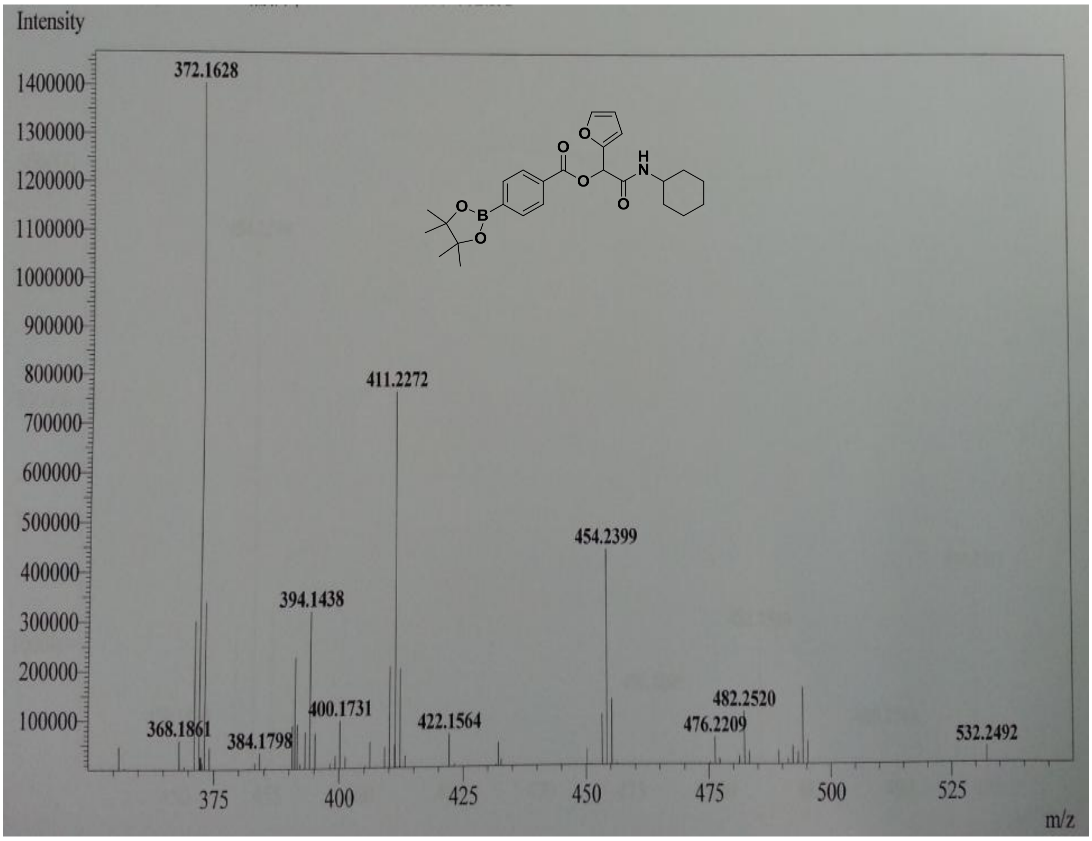

Figure S74. 600 MHz ^{1}H-NMR of Compound (**A19**) in Chloroform-*d*.

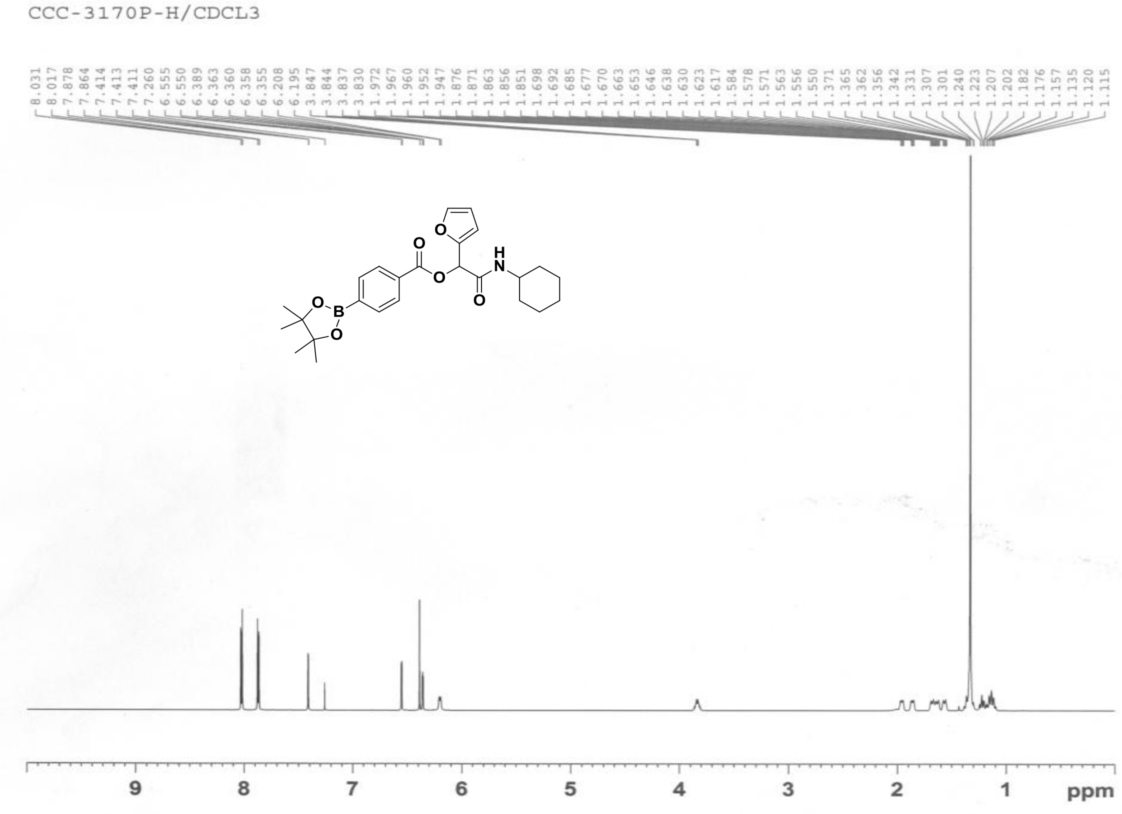

Figure S75. 600 MHz ^{13}C-NMR of Compound (**A19**) in Chloroform-*d*.

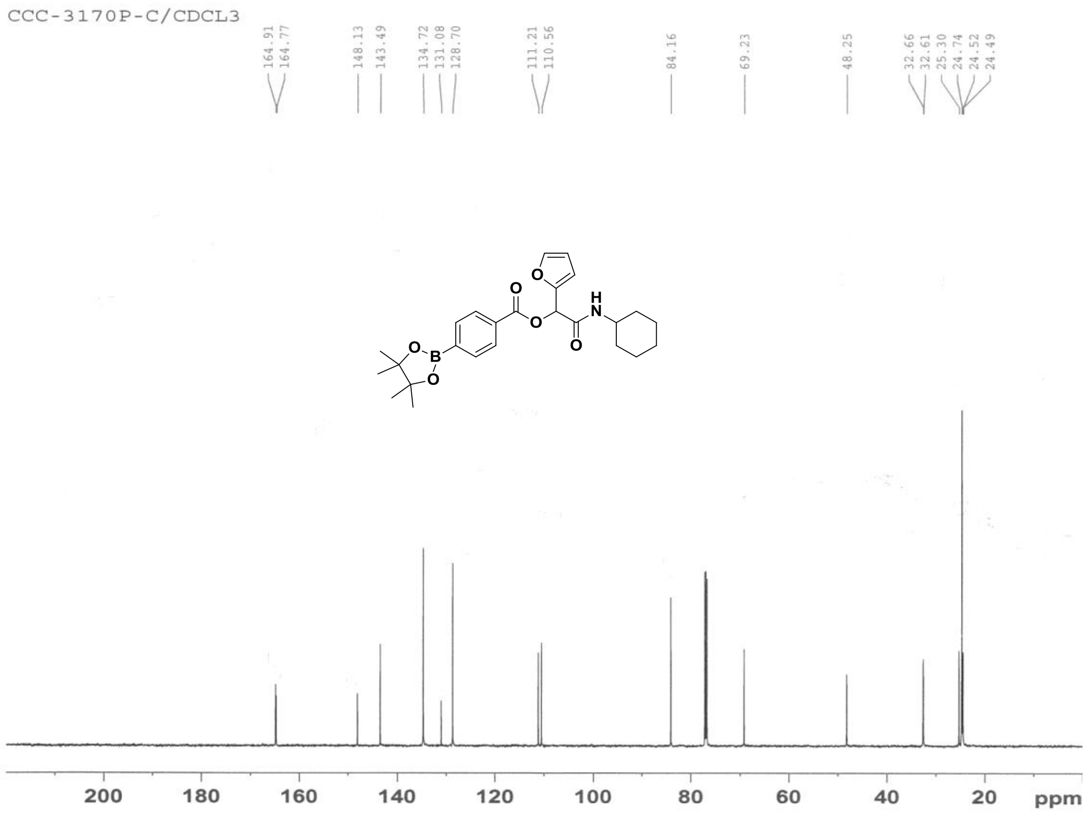

Figure S76. 600 MHz ^{11}B-NMR of Compound (**A19**) in Chloroform-*d*.

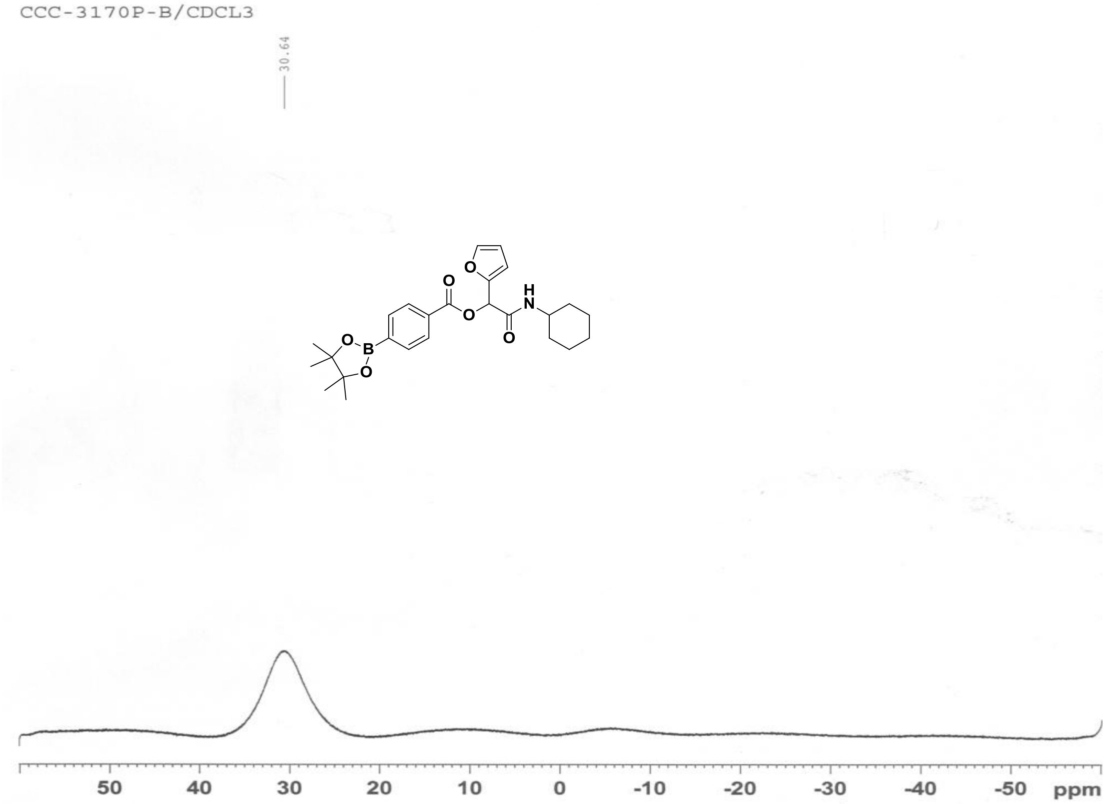

Figure S77. HRMS (ESI, positive ion) $[M+H]^+$ of Compound (**A20**).

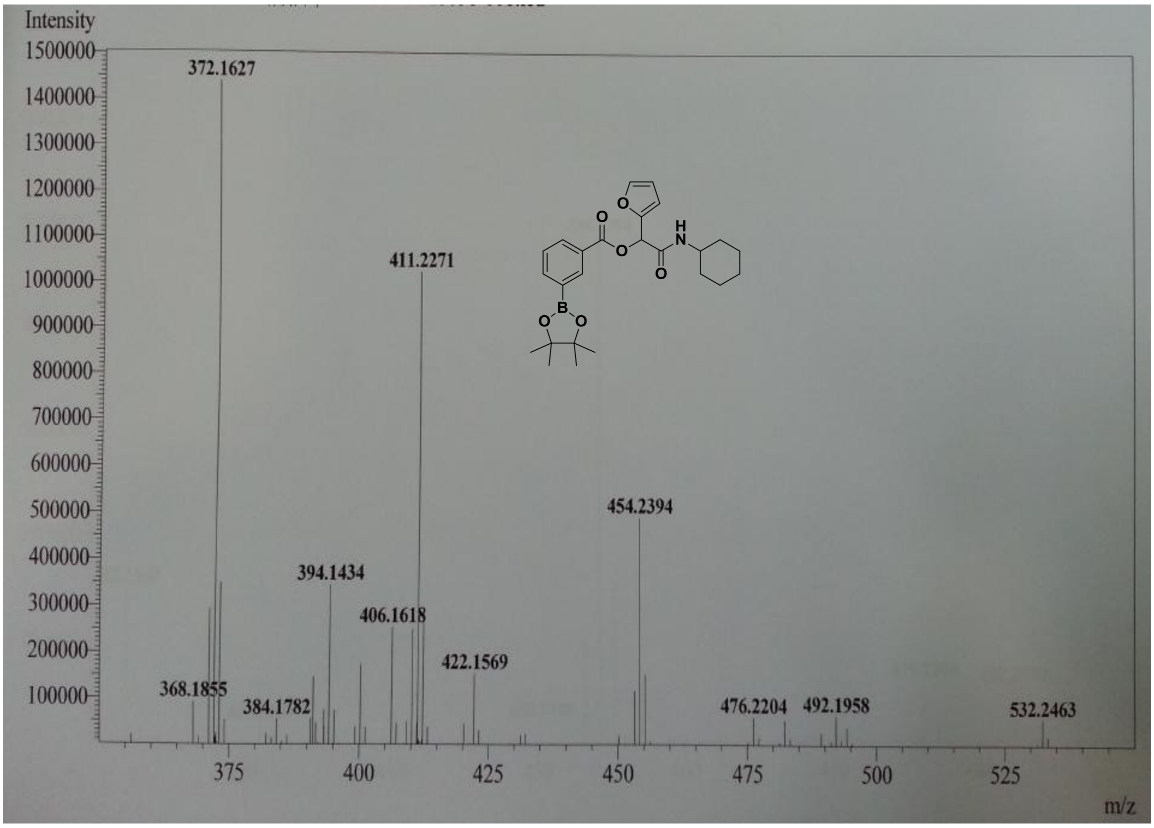

Figure S78. 600 MHz ^{1}H-NMR of Compound (**A20**) in Chloroform-*d*.

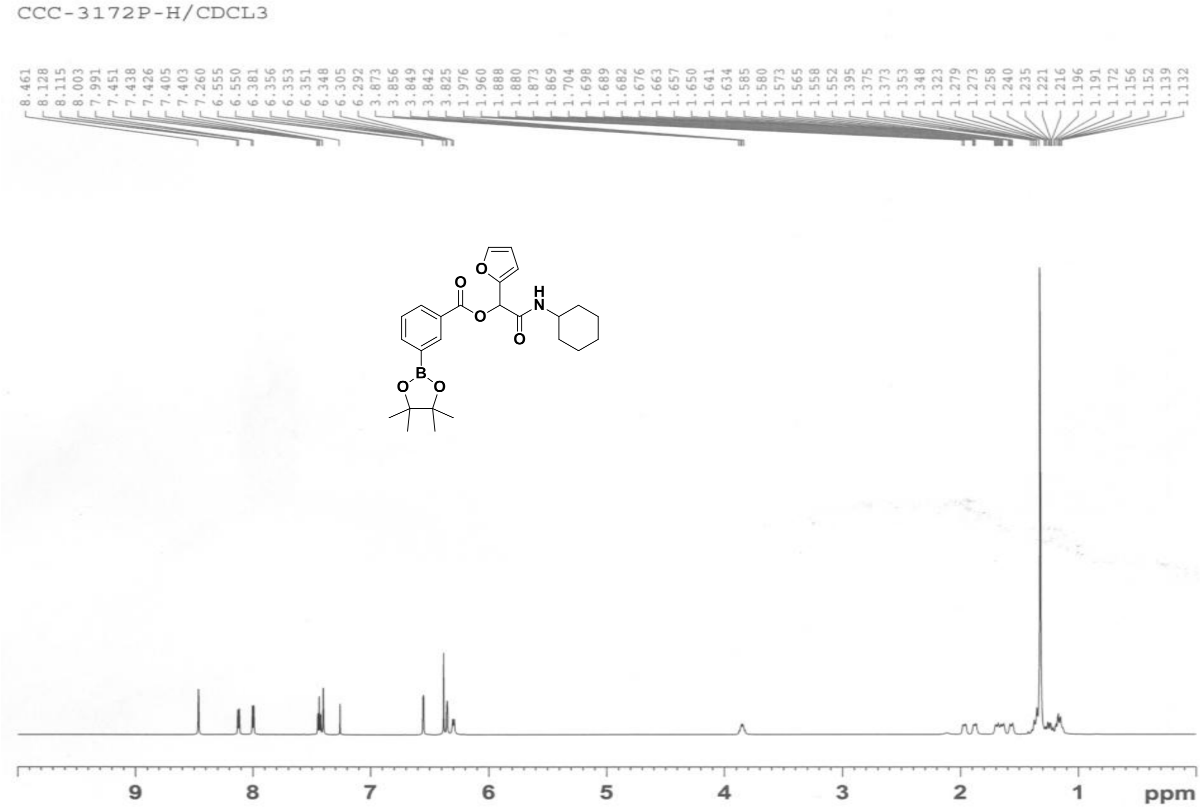

Figure S79. 600 MHz ^{13}C-NMR of Compound (**A20**) in Chloroform-*d*.

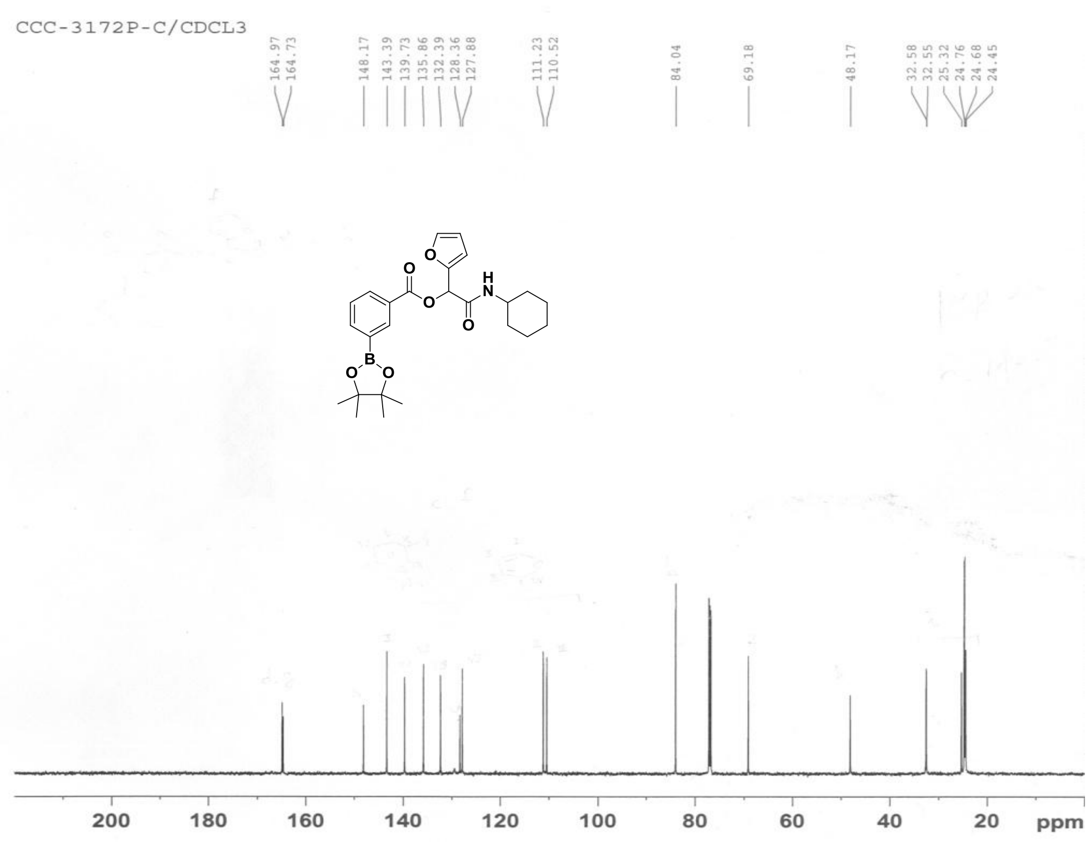

Figure S80. 600 MHz ^{11}B-NMR of Compound (**A20**) in Chloroform-*d*.

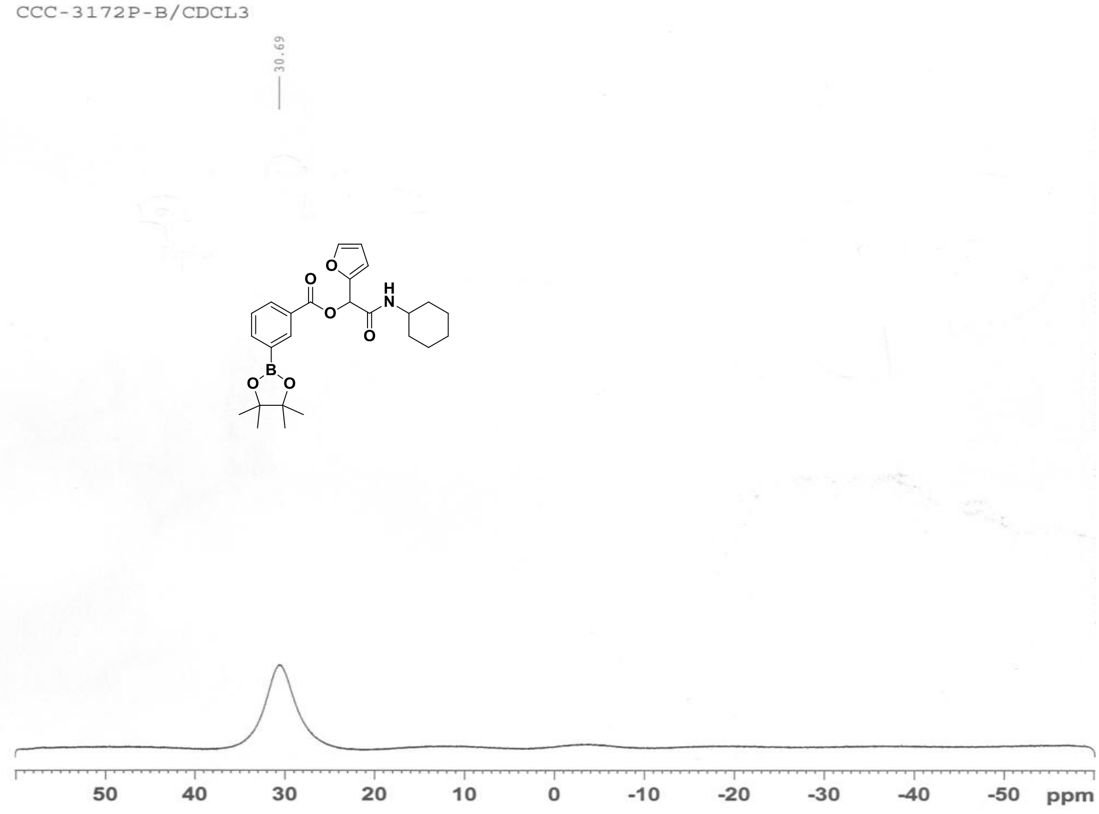

Figure S80. 600 MHz ^{11}B-NMR of Compound (**A20**) in Chloroform-*d*.

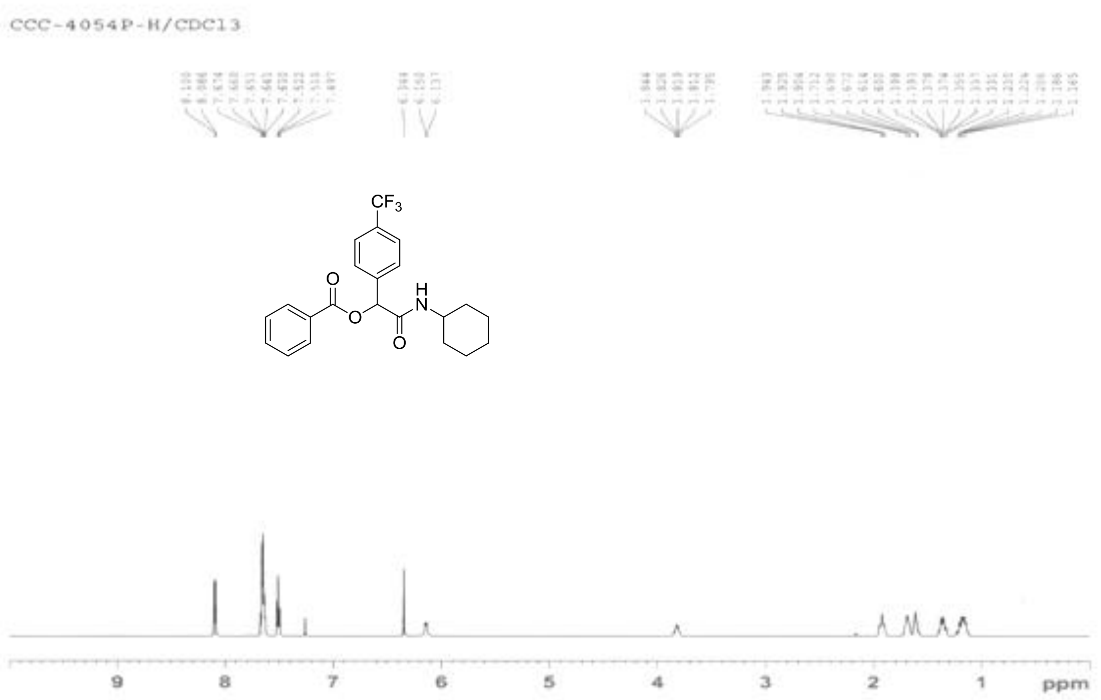

Figure S82. 600 MHz ^{13}C-NMR of Compound (**A21**) in Chloroform-*d*.

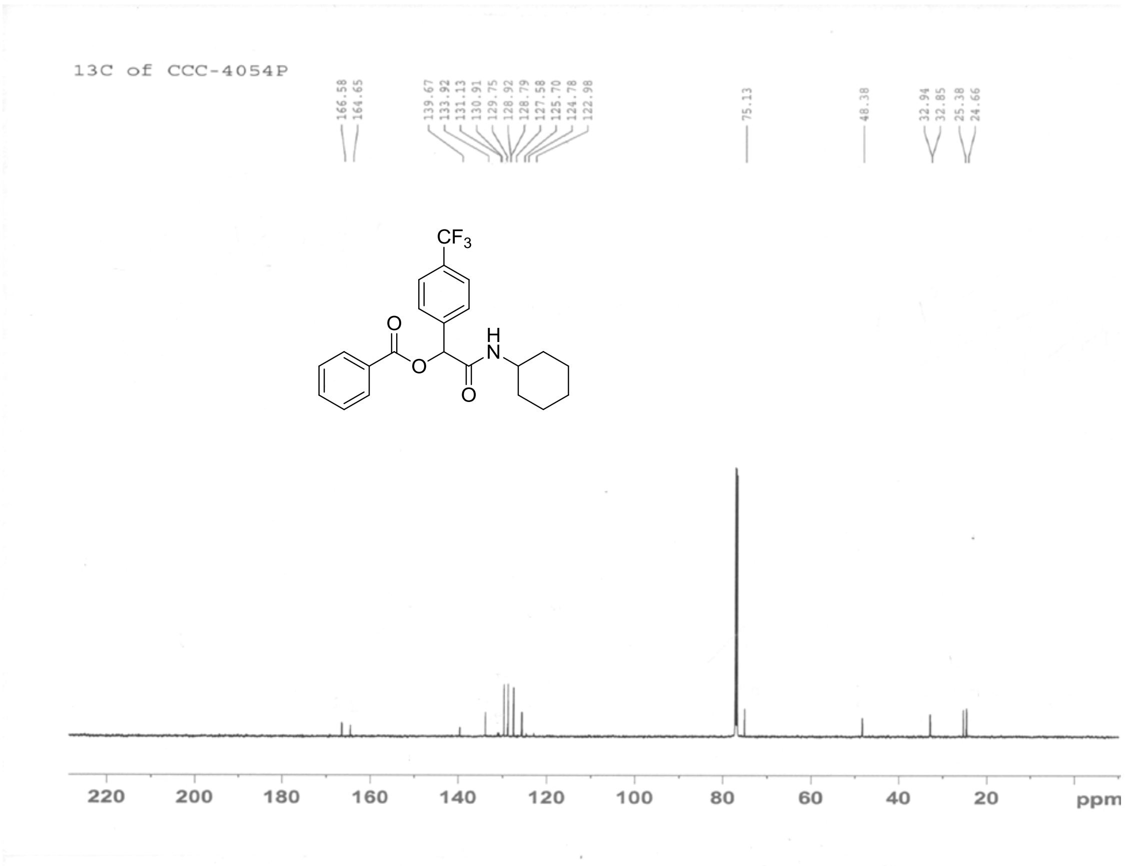

Figure S83. HRMS (ESI, positive ion) $[M+H]^+$ of Compound (**B1**).

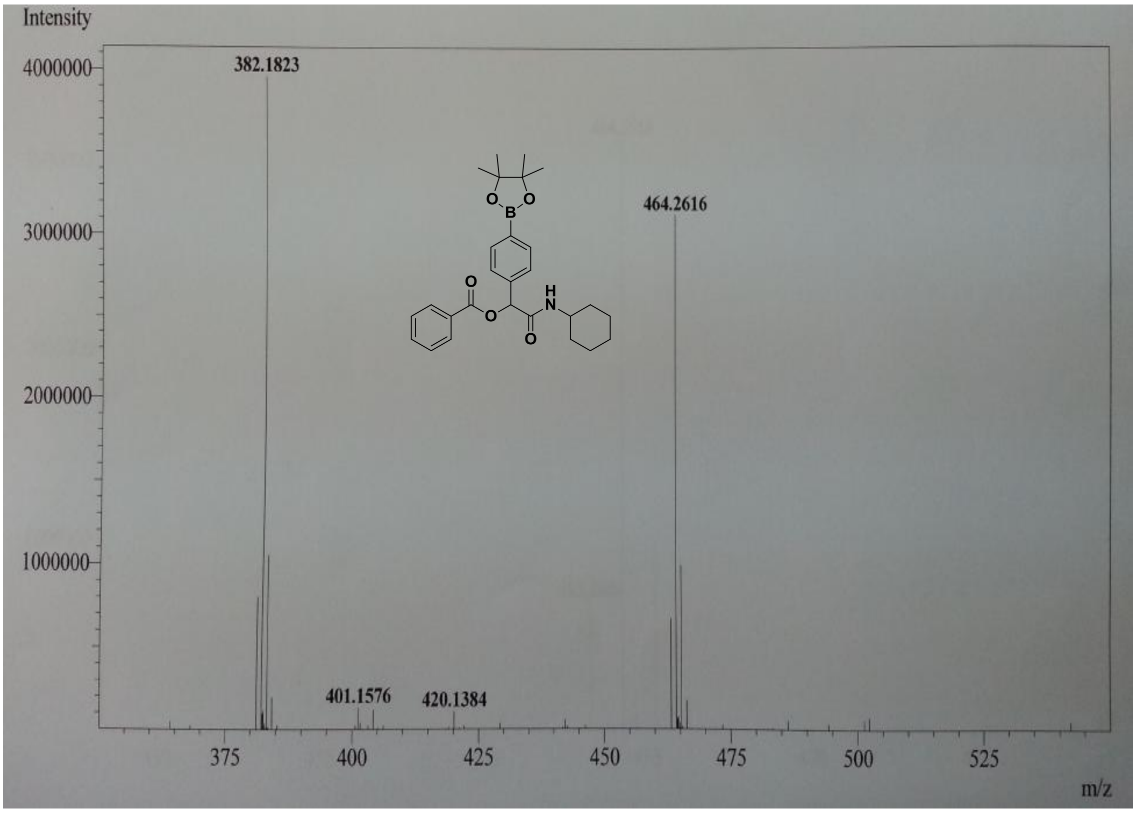

Figure S84. 600 MHz ^{1}H-NMR of Compound (B1) in Chloroform-*d*.

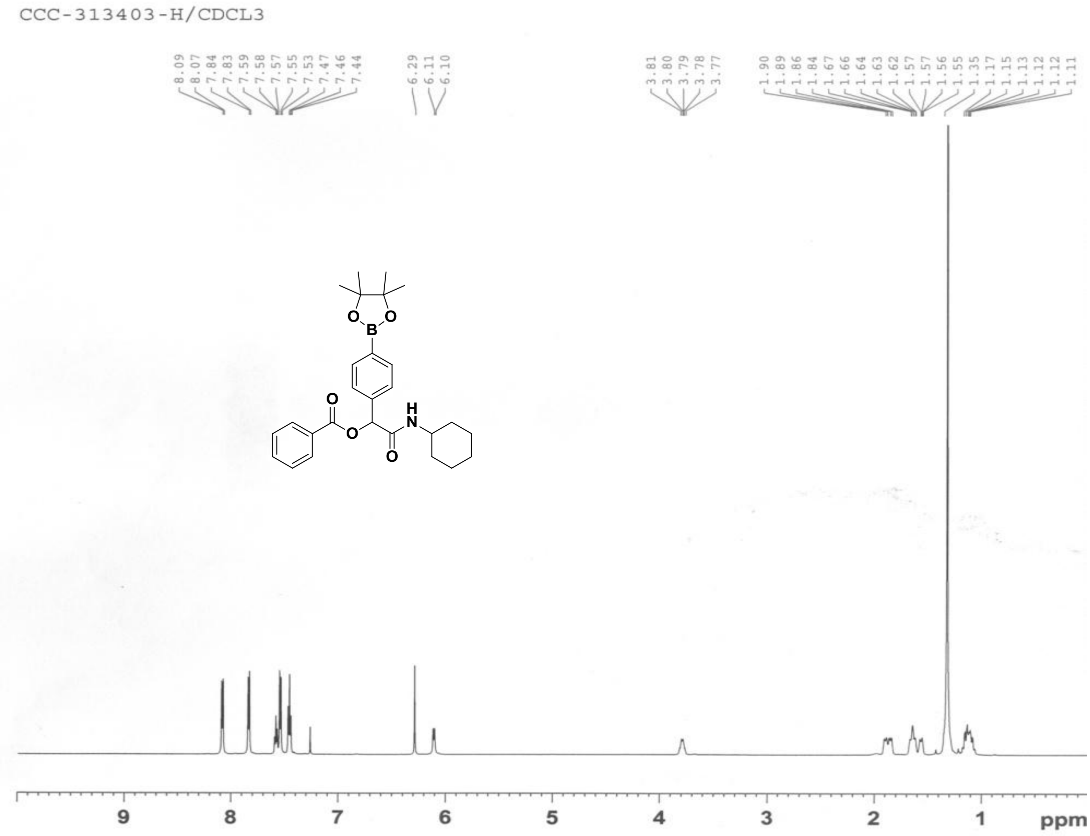

Figure S85. 600 MHz ^{13}C-NMR of Compound (B1) in Chloroform-*d*.

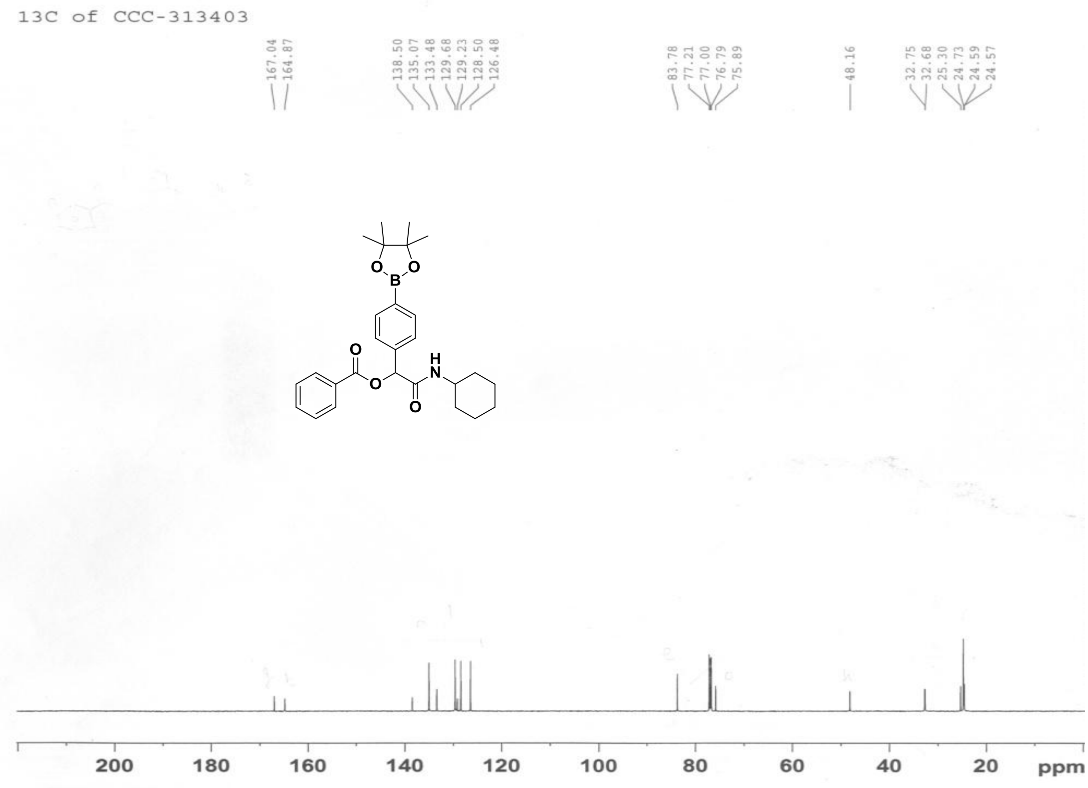

Figure S86. 600 MHz ^{11}B-NMR of Compound (B1) in Chloroform-*d*.

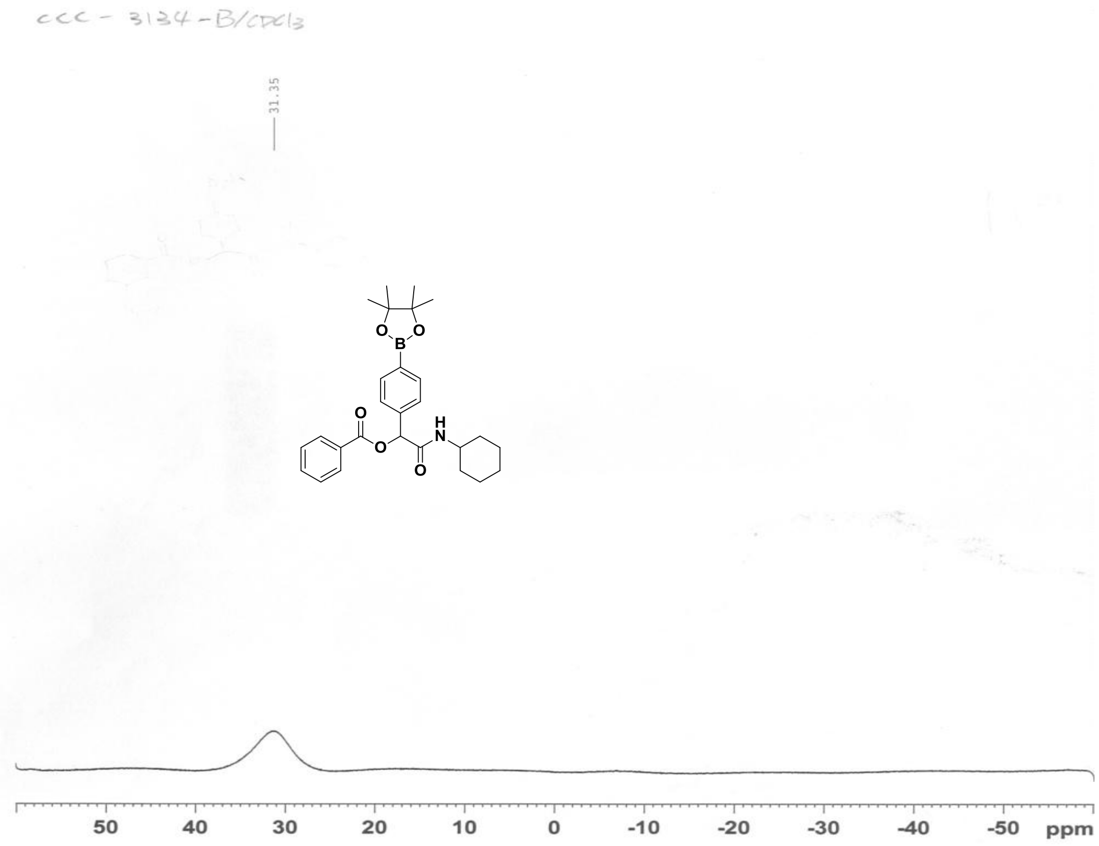

Figure S87. HRMS (ESI, positive ion) $[M+H]^+$ of Compound (**B2**).

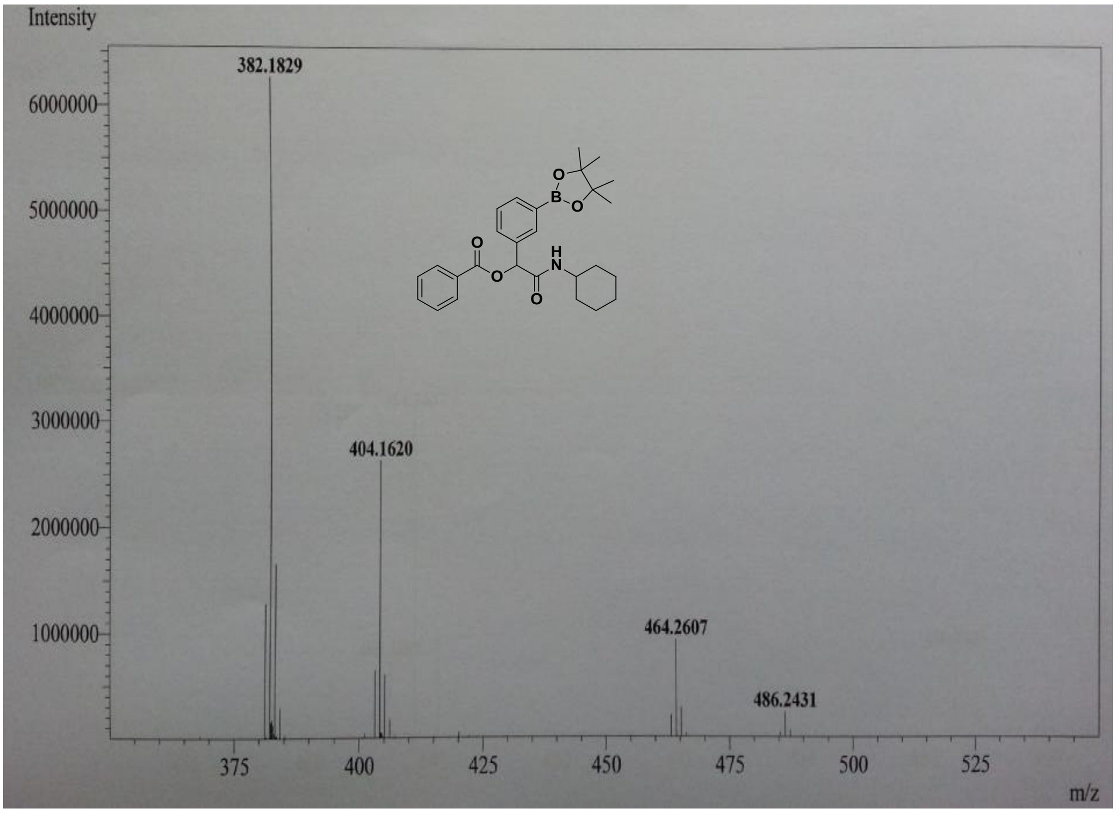

Figure S88. 600 MHz ^{1}H-NMR of Compound (**B2**) in Chloroform-*d*.

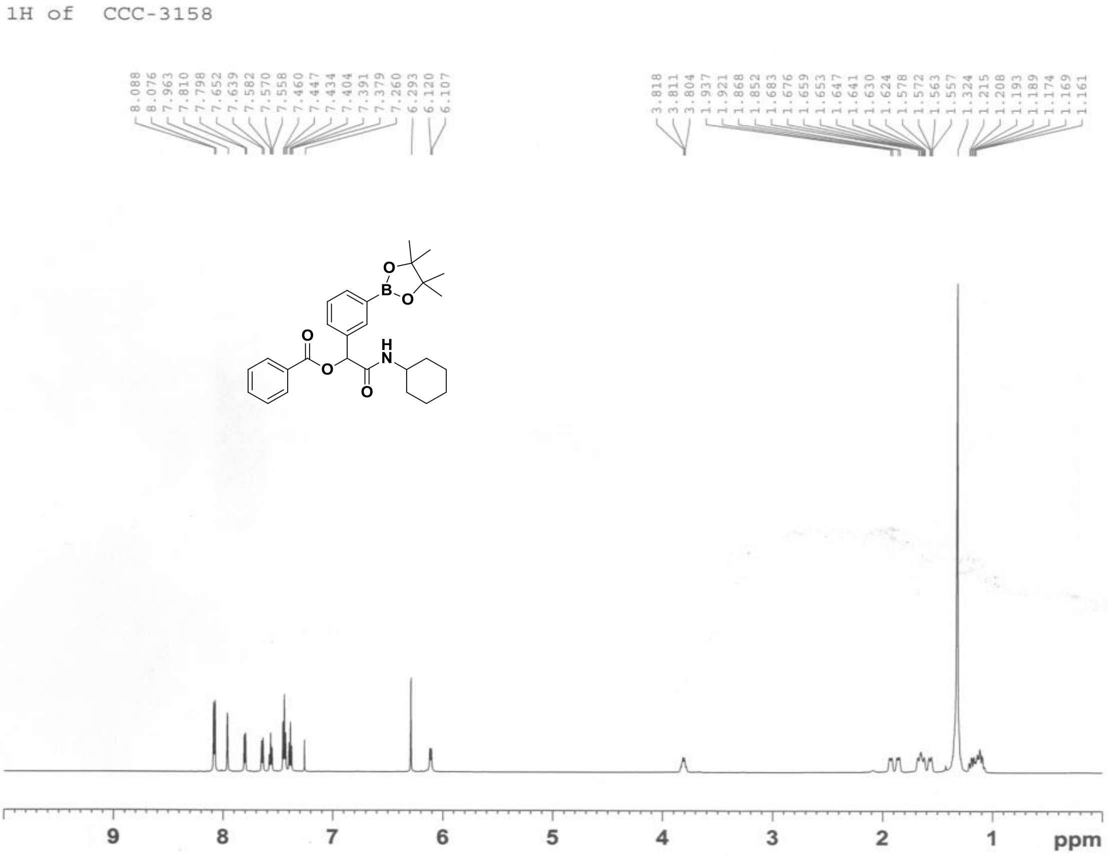

Figure S89. 600 MHz ^{13}C-NMR of Compound (**B2**) in Chloroform-*d*.

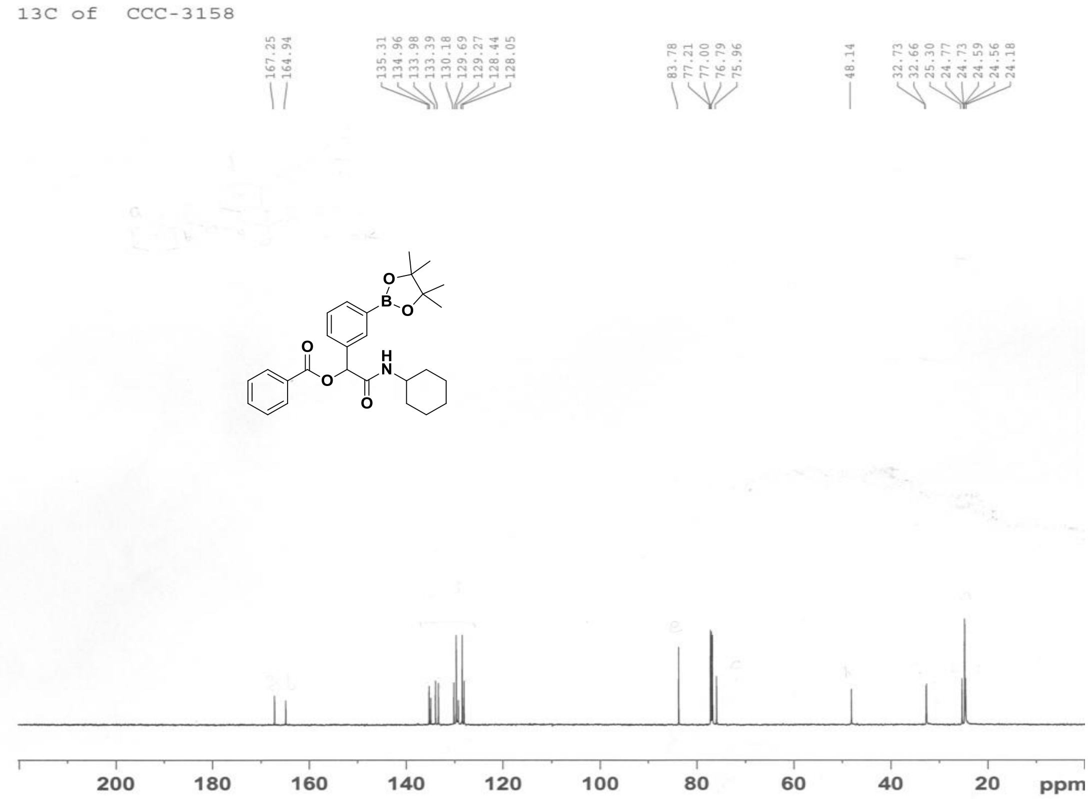

Figure S90. 600 MHz ^{11}B-NMR of Compound (**B2**) in Chloroform-*d*.

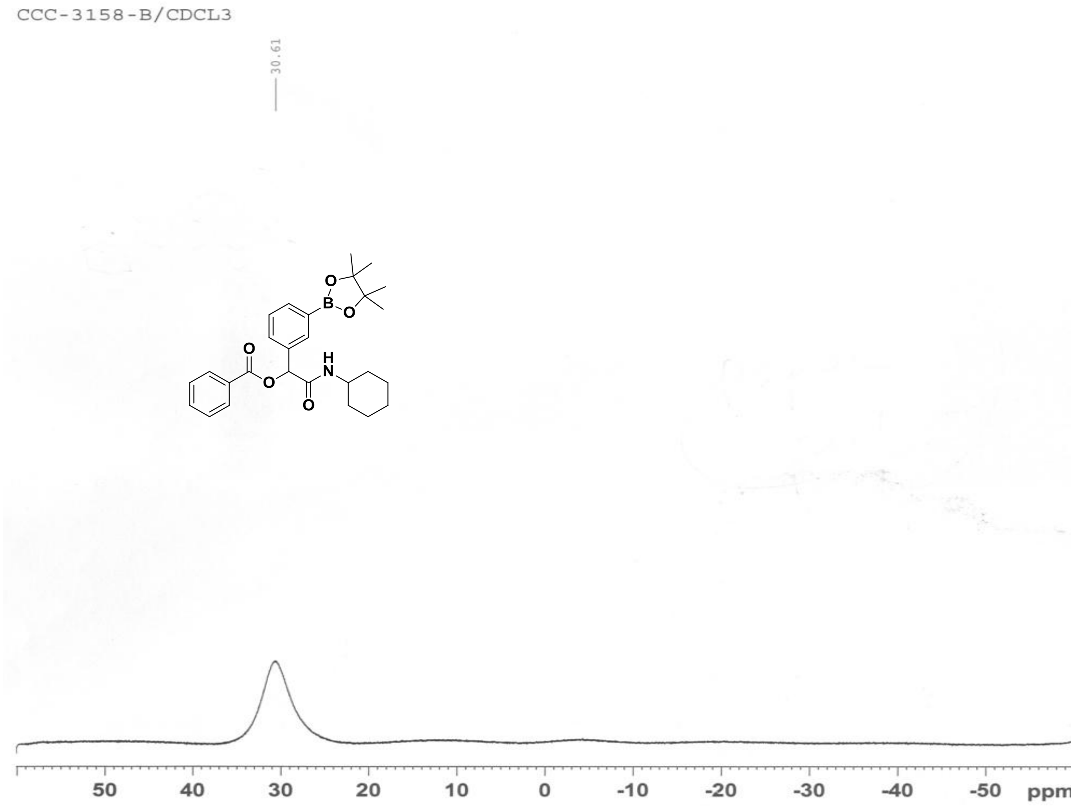

Figure S91. HRMS (ESI, positive ion) $[M+H]^+$ of Compound (**B3**).

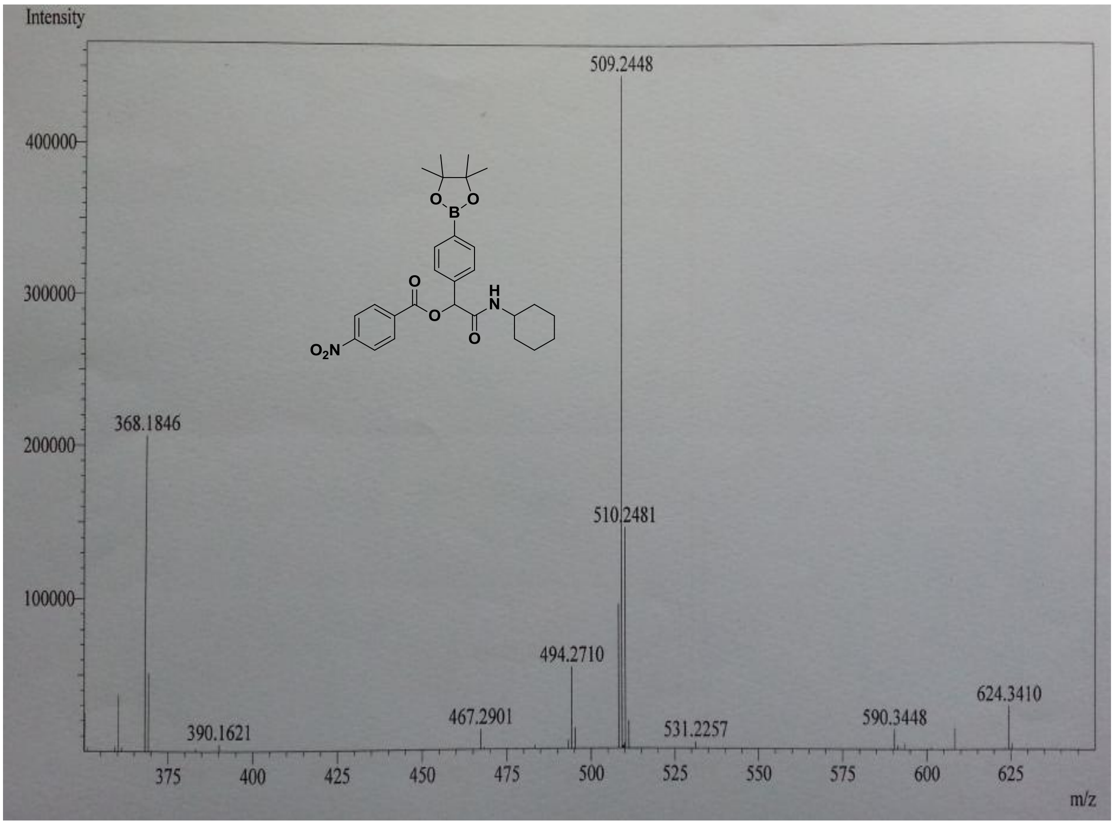

Figure S92. 600 MHz ^{1}H-NMR of Compound (**B3**) in Chloroform-*d*.

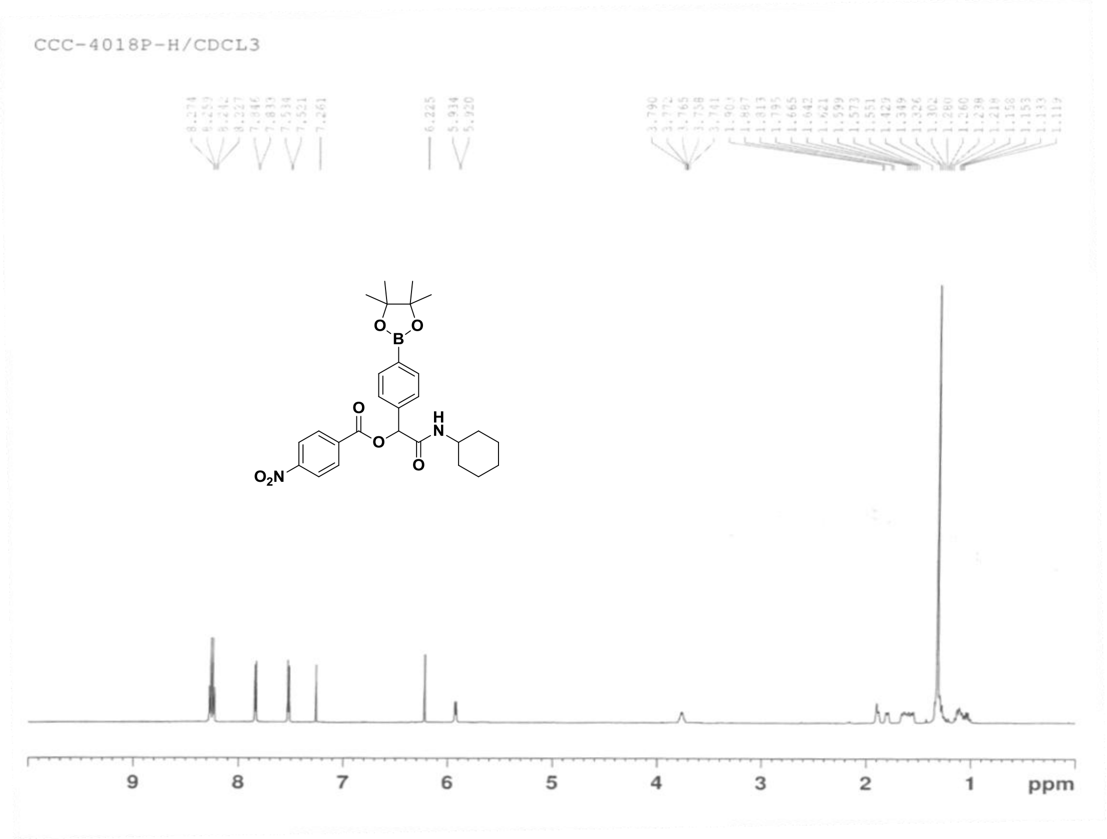

Figure S93. 600 MHz ^{13}C-NMR of Compound (**B3**) in Chloroform-*d*.

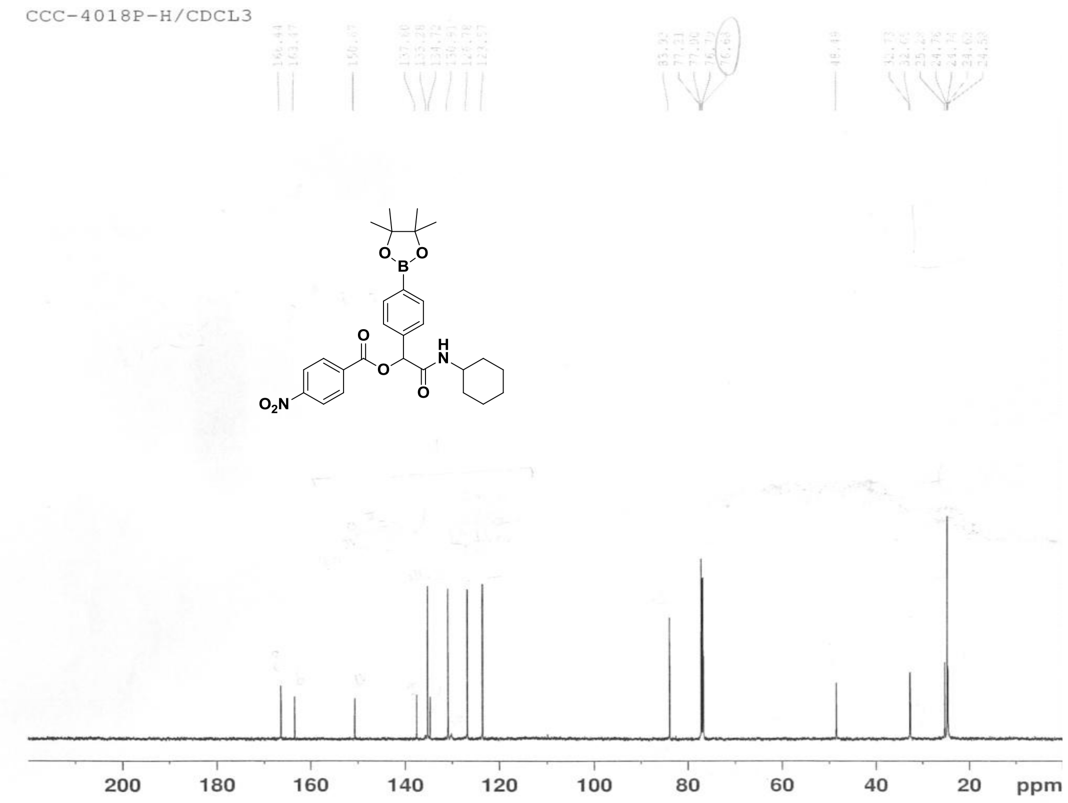

Figure S94. 600 MHz ^{11}B-NMR of Compound (**B3**) in Chloroform-*d*.

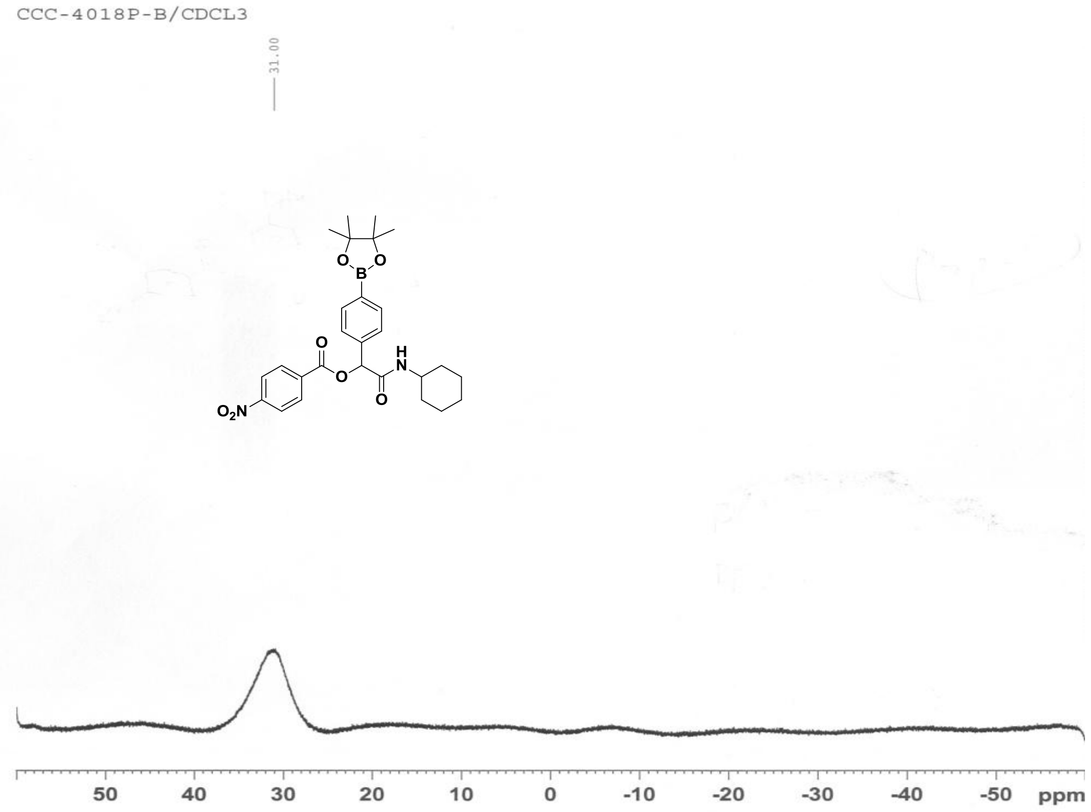

Figure S95. HRMS (ESI, positive ion) $[M+H]^+$ of Compound (**B4**).

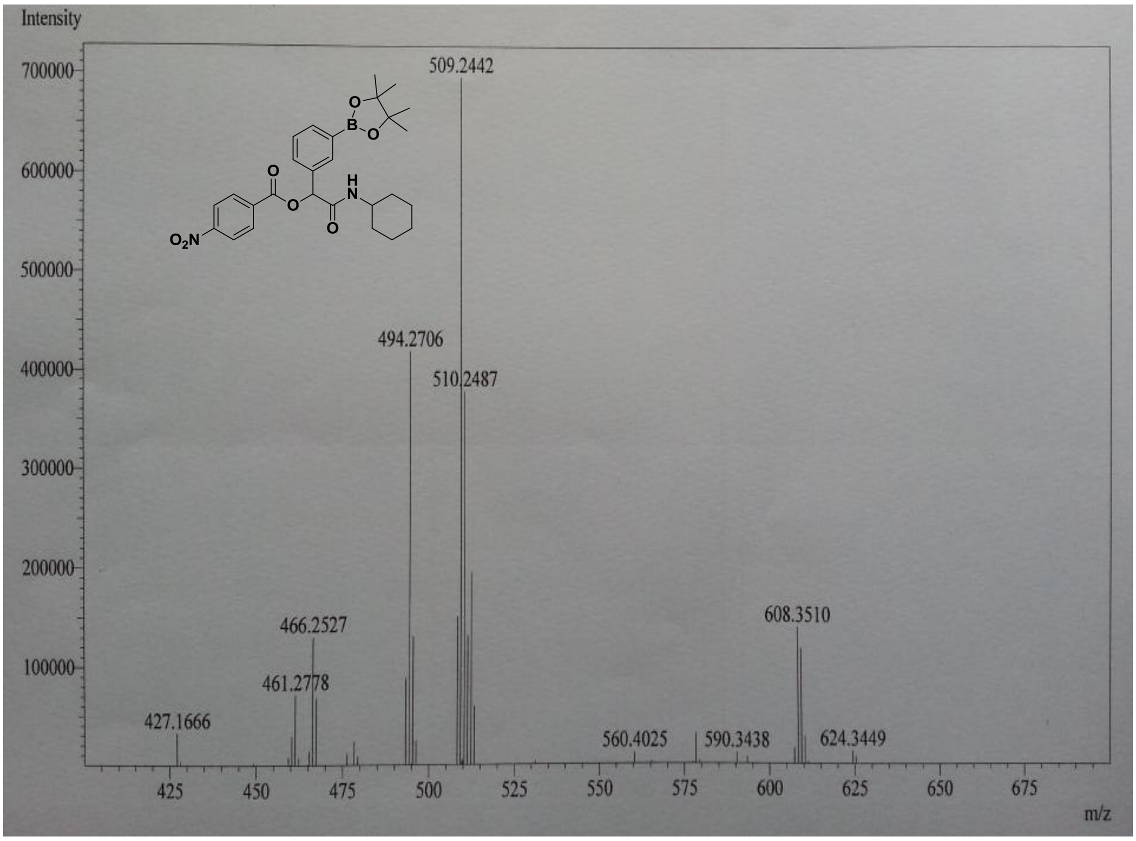

Figure S96. 600 MHz ^{1}H-NMR of Compound (**B4**) in Chloroform-*d*.

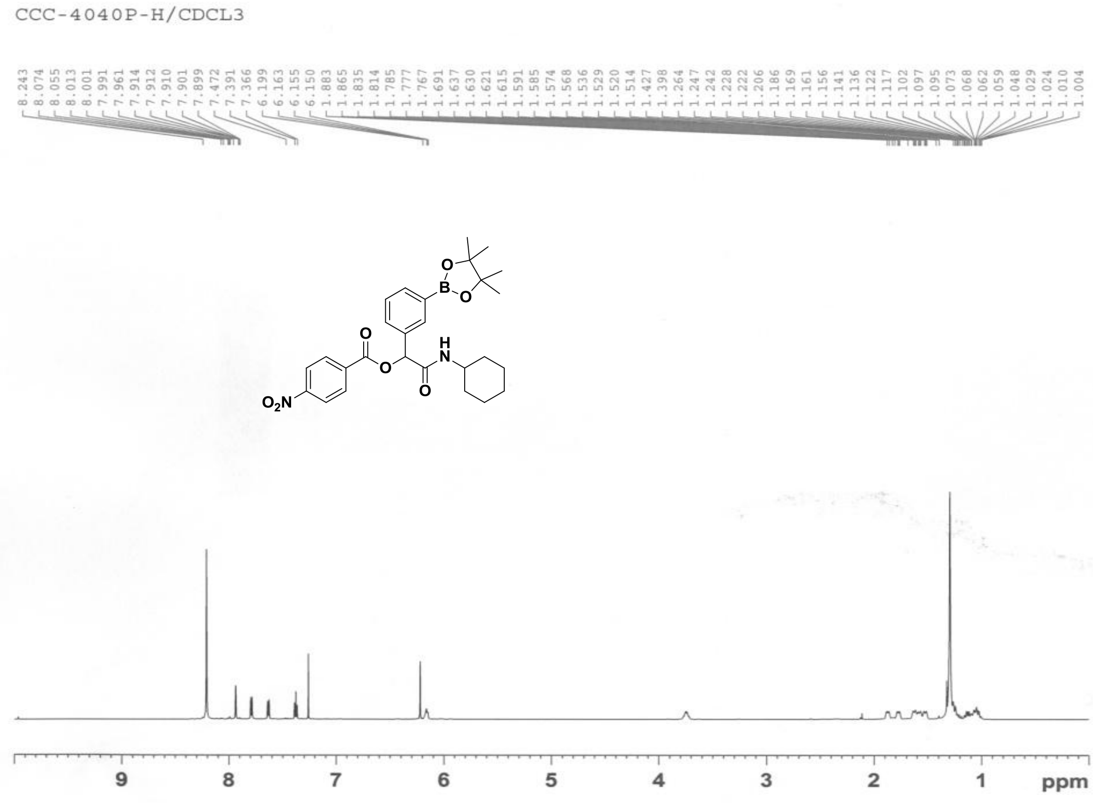

Figure S97. 600 MHz ^{13}C-NMR of Compound (**B4**) in Chloroform-*d*.

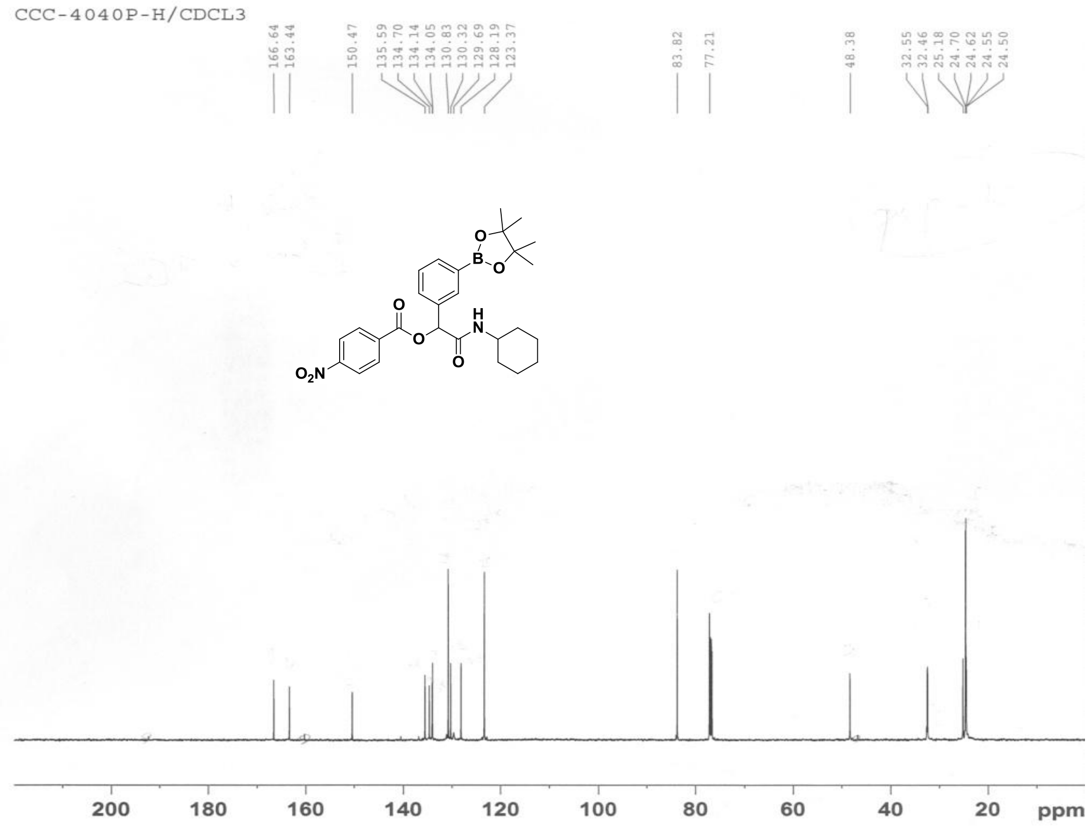

Figure S98. 600 MHz ^{11}B-NMR of Compound (**B4**) in Chloroform-*d*.

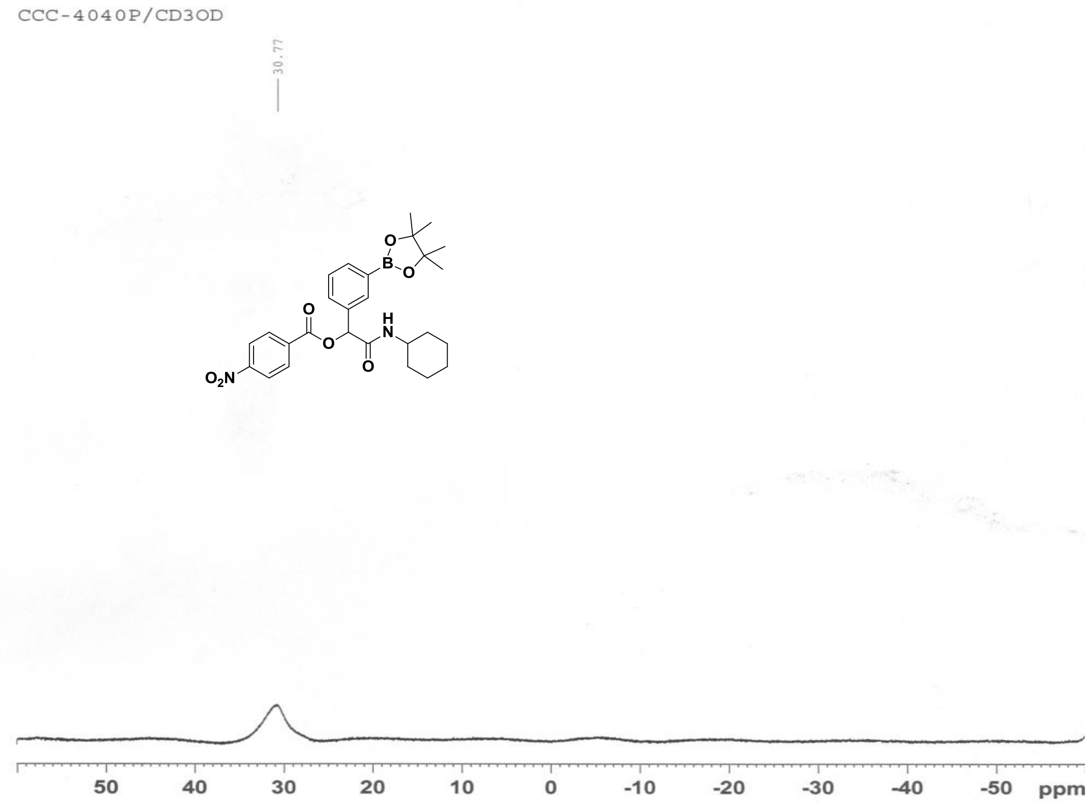

Figure S99. HRMS (ESI, positive ion) $[M+H]^+$ of Compound (**B5**).

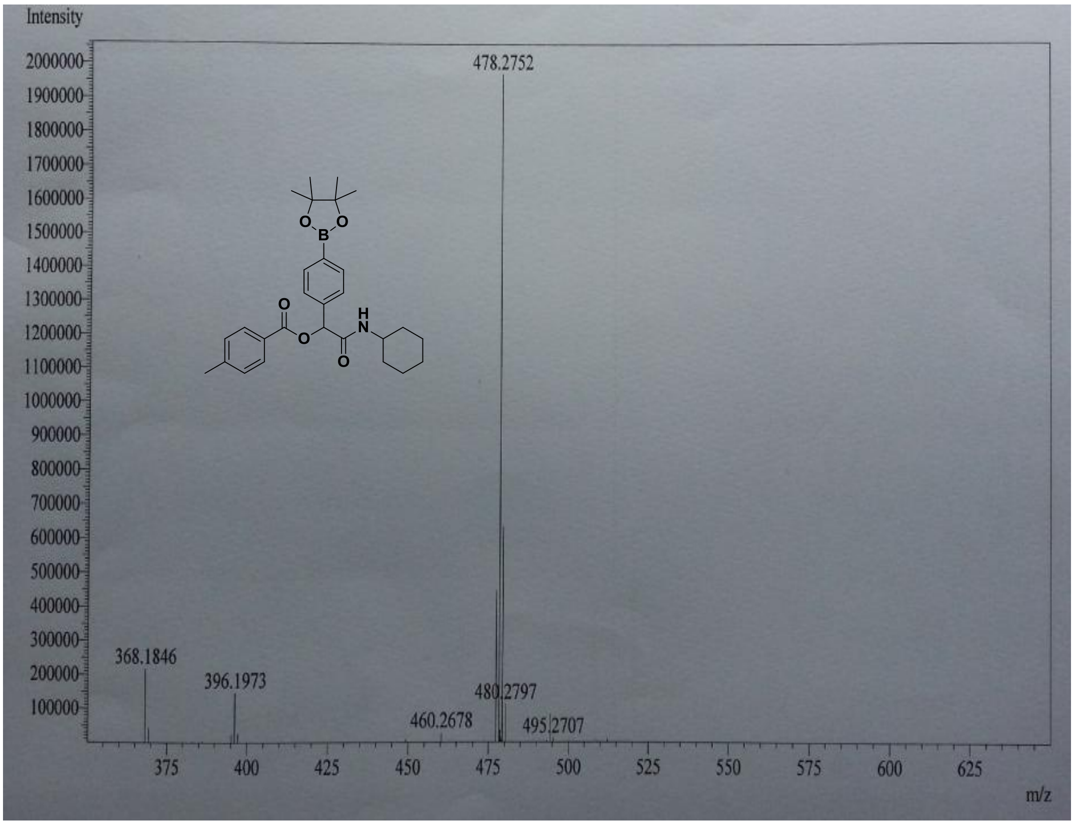

Figure S100. 600 MHz ^{1}H-NMR of Compound (**B5**) in Chloroform-*d*.

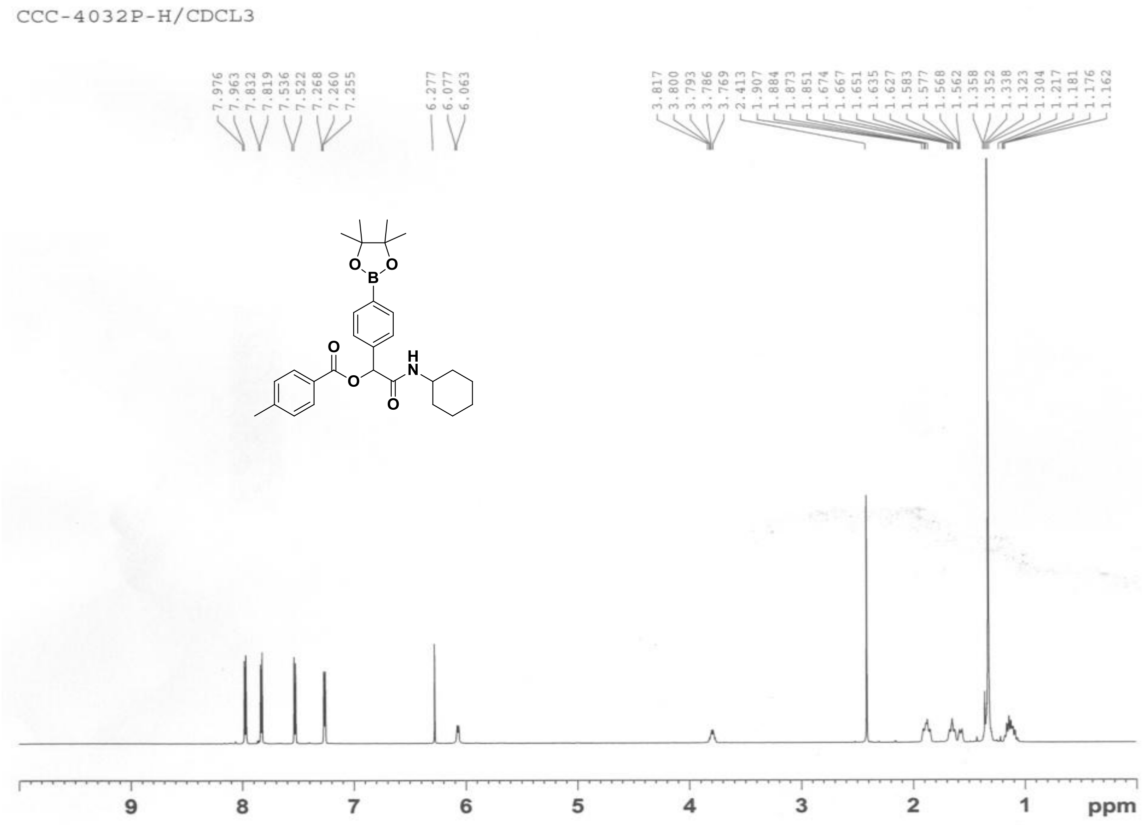

Figure S101. 600 MHz ^{13}C-NMR of Compound (**B5**) in Chloroform-*d*.

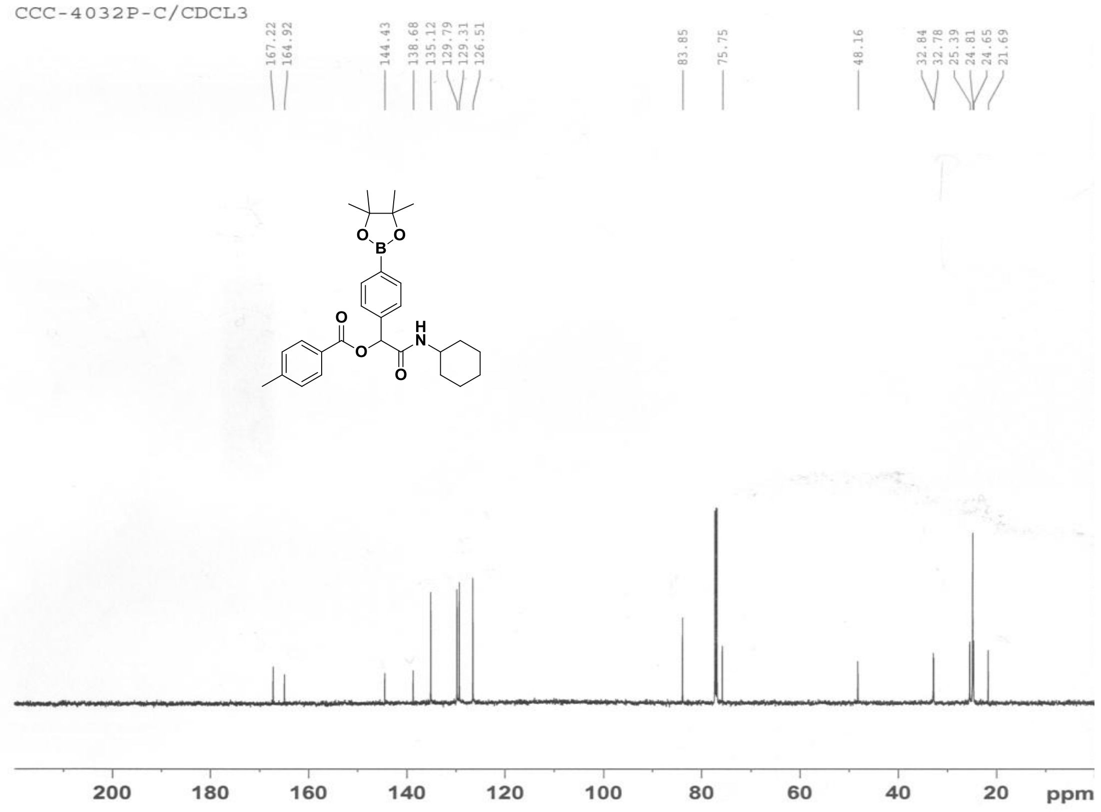

Figure S102. 600 MHz ^{11}B-NMR of Compound (**B5**) in Chloroform-*d*.

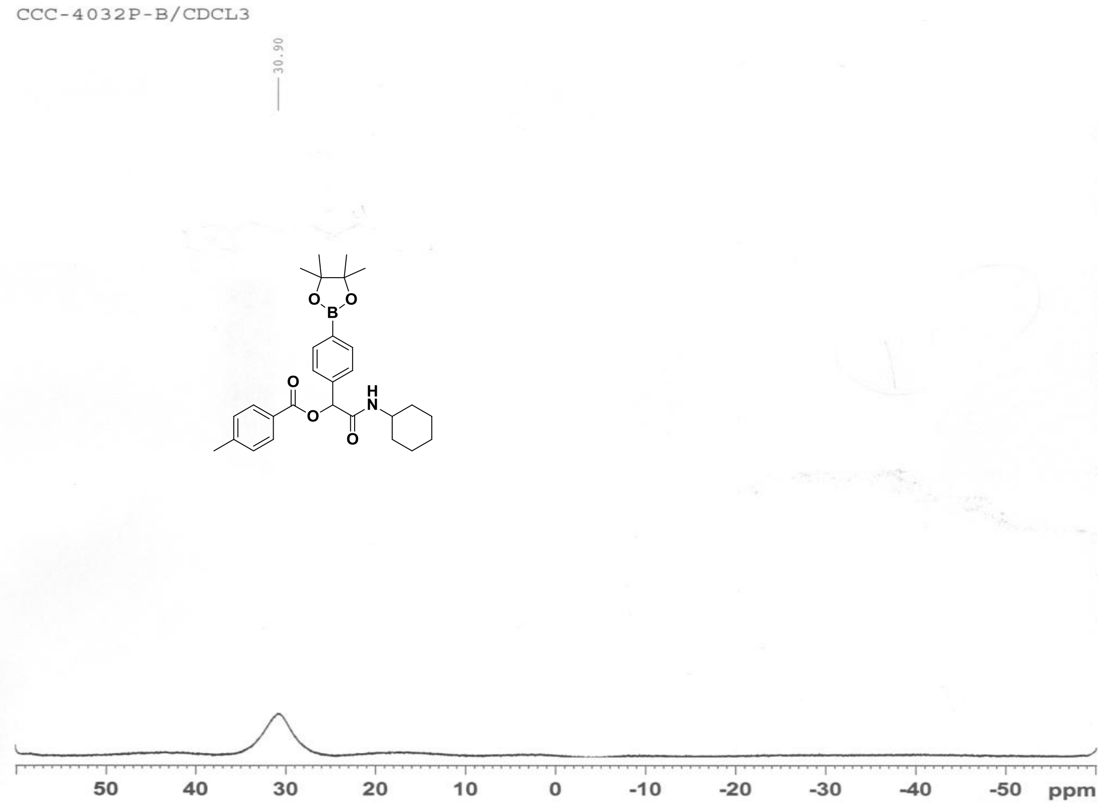

Figure S103. HRMS (ESI, positive ion) $[M+H]^+$ of Compound (**B6**).

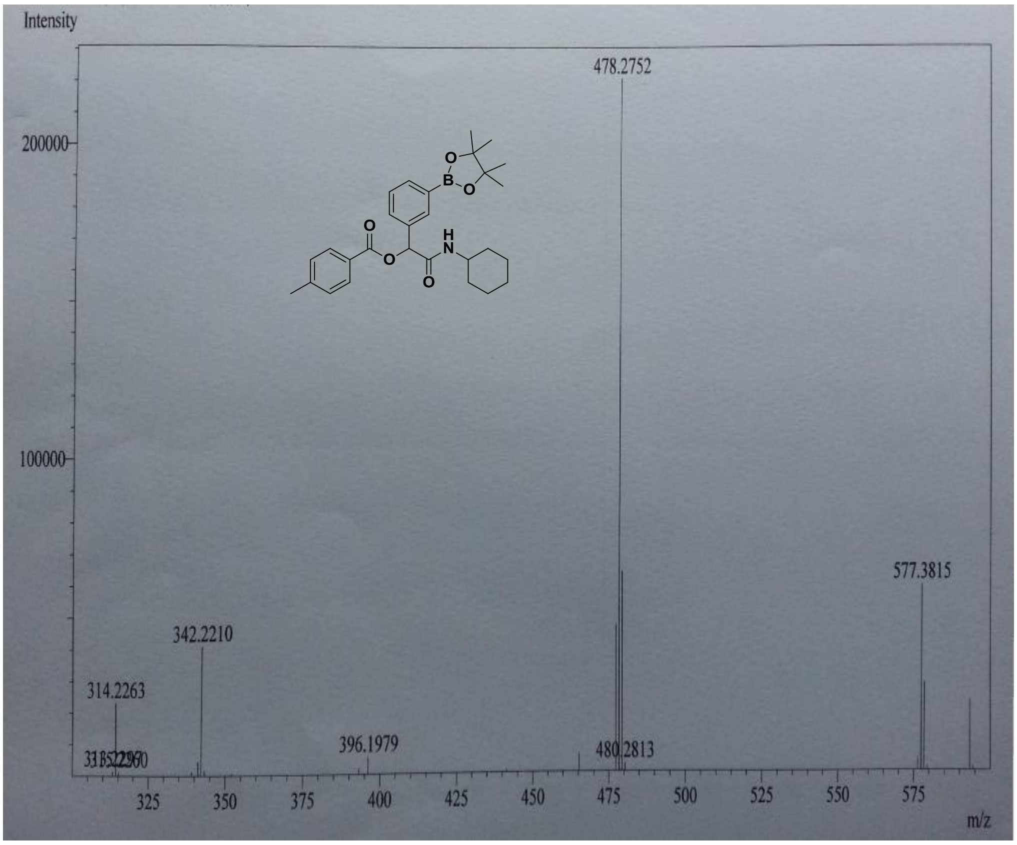

Figure S104. 600 MHz ^{1}H-NMR of Compound (**B6**) in Chloroform-*d*.

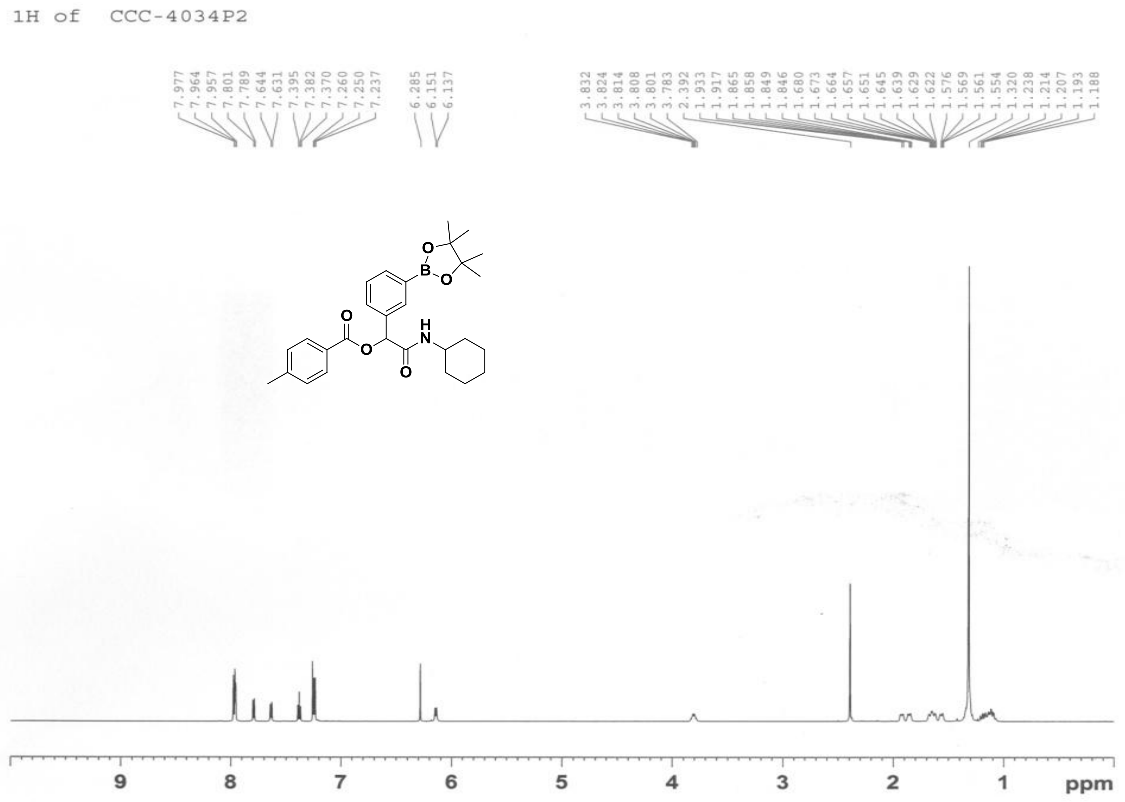

Figure S105. 600 MHz ^{13}C-NMR of Compound (**B6**) in Chloroform-*d*.

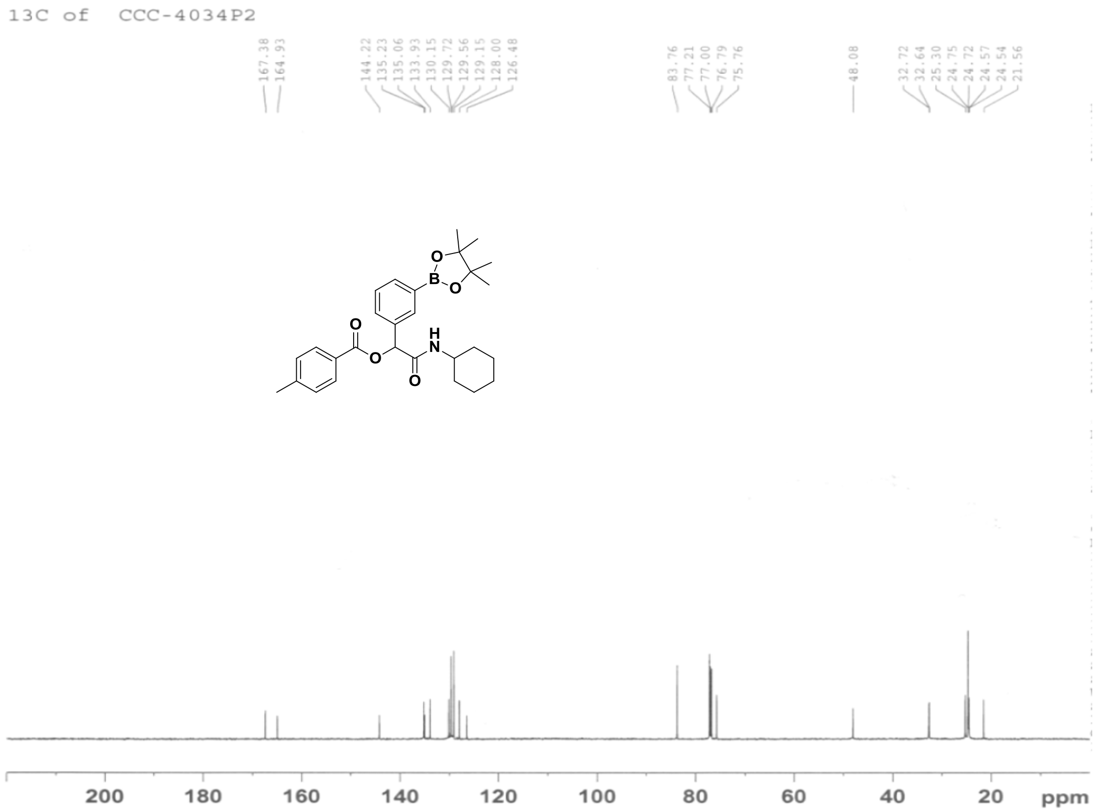

Figure S106. 600 MHz ^{11}B-NMR of Compound (**B6**) in Chloroform-*d*.

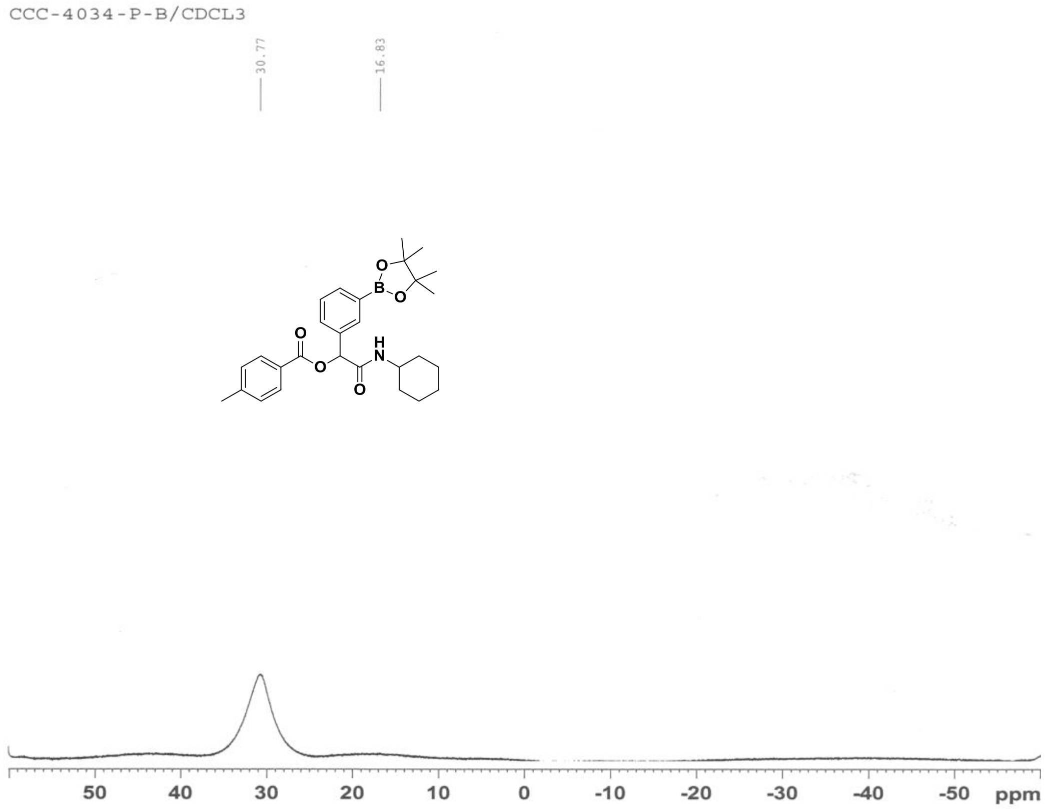

Figure S107. HRMS (ESI, positive ion) $[M+H]^+$ of Compound (**B7**).

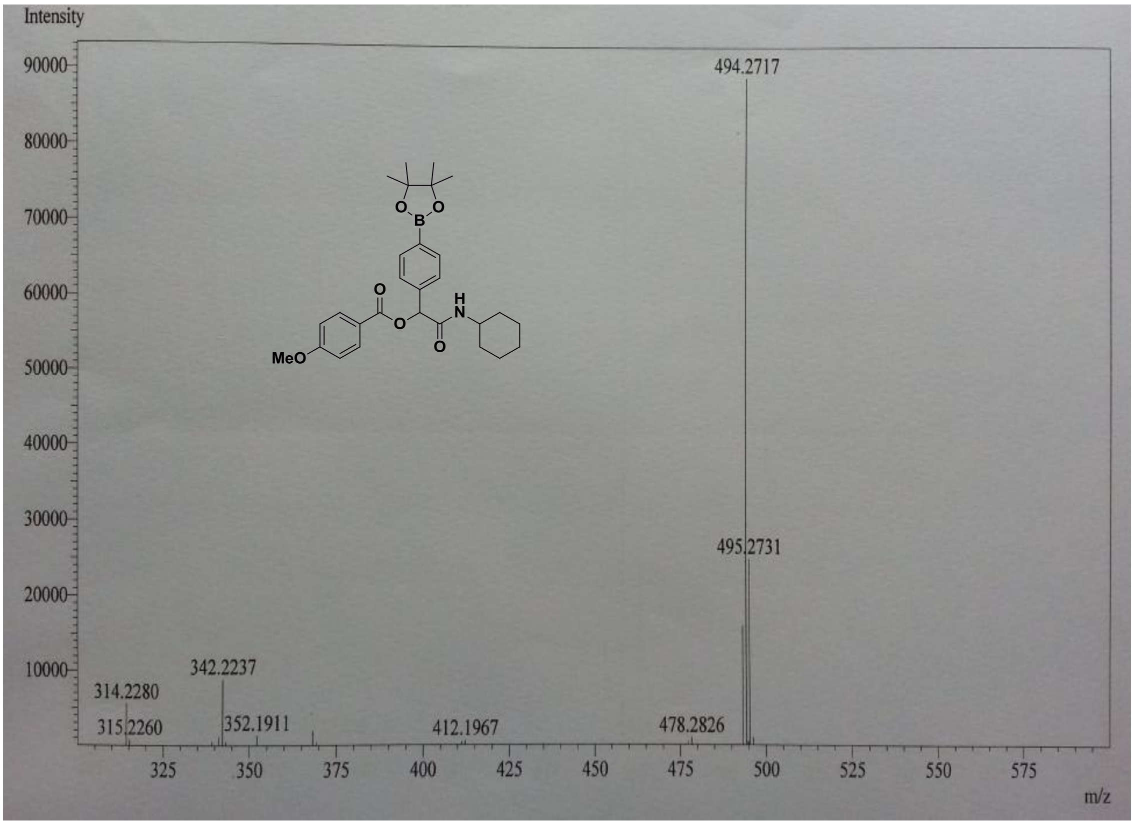

Figure S108. 600 MHz ^{1}H-NMR of Compound (B7) in Chloroform-*d*.

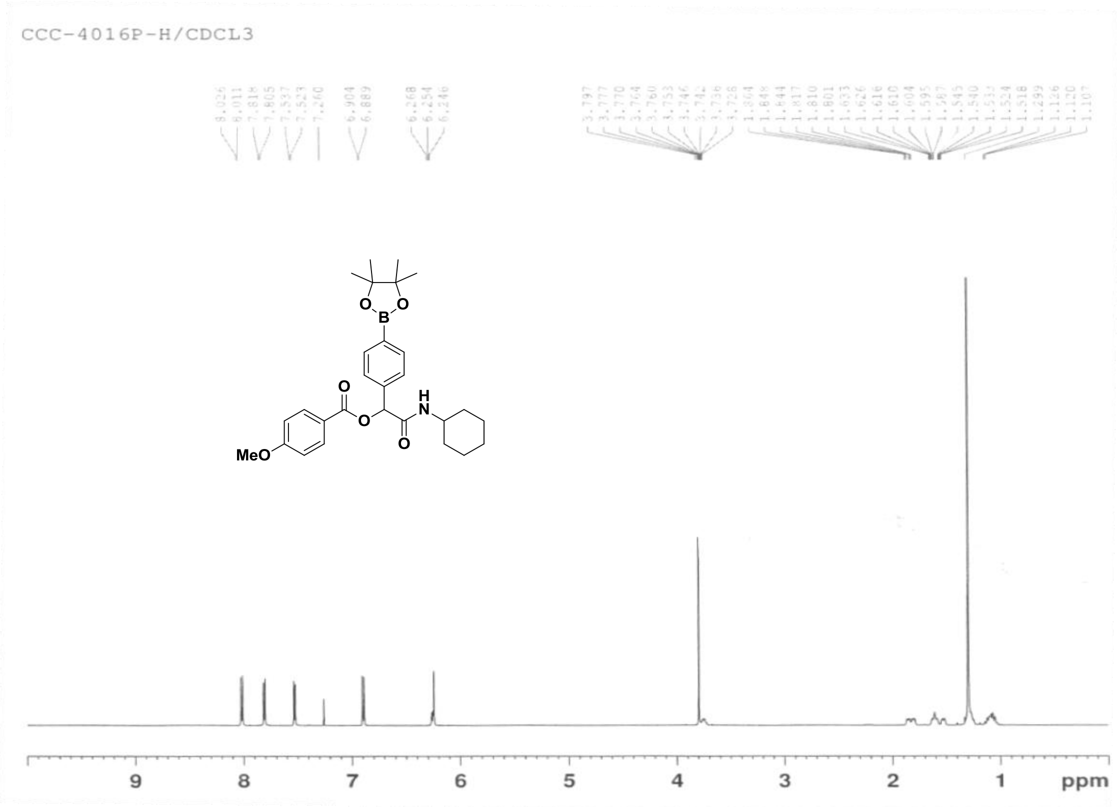

Figure S109. 600 MHz ^{13}C-NMR of Compound (B7) in Chloroform-*d*.

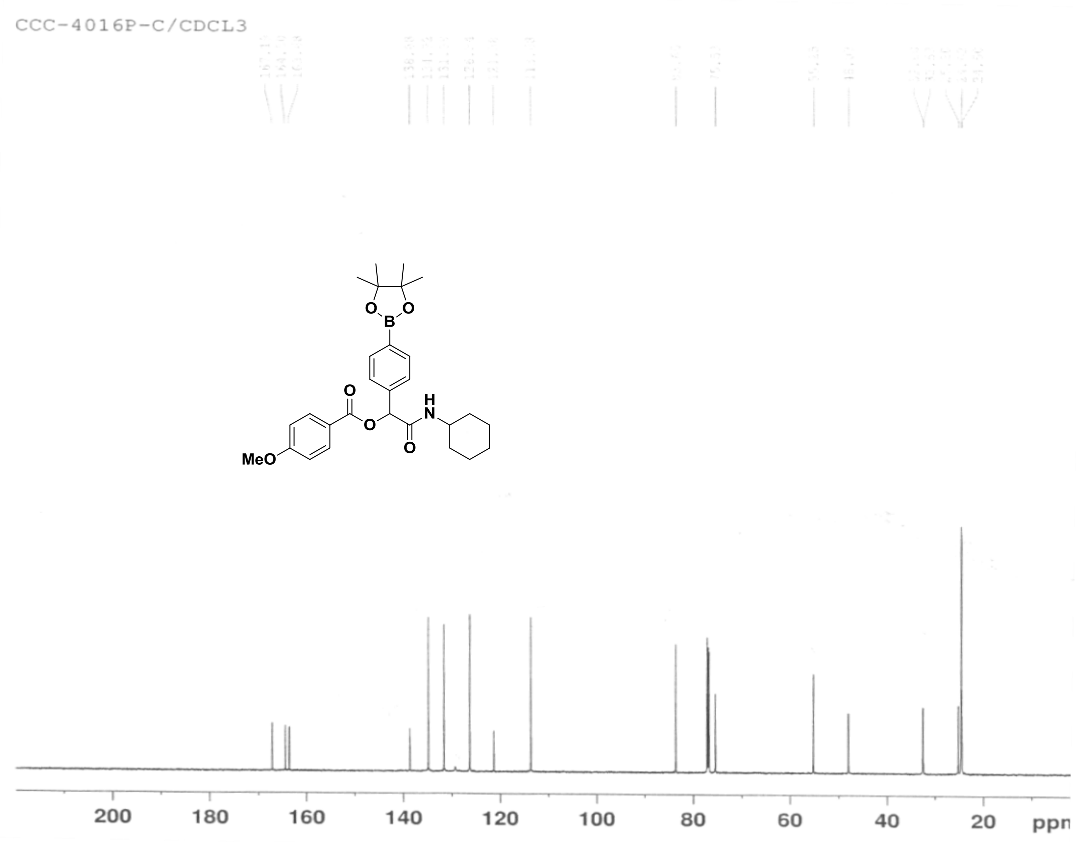

Figure S110. 600 MHz ^{11}B-NMR of Compound (B7) in Chloroform-*d*.

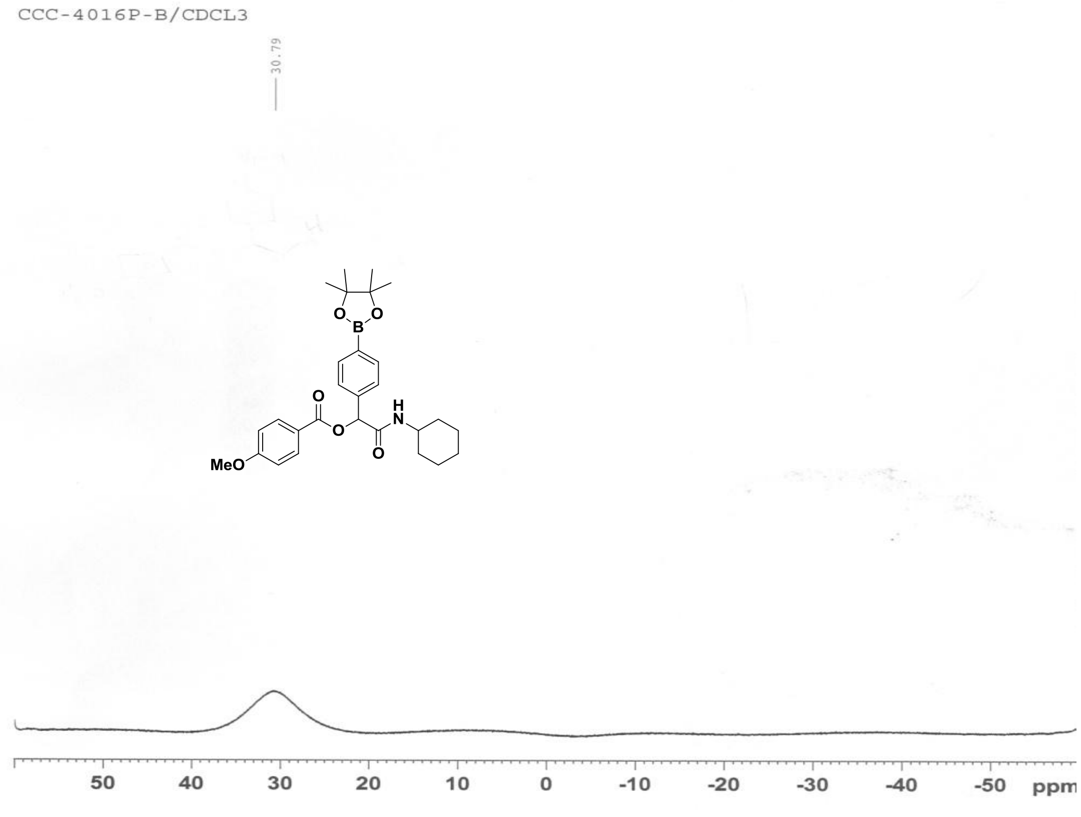

Figure S111. HRMS (ESI, positive ion) $[M+H]^+$ of Compound (**B8**).

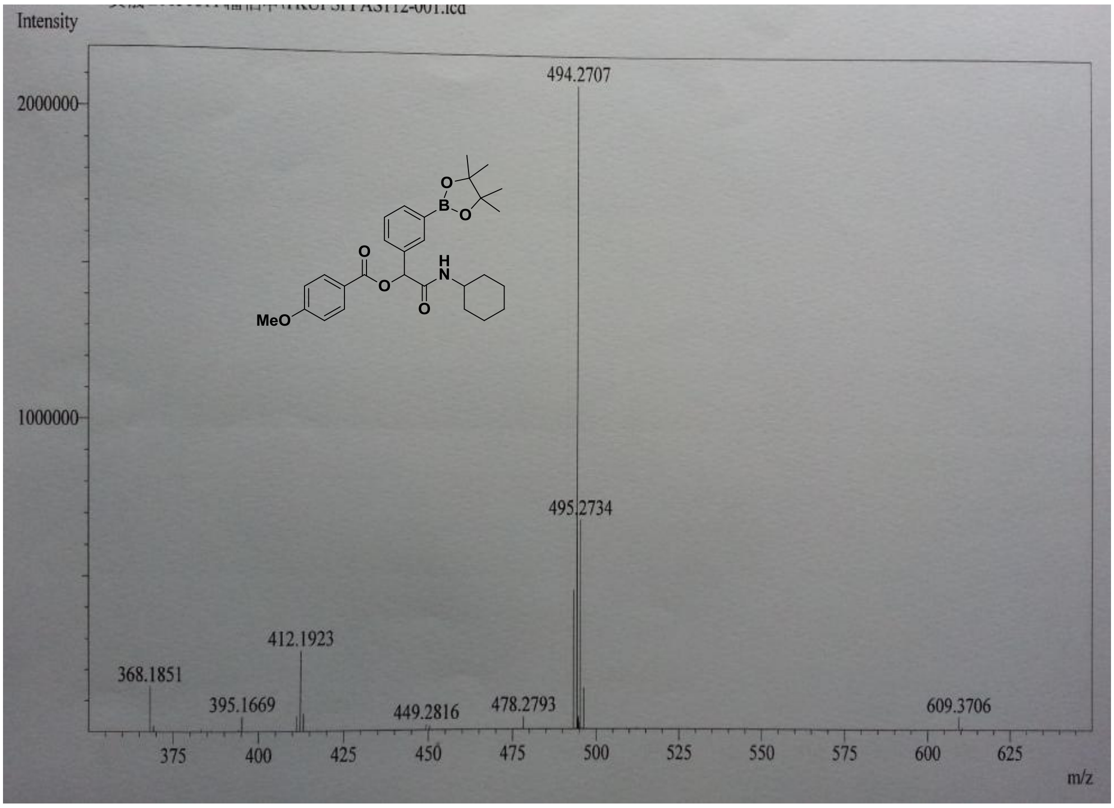

Figure S112. 600 MHz ^{1}H-NMR of Compound (**B8**) in Chloroform-*d*.

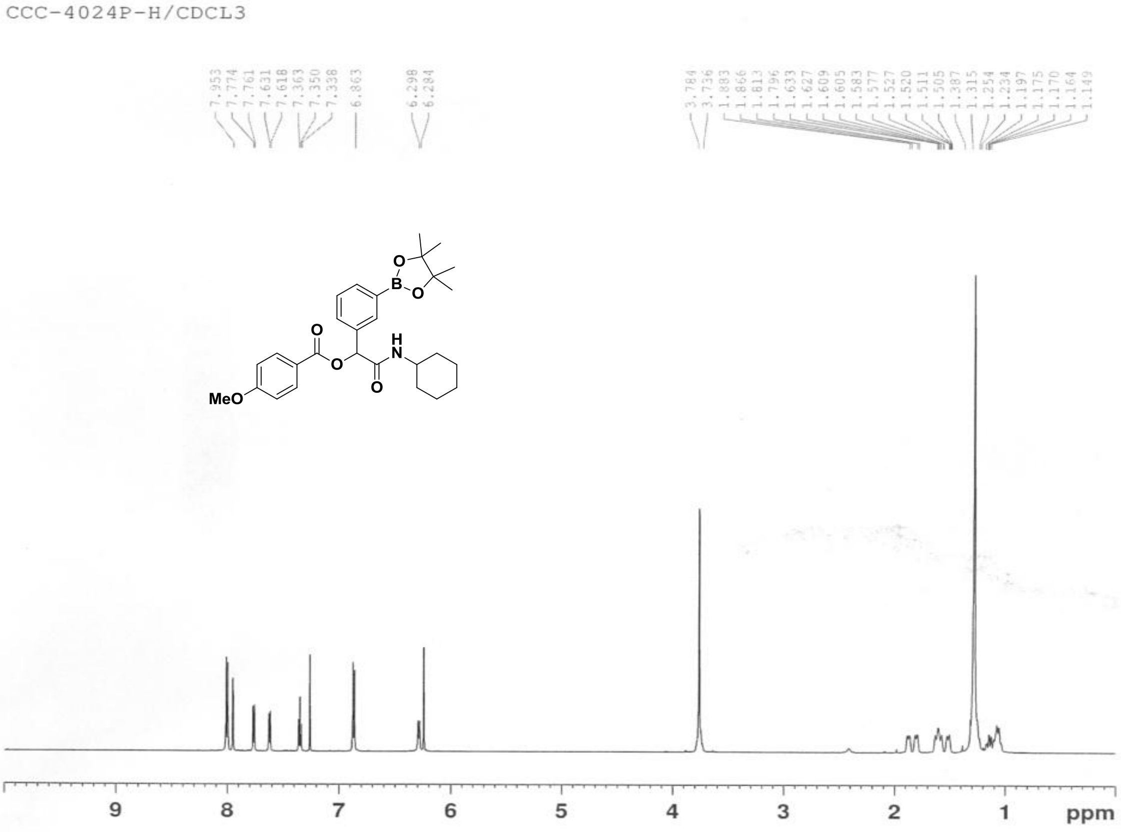

Figure S113. 600 MHz ^{13}C-NMR of Compound (**B8**) in Chloroform-*d*.

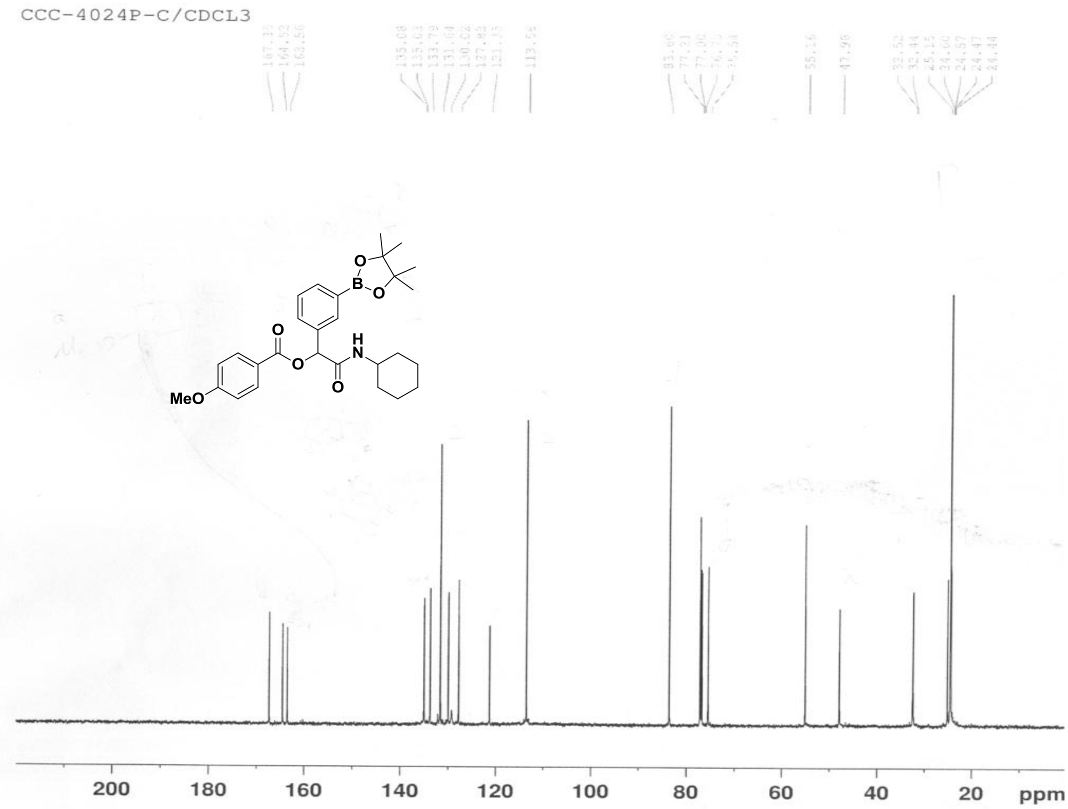

Figure S114. 600 MHz ^{11}B-NMR of Compound (**B8**) in Chloroform-*d*.

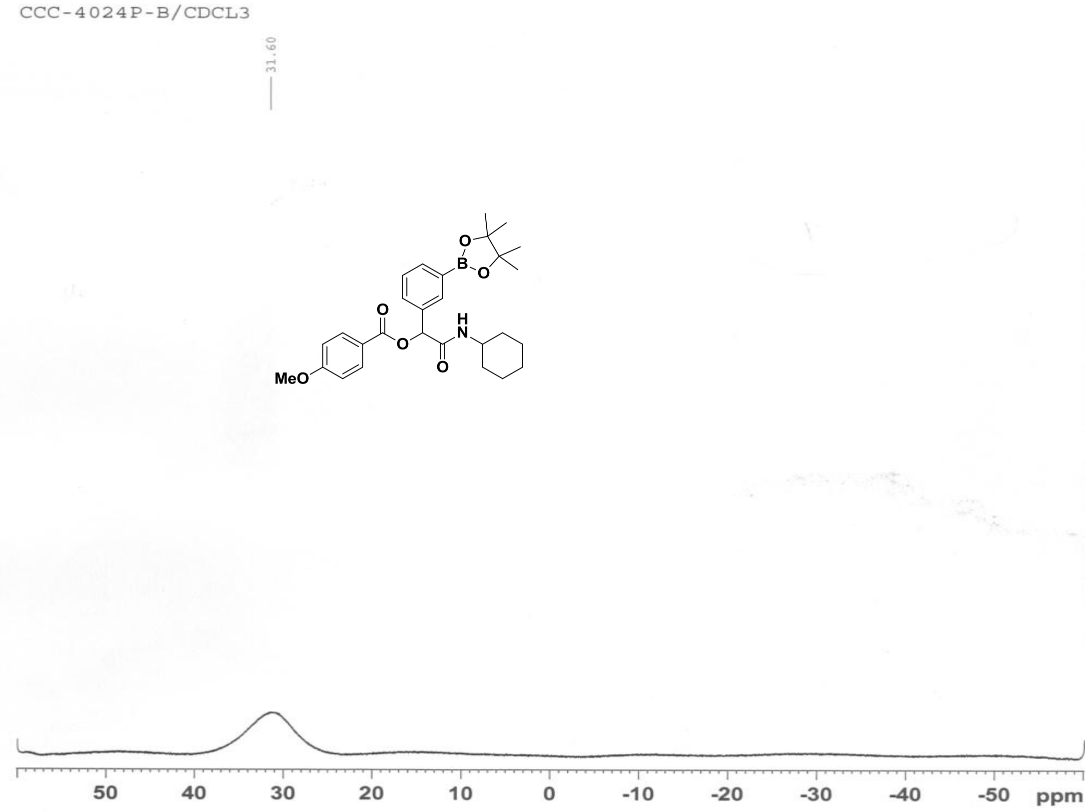

Figure S115. HRMS (ESI, positive ion) $[M+H]^+$ of Compound (**B9**).

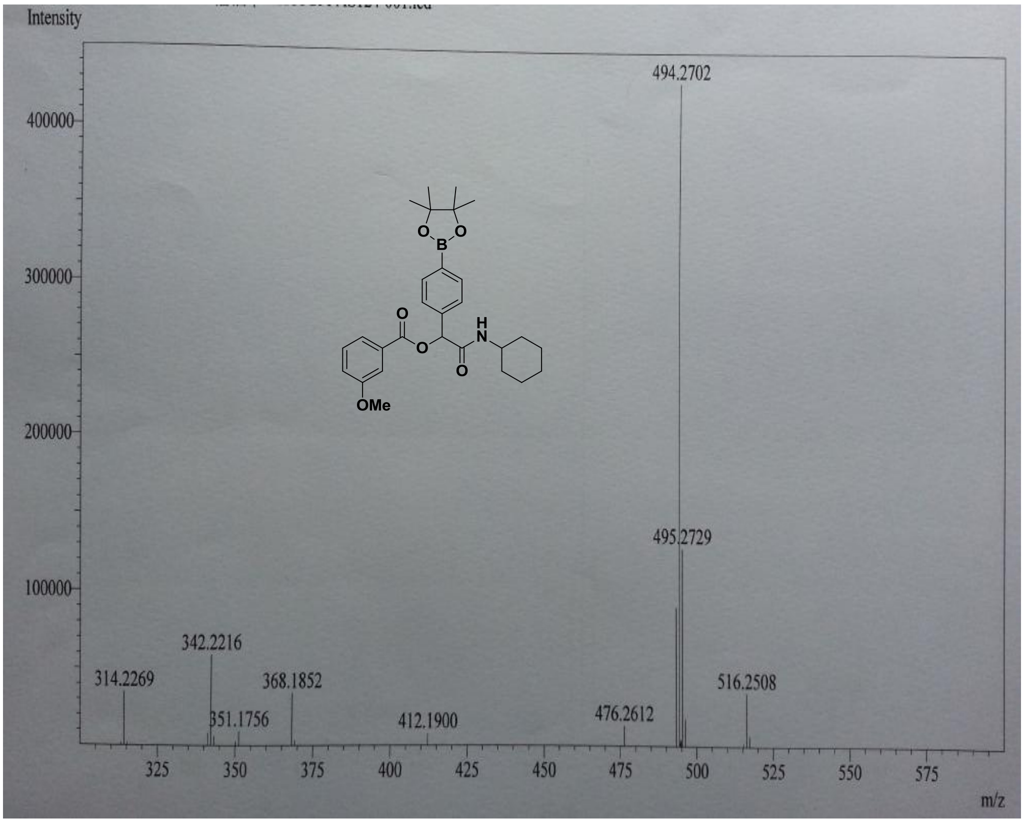

Figure S116. 600 MHz ^{1}H-NMR of Compound (**B9**) in Chloroform-*d*.

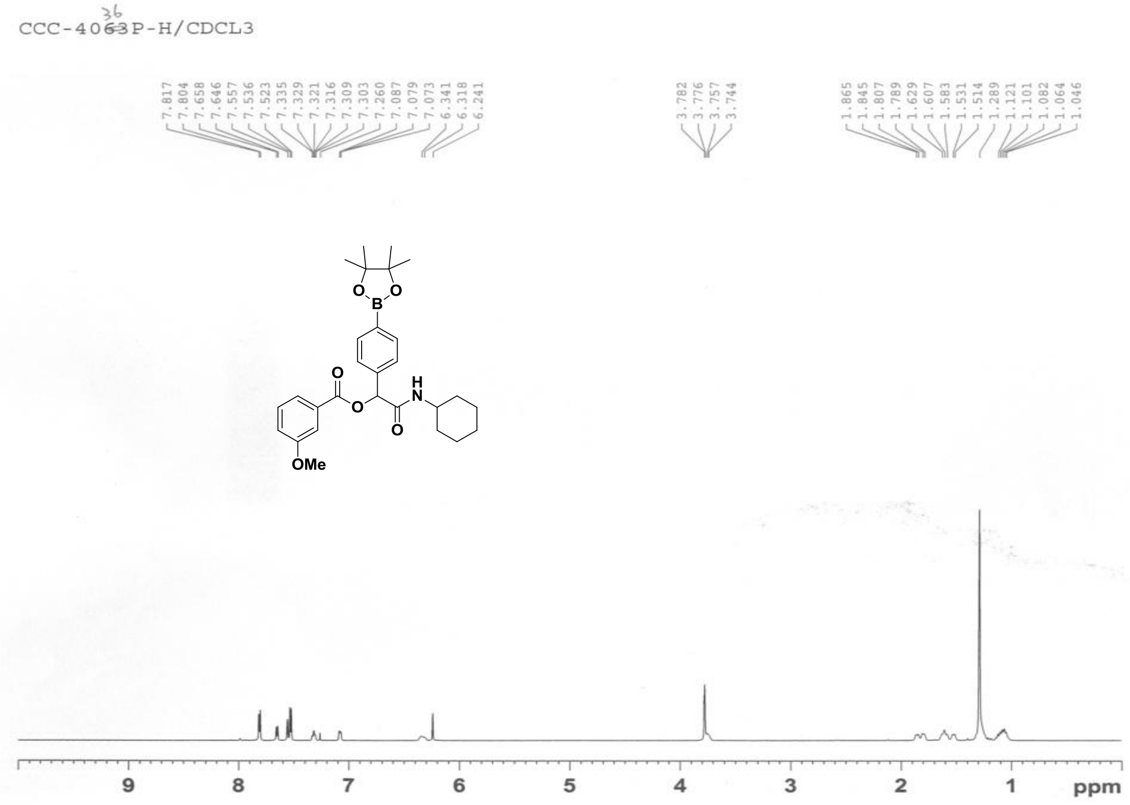

Figure S117. 600 MHz ^{13}C-NMR of Compound (**B9**) in Chloroform-*d*.

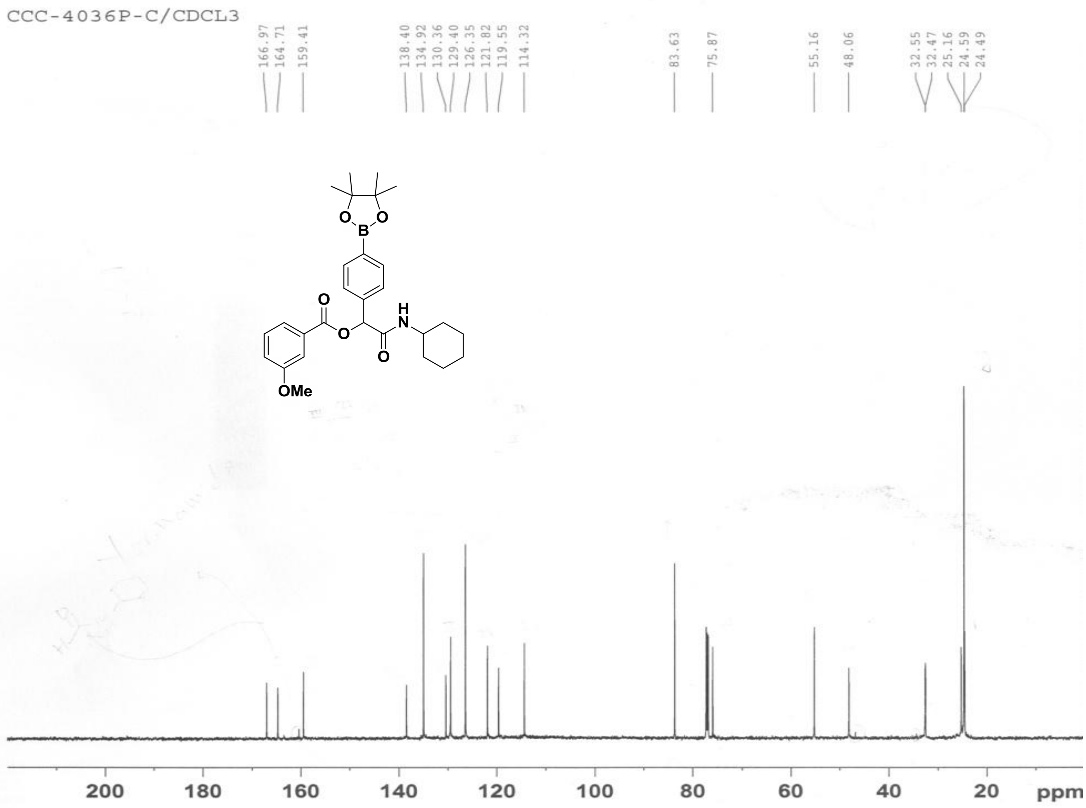

Figure S118. 600 MHz ^{11}B-NMR of Compound (**B9**) in Chloroform-*d*.

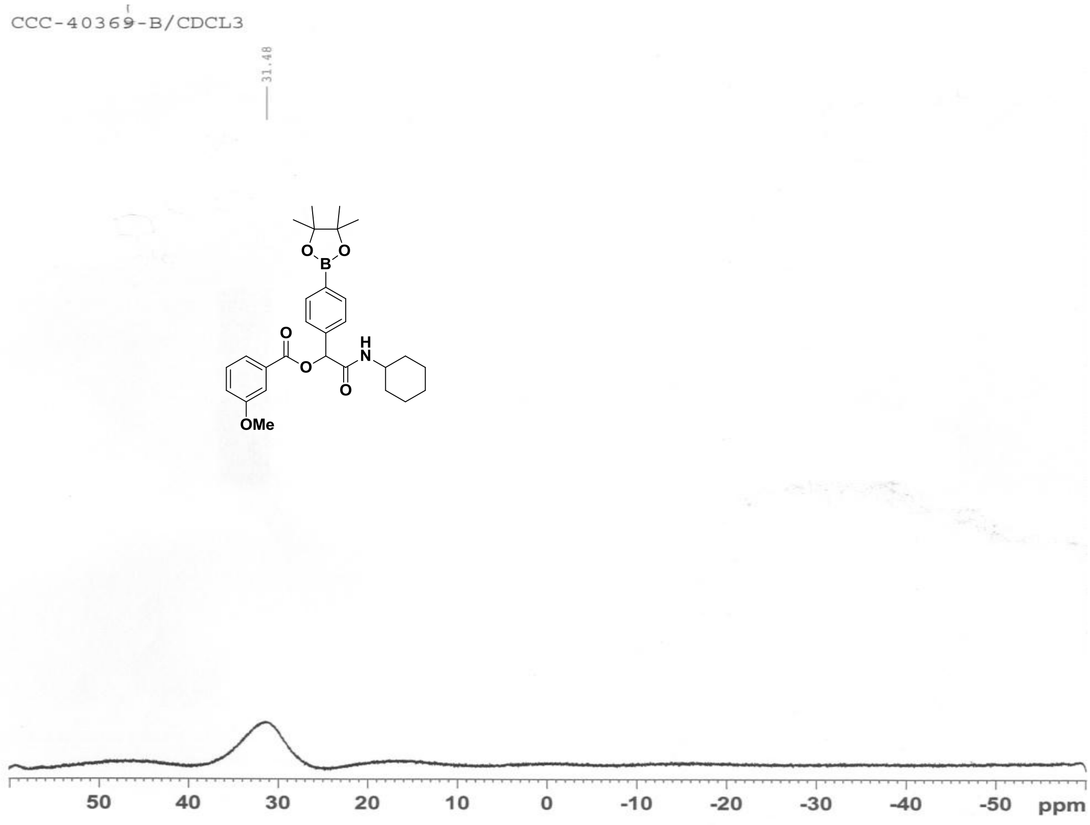

Figure S119. HRMS (ESI, positive ion) $[M+H]^+$ of Compound (**B10**).

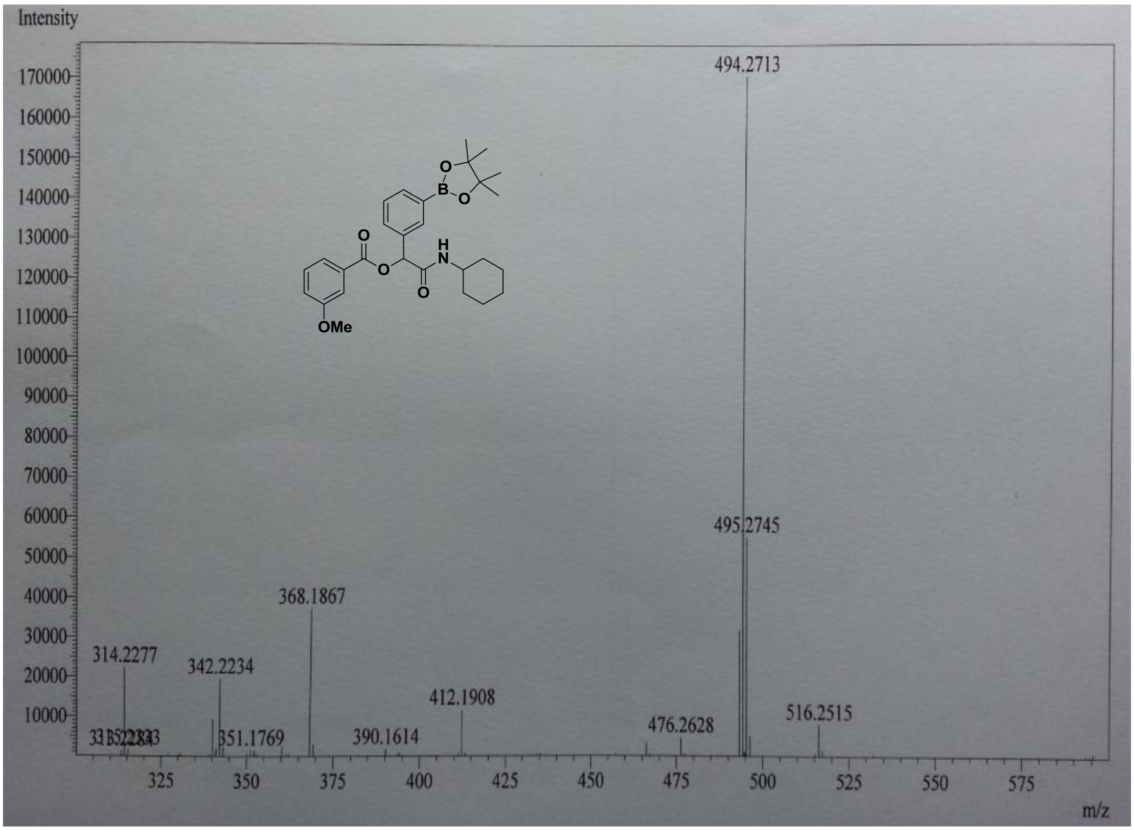

Figure S120. 600 MHz ^{1}H-NMR of Compound (**B10**) in Chloroform-*d*.

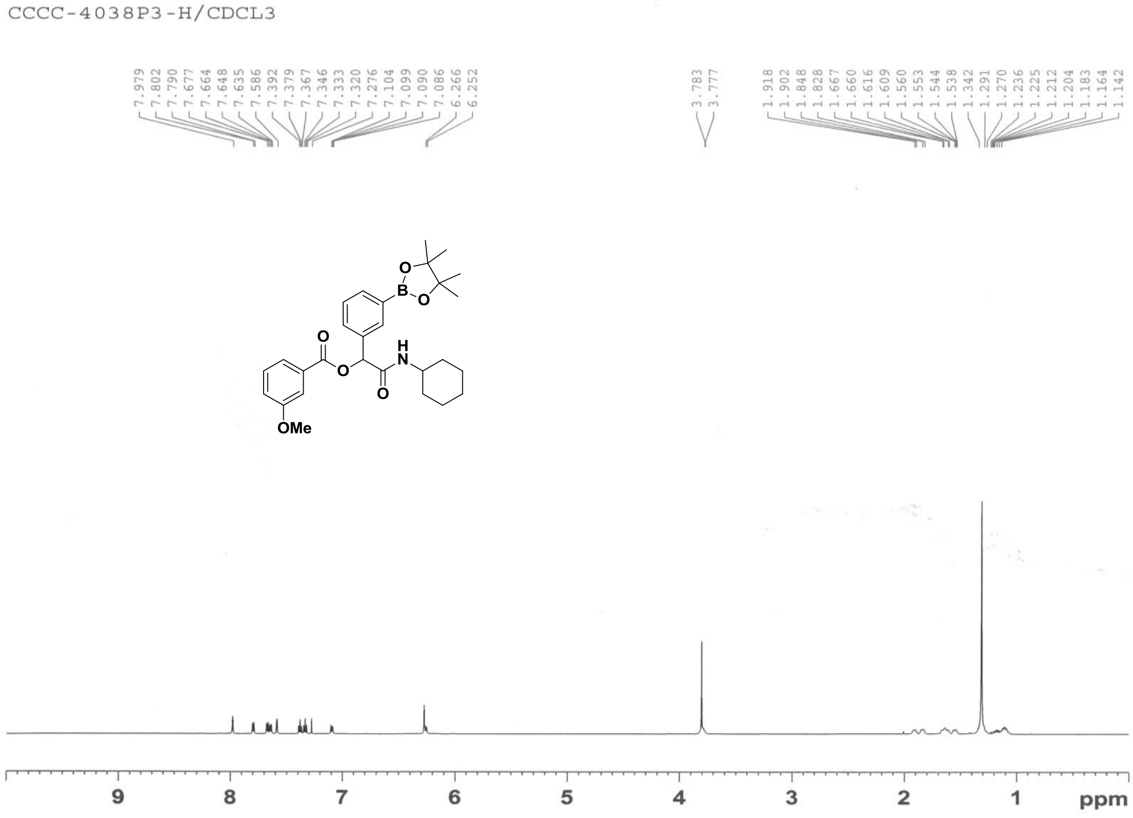

Figure S121. 600 MHz ^{13}C-NMR of Compound (**B10**) in Chloroform-*d*.

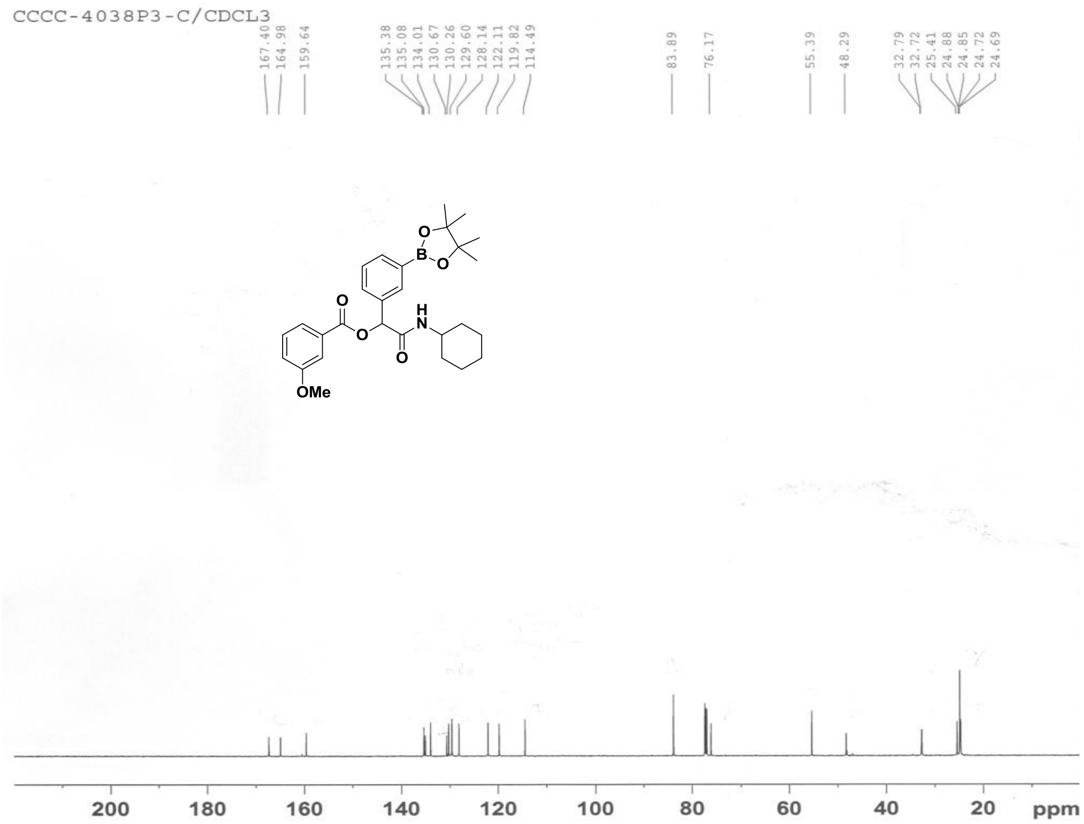

Figure S122. 600 MHz ^{11}B-NMR of Compound (**B10**) in Chloroform-*d*.

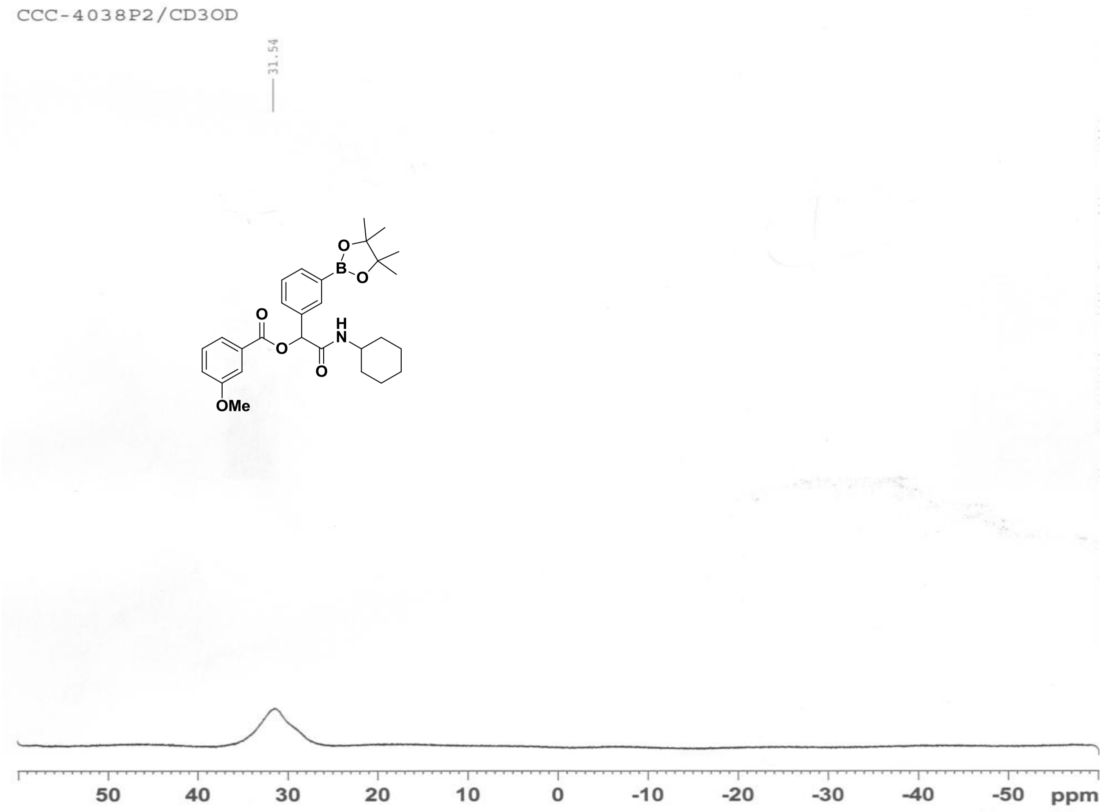

Figure S123. HRMS (ESI, positive ion) $[M+H]^+$ of Compound (**B11**).

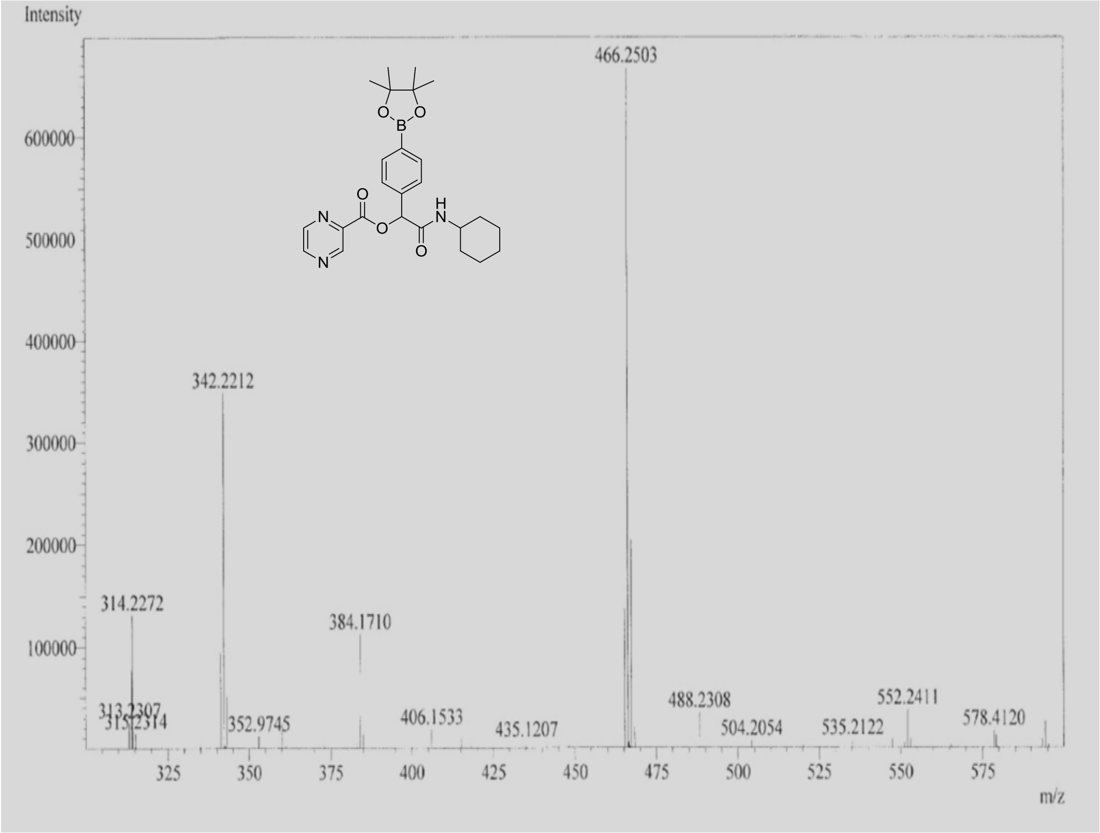

Figure S124. 600 MHz ^{1}H-NMR of Compound (**B11**) in Chloroform-*d*.

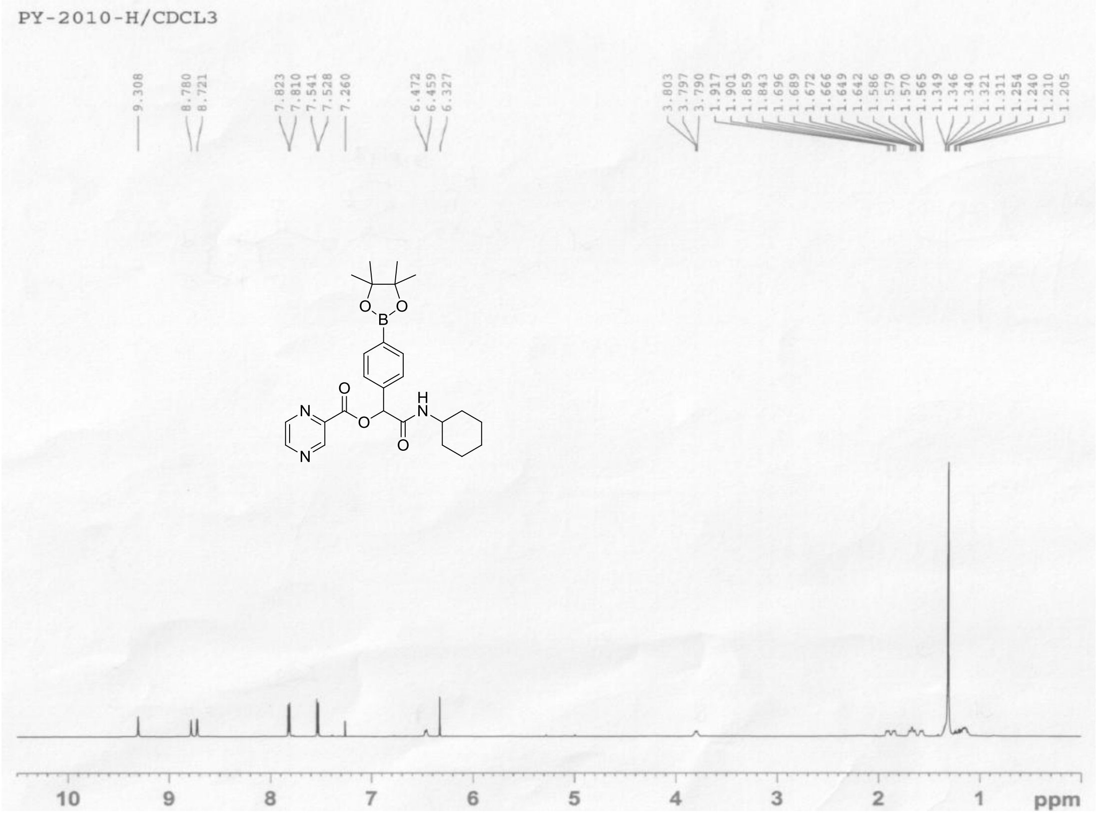

Figure S125. 600 MHz ^{13}C-NMR of Compound (**B11**) in Chloroform-*d*.

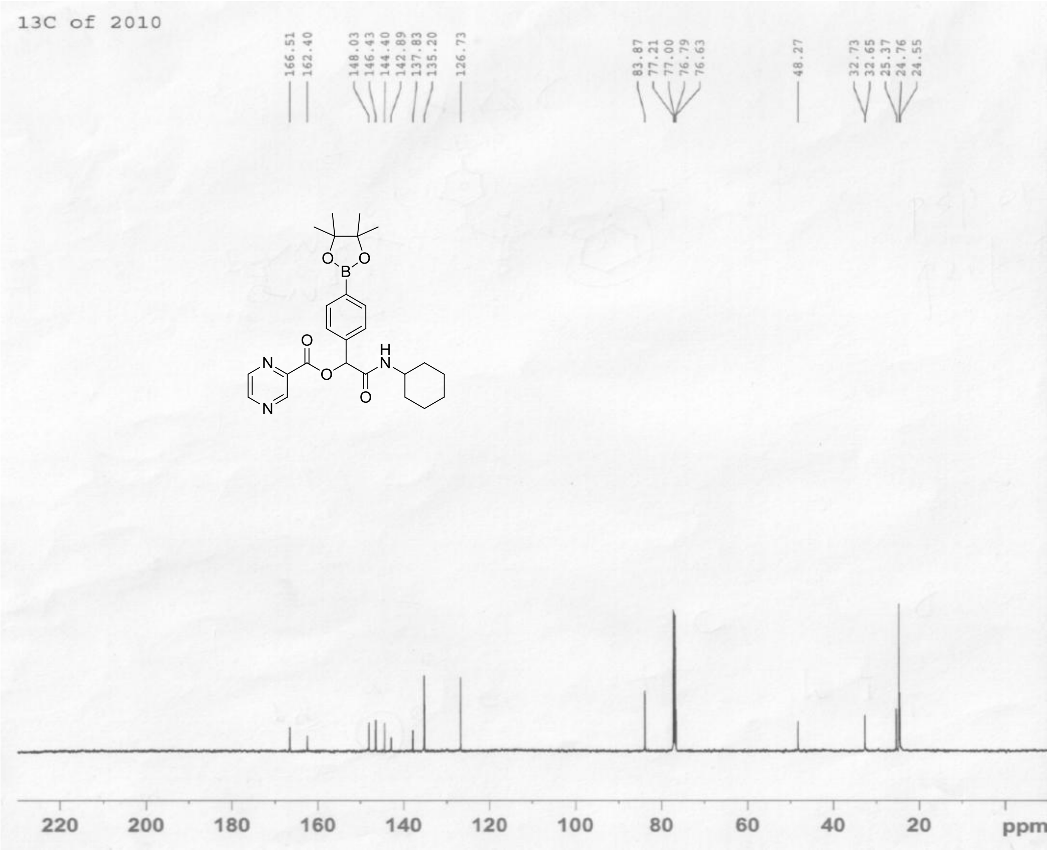

Figure S126. 600 MHz ^{11}B-NMR of Compound (**B11**) in Chloroform-*d*.

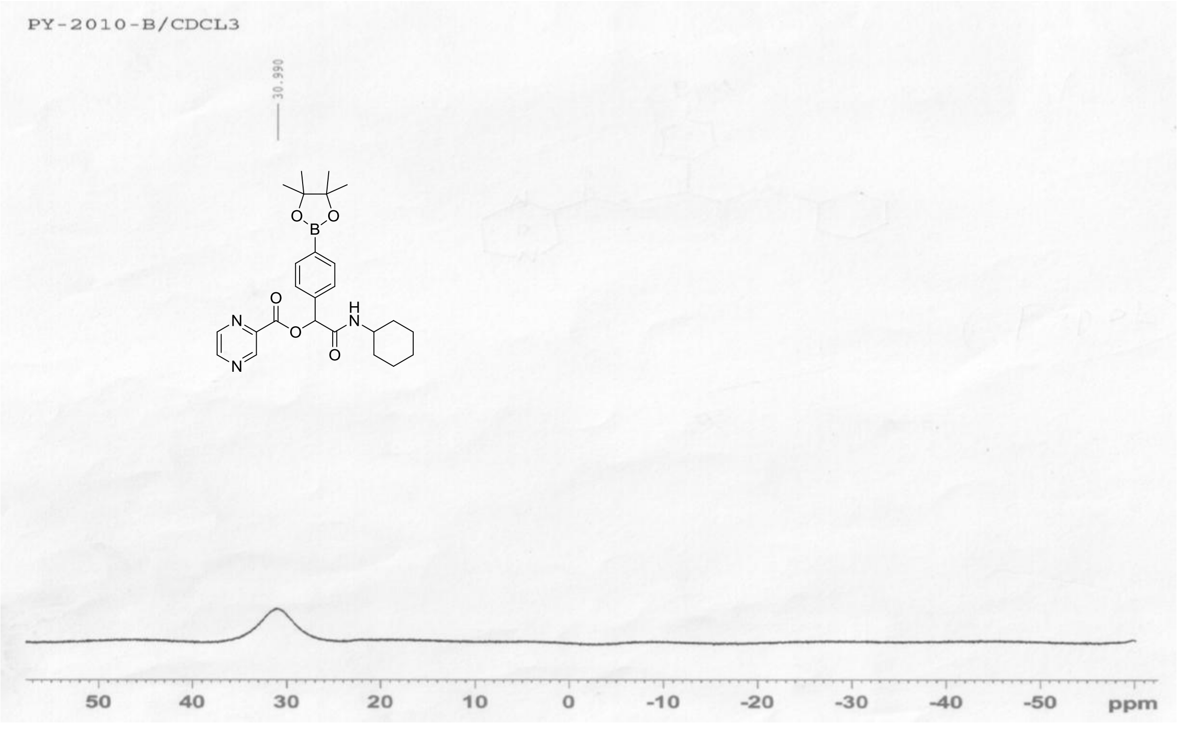

Figure S127. HRMS (ESI, positive ion) $[M+H]^+$ of Compound (**B12**).

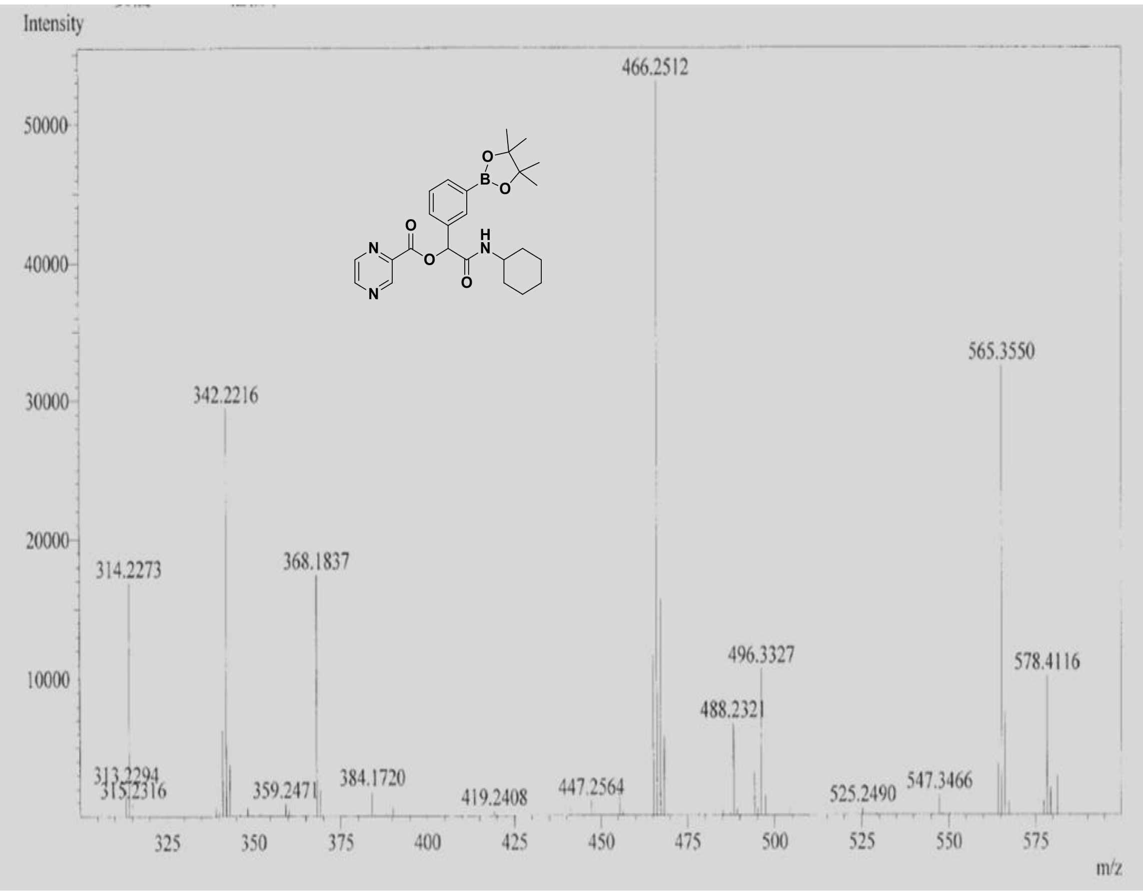

Figure S128. 600 MHz ^{1}H-NMR of Compound (**B12**) in Chloroform-*d*.

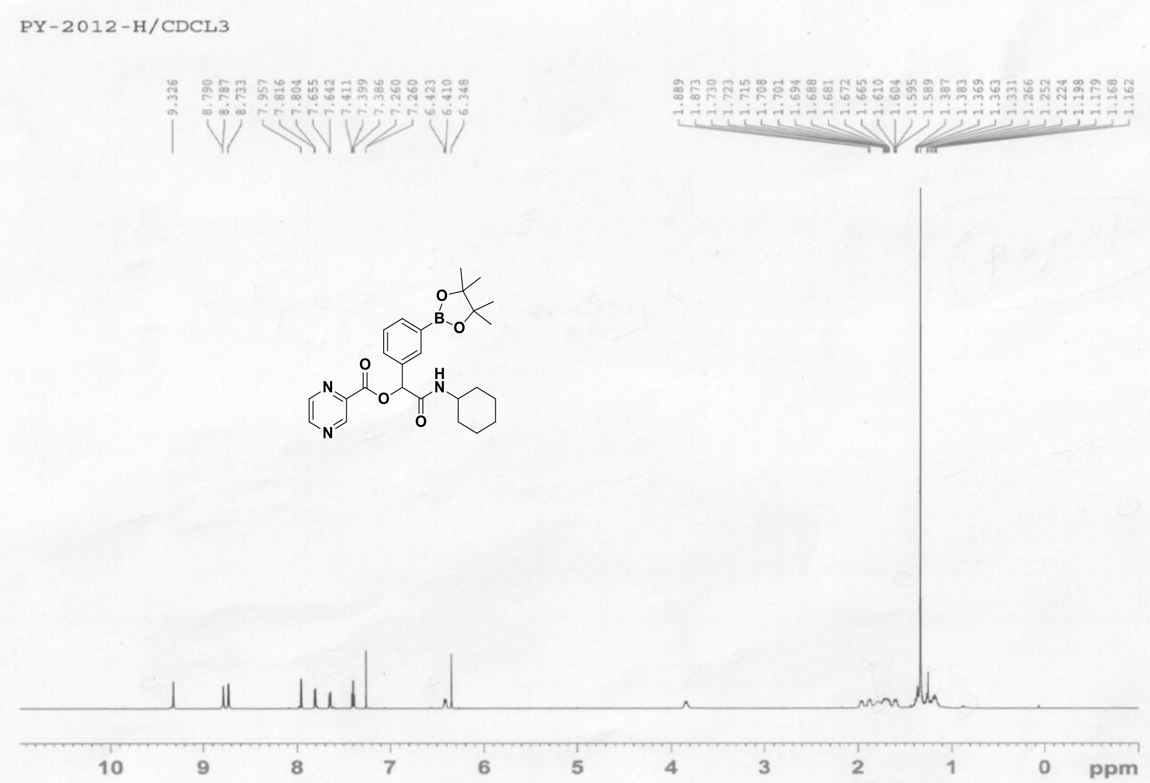

Figure S129. 600 MHz ^{13}C-NMR of Compound (**B12**) in Chloroform-*d*.

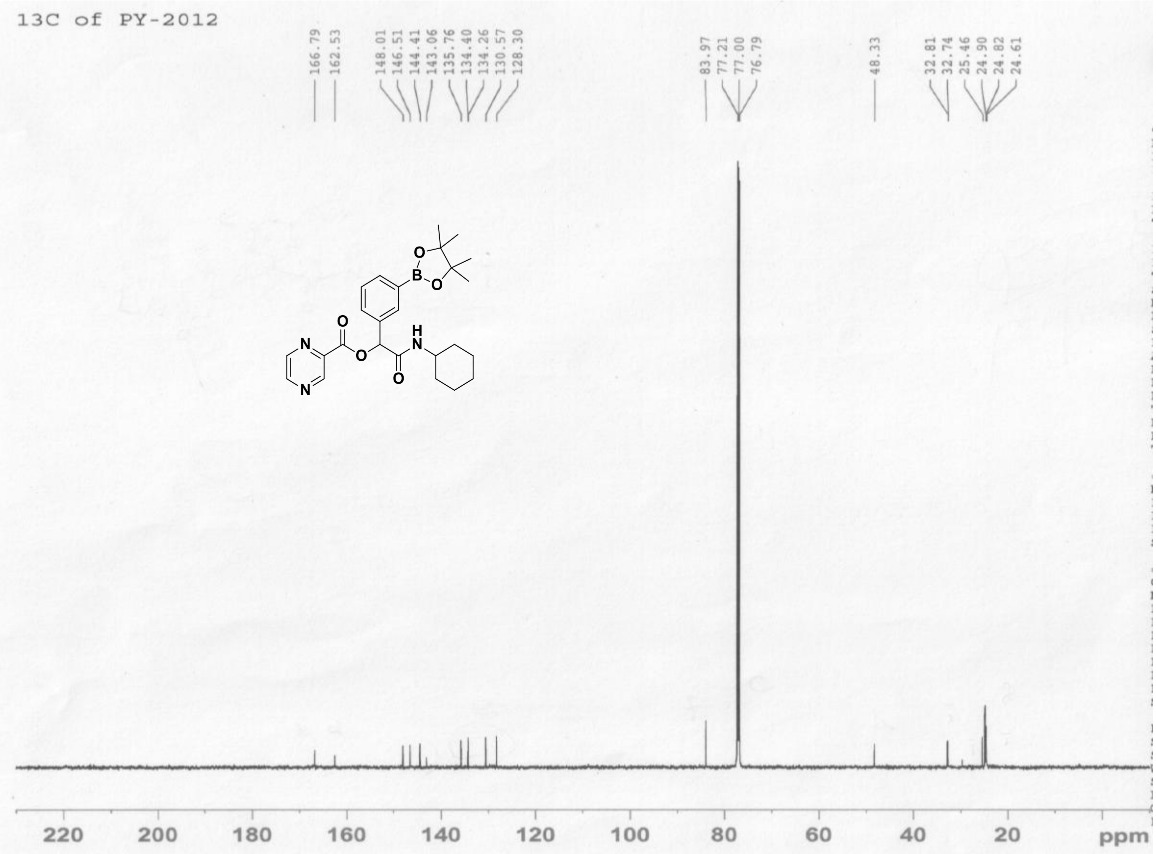

S130. 600 MHz ^{11}B-NMR of Compound (**B12**) in Chloroform-*d*.

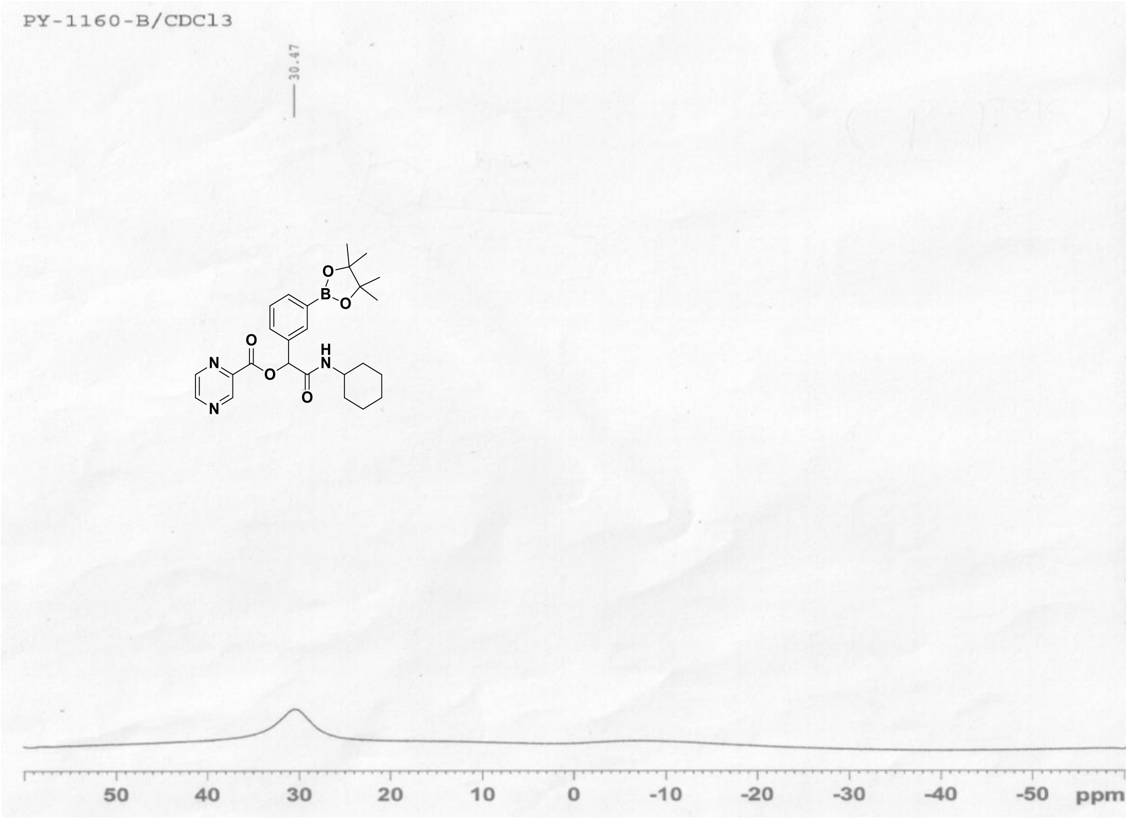

Supplement: Supplementary file 1 [file molecules-18-09488-s001.pdf]
